# The ancient function of RB-E2F Pathway: insights from its evolutionary history

**DOI:** 10.1186/1745-6150-5-55

**Published:** 2010-09-20

**Authors:** Lihuan Cao, Bo Peng, Lei Yao, Xinming Zhang, Kuan Sun, Xianmei Yang, Long Yu

**Affiliations:** 1 State Key Laboratory of Genetic Engineering, Institute of Genetics, School of Life Sciences, Fudan University, Shanghai 200433, PR China

## Abstract

**Background:**

The RB-E2F pathway is conserved in most eukaryotic lineages, including animals and plants. E2F and RB family proteins perform crucial functions in cycle controlling, differentiation, development and apoptosis. However, there are two kinds of E2Fs (repressive E2Fs and active E2Fs) and three RB family members in human. Till now, the detail evolutionary history of these protein families and how RB-E2F pathway evolved in different organisms remain poorly explored.

**Results:**

We performed a comprehensive evolutionary analysis of E2F, RB and DP (dimerization partners of E2Fs) protein family in representative eukaryotic organisms. Several interesting facts were revealed. First, orthologues of RB, E2F, and DP family are present in several representative unicellular organisms and all multicellular organisms we checked. Second, ancestral E2F, RB genes duplicated before placozoans and bilaterians diverged, thus E2F family was divided into E2F4/5 subgroup (including repressive E2Fs: E2F4 and E2F5) and E2F1/2/3 subgroup (including active E2Fs: E2F1, E2F2 and E2F3), RB family was divided into RB1 subgroup (including RB1) and RBL subgroup (including RBL1 and RBL2). Third, E2F4 and E2F5 share more sequence similarity with the predicted E2F ancestral sequence than E2F1, E2F2 and E2F3; E2F4 and E2F5 also possess lower evolutionary rates and higher purification selection pressures than E2F1, E2F2 and E2F3. Fourth, for RB family, the RBL subgroup proteins possess lower evolutionary rates and higher purification selection pressures compared with RB subgroup proteins in vertebrates,

**Conclusions:**

Protein evolutionary rates and purification selection pressures are usually linked with protein functions. We speculated that function conducted by E2F4/5 subgroup and RBL subgroup proteins might mainly represent the ancient function of RB-E2F pathway, and the E2F1/2/3 subgroup proteins and RB1 protein might contribute more to functional diversification in RB-E2F pathway. Our results will enhance the current understanding of RB-E2F pathway and will also be useful to further functional studies in human and other model organisms.

**Reviewers:**

This article was reviewed by Dr. Pierre Pontarotti, Dr. Arcady Mushegian and Dr. Zhenguo Lin (nominated by Dr. Neil Smalheiser).

## Background

The RB-E2F pathway is crucial for regulating cell cycle progression and tumorigenesis [1]. Proteins that are related to the retinoblastoma tumor suppressor RB and the E2F transcription factor are conserved in most eukaryotic lineages, including animals and plants [2].

The retinoblastoma susceptibility gene was the first tumor suppressor gene to be identified. RB-family members are generally believed to function through their effects on the transcription of genes regulated by the E2F proteins. In human, RB1 (pRb), RBL1 (p107), and RBL2 (p130) constitute a small RB family [1,2].

E2F proteins, which share a conserved DNA binding domain, can bind to overlapping sets of target promoters. In human, there are eight E2F genes (E2F1, E2F2, E2F3, E2F4, E2F5, E2F6, E2F7, and E2F8) [2-4]. E2F1-6 all possess one E2F-TDP domain, and E2F1, E2F2, and E2F3 are generally considered as the 'active E2Fs' on the basis of their ability to potently activate transcription. E2F4 and E2F5 are named as the 'repressive E2Fs', which bind their targets coincident with their repression in G0/G1, and only modestly activate transcription [3,4]. E2F1-5, combined with DP family proteins, can interact with the 'pocket protein' family protein (RB1, RBL1, and RBL2). DP family proteins, which contain one E2F-TDP domain and one DP domain, are dimerization partners of E2Fs. E2F6, also possessing one E2F-TDP domain but no RB binding domain, can not bind to RB family proteins [5].

E2F7 and E2F8 own two E2F-TDP domains, and can bind to DNA in the absence of interaction with a DP subunit. However, they lack sequences required for RB family protein binding [2-4].

We will use E2F1-6 family to refer to classic E2F proteins (E2F1, E2F2, E2F3, E2F4, E2F5 and E2F6), and E2F7/8 family to refer to E2F7 and E2F8 proteins in this manuscript.

During past two decades, a large number of studies, mainly conducted on flies, worms and vertebrates [2,6,7], have characterized the molecular properties and functions of RB-E2F pathway. It has been revealed that RB family proteins and E2F family proteins function in a wide range of biological processes, including DNA replication, mitosis, mitotic checkpoint, DNA-damage checkpoints, DNA repair, differentiation, development and apoptosis [2,6,7]. The functional conservation of RB-E2F pathway in different organisms (human, mouse, worm and fly) were found and reviewed [2]. Although, distributions of transcription factors including E2F, RB, DP proteins were reported in eukaryotic lineages recently [8], the detail evolutionary history of E2F family has not been explored. The evolutionary history of RB family had been investigated previously, but usually only with limited organisms [9,10].

Taking the advantage that more and more genomes had been completely sequenced, we probed the evolutionary history of RB-E2F genes in eukaryotic lineages. Totally, 21 representative eukaryotic organisms were selected for E2F, RB, and DP proteins identification. There are 16 organisms from metazoan (*Homo sapiens; Canis familiaris; Bos Taurus; Mus musculus; Rattus norvegicus; Gallus gallus; Xenopus tropicalis; Danio rerio; Tetraodon nigroviridis; Strongylocentrotus purpuratus; Branchiostoma floridae; Ciona intestinalis; Caenorhabditis elegans; Drosophila melanogaster; Nematostella vectensis; and Trichoplax adhaerens*), one choanoflagellate (*Monosiga brevicollis*), two plants *(Arabidopsis thaliana; Oryza sativa*), one social amoebae (*Dictyostelium discoideum AX4*), and one green algae(*Ostreococcus tauri*).

Previously, it was thought that RB-E2F pathway was missed in fungi [2], and it was true for yeast (such as *S. pombe *and *S. cerevisiae*). However, we found that some fungi own E2F family and DP family proteins, for example: *Encephalitozoon cuniculi*, belonging to microsporidia which are once thought to be protists but now known to be fungi [11], owns one E2F1-6 family protein (gi (|19074054), and one DP family protein (gi|19074276). However, no RB family protein is found in *E. cuniculi *and other Fungi, and so RB-E2F pathway might be not complete in fungi, we did not cover fungi proteins in detail analysis.

It is worth to mention that several reasons made some representative model organisms to be selected. The placozoan *T. adhaerens*, represents a primitive metazoan form, and is a basal eumetazoan lineage that diverged before the separation of cnidarians and bilaterians [12]. The sea anemone *N. vectensis *is a non-bilaterian animal, a member of the phylum Cnidaria [13]. The unicellular choanoflagellate (*M. brevicollis*) is the closest relatives of metazoans, which represents a distinct lineage that evolved before the origin and diversification of metazoans [14]. *D. discoideum AX4*, a soil amoeba, branched from the lineage that ultimately led to the metazoa before yeast but after plants, the social amoebae are exceptional in their ability to alternate between unicellular and multicellular forms [15]. *O. tauri *is a genus of unicellular coccoid or spherically shaped green alga, and is the smallest known free-living eukaryote [16].

In this study, we explored particularly, (1) the distribution of RB, E2F genes in eukaryotic lineages; (2) the details about gene duplication events and evolutionary history of RB, E2F genes in metazoa; (3) different evolution rates and selection pressures in subgroup proteins of RB, E2F family; (4) the function insights from the evolutionary history of RB-E2F pathway.

## Methods

### Protein Sequence identification

Using Human E2F4, DP1, E2F7, and RB protein as a query, we performed PSI-Blast searches (E-value less than e-5 as cut-off) at the National Center for Biotechnology Information (NCBI) Web site http://www.ncbi.nlm.nih.gov/ to screen the non-redundant protein database from 21 organisms [17]. All the new results were used as queries to carry out a second round of BLAST search, until no new sequence was found. BLASTP search was also performed in Ensembl database http://www.ensembl.org/ for above organisms. In addition, TBLASTN searches were also carried out at (NCBI) Web site. The collected protein sequences were then analyzed by SMART [18] and Pfam [19] for domain architecture.

As E2F1-6 family, E2F7/8 family, and DP family share some sequence similarity, all of them own the E2F-TDP domain, we use below criterions to classify them: all E2F1-6 members only have one E2F-TDP domains, and share more sequence similarity to human E2F4 than to human E2F7 or DP1; All E2F7/8 members have two E2F-TDP domains, and share more sequence similarity to human E2F7 than to human E2F4 or DP1; all DP family members have one E2F-TDP domain and one DP domain, and share more sequence similarity to human DP1 than to human E2F4 or E2F7. Some retrieved sequences were discarded on the basis of the following criteria: (1) partial sequences or sequences resulting from frameshifts in the underlying mRNA as a result of cloning artifacts or possible aberrant alternative splicing; (2) Protein sequences which did not contain almost the entire E2F-TDP domain or RB domain (3) duplicated database submissions of the same sequence; and alternatively spliced isoforms.

### Protein name used in this study

For proteins from *H. sapiens*, *M. musculus, C. elegans*, *D. melanogaster*, *A. thaliana*, and *O. sativa*, we named them by using their symbols in genbank. For proteins from other organisms, which are unnamed in genbank, we named them on the basis of their evolutionary relationship to human proteins. For all the proteins, we also added their organisms as suffix. Abbreviations of organism names can be found after the conclusions section.

### Phylogenetic analysis

Multiple alignments were performed by MUSCLE 3.6 [20] and Clustal X [21] with the default settings. Maximum likelihood (ML) trees were constructed by using PHYML V.2.4 [22], with 500 bootstrap resamplings and JTT setting, and Gamma parameter values were estimated from the data set using the Tree-Puzzle program [23]. Bayesian inference (BI) was performed using the MRBAYES (version 3) package [24], with a mixture of protein evolution models, fixed rate, 100,000 generations, sampling every 100th generation and discarding initial 25% trees.

### Prediction of Ancestral Protein Sequences

Gapped Ancestral Sequence Prediction program (GASP) [25] was used to predict ancestral sequences from phylogenetic trees and the corresponding multiple sequence alignments.

### Computing Protein distances and selection pressures

Pairwise distances between proteins were calculated in MEGA 4.0 [26] with the amino acid Poisson correction model, uniform rates among sites and lineages, pairwise deletion. The protein-coding DNA sequences were collected from genbank http://www.ncbi.nlm.nih.gov/ and Ensembl database http://www.ensembl.org/. The protein-coding DNA sequences were aligned based on their protein alignments at the web server http://www.bork.embl.de/pal2nal/[27]. The codeml program in PAML(3.0) [28] was used for estimating synonymous and nonsynonymous substitution rates (*Ka/Ks*, *Ka, Ks*) in pairwise comparisons of protein-coding DNA sequences with the Nei-Gojobori method [29]. For group average pairwise distance value comparisons, all pairwise distance values for all protein sequences in each subgroup were computed. Then average values of group pairwise distances were calculated. Pair Student's T Test was used as statistical evaluation. For group average *Ka/ks *value comparisons, all *Ka/ks *values for all protein-coding DNA sequences in each subgroup were first computed for further study.

## Results

### The origination and distribution of E2F1-6, E2F7-8, RB, and DP family proteins in Eukaryotes

**Figure 1:**
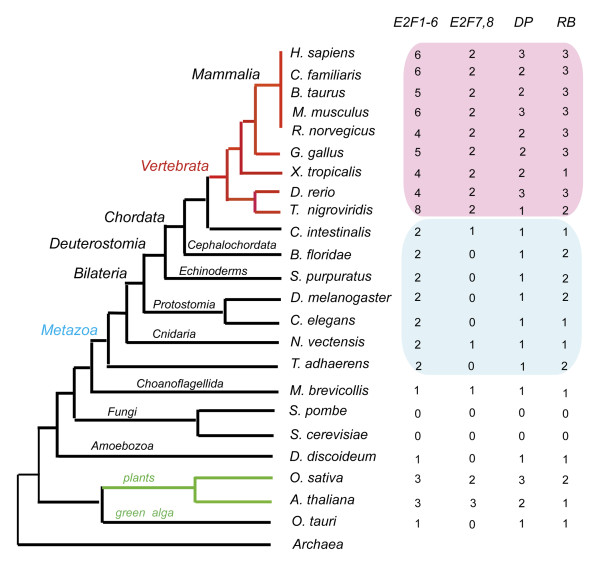
**Distribution of RB-E2F proteins in 21 representative Eukaryotic organisms**. The gene number of E2F1-6, E2F7/8, RB, and DP family in 20 representative Eukaryotic organisms were listed. *M. brevicollis, D. discoideum*, and *O. tauri *are unicellular organisms. The phylogenetic relationship of these organisms was drawn according to the results of proteome-based phylogeny [12,15]. The accession numbers and sequences of all these proteins are listed in additional File 1

Based on protein similarity searching and domain associations checking (Detail in material and methods section), E2F1-6, E2F7/8, RB, DP family proteins were identified in representative organisms. At last, 70 E2F1-6 family proteins, 25 E2F7/8 family proteins, 34 DP family proteins, and 41 RB family sequences were identified in 21 eukaryotic organisms (Figure 1). The accession numbers and sequences of all these proteins are listed in additional File 1.

We found that unicellular organisms, *D. discoideum AX4, O. tauri, and M. brevicollis *contain the representative orthologues of E2F1-6, DP, and RB family (figure 1). Based on the phylogenetic relationship among fungi and other organisms, the absence of RB and E2F genes in some fungi (such as *S. pombe and S. cerevisiae) *might due to gene losses in evolution. And as mentioned in the introduction section, fungi *E. cuniculi*, own E2F family protein (gi:19074054) and DP family proteins (gi:19074276), but no RB family protein.

For the multicellular organisms, we found that all multicellular lineages possess orthologues of E2F1-6, DP, and RB family. E2F7/8 family genes were present in protist *M. brevicollis*, animals, and plants. So E2F7/8 family originated before the separation of protists, animals, and plants. However, orthologues of E2F7/8 were not found in *D. melanogaster*, *C. elegans*, *B. floridae*, and *S. purpuratus*. We speculated that E2F7/8 family was not as indispensable as E2F1-6, RB, and DP family.

### Detailed Evolutionary relationship of the E2F, RB, and DP family in metazoa

Phylogenetic analysis for E2F, RB and DP proteins from representative eukaryotic organisms were carried out (results could be found in additional File 2). However, the higher sequence variation from distantly related eukaryotic organisms disrupted the global phylogenetic results, and made global phylogenetic trees not very robust and reliable. As our main interest was on the evolution of RB-E2F pathway in metazoa, the phylogenetic analyses on E2F, RB, and DP family in metazoan organisms were performed, and the results were discussed in detail.

### Evolution of E2F1-6 family, E2F7/8 family proteins in metazoa

**Figure 2:**
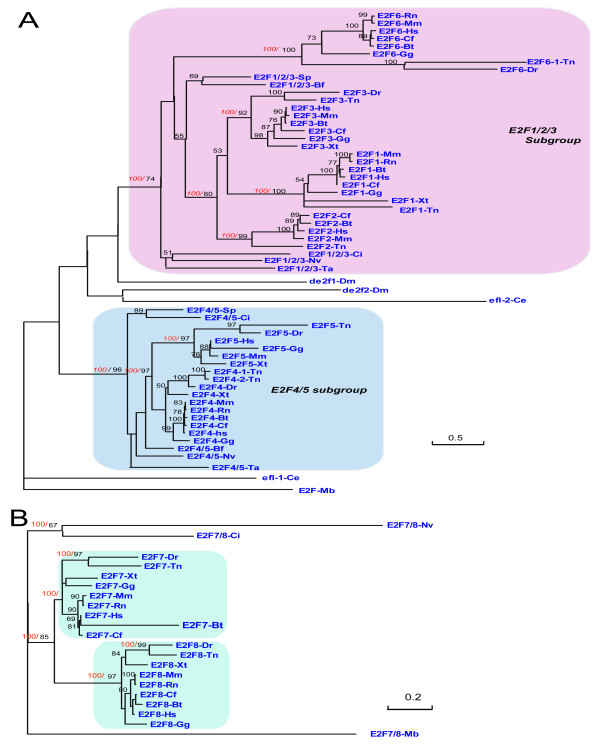
**Phylogenetic analyses of E2F1-6 and E2F7/8 family genes in metazoan**. Maximum likelihood analysis was conducted using Phyml program, and Bayesian analyses were carried out using MrBayes 3.1. Both methods produced nearly identical topologies. Black numbers above branches indicate ML bootstrap support (only greater than 50% values are labeled), and red numbers above branches indicate Bayesian posterior probabilities (only these key branches are labeled). The scale bar shows the number of substitutions per site. The detail information for protein names used in this figure can be found in materials and methods section. The accession numbers and sequence of all these proteins are listed in additional File 1. **(A) **Phylogenetic analyses of E2F1-6 family, and E2F-Mb (gi:167523471) was used as the outgroup; **(B) **Phylogenetic analyses of E2F7/8 family, and E2F7/8-Mb (gi:167517423) was used as the outgroup.

There are 61 E2F1-6 family proteins identified in 16 metazoan genomes (Figure 1). Both maximum likelihood method (ML) and Bayesian Inference method (BI) were employed for phylogenetic analyses, E2F-Mb (gi:167523471), the E2F protein from *M. brevicollis*, was used as an outgroup. Both methods produced nearly identical topologies (Figure 2).

In our analysis, E2F1-6 family proteins were divided into E2F4/5 subgroup and E2F1/2/3 subgroup with high statistical supports (Figure 2A). In vertebrates, the E2F1/2/3 subgroup could be again divided into two classes: E2F1/2/3-vert subgroup and E2F6 subgroup (Figure 2). The genbank accession numbers of proteins in the Figure 2 could be found in additional File 1. E2F1-6 family proteins in Figure 2A were also summarized in Table 1.

**Table 1:** Summary of the distribution and sub-grouping of E2F1-6 Family proteins in different organisms

	E2f1-6 family					
*M brevicollis*	E2F-Mb (gi|167523471)					

	**E2F1/2/3 subgroup**				E2F4/5 subgroup	

T. adhaerens	E2F1/2/3-Ta (gi|196010483)				E2F4/5-Ta (gi|196012606)	

*N. vectensis*	E2F1/2/3-Nv (gi|156371340)				E2F4/5-Nv (gi|156368461)	

*C. elegans*					EFL-1-Ce (gi|17559226)	

*D.me lanogaster*	E2F1-Dm (gi|24648770)				E2F2-Dm (gi|17137542)	

*S. purpuratus*					E2F4/5-Sp (gi|115696783)	

*C. intestinalis*	E2F1/2/3-Ci (gi|118343729)				E2F4/5-Ci (gi|118343737)	

*B. floridae*	E2F1/2/3-Bf (gi|260790430)				E2F4/5-Bf (gi|260798626)	

	**E2F1**	**E2F2**	**E2F3**	**E2F6**	**E2F4**	**E2F5**

*T. nigroviridis*	ENSTNIT00000007876	ENSTNIT00000020634	ENSTNIT00000012687	ENSTNIT00000008935 ENSTNIT00000013829	ENSTNIT00000002389 ENSTNIT00000002758	ENSTNIT00000004791

*D. rerio*			gi|220673319	gi|71892405	gi|47087407	gi|68533607

*X. tropicalis*	ENSXETT00000046332		gi|58331835		gi|167560905	gi|188528909

*G. gallus*	gi|45382583		gi|118086362	ENSGALT00000031596	gi|118096144	gi|71896455

*R. norvegicus*	gi|189217865		gi|212549627	gi|109479090	ENSRNOT00000021145	

*M. musculus*	gi|6681243	gi|29244208	gi|83523736	gi|237681138	gi|22507329	gi|31982405

*B. taurus*	gi|194672360	gi|76611569	gi|76663083	gi|116003911	gi|115497534	

*C. familiaris*	gi|73992245	ENSCAFT00000021059	gi|74004128	gi|73980432	gi|73957515	gi|73999542

*H. sapiens*	gi|12669911	gi|4758226	gi|4503433	gi|109637795	gi|12669915	gi|134142811

According to results from Figure 2A, E2F1-6 family proteins (accession numbers from genbank or Ensembl database) identified in *M. brevicollis *and 16 metazoan organisms were listed. The detail explains could be found in main text

It was found that the placozoan, *T. adhaerens*, owns bone fide member E2F1/2/3-Ta (gi:196010483) in E2F1/2/3 subgroup, and E2F4/5-Ta (gi:196012606) in E2F4/5 subgroup. Non-bilaterian animal sea anemone (*N. vectensis*), bilaterian invertebrates *S. purpuratus, B. floridae*, and *C. intestinalis *all possess their representative genes in E2F1/2/3 subgroup and E2F4/5 subgroup (Figure 2, Table 1). This indicated that the first gene duplication for E2F1-6 family happened before placozoans and bilaterians separation.

In vertebrates, the number of E2F1-6 family member dramatically increased. All human E2F1-6 family proteins could found their direct orthologues in *T. nigroviridis *(Figure 2, Table 1), indicating all human E2F1-6 family proteins emerged in early vertebrates, and gene duplications in E2F4/5 subgroup and E2F1/2/3 subgroup happened before tetrapoda and teleostei divergence (Figure 2, Table 1). In detail, our results demonstrated that human E2F1, E2F2, E2F3 and E2F6 emerged by gene duplications of the common E2F1/2/3 subgroup ancestor. And E2F1/2/3 subgroup members from invertebrates *T. adhaerens*, *S. purpuratus, B. floridae*, and *C. intestinalis*, which we named them as E2F1/2/3-Ta, E2F1/2/3-Sp, E2F1/2/3-Ci, and E2F1/2/3-Bf, should be recognized as the co-orthologues of human E2F1, E2F2, E2F3 and E2F6 genes. Human E2F4 and E2F5 emerged by gene duplications of the common E2F4/5 subgroup ancestor. And E2F4/5 subgroup members from invertebrates *T. adhaerens*, *N. vectensis, S. purpuratus, B. floridae*, and *C. intestinalis*, which we named them as E2F4/5-Ta, E2F4/5-Nv, E2F4/5-Sp, E2F4/5-Bf, E2F4/5-Ci, should be recognized as the co-orthologues of human E2F4 and E2F5 genes. It needs to be mentioned that though E2F6 emerged together with E2F1, E2F2 and E2F3 from E2F1/2/3 subgroup ancestor, it lost the RB binding domain and did not function in classical RB-E2F pathway [2], and we will not discuss it later.

Species-specific gene duplications and gene losses were also found in E2F1-6 family in vertebrates. *H. sapiens*, *C. familiaris, M. musculus *all own 6 members of E2F1-6 family, *T. nigroviridis *has *8 *members of E2F1-6 family, with two E2F4 (E2F4-1-Tn(ENSTNIT00000002389), E2F4-2-Tn (ENSTNIT00000002758)) and two E2F6(E2F6-1-Tn(ENSTNIT00000008935), E2F6-2-Tn(ENSTNIT00000013829)). There are 5 members of E2F1-6 family in *B. Taurus*, with E2F5 orthologue absent; 4 members in *R. norvegicus*, with E2F2 and E2F5 orthologues absent; 5 members in *G. gallus*, with E2F2 orthologue absent; 4 members in *X. tropicalis*, with E2F2, E2F6 orthologues absent; 4 members in *D. rerio*, with E2F1 and E2F orthologues absent (Figure 2, Table 1).

It is a little surprise that so many gene losses happened in the E2F1-6 family in vertebrates. However, we found that all vertebrates have at least one member from E2F1/2/3 subgroup, and also one member from E2F4/5 subgroup (Figure 2, Table 1).

The ancestral E2F gene duplication happened before placozoans and bilateria separation. Therefore, bilateria *D. melanogaster *and *C. elegans *should theoretically have representative orthologues of E2F4/5 subgroup and E2F1/2/3 subgroup. However, in our analysis, dE2F1-Dm, dE2f2-Dm, efl-1-Ce, and efl2-Ce all failed to be clustered into E2F1/2/3 subgroup or E2F4/5 subgroup, possibly due to their high sequence divergence.

**Figure 3:**
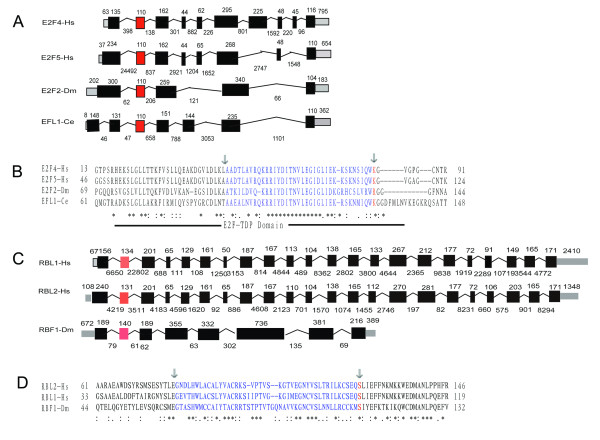
**Conservation of exon intron structures in related genes from human, fly, and worm**. The exon and intron structures of related genes from human, fly, and worm are showed, **(A): **E2F1-6 family, **(C)**: RB family, and Protein sequences alignments of the region with interspecies conserved exons are also showed, **(B)**: E2F1-6 family, **(D)**: RB family. Boxes correspond to exons. Non-coding exons are shown in grey. The interspecies conserved exons are labeled with red color. The size of introns and exons in nucleotides is shown. Introns and non-coding regions are not drawn to scale. In the alignment, the protein sequences coded by interspecies conserved exons are labeled with blue color, and amino acid residues overlap splice sits are labeled with red color. The exon intron structures information are got from ensemble data base, detail of transcripts used for analysis are: E2F4-Hs: ENST00000379378; E2F5-Hs: ENST00000416274; E2F2-Dm: FBtr0081501; EFL1-Ce: Q9XX87_CAEEL (Y102A5C.18); RBL1-Hs: ENST00000373664; RBL2-Hs: ENST00000262133; RB1-Hs: ENST00000267163; RBF1-Dm: FBtr0070146. The exon intron structures of all E2F1-6 and RB Family genes from human, fly, and worm can be found in additional file 6.

In the further analysis (such as Blast), we found that dE2F2-Dm, EFL-1-Ce share more sequence similarity with mammalian E2F4, E2F5 than with E2F1, E2F2, E2F3. In contrast, dE2F1-Dm shares more sequence similarity with E2F1, E2F2, E2F3 than with E2F4, E2F5. In addition, conversation of exon-intron structure for dE2F2-Dm, EFL-1-Ce, human E2F4, and E2F5 gene (Figure 3, additional file 3) was also found. We speculated that dE2F2-Dm and EFL-1-Ce belong to E2F4/5 subgroup, while dE2F1-Dm belong to E2F1/2/3 subgroup. And this classification was also consistent with results of previous functional studies which indicated that EFL-1-Ce and E2F2-Dm usually functioned as repressive E2Fs, while E2F1-Dm functioned as active E2Fs [2]. For EFL-2-Ce, as no function was reported and its sequence is highly diverged, we have no cues whether EFL-2-Ce is a product of species-specific duplication from EFL-1-Ce or a function lost gene which early derived from E2F1/2/3 subgroup. And we will not discuss it later. In anyway, the representative functional gene from E2F1/2/3 subgroup was absent in worm.

For E2F7/8 family (Figure 2), as mentioned in previous section, E2F7/8 family originated in early eukaryotes. However, orthologues of E2F7/8 subgroup were absent in *S. purpuratus*, *B. floridae, C. elegans*, and *D. melanogaster *(Figure 2B, Table 2). For E2F7/8 family, the first gene duplication happened before tetrapoda and teleostei divergence. By the way, protein E2F8-Xt (ENSXETG00000004436), which did not have the first E2F-TDP domain, and only have the second E2F-TDP domain, was not covered in our evolutionary analysis.

**Table 2:** Summary of the distribution and sub-grouping of RB Family, E2F7/8 family and DP family proteins in different organisms

	RB family			E2F7/8 Family		DP Family	
*M. brevicollis*	RB-Mb (gi:167523296|)			E2F7/8-Mb (gi|167517423)		DP-Mb (gi|167516980)	

	**RB1 subgroup**	**RBL subgroup**					

*T. adhaerens*	RB1-Ta (gi|196012646)	RBL-Ta (gi|196011866)				DP-Ta (gi|196015549)	

*N. vectensis*		RBL-Nv (gi|156399369|)		E2F7/8-Nv (gi|156344376)		DP-Nv (gi|156375187)	

*C. elegans*	LIN35-Ce (gi|17508261)					DPL-1-Ce (gi|17532739)	

*D. melanogaster*	RBF1-Dm (gi|24638969);	RBF2-Dm (gi17737995)				DP-Dm (gi|17136994)	

*S. purpuratus*	RB1-Sp (gi|115660767)	RBL-SP (gi|115968799)				DP-Ce (gi|72048148)	

*C. intestinalis*		RBL-Ci (gi|198437827)		E2F7/8-Ci (gi|198432739)		DP-Ci (gi|118343721)	

*B. floridae*	RB1-Bf (gi|260800656)	RBL-Rf (gi|260834765)				DP-Bf (gi|260816838)	

	**RB1**	**RBL1**	**RBL2**	**E2F7**	**E2F8**	**DP1**	**DP2**

*T. nigroviridis*	ENSTNIT00000014938	ENSTNIT00000009683		ENSTNIT00000007956	ENSTNIT00000000955	ENSTNIT00000017108	

*D. rerio*	gi|118150572	gi|194578849	gi|189537876	gi|169234759	gi|189521060	gi|41152118 gi|189523624	gi|38016161

*X. tropicalis*		gi|213982839		ENSXETT00000031323	ENSXETG00000004436	gi|58332126	gi|166158068

*G. gallus*	gi|45383327	gi|118100471	gi|118123617	gi|118082443	gi|118091079	gi|50730508	gi|118095071

*R. norvegicus*	gi|109501744	gi|109469134	gi|13592041	gi|157821575	gi|109462000	gi|71043770	gi|157818883

*M. musculus*	gi|188528630	gi|213417847	gi|170932488	gi|40254337	gi|67972650	gi|6678305	gi|182765448: gi|149260219

*B. taurus*	gi|116004031	gi|119905907	gi|148225699	gi|194666654	gi|194679774	gi|115496726	ENSBTAP00000008169

*C. familiaris*	gi|73989274	gi|73992372	gi|73949852	gi|73978133	gi|73988917	gi|73989562	gi|73990311

*H. sapiens*	gi|108773787	gi|34577079	gi|172072597	gi|145580626	gi|38505226	gi|6005900| gi|189409125	gi|5454112

According to the results from Figure 3 and Figure 2, RB family, E2F7/8 family and DP family proteins (accession numbers from genbank or Ensembl Database) identified from *M. brevicollis *and 16 metazoan organisms were listed. The detail explains could be found in main text.

### Evolution of RB family and DP family proteins in metazoa

**Figure 4:**
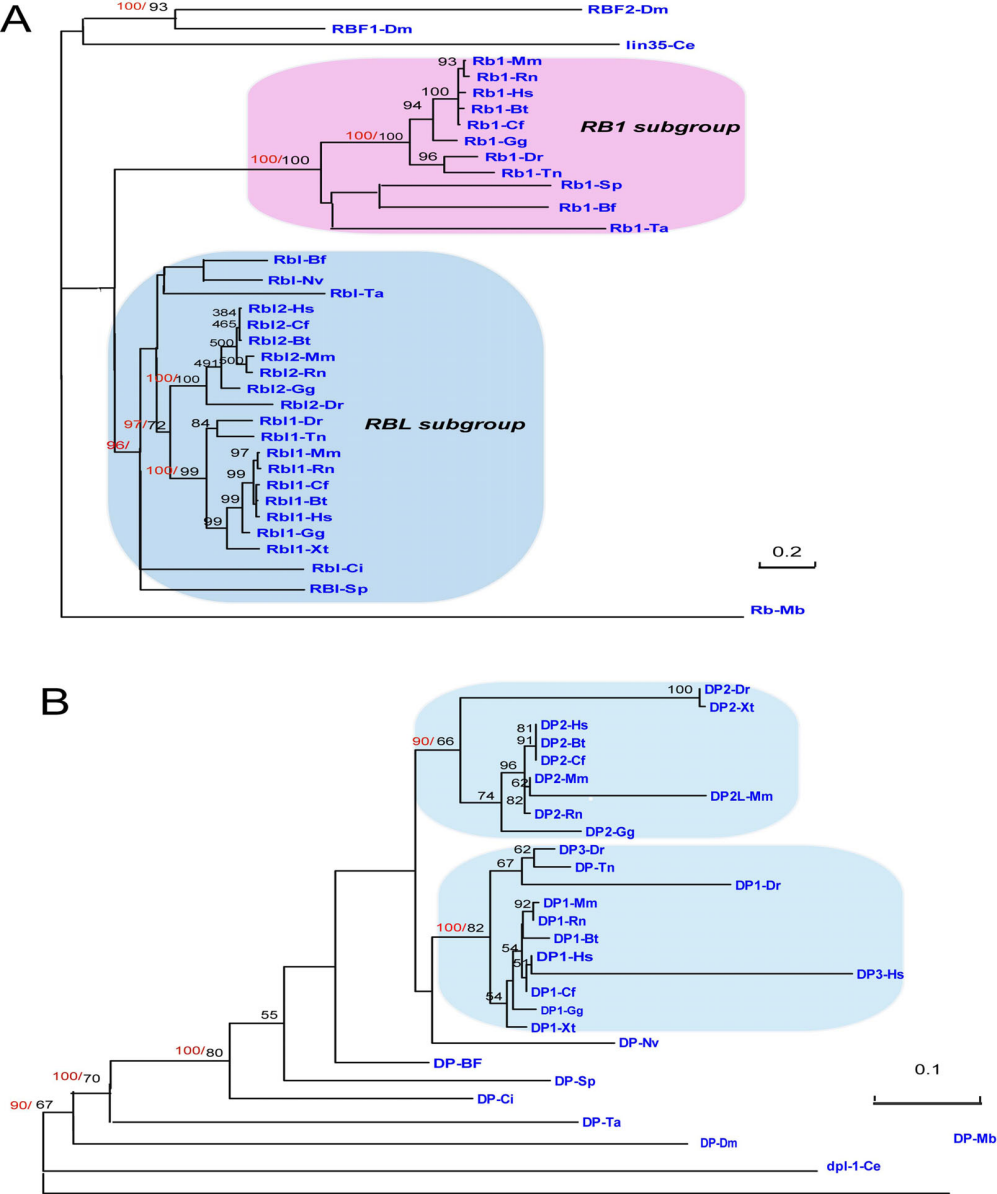
**Phylogenetic analyses of RB and DP family genes in metazoan**. Maximum likelihood analysis was conducted using Phyml program, and Bayesian analyses were carried out using MrBayes 3.1. Both methods produced nearly identical topologies. Black numbers above branches indicate ML bootstrap support (only greater than 50% values are labeled), while red numbers above branches indicate Bayesian posterior probabilities (only these key branches are labeled). The scale bar shows the number of substitutions per site. The detail information for protein names used in this figure can be found in materials and methods section. The accession numbers and sequence of all these proteins are listed in additional File 1. **(A) **Phylogenetic analyses of RB family, with RB-Mb (gi|167523296|) being used as the outgroup; **(B) **Phylogenetic analyses of DP family, with DP-Mb (gi:167516980) being used as the outgroup.

There are 36 RB family proteins identified in 16 metazoan genomes (Figure 1). Both ML method and BI method were employed for phylogenetic analyses, RB-Mb (gi:167523296), the RB family protein from *M. brevicollis*, was used as an outgroup. Both methods produced nearly identical topologies (Figure 4). All the RB family proteins in Figure 4 were also summarized in Table 2.

RB family proteins were divided into RB1 subgroup and RBL subgroup with high statistical supports in metazoa (Figure 4). The placozoan, *T adhaerens*, owns bone fide member RB1-Ta (gi:196012646) in RB1 subgroup, and RBL-Ta ((gi:196011866) in RBL subgroup. No-bilaterian animal sea anemone (*N. vectensis*) only possesses one gene of RB family, and was clustered into RBL subgroup in ML and BI phylogenetic analysis, Bilaterian, *S. purpuratus and B. floridae*, also possess their representative members in RB subgroup and RBL subgroup respectively. This indicated that the first gene duplication for ancient RB gene happened before the separation of placozoans and bilaterians. As the placozoans are thought to be diverged before the separation of cnidarians and bilaterians, the absence of RB1 subgroup orthologue in sea anemone (*N. vectensis*) is possibly due to independent gene loss.

There are two RB family proteins, RBF1-Dm and RBF2-Dm, in *D. melanogaster*, and one RB family protein, Lin35, in *C. elegans*. None of them can be classed into any subgroup due to their high sequence divergence. As RBF1 and RBF2 are tightly clustered in our tree, we tend to think that RBF1 and RBF2 were formed by lineage specific gene duplication. Interestingly, we found that fly RBF1 gene share an interspecies conserved exon with Human RBL1 and RBL2 genes (Figure 3, additional file 3), this data added the possibility that RBF1 belong to RBL subgroup. As the evidence is still limited, we do not class RBF1 into any subgroup in this study.

According to Figure 4, the second gene duplication occurred in RB family, but this only happened in RBL subgroup, not in RB1 subgroup. This gene duplication in RBL subgroup happened before tetrapoda and teleostei divergence, as *H. sapiens and D. rerio *possess representative RBL1 and RBL2 genes respectively. Human RBL1 and RBL2 gene emerged by gene duplications of the common RBL subgroup ancestor, and representative RBL subgroup members from invertebrates *T. adhaerens*, *N. vectensis, S. purpuratus, B. floridae*, and *C. intestinalis*, which we named them as RBL-Ta, RBL-Nv, RBL-Sp, RBL-Ci, and RBL-Bf, should be recognized as the co-orthologues of Human RBL1 and RBL2 genes (Table 2). Gene losses of RB family might also happen in some vertebrates. For example, RBL2 orthologue were not found in *T. nigroviridis*, and RB1 orthologue was not found in *X. tropicalis*.

For DP family (Figure 4, Table 2), all invertebrates only have one orthologue of DP family. Similar to E2F7/8 family, gene duplication for DP family happened before tetrapoda and teleostei divergence. Incidentally, it was found that species-specific gene duplications for DP family happened in *H. sapiens*, *M. musculus*, and *D. rerio*. For example: Human DP3-Hs (gi:189409125) should be thought as the product of species-specific gene duplication of human DP1-Hs (gi:6005900).

### Repressive E2Fs (E2F4, E2F5) evolved slower than active E2Fs (E2F1, E2F2, E2F3)

In ML tree, the branch lengths for E2F1/2/3 subgroup proteins are longer than for E2F4/5 subgroup proteins in E2F1-6 protein family. This indicated E2F1/2/3 subgroup may evolve more rapidly than E2F4/5 subgroup. So we did a detailed investigation of evolutionary rates of two subgroup proteins.

At first, the eukaryotic ancestral E2F sequence was predicted using the full alignment of E2F sequences of eukaryotic organisms and its phylogenetic tree by the software GASP. The ancestral E2F sequence could be found in additional File 4.

**Figure 5:**
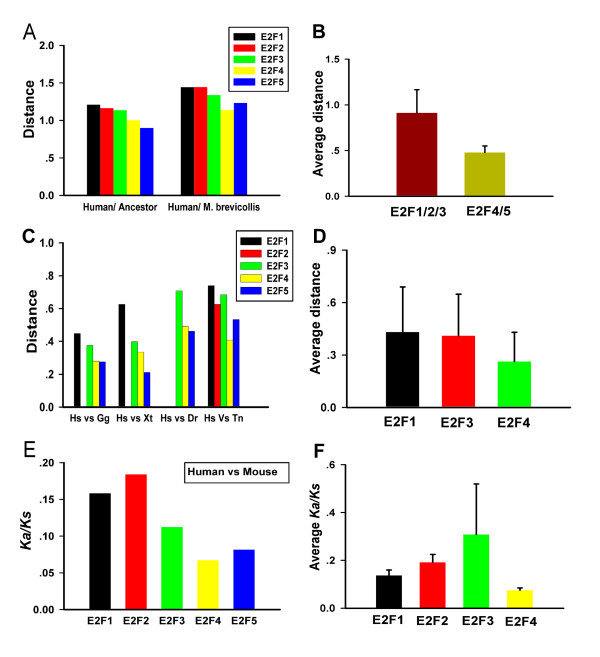
**E2F4/5 subgroup evolved slower than E2F1/2/3 subgroup**. **(A) **Distance between Human E2F proteins and predicted ancestral E2F sequence. The pairwise distances between human E2Fs and predicted ancestral E2F sequence, E2F-Mb(gi|167523471). **(B) **The group average pairwise distance of E2F4/5 subgroup and E2F1/2/3 subgroup in invertebrates. Based on the sequence data from Ta, Nv, Ci. Sp and Bf, the average distance of E2F4/5 subgroup is smaller than E2F1/2/3 subgroup (*P *< 0.001, with Pair Student's T Test), standard deviation values are showed as error bars. **(C) **Distance between human E2F proteins and other vertebrate orthologues. Pair wise distances between human E2F proteins and their orthologues from Gg, Xt. Dr, Tn were computed respectively. **(D) **The group average distances of E2F4, E2F1, and E2F3 in vertebrates. Based on protein sequence data from 8 organisms (Hs, Mm, Cf, Bt, Rn, Gg, Xt, Tn), the group average distance of E2F4 is smaller than E2F1 and E2F3 (E2F1:E2F4, *P *< 0.001; E2F3:E2F4, *P *< 0.001). **(E) ***Ka/Ks *values for E2F genes based on codon sequences from human and mouse. **(F) **The group average *Ka/ks *of E2F1, E2F2, E2F3, and E2F4 in mammals. Based on *Ka/ks *values for protein-coding DNA sequences from four mammals (Hs, Cf, Bt, Mm) in each subgroup, The group average *Ka/ks *value of E2F4 subgroup is smaller than E2F1 subgroup, E2F2 subgroup and E2F3 subgroup (For E2F1:E2F4, *P *< 0.001; E2F2:E2F4, *P *< 0.001; E2F3:E2F4, *P *< 0.05.). Detail information could be found in additional file 5 and additional file 6. Abbreviations: *Hs*, *H. sapiens*; *Mm*, M. musculus; Cf, *C. familiaris; Bt, B. Taurus; Gg*, *G. gallus*; *Xt, X. tropicalis*; *Dr*, *D. rerio*; *Tn, T. nigroviridis; Ci*, *C. intestinalis*; *Sp*, *S. purpuratus*; Bf, *B. floridae, Ta, T. adhaerens*.

It was found that human E2F4 and E2F5 possess smaller distance to the predicted ancestral E2F sequence or the E2F-Mb (gi|167523471) compared with human E2F1, E2F2 and E2F3 (Figure 5A). However this kind of differences is not significant, as the small substitution rate difference could be partly masked by mutational saturation in comparisons between distantly related species. So we further investigated the substitute rate in E2F1/2/3 subgroup and E2F4/5 subgroup in more closely related organisms.

For invertebrates, based on the sequences data from 5 organisms (Ta, Nv, Sp, Ci, and Bf), we found that the group average pairwise distance among the orthologues in E2F4/5 subgroup were smaller than that in E2F1/2/3 subgroup (*P <*0.001, with pair Student's T Test) (Figure 5B). In vertebrates, the distances between human E2F4, E2F5 and their orthologues from other vertebrates (*G. gallus*; *X tropicalis*; *D. rerio*; *T. nigroviridis) *were smaller than the distances between human E2F1, E2F2, E2F3 and their orthologues respectively (Figure. 5C). As the group average pairwise distance comparisons between different genes general require these genes are derived from a same collection of organisms, Gene losses for E2F2 and E2F5 in several vertebrate organisms (Table 1) make E2F2 and E2F5 was not suitable to compute their group average pairwise distances and then make a statistical meaningful comparing in our study. For E2F1, E2F3, and E2F4 gene in vertebrate, the sequences data from 8 vertebrate organisms (Hs, Mm, Cf, Bt, Rn, Gg, Xt, Tn) was used for analysis, we found that the average group pairwise distance of E2F4 gene is smaller than E2F1 and E2F3 (*P *< 0.001, with Pair Student's T Test) (Figure 5D). In general, we thought the slower evolvement of E2F4/5 subgroup compared with E2F1/2/3 subgroup was conserved from invertebrates to vertebrates,

Finally, the selection pressures on human E2F proteins were examined by estimating the average ratio of nonsynonymous to synonymous substitutions (*Ka/Ks*) at each codon position. The codoning sequences alignments for E2F family proteins, and detail the *Ka/ks *data could be found in additional file 5 and additional file 6. We found that E2F1, E2F2 and E2F3 have severely reduced selection in comparison with E2F4 and E2F5 in cordon sequence comparison between human and mouse (Figure 5E). In four mammals (Bt, Hs, Mm, and Cf), we also found that the group average *Ka/Ks *values for E2F4 was smaller than the group average *Ka/Ks *values E2F1, E2F2, or E2F3 with statistical support (*P *< 0.05, with pair Student's T Test) (Figure 5F). As E2F5 was absent in Bt and Rn, E2F5 was not covered in group average *Ka/Ks *value study.

### RBL subgroup proteins evolved slower than RB1 subgroup proteins in vertebrates

**Figure 6:**
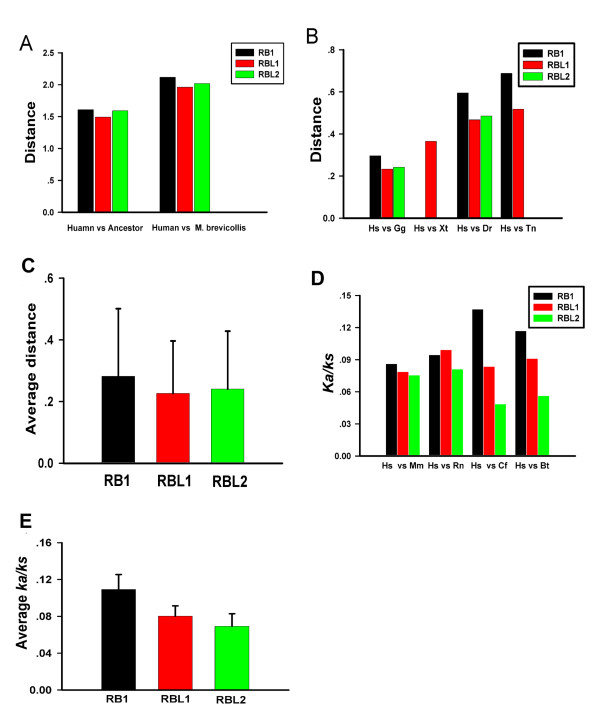
**RBL subgroup evolved slightly slower than RB1 subgroup in vertebrates**. **(A) **Distance between Human RB family proteins and predicted ancestral RB sequence. The pair wise distances between human RB1, RBL1, RBL2 and predicted ancestral RB sequence, RB-Mb (gi:167523296) sequence were computed. **(B) **Distance between human RB family proteins and their vertebrate orthologues. Pair wise distances were calculated between human RB1, RBL1, RBL2 and their orthologues in Gg, Xt, Dr, Tn, respectively. **(C) **The group average pairwise distances RB family in vertebrates. Based on protein sequences from 7 organisms (Hs, Mm, Cf, Bt, Rn, Gg, and Dr), the group average distance of RBL1 subgroup and RBL2 subgroup is smaller than RB1 subgroup (For RB1: RBL1, *P *< 0.001; RB1:RBL2, *P *< 0.001; with pair Student's T test), standard deviation values are showed as error bars. **(D) ***Ka/Ks *values for RB family genes deduced from sequences comparisons between human and other mammals. The ratio of nonsynonymous (Ka) to synonymous substitutions (Ks) of RB1, RBl1 RBL2 genes were computed, based on codoning sequences pairwise comparisons (Hs vs Mm, Hs vs Rn, hs vs Cf, hs vs Bt) respectively. **(E) **Average Ka/ks values for RB family gene in mammals. Based on the *Ka/ks *values for all protein-coding DNA sequences from 5 mammals (Hs, Cf, Bt, Mm, and Rn) in each subgroup, The group average *Ka/ks *value of RBL1 subgroup and RBL2 subgroup is smaller than RB1 subgroup with statistical support (For RBL1:RB1, *P *< 0.01; RBL2:RB1, *P *< 0.01), Detail information could be found in additional file 5 and additional file 6. Abbreviations used: *Hs*, *H. sapiens*; *Mm*, M. musculus; Cf, *C. familiaris; Bt, B. Taurus; Gg*, *G. gallus*; *Xt*, *X tropicalis*; *Dr*, *D. rerio*; *Tn, T. nigroviridis; Ci*, *C. intestinalis*; *Sp*, *S. purpuratus*; Bf, *B. floridae*.

For RB family proteins, eukaryote ancestral RB sequence was predicted using the full alignments of all 39 RB like protein sequences of eukaryotic organisms and its phylogenetic tree by the software GASP. The ancestral E2F sequence could be found in additional File 3. However, differences in the distance between three human RB family proteins and the ancestral RB sequence or RB-Mb (gi:167523296) were small (Figure 6A).

In vertebrates, pairwise distances between human RB1, RBL1, RBL2 and their orthologues in *G. gallus, X. tropicalis, D. rario, T. nigroviridis *were calculated. It was found that RB1 homologue evolved more rapidly compared with RBL1 homologue and RBL2 homologue (Figure 6B). Based on the sequence data from 6 organisms (Hs, Mm, Cf, Bt, Rn, Gg, and Dr), We find that the group average pairwise distance of RB1 gene was bigger than the group average pairwise distances of RBL1 gene or RBL2 gene (*P <*0.001, Pair T Test) (Figure 6C).

To check the selection pressure, we first investigated *Ka/Ks *values by sequences comparisons between human and other mammals (Figure 6D). The codoning sequences alignments for RB family proteins, and detail the *Ka/ks *data could be found in additional file 5 and additional file 6. In 5 mammals (Bt, Hs, Mm, Rn, and Cf), the average *Ka/ks *for RB1, RBL1, and RBl2 genes were also investigated (Figure 6E). In general, RB1 gene has higher *Ka/Ks *ratios in comparison with RBL1 and RBL2 genes in mammals (*P *< 0.01, pair T Test). Thus RB1 gene owns decreased selection pressure compared with RBL1 and RBL2.

## Discussion

### Origination of the RB-E2F pathway and its possible contribution for multicellular organisms emerging

In our analysis, it was found that E2F1-6 family, RB family and DP family proteins are present in protist *D. discoideum*, green agar *O. tauri*, choanoflagellate *M. brevicollis*. This suggested that E2F and RB proteins were present in early ancestor of eukaryotes, and RB-E2F pathway originated in ancestor of eukaryotes before animal, plant and protist separation. One interesting finding in our study was that all multicellular organisms we checked owned orthologues from E2F1-6 family, RB family, and DP family, which indicated that the RB-E2F pathway is strongly conserved in multicellular organisms.

The emergence of multicellular organisms from single-celled ancestors marks one of the most pivotal events in life's history, which occurred several times, independently in different branches of the eukaryotic tree [30,31]. It was usually thought genes involved in cell-cell communication, cell adhesion and cell differentiation probably arose before, or concomitant with, the origins of multicellularity [30,31]. Given that RB-E2F pathway was found in all multicellular organisms we checked, we speculated this RB-E2F pathway might contribute to multicellular emergence. At least the core function of RB-E2F pathway (discussed in later) is consistent with this speculation. It was known that some cells may continue to proliferate and some cells may differentiate and give out its previous proliferate ability during the transition from unicellular organism to multicellular organism. The cycle controlling and/or differentiation function of RB-E2F clearly could contribute to this progress. In fact, it was found that the expression of retinoblastoma orthologue was increased about 200 times when *D. discoideum *transited from unicellular to multicellular form [32]. By the way, beside E2F and RB proteins, some cycle regulatory components such as the KIP/WAF-type CDK inhibitor, Cyclin D, BRCA1 and BRCA2 are only found in plants and animals to the exclusion of the yeast [33].

### The ancient function of RB-E2F pathway: insights from its evolutionary history

**Figure 7:**
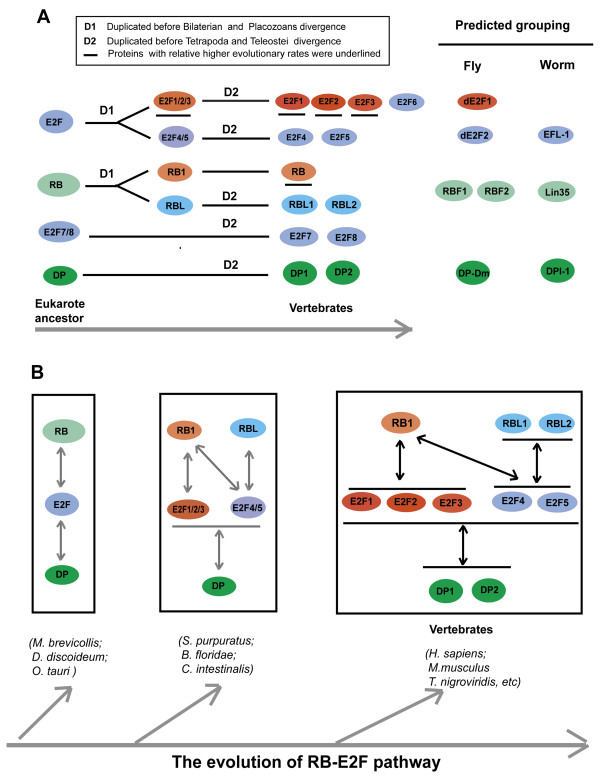
**Overview of the evolutionary history of RB-E2F pathway in metazoan**. **(A) **Summaries of the gene duplication events for E2F, RB and DP proteins in metazoa. The proteins of E2F and RB family from fly and worm were also tried to be classed into suitable subgroup based one sequence and functional data. **(B) **A simple summary of the evolutionary history of RB-E2F pathway. The protein interaction information of RB-E2F pathway in vertebrates was labeled with black arrows, mainly based on the recent literature [2]. The protein interaction information in invertebrates and some unicellular organisms were predicted by interaction data of their vertebrate orthologues, and were labeled with grey arrows.

In this study, we mapped the evolutionary history of E2F and RB proteins. We summarized the evolutionary history of E2F1-6, RB, DP, and E2F7/8 family by listing their gene duplication events (Figure 7A). Ancestral E2F, RB genes duplicated before placozoans and bilaterians diverged, thus E2F family was divided into E2F4/5 subgroup and E2F1/2/3 subgroup, RB family was divided into RB1 subgroup and RBL subgroup (Figure 7A, Table 2). In vertebrates, the genes number in E2F1-6, RB, DP and E2F7/8 families increased by genes duplication events, these duplications occurred no later than tetrapoda and teleostei divergence (Figure 7A, Table 1, and Table 2). In general, we thought that the complexity increase of RB-E2F pathway is compatible with the increasing functional complexity in evolutionary history.

As mentioned in the results section, the fly dE2F1 was classed into E2F1/2/3 subgroup, and fly dE2F2 and worm EFL-1 was classed into E2F4/5 subgroup. This classification was consistent with results of previous functional studies [2]. As similar to repressive E2Fs (E2F4/5 subgroup), fly dE2F2 can form stable repressor complexes in conjunction with RBF1 and RBF2 [34], worm EFL-1 was also reported to form a stable repressor complex which represses the expression of many genes together with Lin35 [35]. And similar to active EFs, dE2F1 reverses the effects of dE2F2 repressor complexes [36]. As to fly RBF1, RBF2, and worm LIN35, as all of them have similar evolutionary distance to RB1 subgroup and RBL subgroup proteins, we do not classify them into any subgroup (Figure.7A).

One intriguing finding in our study was that E2F4/5 subgroup protein share more sequence similarity with the predicted E2F ancestral sequence than E2F1/2/3 subgroup proteins; E2F4/5 subgroup proteins evolved slower and own increased negative selection pressure than E2F1/2/3 subgroup. For RB family proteins, RBL subgroup proteins possess increased negative selection pressure compared with RB1 subgroup in mammals.

Protein evolutionary rates usually linked with protein function [37,38]. The rate of evolution of a protein-coding gene depends primarily on the structural-functional constraints that are intrinsic to the encoded protein [37,38]. As to duplication genes, in neofunctionalization model, it was thought that original copy gene maintain its ancestral function and keep the previous evolutionary rate, the new copy if not pseudogenized or lost, may acquire a new gene function. During this process of acquiring a new gene function, the new copy gene evolved faster than the original copy gene [39,40]. Thus, based on significantly different evolutionary rates within E2F family, we speculated that E2F4/5 subgroup proteins might mainly maintain the ancestral E2F function. And for RB family, RBL1 and RBL2 gene was under more strictly purification selection in mammals, we also speculated the ancestral RB family function might also mainly be represented by RBL subgroup proteins.

For RB-E2F pathway, it is general known that E2F1, E2F2 and E2F3 are active E2Fs and E2F4 and E2F5 are repressive E2Fs. RB family proteins are thought to function in cell cycle and cell differentiation. Though RB family might share similar functions, different RB family members might also function differently [2], only RB1 protein, but not RBL1 and RBL2, could interact to active E2Fs (E2F-1, E2F-2 and E2F-3); RBL1 and RBL2 can only interact to repressive E2Fs (E2F4 and E2F5) which is much more abundant and functional in G0-arrested cells than other E2F factors [2,41]. Interestingly, In *C. elegans*, there is only one RB family protein Lin-35, Lin 35 is somewhat more closely related to human RBL2 and RBL1 (Lin-35 shares 20, 19, and 15% overall amino-acid identity with RBL2, RBL1, and RB1, respectively) [42], previous studies did not reveal a cell-cycle role for Lin35, Lin35 is not rate limiting for S-phase entry [43]. In dictyostelium, the RB family orthologue also reported not function in cell cycle [32]. In Arabidopsis, local reduction of expression of the retinoblastoma-related (RBR) gene (homologue of human RB family) in roots increases the amount of stem cells without affecting cell cycle [44]. We speculated the ancient function of RB family was mainly related to cell differentiation, but not tightly related to cell cycle.

Functional studies in RB-E2F pathway also indicated that the function conducted by E2F4/5 subgroup and RBL subgroup proteins is extremely conserved. A key finding about RB-E2F pathway came with the biochemical purification of native E2F-RBF complexes from *D. melanogaster *embryo extracts (dREAM [45] and Myb-MuvB (MMB) [46]), and the repressive dE2F2, RBF1 and RBF2 are members of these complexes. Similar complexes were subsequently purified from C. elegans (DRM [47]), which contain Lin35 and EFL-1. For mammals, similar complex was also found (DREAM [48,49] and lINC [50]). These complexes contain the repressive E2F protein E2F4 or E2F5, and RBL subgroup protein RBlL1 or RBL2. Human DREAM was found to bind to more than 800 human promoters in G0 and was required for repression of E2F target genes [49]. As to the E2F1/2/3 subgroup and RB1 subgroup members, no similar conserved protein complex was found till now.

Taken all together, it was temptation to think that the function conducted by E2F4/5 subgroup and RBL subgroup might be more conserved than those function conducted by E2F1/2/3 subgroup and RB1 subgroup.

## Conclusions

The most important cell fate decisions, such as whether to divide, differentiate or die, usually are very strictly regulated. The RB-E2F pathway plays critical roles on these fundamental cellular processes. We carried out comprehensive evolutionary analyses for RB and E2F family, our data demonstrated that E2F4 and E2F5 share more sequence similarity with the predicted E2F ancestral sequence than E2F1, E2F2 and E2F3; E2F4 and E2F5 also possess significantly lower evolutionary rates and higher negative selection pressures than E2F1, E2F2 and E2F3. For RB family, the RBL subgroup proteins evolved slower and also possess higher purification selection pressures compared with RB1 subgroup proteins. We speculated that that ancient function of RB-E2F pathway might mainly link with repressive E2F and RBL subgroup proteins. And the active E2F and RB1 subgroup proteins might contribute more to functional diversification in RB-E2F pathway. Our results will enhance the current understanding of RB-E2F pathway and will also be useful to further functional studies in human and other model organisms.

## Abbreviations

HS: Homo sapiens; CF: Canis familiaris; BT: BOS TAURUS; MM: Mus musculus; RN: Rattus norvegicus; GG: Gallus gallus; XT: Xenopus tropicalis; DR: Danio rerio; TN: Tetraodon nigroviridis; SP: Strongylocentrotus purpuratus; Bf: Branchiostoma floridae; CI: Ciona intestinalis; CE: Caenorhabditis elegans; DM: Drosophila melanogaster; NV: Nematostella vectensis; Ta: Trichoplax adhaerens; Mb: Monosiga brevicollis: At: Arabidopsis thaliana; Os: Oryza sativa; Dd: Dictyostelium discoideum AX4; Ot: Ostreococcus tauri.

## Competing interests

The authors declare that they have no competing interests.

## Authors' contributions

LC and LY conceived this study; LC, BP, MZ, and KS collected the data; LC, BP and LY made the analysis; LC, BP and MY wrote the manuscript.

## Reviewer's report 1

### Pierre Pontarotti (UMR 6632 Université de Aix Marseille/CNRS. Equipe Evolution biologique et Modélisation France)

This article describes the phylogenetic analysis of three gene families involved in the same pathway. The robustness of the phylogenetic analysis is proper, however I disagree with the title of the article that do not reflect exactly the work carried out. Indeed the authors performed only a classification. However I think that this classification of protein families is helpful for the scientific community and thus I recommend the paper to be published.

***Author's response: *** *As the article also discussed much about the ancient function of RB-E2F pathway, we still kept the title "The ancient function of RB-E2F Pathway: insights from its evolutionary history"*

#### General remark

The paper is difficult to understand and should be clarified, in particular the fact that some duplications are lineage-specific and other are older. The authors should use terms such as in paralog or co-orthologs to make the information clearer. I think that the paper should be reworked and the concept and result clarified. I wonder also if the authors should propose a new nomenclature, take as example this article: "Nme protein family evolutionary history, a vertebrate perspective by Desvignes et al 2009". This will clarify the issue.

***Author's response: ****We agree that it is a little difficult to understand for some evolutionary relationship in E2F1-6 family and RB family, as there were ancient gene duplications and lineage-specific gene duplications in E2F1-6 family and RB family. We use the term co-orthologues to describe some evolutionary relationships in the revision, for example, E2F4/5 subgroup members from invertebrates T adhaerens, E2F4/5-Ta, should be recognized as the co-orthologues of vertebrate E2F4 and E2F5 gene. We added two tables, similar to the article: "Nme protein family evolutionary history, a vertebrate perspective by Desvignes et al 2009" *[51] .

In several instances, the phylogeny based only on bases substitution could not be informative, therefore other characters should be taken into an account, for example exon intron organization (see for example page 10, third paragraph).

***Author's response: ****E2F and RB family protein sequences from fly and worm usually diverged highly and could not be clustered into subgroup in phylogenetic analysis based on protein sequences. As the reviewer's suggestion, The Exon intron structure of E2F1-6 family, RB family genes from fly, worm, and human were studied in this revision (figure *3*, additional file *3*). We found that E2F2-Dm, E2FL1-Ce, E2F4-hs, and E2F5-hs gene share some Exon intron structure similarity, which support our speculation that E2F2-Dm and E2FL1-ce gene should be classified into E2F4/5 subgroup. However, For E2F1-Dm gene, no Exon intron structure similarity with human E2F1/2/3 subgroup or E2F4/5 subgroup genes were found. For RB family, we found that fly RBF1 gene share an interspecies conserved exon with Human RBL1 and RBL2 genes (Figure *3*), these data added the possibility that RBF1 belong to RBL subgroup. As the evidence is still limited, we do not class RBF1 into any subgroup in this study*.

My major problem concerned the figure 6 in which the author indicate that for example that B Floridae is the vertebrate ancestor. The entire lineage evolved, some show, of course, more apomorphism or plesiomorphism than other, However none of the present day specie is the ancestor of another present day specie.

***Author's response: ****We corrected these problems in Figure *6* (Now is Figure *7*)*.

The sentence page 11: "during the transition from invertebrate to vertebrate" is also misleading. The authors should write a second gene duplication occurred in the RB family in the vertebrate lineage after their separation with the amphioxus ancestor.

***Author's response: *** *We corrected these problems. We changed it to "The second gene duplication occurred in RB family, but this only happened in RBL subgroup, not in RB1 subgroup. This gene duplication in RBL subgroup happened before tetrapoda and teleostei divergence, as H. sapiens and D. rerio possess representative RBL1 and RBL2 genes respectively"*

#### Other comments

Lot of misspellings are present in this report. This start with the first line of the paper where the word background is misspelled. In general vocabulary and grammar should be checked by a native English speaker. Please ask to a native Anglophone to review the article All the phylogenetic trees presented in the figures show problems with the species branching For example; figure 1 E2F1 of *Xenopus *and *Tetraodon *form a monophylogenetic group; instead *Xenopus *should branch with Gallus and mammalian. Moreover, the authors have to check all the phylogenetic incongruence and comment them.

***Author's response: ****We corrected several misspellings which have been mentioned by three reviewers and also asked one native English speaker to review this article. We corrected the mistake about "Xenopus and Tetraodon form a monophylogenetic group"*.

*The phylogenetic incongruences sometimes are due to protein sequence highly diverged in some organisms (such as in S. purpuratus; C. intestinalis; C. elegans; and D. melanogaster). We added some comments for these phylogenetic incongruences in the revision. For example, According to Dr. Arcady Mushegian's suggestion, we added the protein sequences from the placozoan T. adhaerens in our analysis. T. adhaerens is a basal eumetazoan lineage that diverged before the separation of cnidarians and bilaterians. As T adhaerens owns representative members for RB1 subgroup and RBL subgroup. And N. vectensis possess representative member in RBL1 subgroup, but no member in RB1 subgroup. Based on the data in T. adhaerens, we speculated that this absence of orthologue of RB1 subgroup in N. vectensis is due to independent gene loss*.

## Reviewer's report 2

### Dr. Arcady Mushegian (Stowers Institute, Kansas City, United States)

p.3 "Proteins that are related to the retinoblastoma tumor suppressor RB and the E2F transcription factor... have been missing from yeasts and other fungi" -- this is incorrect. Orthologs of retinoblastoma may be missing from the fungi, but "proteins related" to retinoblastoma are certainly there, i.e., any protein that contains a BRCT domain.

***Author's response:** We corrected this mistake, and we mentioned that some fungi possess orthologues of E2F1-6 family and DP family, but no orthologue of RB family in the introduction*.

p.5 E-value less than e-5 was used to collect the homologs. At a more common default value of e-3, have there been more matches, especially in the species that the authors say lacked any homologs (e.g., fungi)?

***Author's response:** As reviewer's suggestion, using human E2F4, E2F7, DP1, RB1 proteins, we performed Blast searchings, with *E-v*alue less than e-3 as cut-off, in NCBI data base. We got the same results in the organisms we checked. For Fungi, I will answer it below*.

The authors do not mention Trichoplax adhaerens at all. This is a shortcoming: even if Placozoa are not the most primitive metazoan animal, it is still primitive clade, perhaps close to the split of cnidaria and bilateria. All hypotheses about what happened before or after the latter split may gain additional evidence if this species is included (genes are not very well annotated in Trichoplax, so some prediction by homology may be needed).

***Author's response:** Thanks for the good suggestion. We found Trichoplax adhaerens own two orthologues of E2F1-6 family, and two orthologues of RB family, and one orthologue of DP family. T. adhaerens are thought to be diverged before the separation of cnidarians and bilaterians *[12].* E2F and RB family proteins from T. adhaerens are now covered in analysis in the revision*.

I would be happier if multiple fungi with complex morphology were included in the analysis, not only yeasts with what we know to be abbreviated genomes.

***Author's response:** We did the Blast searching, with human E2F4, E2F7, RB1, and DP as queries, in fungi (taxid:4751) in NCBI database. In addition, domain associations of all possible proteins were checked in SMART and pfam database. In total, we found six proteins which belong to E2F1-6 family or DP family in fungi. Below is the detail: Nosema ceranae BRL01 owns one E2F1-6 family protein (gi:239605701), one DP family protein (gi:239605391); Enterocytozoon bieneusi H348 owns one E2F1-6 family protein gi|169806750), one DP family protein (gi|169806250); Encephalitozoon cuniculi GB-M1 owns one E2F1-6 family protein gi|19074054), and one DP family protein (gi|19074276), However, No RB family protein was found in fungi. N. ceranae, E. bieneusi, and E. cuniculi belong to the phylum Microsporidia, The microsporidia constitute a phylum of spore-forming unicellular parasites. They were once thought to be protists but are now known to be fungi *[10] .

*As our main interest is on RB-E2F protein evolution in metazoa, and no RB family protein was found in fungi, we did not cover these fungi E2F and DP protein in detail analysis this time. However, we mentioned the E2F1-6 family, and DP family proteins from E. cuniculi in the introduction in this revision*.

p.10, par.3 replace "homologue" with "orthologoue" twice? All E2F family members are homologs by definition. Similarly elsewhere in the manuscript, for example the fourth paragraph on the same page.

**Author's response:** we correct them and similar problems in elsewhere in the manuscript

p.16-17: "based on the significantly evolutionary rate differences [should be: significantly different evolutionary rates] within E2F family and RB family, we speculated that E2F4/5 subgroup proteins might mainly maintain the ancestral E2F function. And for RB family, the ancestral RB family 17 function might also mainly represent by RBL subgroup proteins." -- What those ancestral functions might be, as opposed to derived functions of the other family members? This is answered very briefly in penultimate paragraph on p. 17, but needs to be elaborated.

***Author's response:** we change to "significantly different evolutionary rates" as reviewer suggested, We add one paragraph which was tried to describe about function difference between RB1 subgroup and RBL subgroup, and speculated that ancient function of RB family might be linked with cell differentiation but not with cell cycle controlling*.

## Reviewer's report 3

### Dr. Zhenguo Lin, Department of Ecology and Evolution, The University of Chicago (nominated by Dr. Neil Smalheiser)

The RB-E2F pathway is involved in several fundamental processes of cellular activities, including regulating the initiation of DNA replication. Disruption of the RB-E2F pathway has been shown to be associated with all human tumors. Therefore, it is of great interest to study the origin and evolution of this pathway. The authors reconstructed the evolutionary histories for the three gene families (E2F, RB and DP) in this pathway. They also found that different members in each of the RB and E2F family might have experienced different selection constraints. This study provides some new understandings about this important pathway.

#### Major comments

One of the major conclusions of this study is that "E2F4/5 genes have "significantly lower evolutionary rates" than E2F1/2/3, and RBL1/2 "also evolved slightly slower compared with RB1 in vertebrates". These conclusions are based on comparing values of protein divergence data or *Ka/Ks*. I do not know what criteria were used to determine if the evolutionary rate is "significantly lower" or "slightly slower". For example, the authors also used "this kind of differences is not big," "no big difference was found in the distance" in Page 13. Without any appropriate statistical inference, such claims seem to be invalid. In addition, the authors only compared human with other species to obtain protein distances and *Ka/Ks*. I would suggest authors to calculate all pairwise distance values for all protein sequences in each subgroup, and perform a statistical evaluation to determine if two subgroups evolve under significant different rates.

***Author's response:** As the reviewer's suggestion, we did group average pairwise distance value comparisons, and group average Ka/ks value comparisons, and perform statistical evaluations*.

*For group average pairwise distance values comparisons, all pairwise distance values for all protein sequences in each subgroup was first computed. Then average value of group pairwise distances was calculated. For group average Ka/ks values comparing, all Ka/ks values for all protein-coding DNA sequences in each subgroup was first computed. Pair Student's T Test was used for statistical analysis. Considering the effect of gene losses, we thought that the group average pairwise distances comparing between different genes general require these genes are derived from a same collection of organisms. For E2F1-6 family, we did group average pairwise distances comp comparison invertebrates, (Figure *5B*), and in vertebrate (Figure *5D*), For RB family, we did group average pairwise distances comp comparison in vertebrate (Figure *6C*), For group average Ka/ks values comparison, we did them in E2F1-6 family (Figure *5F*), and in RB family (Figure *6E*). In the new additional file *6*, all the data about group average distances and group average ka/ks values could be found*.

In general, the E2F4/5 subgroup evolved significantly slower than E2F1/2/3 subgroup(P < 0.001, with pair T Test), For RB family, RBL subgroup proteins evolved slower than RB1 subgroup proteins in vertebrates (P < 0.01, with Pair T Test)

The basal groups on the tree of E2F1-6 subfamily in Fig 2A and Fig S3A are poorly resolved, therefore the evolutionary relationships between the *Monosiga brevicollis E2F *genes with other E2F member remains unclear. Under this condition, *MbE2F *gene could be a member of either E2F1/2/3 group or E2F4/5 groups. So, I am not sure if it is appropriate to use MbE2F as the outgroup to infer that E2F1/2/3 group or E2F4/5 groups.

***Author's response:** One reason for our using Monosiga brevicollis E2F as the outgroup of Metazan E2F1-6 family was that M. brevicollis is the close relative of metazoa, And we had tried many times, but we still failed to classified M. brevicollis E2F genes into either E2F1/2/3 group or E2F4/5 groups. When the E2F protiens from D. discoideum or plant were used as outgroups, the topology of E2F1/2/3 group or E2F4/5 groups in metazoa do not change comparing with E2F from M. brevicollis was used as the outgroup*.

E2F1/2/3-Nv (gi:156371340) sequence is much shorter than other E2F proteins, it contains only the N-terminal domains and it is about ½ length of its orthologous genes. The prediction of its CDS is probably incorrect. I would suggest the authors to do TBLASTN against its genomic sequence to obtain its complete sequence. Otherwise, it is not a good idea to include partial sequence in the alignment for tree building. In addition, E2F3-Rn has the same problem. The authors need to carefully inspect the alignments to detect obscure sequences. Better trees are needed for better supports of their conclusions.

***Author's response:** We tried to do TBLASTN against its genomic sequence using the E2F1/2/3-Ta (gi:196010483) which is most similar to E2F1/2/3-Nv (gi:156371340) in our analysis, But still good result. As E2F1/2/3(gi:156371340) sequence contained the E2F-TDP domain, and still be good cluster into E2F1/2/3 subgroup, so We kept it in analysis, For E2F3-Rn, We rechecked it sequences in genbank and in ensembl data base, In ensembl database, Rat E2F3 gene, LOC691420 (ENSRNOT00000050261), also only coded 245 AA. When we did two sequence blast for mRNA sequences of Human E2F3 (GI:16848011) and Rat E2F3(GI:212549626), We found that a reading frame shift for Rat E2F3 which induced rat E2F3 protein sequence had been unexpected stopped in comparison with human E2F3 mRNA.. In detail: the sequence at the position 1231-1254 of human E2F3 mRNA (GI:168480112) is "CAA-GAT-ATT-CGA-AAA-ATT-AGT-GGC", the sequence at the position 1045-1068 of Rat E2F3 mRNA (GI:212549626) is "TCA-AGA-TAT-TCG-AAA-AAT-TAG-TGG"*.

#### Minor comments

Page 5. "in additional" should be "in addition", or "additionally".

***Author's response:** we corrected it*.

Page 8. First line" We found that all three unicellular organisms, *D. discoideum AX4, O. tauri, and M.brevicollis *contain". In fact, S. pombe and S. cerevisiae are also unicellular organisms.

***Author's response:** The fact is that some unicellular organisms owns E2F, RB family protein, and some unicellular organisms (S. pombe and S. cerevisiae) not. We removed the word "all"*.

Page 8. Last line of first paragraph. "E2F7/8 family was not as indispensable as E2F1-6, RB, and DP family" if it is not your conclusion, reference is needed.

***Author's response:** it is our conclusion*.

Page 9, Second paragraph "did not function in classical RB-E2F pathway" any reference?

***Author's response:** we added the reference*.

Page 10, third paragraph "And this classification was also consistent with results of previous functional studies." any reference?

***Author's response:** we added the reference*.

Page 10, third paragraph "its sequence highly diverged" should be "its sequence IS highly diverged".

***Author's response:** we corrected it*.

Page 11. "RBF1 and RBF2 are formed by lineage specific gene duplication, and this gene duplication may not happen very early" Only one arthropod species (fruit fly) was used in this study, I agree that RBF1 and RBF2 are formed by lineage specific gene duplication, but I am not sure about "not happen very early". The two branches seem to be long; the duplication could happen as early as before divergence of arthropods. Therefore, without including more arthropod species, this conclusion seems to be under question.

**Author's response:** we corrected it, and removed " and this gene duplication may not happen very early"

Page 14 "partly" should be "partially"

***Author's response:** we corrected it*.

Figure legend, Fig 4. "Codon sequence", I think it is better to use "Coding sequences"

***Author's response:** we corrected it*.

## Supplementary Material

Additional file 1 **E2F1-6, E2F7-8, RB, and DP family proteins sequences**. The protein name and Genbank accession number for all of E2F1-6, E2F7-8, RB, and DP family proteins we identified in this study. The sequence of all E2F1-6, E2F7-8, RB, and DP family proteins were also listed. Click here for file

Additional file 2 **Phylogenetic analyses of E2F1-6, E2F7/8, RB, and DP family in eukaryote**. Maximum likelihood (ML) trees were constructed by using PHYML V.2.4 for E2F1-6, E2F7/8, RB, and DP family in eukaryota, with 200 bootstrap resamplings and JTT setting, Click here for file

Additional file 3 **Exon intron structures**. The exon and intron structures of E2F and RB family genes from human, fly, and worm. Click here for file

Additional file 4 **Predicted ancestral sequences of E2F and RB**. Detail ancestral sequences of E2F and RB predicted by Gapped Ancestral Sequence Prediction program (GASP) program Click here for file

Additional file 5 **The codoning sequences alignments for E2F and RB family proteins**. The codoning sequences alignments for E2F and RB family proteins used in computing *Ka/Ks *in Figure 5 and Figure 6. Click here for file

Additional file 6 **The protein pairwise distance and the *Ka/ks *data**. The detail data of the protein pairwise distance and the *Ka/ks *Values used for group comparisons in this study. Click here for file
