# Supplementary material for: The ancient function of RB-E2F Pathway: insights from its evolutionary history
Source: Biol Direct. 2010 Sep 20;5:55. doi: 10.1186/1745-6150-5-55 (PMC3224931; doi:10.1186/1745-6150-5-55)
Supplement: Additional file 1 — E2F1-6, E2F7-8, RB, and DP family proteins sequences. The protein name and Genbank accession number for all of E2F1-6, E2F7-8, RB, and DP family proteins we identified in this study. The sequence of all E2F1-6, E2F7-8, RB, and DP family proteins were also listed. [file 1745-6150-5-55-S1.DOC]

**Tables for E2F1-6, E2F7-8, RB, and DP family proteins in pages 1-8**

**Sequences for E2F1-6, E2F7/8, RB, and DP family in pages 9-52**

**Tables for E2F1-6, E2F7-8, RB, and DP family proteins**

**Table-S1. E2F1-6 family proteins**

| Organism | Protein name used in This Study | Genbank accession number | length |
| --- | --- | --- | --- |
| *Homo sapiens* | e2f1-Hs | gi|12669911|ref|NP_005216.1| | 437 |
|  | e2f2-hs | gi\|4758226\|ref\|NP_004082.1\| | 437 |
|  | e2f3-hs | gi\|4503433\|ref\|NP_001940.1\| | 465 |
|  | e2f4-hs | gi\|12669915\|ref\|NP_001941.2\| | 413 |
|  | e2f5-hs | gi\|134142811\|ref\|NP_001942.2\| | 346 |
|  | e2f6-hs | gi\|109637795\|ref\|NP_937987.2\| | 281 |
| *Canis familiaris* | e2f1-Cf | gi|73992245|ref|NP_542963.2| | 571 |
|  | e2f2-Cf | ENSCAFT00000021059 | 443 |
|  | e2f3-Cf | gi\|74004128\|ref\|NP_545361.2\| | 509 |
|  | e2f4-Cf | gi\|73957515\|ref\|NP_853975.1\| | 409 |
|  | e2f5-Cf | gi\|73999542\|ref\|NP_853833.1\| | 304 |
|  | e2f6-Cf | gi\|73980432\|ref\|NP_852463.1\| | 282 |
| *Bos taurus* | e2f1-bt | gi|194672360|ref|NP_615437.4| | 437 |
|  | e2f2-bt | gi\|76611569\|ref\|NP_874289.1\| | 367 |
|  | e2f3-bt | gi\|76663083\|ref\|NP_614932.2\| | 463 |
|  | e2f4-bt | gi\|115497534\|ref\|NP_001069341.1\| | 404 |
|  | e2f6-bt | gi\|116003911\|ref\|NP_001070316.1\| | 285 |
| *Mus musculus* | e2f1-mm | gi|6681243|ref|NP_031917.1| | 430 |
|  | e2f2-mm | gi\|29244208\|ref\|NP_808401.1\| | 443 |
|  | e2f3-mm | gi\|83523736\|ref\|NP_034223.1\| | 457 |
|  | e2f4-mm | gi\|22507329\|ref\|NP_683754.1\| | 410 |
|  | e2f5-mm | gi\|31982405\|ref\|NP_031918.2\| | 335 |
|  | e2f6-mm | gi\|237681138\|ref\|NP_150373.2\| | 272 |
| *Rattus norvegicus* | e2f1-rn | gi|189217865|ref|NP_001094248.1| | 432 |
|  | e2f3-rn | gi\|212549627\|ref\|NP_001131098.1\| | 245 |
|  | e2f4-rn | ENSRNOT00000021145 | 412 |
|  | e2f6-rn | gi\|109479090\|ref\|NP_001069501.1\| | 272 |
| *Gallus gallus* | e2f1-gg | gi|45382583|ref|NP_990550.1| | 403 |
|  | e2f3-gg | gi\|118086362\|ref\|NP_418915.2\| | 455 |
|  | e2f4-gg | gi\|118096144\|ref\|NP_001231948.1\| | 414 |
|  | e2f5-gg | gi\|71896455\|ref\|NP_001026113.1\| | 364 |
|  | e2f6-gg | ENSGALT00000031596 | 216 |
| *Xenopus tropicalis* | e2f1-xt | ENSXETT00000046332 | 381 |
|  | e2f3-xt | gi\|58331835\|ref\|NP_001011109.1\| | 427 |
|  | e2f4-xt | gi\|167560905\|ref\|NP_001107966.1\| | 374 |
|  | e2f5-xt | gi\|188528909\|ref\|NP_001120880.1\| | 371 |
| *Danio rerio* | e2f3-dr | gi|220673319|emb|cax13650.1| | 429 |
|  | e2f4-dr | gi\|47087407\|ref\|NP_998597.1\| | 393 |
|  | e2f5-dr | gi\|68533607\|gb\|aah98598.1\| | 363 |
|  | e2f6-dr | gi\|71892405\|ref\|NP_001025315.1\| | 405 |
| *Tetraodon nigroviridis* | e2f1-tn | ENSTNIT00000007876 | 436 |
|  | e2f2-tn | ENSTNIT00000020634 | 445 |
|  | e2f3-tn | ENSTNIT00000012687 | 439 |
|  | e2f4-1-tn | ENSTNIT00000002389 | 403 |
|  | e2f4-2-tn | ENSTNIT00000002758 | 400 |
|  | e2f5-tn | ENSTNIT00000004791 | 301 |
|  | e2f6-1-tn | ENSTNIT00000008935 | 283 |
|  | e2f6-2-tn | ENSTNIT00000013829 | 391 |
| *Strongylocentrotus purpuratus* | e2f4/5-sp | gi|115696783|ref|NP_799123.2| | 356 |
| *Ciona intestinalis* | e2f1/2/3-ci | gi|118343729|ref|NP_001071687.1| | 441 |
|  | e2f4/5-ci | gi\|118343737\|ref\|NP_001071688.1\| | 269 |
| *Branchiostoma floridae* | e2f1/2/3-bf | gi|260790430|ref|NP_002590245.1| | 280 |
|  | e2f4/5-bf | gi\|260798626\|ref\|NP_002594301.1\| | 327 |
| *Drosophila melanogaster* | de2f1-dm | gi|24648770|ref|NP_524437.2| | 805 |
|  | de2f2-dm | gi\|17137542\|ref\|NP_477355.1\| | 370 |
| *Caenorhabditis elegans* | efl-1-ce | gi|17559226|ref|NP_507289.1| | 342 |
|  | efl-2-ce | gi\|71998283\|ref\|NP_496825.3\| | 412 |
| *Nematostella vectensis* | e2f-4/5-nv | gi|156368461|ref|NP_001627712.1| | 335 |
|  | e2f1/2/3-nv | gi\|156371340\|ref\|NP_001628722.1\| | 263 |
| *Trichoplax adhaerens* | E2F4/5-Ta | gi|196012606|ref|XP_002116165.1| | 241 |
|  | E2F1/2/3-Ta | gi\|196010483\|ref\|XP_002115106.1\| | 502 |
| *Monosiga brevicollis mx1* | e2f-mb | gi|167523471|ref|NP_001746072.1| | 413 |
| *Dictyostelium discoideum ax4* | e2f-dd | gi|66810101|ref|NP_638774.1| | 863 |
| *Oryza sativa* (japonica cultivar-group) | os02g0537500-Os | gi|115446539|ref|NP_001047049.1| | 475 |
|  | os12g0158800-Os | gi\|115487438\|ref\|NP_001066206.1\| | 446 |
|  | os04g0416100-Os | gi\|115458326\|ref\|NP_001052763.1\| | 319 |
| *Arabidopsis thaliana* | ate2f1-at | gi|18420430|ref|NP_568413.1| | 469 |
|  | ate2f3-at | gi\|42571075\|ref\|NP_973611.1\| | 485 |
|  | ate2f2-at | gi\|15220994\|ref\|NP_175222.1\| | 396 |
| *Ostreococcus tauri* | E2F-Ot | gi|55978010|gb|AAV68605.1| | 312 |

**Table-S2.**  E2F7-8 family proteins

| Organism | Protein name used in this study | Genbank Accession number | Length |
| --- | --- | --- | --- |
| *Homo sapiens* | e2f7-hs | gi|145580626|ref|NP_976328.2| | 911 |
|  | e2f8-hs | gi\|38505226\|ref\|NP_078956.2\| | 867 |
| *Canis familiaris* | e2f7-cf | gi|73978133|ref|NP_539692.2| | 955 |
|  | e2f8-cf | gi\|73988917\|ref\|NP_534087.2\| | 864 |
| *Bos taurus* | e2f7-bt | gi|194666654|ref|NP_604488.4| | 995 |
|  | e2f8-bt | gi\|194679774\|ref\|NP_001254261.2\| | 866 |
| *Mus musculus* | e2f7-mm | gi|40254337|ref|NP_848724.2| | 904 |
|  | e2f8-mm | gi\|67972650\|ref\|NP_001013386.2\| | 860 |
| *Rattus norvegicus* | e2f7-rn | NP_001101562.1 | 902 |
|  | e2f8-rn | gi\|109462000\|ref\|NP_001080259.1\| | 877 |
| *Gallus gallus* | e2f7-gg | gi|118082443|ref|NP_416110.2| | 975 |
|  | e2f8-gg | gi\|118091079\|ref\|NP_420910.2\| | 1081 |
| *Xenopus tropicalis* | e2f7-xt | ENSXETT00000031323 | 862 |
|  | e2f8-xt | ENSXETG00000004436 | 694 |
| *Danio rerio* | e2f7-dr | gi|169234759|ref|NP_001038612.2| | 723 |
|  | e2f8-dr | gi\|189521060\|ref\|NP_694311.3\| | 860 |
| *Tetraodon nigroviridis* | e2f7-tn | ENSTNIT00000007956 | 613 |
|  | e2f8-tn | ENSTNIT00000000955 | 773 |
| *Ciona intestinalis* | e2f7/8-ci | gi|198432739|ref|NP_002131779.1| | 978 |
| *Monosiga brevicollis mx1* | e2f7/8-mb | gi|167517423|ref|NP_001743052.1| | 550 |
| *Nematostella vectensis* | e2f7/8-nv | gi|156344376|ref|NP_001621163.1| | 475 |
| *Oryza sativa*(japonica cultivar-group) | Os02g0739700-Os | gi|115448579|ref|NP_001048069.1| | 441 |
|  | Os06g0245900-Os | gi\|115467360\|ref\|NP_001057279.1\| | 425 |
| *Arabidopsis thaliana* | del1-at | gi|30692988|ref|NP_851012.1| | 379 |
|  | del2-at | gi\|15242176\|ref\|NP_197000.1\| | 359 |

**Table-S3. RB family proteins**

| Organism | Protein Name used in this study | Genbank Accession Number | Length |
| --- | --- | --- | --- |
| *Homo sapiens* | rb1-hs | gi|108773787|ref|NP_000312.2| | 928 |
|  | rbl1-hs | gi\|34577079\|ref\|NP_002886.2\| | 1068 |
|  | rbl2-hs | gi\|172072597\|ref\|NP_005602.3\| | 1139 |
| *Canis familiaris* | Rb1-cf | gi|73989274|ref|NP_534118.2| | 897 |
|  | rbl1-cf | gi\|73992372\|ref\|NP_534412.2\| | 1068 |
|  | rbl2-cf | gi\|73949852\|ref\|NP_535303.2\| | 1139 |
| *Bos taurus* | rb1-bt | gi|116004031|ref|NP_001070375.1| | 928 |
|  | rbl1-bt | gi\|119905907\|ref\|NP_603207.3\| | 1068 |
|  | rbl2-bt | gi\|148225699\|ref\|NP_001091542.1\| | 1140 |
| *Mus musculus* | rb1-mm | gi|188528630|ref|NP_033055.2| | 921 |
|  | rbl1-mm | gi\|213417847\|ref\|NP_035379.2\| | 1063 |
|  | rbl2-mm | gi\|170932488\|ref\|NP_035380.3\| | 1135 |
| *Rattus norvegicus* | rb1-rn | gi|109501744|ref|NP_344435.3| | 920 |
|  | rbl1-rn | gi\|109469134\|ref\|NP_001055763.1\| | 1063 |
|  | rbl2-rn | gi\|13592041\|ref\|NP_112356.1\| | 1135 |
| *Gallus gallus* | rb-gg | gi|45383327|ref|NP_989750.1| | 921 |
|  | rbl1-gg | gi\|118100471\|ref\|NP_417312.2\| | 1060 |
|  | rbl2-gg | gi\|118123617\|ref\|NP_414087.2\| | 979 |
| *Xenopus tropicalis* | rbl1-xt | gi|213982839|ref|NP_001135587.1| | 1048 |
| *Danio rerio* | rb1-dr | gi|118150572|ref|NP_001071248.1| | 903 |
|  | rbl1-dr | gi\|194578849\|ref\|NP_001124082.1\| | 1058 |
|  | rbl2-dr | gi\|189537876\|ref\|NP_001922168.1\| | 1009 |
| *Tetraodon nigroviridis* | rb1-tn | ENSTNIT00000014938 | 917 |
|  | rbl1-tn | ENSTNIT00000009683 | 1036 |
| *Ciona intestinalis* | rbl-ci | gi|198437827|ref|NP_002124769.1| | 1023 |
| *Strongylocentrotus purpuratus* | rb1-sp | gi|115660767|ref|NP_790323.2| | 774 |
|  | rbl-sp | gi\|115968799\|ref\|NP_001194030.1\| | 665 |
| Branchiostoma floridae | rb1-bf | gi|260800656|ref|NP_002595214.1| | 305 |
|  | rbl-bf | gi\|260834765\|ref\|NP_002612380.1\| | 1053 |
| Rrosophila melanogaster | RBF1-dm | gi|24638969|ref|NP_525036.2| | 845 |
|  | RBF2-dm | gi17737995\|ref\|NP_524372.1\| | 783 |
| Caenorhabditis elegans | lin35-ce | gi|17508261|ref|NP_491686.1| | 961 |
| Nematostella vectensis | rbL-nv | gi|156399369|ref|NP_001638474.1| | 1083 |
| Trichoplax adhaerens | RBL-Ta | gi|196011866| | 960 |
|  | RB1-Ta | gi\|196012646\| | 820 |
| Monosiga brevicollis | rb-mb | gi|167523296| mb|a9uzq5| | 951 |
| Dictyostelium discoideum ax4 | rb-dd | gi|60463973|gb|eal62136.1| | 1312 |
| Oryza sativa (japonica cultivar-group) | Os08g0538700-Os | gi|115477553|ref|NP_001062372.1| | 1010 |
|  | Os11g0533500-Os | gi\|115485753\|ref\|NP_001068020.1\| | 978 |
| Arabidopsis thaliana | rbr1-at | gi|30682129|ref|NP_566417.3| | 1013 |
| Ostreococcus tauri | Rb-Ot | gi|116055560|emb|CAL58228.1| | 962 |

**Table-S4. DP family proteins**

| Organism | Protein name used in this study | Genbank Accession Number | Length |
| --- | --- | --- | --- |
| *Homo sapiens* | dp1-hs | gi|6005900|ref|NP_009042.1| | 410 |
|  | dp2-hs | gi\|5454112\|ref\|NP_006277.1\| | 386 |
|  | dp3-hs | gi\|189409125\|ref\|NP_057605.3\| | 405 |
| *Canis familiaris* | dp1-cf | gi|73989562|ref|NP_849597.1| | 426 |
|  | dp2-cf | gi\|73990311\|ref\|NP_852372.1\| | 446 |
| *Bos taurus* | dp1-bt | gi|115496726|ref|NP_001069497.1| | 410 |
|  | dp2-bt | ENSBTAP00000008169 | 385 |
| *Mus musculus* | dp1-mm | gi|6678305|ref|NP_033387.1| | 410 |
|  | dp2-mm | gi\|182765448\|ref\|NP_848782.2\| | 385 |
|  | dp2l-mm | gi\|149260219\|ref\|NP_001481321.1\| | 447 |
| *Rattus norvegicus* | dp1-rn | gi|71043770|ref|NP_001020889.1| | 395 |
|  | dp2-rn | gi\|157818883\|ref\|NP_001100317.1\| | 385 |
| *Gallus gallus* | dp1-gg | gi|50730508|ref|NP_416938.1| | 411 |
|  | dp2-gg | gi\|118095071\|ref\|NP_422598.2\| | 445 |
| *Xenopus tropicalis* | dp1-xt | gi|58332126|ref|NP_001011211.1| | 411 |
|  | dp2-xt | gi\|166158068\|ref\|NP_001107442.1\| | 374 |
| *Danio rerio* | dp1-dr | gi|41152118|ref|NP_957070.1| | 386 |
|  | dp2-dr | gi\|38016161\|ref\|NP_937851.1\| | 409 |
|  | dp3-dr | gi\|189523624\|ref\|NP_001918928.1\| | 409 |
| *Tetraodon nigroviridis* | dp-tn | ENSTNIT00000017108 | 408 |
| *Strongylocentrotus purpuratus* | dp-sp | gi|72048148|ref|NP_798717.1| | 500 |
| *Ciona intestinalis* | dp-ci | gi|118343721|ref|NP_001071681.1| | 534 |
| *Branchiostoma floridae* | dp-bf | gi|260816838|ref|NP_002603294.1| | 359 |
| *Nematostella vectensis* | dp-nv | gi|156375187|ref|NP_001629963.1| | 280 |
| *Trichoplax adhaerens* | DP-Ta | gi|196015549|ref|XP_002117631.1| | 232 |
| *Monosiga brevicollis mx1* | dp-mb | gi|167516980|ref|NP_001742831.1| | 310 |
| *Drosophila melanogaster* | dp-dm | gi|17136994|ref|NP_477039.1| | 445 |
| *Caenorhabditis elegans* | dpl-1-ce | gi|17532739|ref|NP_495957.1| | 598 |
| *Dictyostelium discoideum ax4* | dp-dd | gi|66818451|ref|NP_642885.1| | 657 |
| *Oryza sativa* (japonica cultivar-group) | Os03g0152100-Os | gi|115450779|ref|NP_001048990.1| | 379 |
|  | Os10g0440100-Os | gi\|115482194\|ref\|NP_001064690.1\| | 346 |
|  | Os01g0678700-Os | gi\|115439161\|ref\|NP_001043860.1\| | 294 |
| *Arabidopsis thaliana* | dpa-At | gi|22326573|ref|NP_195867.2| | 292 |
|  | dpb-at | gi\|30680032\|ref\|NP_850757.1\| | 385 |
| *Ostreococcus tauri* | DP-Ot | gi|116060017|emb|CAL56076.1| | 361 |

**Sequences for E2F1-6, E2F7/8, RB, and DP family**

**Sequences for E2F1-6 family**

>E2F1-Hs gi|12669911|ref|NP_005216.1| E2F transcription factor 1 [Homo sapiens]

MALAGAPAGGPCAPALEALLGAGALRLLDSSQIVIISAAQDASAPPAPTGPAAPAAGPCDPDLLLFATPQAPRPTPSAPR

PALGRPPVKRRLDLETDHQYLAESSGPARGRGRHPGKGVKSPGEKSRYETSLNLTTKRFLELLSHSADGVVDLNWAAEVL

KVQKRRIYDITNVLEGIQLIAKKSKNHIQWLGSHTTVGVGGRLEGLTQDLRQLQESEQQLDHLMNICTTQLRLLSEDTDS

QRLAYVTCQDLRSIADPAEQMVMVIKAPPETQLQAVDSSENFQISLKSKQGPIDVFLCPEETVGGISPGKTPSQEVTSEE

ENRATDSATIVSPPPSSPPSSLTTDPSQSLLSLEQEPLLSRMGSLRAPVDEDRLSPLVAADSLLEHVREDFSGLLPEEFI

SLSPPHEALDYHFGLEEGEGIRDLFDCDFGDLTPLDF

>E2F2-Hs gi|4758226|ref|NP_004082.1| E2F transcription factor 2 [Homo sapiens]

MLQGPRALASAAGQTPKVVPAMSPTELWPSGLSSPQLCPATATYYTPLYPQTAPPAAAPGTCLDATPHGPEGQVVRCLPA

GRLPAKRKLDLEGIGRPVVPEFPTPKGKCIRVDGLPSPKTPKSPGEKTRYDTSLGLLTKKFIYLLSESEDGVLDLNWAAE

VLDVQKRRIYDITNVLEGIQLIRKKAKNNIQWVGRGMFEDPTRPGKQQQLGQELKELMNTEQALDQLIQSCSLSFKHLTE

DKANKRLAYVTYQDIRAVGNFKEQTVIAVKAPPQTRLEVPDRTEDNLQIYLKSTQGPIEVYLCPEEVQEPDSPSEEPLPS

TSTLCPSPDSAQPSSSTDPSIMEPTASSVPAPAPTPQQAPPPPSLVPLEATDSLLELPHPLLQQTEDQFLSPTLACSSPL

ISFSPSLDQDDYLWGLEAGEGISDLFDSYDLGDLLIN

>E2F3-Hs gi|4503433|ref|NP_001940.1| E2F transcription factor 3 [Homo sapiens]

MRKGIQPALEQYLVTAGGGEGAAVVAAAAAASMDKRALLASPGFAAAAAAAAAPGAYIQILTTNTSTTSCSSSLQSGAVA

AGPLLPSAPGAEQTAGSLLYTTPHGPSSRAGLLQQPPALGRGGSGGGGGPPAKRRLELGESGHQYLSDGLKTPKGKGRAA

LRSPDSPKTPKSPSEKTRYDTSLGLLTKKFIQLLSQSPDGVLDLNKAAEVLKVQKRRIYDITNVLEGIHLIKKKSKNNVQ

WMGCSLSEDGGMLAQCQGLSKEVTELSQEEKKLDELIQSCTLDLKLLTEDSENQRLAYVTYQDIRKISGLKDQTVIVVKA

PPETRLEVPDSIESLQIHLASTQGPIEVYLCPEETETHSPMKTNNQDHNGNIPKPASKDLASTNSGHSDCSVSMGNLSPL

ASPANLLQQTEDQIPSNLEGPFVNLLPPLLQEDYLLSLGEEEGISDLFDAYDLEKLPLVEDFMCS

>E2F4-Hs gi|12669915|ref|NP_001941.2| E2F transcription factor 4 [Homo sapiens]

MAEAGPQAPPPPGTPSRHEKSLGLLTTKFVSLLQEAKDGVLDLKLAADTLAVRQKRRIYDITNVLEGIGLIEKKSKNSIQ

WKGVGPGCNTREIADKLIELKAEIEELQQREQELDQHKVWVQQSIRNVTEDVQNSCLAYVTHEDICRCFAGDTLLAIRAP

SGTSLEVPIPEGLNGQKKYQIHLKSVSGPIEVLLVNKEAWSSPPVAVPVPPPEDLLQSPSAVSTPPPLPKPALAQSQEAS

RPNSPQLTPTAVPGSAEVQGMAGPAAEITVSGGPGTDSKDSGELSSLPLGPTTLDTRPLQSSALLDSSSSSSSSSSSSSN

SNSSSSSGPNPSTSFEPIKADPTGVLELPKELSEIFDPTRECMSSELLEELMSSEVFAPLLRLSPPPGDHDYIYNLDESE

GVCDLFDVPVLNL

>E2F5-Hs gi|134142811|ref|NP_001942.2| E2F transcription factor 5 isoform 1 [Homo sapiens]

MAAAEPASSGQQAPAGQGQGQRPPPQPPQAQAPQPPPPPQLGGAGGGSSRHEKSLGLLTTKFVSLLQEAKDGVLDLKAAA

DTLAVRQKRRIYDITNVLEGIDLIEKKSKNSIQWKGVGAGCNTKEVIDRLRYLKAEIEDLELKERELDQQKLWLQQSIKN

VMDDSINNRFSYVTHEDICNCFNGDTLLAIQAPSGTQLEVPIPEMGQNGQKKYQINLKSHSGPIHVLLINKESSSSKPVV

FPVPPPDDLTQPSSQSLTPVTPQKSSMATQNLPEQHVSERSQALQQTSATDISSAGSISGDIIDELMSSDVFPLLRLSPT

PADDYNFNLDDNEGVCDLFDVQILNY

>E2F6-Hs gi|109637795|ref|NP_937987.2| E2F transcription factor 6 [Homo sapiens]

MSQQRPARKLPSLLLDPTEETVRRRCRDPINVEGLLPSKIRINLEDNVQYVSMRKALKVKRPRFDVSLVYLTRKFMDLVR

SAPGGILDLNKVATKLGVRKRRVYDITNVLDGIDLVEKKSKNHIRWIGSDLSNFGAVPQQKKLQEELSDLSAMEDALDEL

IKDCAQQLFELTDDKENERLAYVTYQDIHSIQAFHEQIVIAVKAPAETRLDVPAPREDSITVHIRSTNGPIDVYLCEVEQ

GQTSNKRSEGVGTSSSESTHPEGPEEEENPQQSEELLEVSN

>E2F1-Cf gi|73992245|ref|XP_542963.2| PREDICTED: similar to E2F transcription factor 1 [Canis familiaris]

MEEMAGKFIEMIKSHVSALKAGTRPGTEERTILVKSADLSNEKWLMERWRSLGGHGRSEYGVKRRTDTDPKGSTCRDPES

AGRRGASRATTPGWPGLCRQRRPGAERDRALAAACRHGPAPPPPPATRAARAVSAMAVAGAPAGGPCAPALEALLGAGAL

RLLDSSQIVIISTAQDASAPPAPAGPAAPAAGPRDPDLLLFATPQAPRPTPSAPRPALGRPPVKRRLDLETDHQYLAESS

GPARGRGRHPGKGVKSPGEKSRYETSLNLTTKRFLELLSRSADGVVDLNWAAEVLKVQKRRIYDITNVLEGIQLIAKKSK

NHIQWLGSHAAVGISGRLEGLTQDLRQLQESERQLDHLIHICTTQLRLLAEDSDSQRLAYVTCQDLRSIADPAEQMVMVI

KAPPETQLQAIDSSETFQISLKSKQGPIDVFLCPEESAGGISPGKTPSQGTASGEEDRPVDPATTVPPPSSPPSSPASDP

SQSLLSLEQEPLLSRMGGLRAPVDEDRLSPLVAADSLLEHAREDFSGLLPEEFISLSPPHEALDYHFGLEEGEGIRDLFD

CDFGDLTPLDF

>E2F2-Cf ENSCAFT00000021059 [Canis familiaris]

LHRGTQTSLPARWAGCKALPAMSPTEPWPPGLSSPQLCPATATYCTSLYPQTVPPPAAAGTCLDATPHGPEGQAVRCVPA

GRLPAKRKLDLEGIGRPAIPEFRTPKGKCIRVEGLPSPRTPKSPGEKTRYDTSLGLLTKKFIYLLSESKDGVLDLNWAAE

VLDVQKRRIYDITNVLEGIQLIRKKAKNNIQWVGRGMFEDPTRPGKQQQLGQELKELMSMEQALDQLIQSCSLNFKHLTE

DKANKRLAYVTYQDIRAVGNFKEQTVIAVKAPPQTRLEVPDRNEENLQIYLKSTQGPIEVYLCPEEVQDPDSPAKERLPP

ASTLGPSPDSTQPSSSTDPGMTDPVASSACVFQPSFKAPPPGLPPPKSPSLVPLEATDSMLELPHPLLQQTEDQFLSPTL

PCSSPLISFSPPLDQDDYLWGLDGGEGISDLFDSYDLGDLLIN

>E2F3-Cf gi|74004128|ref|XP_545361.2| PREDICTED: similar to Transcription factor E2F3 (E2F-3) [Canis familiaris]

MPSPVASDVPVPRHGHVQQLTPASVHVRDLEMVHYSPAASSSPNVVSASGGRTSRVSRGQPVSASTPYILERKARQGCCC

PEEDKTVTRTSAENRSGCKRYSPHQDQKRVLQALMIEQKDLRTAGQNKRVLLCGRDRAFQTIFWYEAVVRKQEGAGNMLP

IIEAPDFSACAFTLQAKRRLELGESGHQYLSDGLKTPKGKGRATLRSPDSPKTPKSPSEKTRYDTSLGLLTKKFIQLLSQ

SPDGVLDLNKAAEVLKVQKRRIYDITNVLEGIHLIKKKSKNNVQWMGCSLSEDGGMLAQCQGLSKEVTELSQEEKKLDEL

IQSCTLDLKLLTEDSENQRLAYVTYQDIRKISGLKDQTVIVVKAPPETRLEVPDPIESLQIHLASTQGPIEVYLCPEETE

THSPMKTTNQDHNGNIPKPPSKDLASTNSGHSDCSISMANLSPLASPANLLQQTEDQIPSNLEGPFVNLLPPLLQEDYLL

SLGEEEGISDLFDAYDLEKLPLVEDFMCS

>E2F4-Cf gi|73957515|ref|XP_853975.1| PREDICTED: similar to E2F transcription factor 4 [Canis familiaris]

MAEAGPQAPPPPGTPSRHEKSLGLLTTKFVSLLQEAKDGVLDLKLAADTLAVRQKRRIYDITNVLEGIGLIEKKSKNSIQ

WKGVGPGCNTREIADKLIELKAEIEELQQREQELDKHKVWVQQSIRNVTEDVQNSCLAYVTHEDICRCFAGDTLLAIRAP

SGTSLEVPIPEGLNGQKKYQIHLKSVSGPIEVLLVNKEAWSSPPVAVPVPPPEDLLQSPPAVSTPPPLPKPTLAQPHDTS

RPSSPQLTTPTPVTGITEAQGVPGPAAEIAVSGGPGTDSKDGGELGSLPPGLAALDTRPLQSSALLDSSSSSSSSSSSNS

SSSGPNPSTSFEPIKADPTGVLELPKELSEIFDPTRECMSSELLEELMSSEVFAPLLRLSPPPGDHDYIYNLDESEGVCD

LFDVPVLNL

>E2F5-Cf gi|73999542|ref|XP_853833.1| PREDICTED: similar to Transcription factor E2F5 (E2F-5) [Canis familiaris]

MGVKAFVRFRAIVPKQLTRSNLGLGSHAVNLIINLFLAADTLAVRQKRRIYDITNVLEGIDLIEKKSKNSIQWKGVGAGC

NTKEVIDRLRYLKAEIEDLELKERELDQQKLWLQQSIKNVMDDSINNRFSYVTHEDICNCFNGDTLLAIQAPSGTQLEVP

IPEMGQNGQKKYQINLKSHSGPIHVLLINKESSSSKPVVFPVPPPDDLTQPSSQPSTPVTAQKSNIATQNLPEHVSERSQ

NLQQTPATDLSSAGSISGDIIDELMSSDVFPLLRLSPTPADDYNFNLDDNEGVCDLFDVQILNY

>E2F6-Cf gi|73980432|ref|XP_852463.1| PREDICTED: similar to E2F transcription factor 6 isoform 1 [Canis familiaris]

MSQQRPARKLPSLLVDPAEETVRRRCRDPINVEGLLPSKIRINLEDNVQYVSMRKALKVKRPRFDVSLVYLTRKFMDLVR

SAPGGILDLNKVATKLGVRKRRVYDITNVLDGIDLVEKKSKNHIRWIGSDLSNFGAVPQQKKLQEELSDLSAMEDALDEL

IKDCAQQLFELTDDKENERLAYVTYQDIHSIQAFHEQIVIAVKAPAETRLDVPAPREDSITVHIRSTRGPIDVYLCEVEQ

GHSSNKSSEGAGTSSSKNKHPEPPNKEENPPQQSEELLEVSN

>E2F1-Bt gi|194672360|ref|XP_615437.4| PREDICTED: E2F transcription factor 1 [Bos taurus]

MAVAGAPAGGSCAPALEALLGAGALRLLDSSQIVIISTAQDASAPPAPAGPAAPAAGPRDPDLLLFATPQAPRPTPSAPR

PALGRPPVKRRLNLETDHQYLAESSGPARGRGRHPGKGVKSPGEKSRYETSLNLTTKRFLELLSRSADGVVDLNWAAEVL

KVQKRRIYDITNVLEGIHLIAKKSKNHIQWLGSHATVGISGRLEGLTQDLQQLQESEQQLDHLLHTCSTQLRLLSEDADS

QRLAYVTCQDLRSIADPAEQMVMVIKAPPETQLQAVDSSENFQISLKSKQGPIDVFLCPEESVGGTSPGKTPSQGAASGE

EDRTADLATAVPPPPSSPRSSPATDPSQSLLSLEQEPLLSRMGGLRAPVDEDRLSPLVAADSLLEHVKEDFSSLLPEEFI

TLSPPHEALDYHFGLEEGEGIRDLFDCDFGDLTPLDF

>E2F2-Bt gi|76611569|ref|XP_874289.1| PREDICTED: similar to E2F transcription factor 2 [Bos taurus]

MLLKLCLSLLSLPKAKRKLDLEGIGRPTVPEFRTPKGKCIRVDGLPSPKTPKSPGEKTRYDTSLGLLTKKFIYLLSESED

GVLDLNWAAEVLDVQKRRIYDITNVLEGIQLIRKKAKNNIQWVGRGLFEDPTRPGKQQQLGQELKELMNMEQALDQLIHS

CSLNFKHLTEDKANKRLAYVTYQDIRAVGNFKEQTVIAVKAPPQTRLEVPDRSEENLQIHLKSTQGPIEVYLCPEEVQEP

HSPAKEPLPSTSALSPSPDSTQLNSNSDPGITEPTASSEPALTSPQVPPPPPPPLVPLEATENMLELPHPLLQQTEDQFL

SPTLPCSSPLISFSPPLDQDDYLWGLDGGEGISDLFDTYDLGDLLIN

>E2F3-Bt gi|76663083|ref|XP_614932.2| PREDICTED: similar to Transcription factor E2F3 (E2F-3) [Bos taurus]

MRKGIQPALEQYLVTAGGGEGAAVVAAAAAASMDKRALLASPGFPAAAAAAPSAYIQILTTNTSTTSCSSSLQSGAVAAG

PLLPSAPGVEQTAGSLIYTTPHGPSGRAGLLQQPPALGRGGSGGGGGPPAKRRLELGESGQQYLSDGLKTPKGKGRAALR

SPDSPKTPKSPSEKTRYDTSLGLLTKKFIQLLSQSPDGVLDLNKAAEVLKVQKRRIYDITNVLEGIHLIKKKSKNNVQWM

GCSLSEDGGMLAQCQGLSKEVTELSQEEKKLDELIQSCTLDLKLLTEDSENQRLAYVTYQDIRKISGLKDQTVIVVKAPP

ETRLEVPDPIESLQIHLASTQGPIEVYLCPEETETHSPMKTNNQDHNGNIPKPTSKDLASTNSGHSDCSISMANLSPLAS

PANLLQQTEDQIPSNLEGPFVNLLPPLLQEDYLLSLGEEEGISDLFDAYDLEKLPLVEDFMCS

>E2F4-Bt gi|115497534|ref|NP_001069341.1| E2F transcription factor 4 [Bos taurus]

MAEAGPQAPPPPGTPSRHEKSLGLLTTKFVSLLQEAKDGVLDLKLAADTLAVRQKRRIYDITNVLEGIGLIEKKSKNSIQ

WKGVGPGCNTREIADKLIELKAEIEELQQREQELDQHKVWVQQSIRNVTEDVHNSCLAYVTHEDICRCFAGDTLLAIRAP

SGTSLEVPIPEGLNGQKKYQIHLKSVSGPIEVLLVNKEAWSSPPVAVPVPPPEDLLQNPPAVSTPPLLPKPSLAQPQDAS

RPSSPQATTPNPVPSSTEAQGVAGPAAEIPVSGGHGTESKDSGELSSLPLGLAALDTRPLQSSALLDSSSSSSNSSSSGP

NPSTSFEPIKEDPTGVLELPKELSEIFDPTRECMSSELLEELMSSEVFAPLLRLSPPPGDHDYIYNLDESEGVCDLFDVP

VLNL

>E2F6-Bt gi|116003911|ref|NP_001070316.1| E2F transcription factor 6 [Bos taurus]

MSQQRPARKLPSLLVDPAEETVRRRCRDPINVEGLLPSKIRINLEDNVQYVSMRKALKVKRPRFDVSLVYLTRKFMDLVR

SAPGGILDLNKVATKLGVRKRRVYDITNVLDGIDLVEKKSKNHIRWIGSDLSNFGAVPQQKKLQEELSDLSAMEDALDEL

IKDCAQQLFELTDDKENERLAYVTYQDIHSIQAFHEQIVIAVKAPAETRLDVPAPKEDSITVHIRSTKGPIDVYLCEVEQ

GSHSSNKTSDNVGTSSSKSKPLEHPQPEKEENPPQQSEEVLEVSN

>E2F1-Mm gi|6681243|ref|NP_031917.1| E2F transcription factor 1 [Mus musculus]

MAVAPAGGQHAPALEALLGAGALRLLDSSQIVIISTAPDVGAPQLPAAPPTGPRDSDVLLFATPQAPRPAPSAPRPALGR

PPVKRRLDLETDHQYLAGSSGPFRGRGRHPGKGVKSPGEKSRYETSLNLTTKRFLELLSRSADGVVDLNWAAEVLKVQKR

RIYDITNVLEGIQLIAKKSKNHIQWLGSHTMVGIGKRLEGLTQDLQQLQESEQQLDHLMHICTTQLQLLSEDSDTQRLAY

VTCQDLRSIADPAEQMVIVIKAPPETQLQAVDSSETFQISLKSKQGPIDVFLCPEESADGISPGKTSCQETSSGEDRTAD

SGPAGPPPSPPSTSPALDPSQSLLGLEQEAVLPRMGHLRVPMEEDQLSPLVAADSLLEHVKEDFSGLLPGEFISLSPPHE

ALDYHFGLEEGEGIRDLFDCDFGDLTPLDF

>E2F2-Mm gi|29244208|ref|NP_808401.1| E2F transcription factor 2 [Mus musculus]

MLRAPRTLAPATAQPTKSLPALNPTELWPSGLSSPQLCPATTATTYYTSLYTQTVPSSVALGTCLDATPHGPEGQIVRCA

PAGRLPAKRKLDLEGIGRPTVPEFRTPKGKCIRVDGLPSPKTPKSPGEKTRYDTSLGLLTKKFIYLLSESEDGVLDLNWA

AEVLDVQKRRIYDITNVLEGIQLIRKKSKNNIQWVGRELFEDPTRPSRQQQLGQELKELMNAEQTLDQLIQSCSLSFKHL

TEDNANKKLAYVTYQDIRAVGNFKEQTVIAVKAPPQTRLEVPDRAEENLQIYLKSTQGPIEVYLCPEEGQEPDSPAKEAL

PSTSALSPIPDCAQPGCSTDSGIAETIEPSVLIPQPIPPPPPPPLPPAPSLVPLEATDNMLELSHPLLQQTEDQFLSPIL

AANSPLISFSPPLDQDEYLWGMDEGEGISDLFDSYDLGDLLIN

>E2F3-Mm gi|83523736|ref|NP_034223.1| E2F transcription factor 3 [Mus musculus]

MRKGIQPALEQYLVTAGGGEGAAVVAAAAAASMDKRALLASPGFAAAAAPGTYIQILTTNPSTTSCATSLQSGALTAGPL

LPSVPGTEPAASSLYTTPQGPSSRVGLLQQPPAPGRGGGGGPPAKRRLELGESGHQYLSDGLKTPKGKGRAALRSPDSPK

TPKSPSEKTRYDTSLGLLTKKFIQLLSQSPDGVLDLNKAAEVLKVQKRRIYDITNVLEGIHLIKKKSKNNVQWMGCSLSE

DGGMLAQCQGLSKEVTELSQEEKKLDELIQSCTLDLKLLTEDSENQRLAYVTYQDIRKISGLKDQTVIVVKAPPETRLEV

PDSIESLQIHLASTQGPIEVYLCPEETETHRPMKTNNQDHNGNIPKPTSKDLASNNSGHSDCSVSTANLSPLASPANLLQ

QTEDQIPSNLEGPFVNLLPPLLQEDYLLSLGEEEGISDLFDAYDLEKLPLVEDFMCS

>E2F4-Mm gi|22507329|ref|NP_683754.1| E2F transcription factor 4 [Mus musculus]

MAEAGPQAPPPPGTPSRHEKSLGLLTTKFVSLLQEAKDGVLDLKLAADTLAVRQKRRIYDITNVLEGIGLIEKKSKNSIQ

WKGVGPGCNTREIADKLIELKAEIEELQQREQELDQHKVWVQQSIRNVTEDVQNSCLAYVTHEDICRCFAGDTLLAIRAP

SGTSLEVPIPEGLNGQKKYQIHLKSMSGPIEVLLVNKEAWSSPPVAVPVPPPDDLLQSPPAVSTPPPLPKPALAQPQESS

PPSSPQLTTPTPVLGSTQVSEVACQTSEIAVSGSPGTENKDSGEVSSLPLGLTALDTRPLQSSALLDSSSSSSSSSSSSS

SSSSGPNPSTSFEPIKADPTGVLDLPKELSEIFDPTRECMSSELLEELMSSEVFAPLLRLSPPPGDHDYIYNLDESEGVC

DLFDVPVLKL

>E2F5-Mm gi|31982405|ref|NP_031918.2| E2F transcription factor 5 [Mus musculus]

MAAAEPTSSAQPTPQAQAQPPPHGAPSSQPSAALAGGSSRHEKSLGLLTTKFVSLLQEAQDGVLDLKAAADTLAVRQKRR

IYDITNVLEGIDLIEKKSKNSIQWKGVGAGCNTKEVIDRLRCLKAEIEDLELKERELDQQKLWLQQSIKNVMEDSINNRF

SYVTHEDICNCFHGDTLLAIQAPSGTQLEVPIPEMGQNGQKKYQINLKSHSGPIHVLLINKESSSSKPVVFPVPPPDDLT

QPSSQSSTSVTPQKSTMAAQNLPEQHVSERSQTFQQTPAAEVSSGSISGDIIDELMSSDVFPLLRLSPTPADDYNFNLDD

NEGVCDLFDVQILNY

>E2F6-Mm gi|237681138|ref|NP_150373.2| E2F transcription factor 6 [Mus musculus]

MSQQRTARRQPSLLVDPAQETVRRRCRDPINVENLLPSKIRINLEENVQYVSMRKALKVKRPRFDVSLVYLTRKFMDLVR

SAPGGILDLNKVATKLGVRKRRVYDITNVLDGIELVEKKSKNHIRWIGSDLNNFGAAPQQKKLQAELSDLSAMEDALDEL

IKDCAQQLLELTDDKENERLAYVTYQDIHGIQAFHEQIVIAVKAPEETRLDVPAPREDSITVHIRSTKGPIDVYLCEVEQ

NHSNGKTNDGIGASPSKSSHPQCPEKEDEPPQ

>E2F1-Rn gi|189217865|ref|NP_001094248.1| E2F transcription factor 1 [Rattus norvegicus]

MAVAPAGGQHAPALEALLGAGALRLLDSSQIVIISTAPDVGAPQVPTGPAAPPAGPRDPDVLLFATPQAPRPAPSAPRPA

LGRPPVKRRLDLETDHQYLAGSSGPFRGRGRHPGKGVKSPGEKSRYETSLNLTTKRFLELLSHSADGVVDLNWAAEVLKV

QKRRIYDITNVLEGIQLIAKKSKNHIQWLGSRTMVGIGQRLEGLTQDLQQLQESEQQLDHLMHICTTQLQLLSEDSDIQR

LAYVTCQDLRSIADPAEQMVIVIKAPPETQLQAVDSAETFQISLKSKQGPIDVFLCPEESAEGISPGRTSYQETSGEDRN

ADSGTAGPPPSPPSTSPTLDPSQSLLGLEQEAVLPRIGNLRAPMEEDRLSPLVAADSLLEHVKEDFSGLLPGEFISLSPP

HEAVDYHFGLEEGEGIRDLFDCDFGDLTPLDF

>E2F3-Rn gi|212549627|ref|NP_001131098.1| E2F transcription factor 3 [Rattus norvegicus]

MRKGIQPALEQYLVTAGGGEGAAVVAAAAAASMDKRALLASSGFAAAAAPGTYIQILTTNPSTTSCATSLQSGALAAGPL

LPSVPGTEPAASSLLYTTPQGPSSRAGLLQQPPAAGRGGGGGPPAKRRLELGESGHQYLSDGLKTPKGKGRAALRSPDSP

KTPKSPSEKTRYDTSLGLLTKKFIQLLSQSPDGVLDLNKAAEVLKVQKRRIYDITNVLEGIHLIKKKSKNNVQWILCYIS

RYSKN

>E2F4-Rn ENSRNOT00000021145 [Rattus norvegicus]

MAEAGPQAPPPPGTPSRHEKSLGLLTTKFVSLLQEAKDGVLDLKLAADTLAVRQKRRIYDITNVLEGIGLIEKKSKNSIQ

WKGVGPGCNTREIADKLIELKAEIEELQQREQELDQHKVWVQQSIRNVAEDVQNSCLAYVTHEDICRCFAGDTLLAIRAP

SGTSLEVPIPEGLNGQKKYQIHLKSMSGPIEVLLVNKEAWSSPPVAVPVPPPDDLLQSPPAVSTPPPLPKPALAHPQETS

RPSSPQITTPTPVLGSTEVSDVAGQTAEIAVSGSPGTENKGSGELSSLPLGLTALDTRPLQSSALLDSSSSSSSSSSSSS

SSSSSSGPNPSTSFEPIKADPTGVLDLPKELSEIFDPTRECMSSELLEELMSSEVFAPLLRLSPPPGDHDYIYNLDESEG

VCDLFDVPVLKL

>E2F6-Rn gi|109479090|ref|XP_001069501.1| PREDICTED: similar to E2F transcription factor 6 isoform 2 [Rattus norvegicus]

MSQQRTARKLPSLLVDPAQETVRRRCRDPINVENLLPSKIRINLEENVQYVSMRKALKVKRPRFDVSLVYLTRKFMDLVR

SAPGGILDLNKVATKLGVRKRRVYDITNVLDGIELVEKKSKNHIRWIGSDLNNFGAAPQQKKLQAELSDLSAMEDALDEL

IKDCAQQLLELTDDKENERLAYVTYQDIHGIQAFHEQIVIAVKAPEETRLDVPAPREDSITVHIRSTKGPIDVYLCEVEQ

NHSNGKTADGVGASPSDSKHLQSPEKEDKPLQ

>E2F1-Gg gi|45382583|ref|NP_990550.1| E2F transcription factor 1 [Gallus gallus]

MATAGGAAGLAALLGGASPHLLIVSASEEPAGGCRPDADLLLFATPQPSRPGPAPRRPALGRPPVKRKLNLETDHQYIAE

SLPAARGRARIPGRGAKSPGEKSRYETSLNLTTKRFLELLSQSPDGVVDLNWAAEVLKVQKRRIYDITNVLEGIQLITKK

SKNNIQWLGSQVAAGASSRQRLLEKELRDLQAAERQLDDLIQTCTVRLRLLTEDPSNQHAAYVTCQDLRSIVDPSEQMVM

VIKAPPETQLQVSDPGEAFQVSVRSTQGPIDVFLCPEDSSGVCSPVKSPFKAPAEELSPGSSQQRASPLLHSAQDVNMLL

PEALLPGTALPTKCPTEDVSLSPLASMDTLLEHGKDDFPGFLADEFIALSPPQPQDYHFGLEEGEGISELFDCDFGDFTH

LDF

>E2F3-Gg gi|118086362|ref|XP_418915.2| PREDICTED: similar to E2F-3 transcription factor [Gallus gallus]

MMGGIAATANQAATGATAQATIQTTIPHMDMTTNHMAVGATAPGTSQSTTRDTDTTADQTATGATAPTTVQTTTSVTHTT

TNQMAMGATARATNQKAMGAAATATDPTAAGGAALATDKTAKRRLELGESGHQYLAEGLKTPKGKGRAATRSPDSPKTPK

SPSEKTRYDTSLGLLTKKFIQLLSQSPDGVLDLNRAAEVLKVQKRRIYDITNVLEGIHLIKKKSKNNIQWMGCSLSEDGG

MMAQRQGLTKEVTELTQEEKKLDELIQSCTLDLKLLTEDSENQRLAYVTYQDIRKISGLKDQTVIVVKAPPETRLEVPDP

VESALIHLSSTQGPIEVYLCPEENDALSPMKTYSQDHNGNISKTISKEVASVNSGQGDCSVNMATISPLASPANLLQQTE

DQIPSNFEGPFVNLLPPLLQEDYLLSLGDEEGISDLFDAYDLEKLPSLVDEFIYS

>E2F4-Gg gi|118096144|ref|XP_001231948.1| PREDICTED: hypothetical protein [Gallus gallus]

MAECGPQPPGAGGGGGGGGGGGSGGAGAPSRHEKSLGLLTTKFVSLLQEAKDGVLDLKLAADTLAVRQKRRIYDITNVLE

GIGLIEKKSKNSIQWKGVGPGCNTREIAHKLIELKADIEDLEQQEQELEKQKMWVQQSIKNVTEDVQNNWLAYVTHEDIC

KCFTGDTLLAIRAPSGTRLEVPIPEGPSRQKKYQIHLKSTSGPIDVLLVNKDAWSSPPVVLPVPPPEDLIQCQAVAPSKP

QIPPLAHFQEASVPSSTQPSTPTPSSTQDHSPPEQKDTSTECSISVVESKSSSSDLDPLSNVSAPSGHTARLDTQLLQSS

ASLDSSSVLPSPSASFEPIKPDPAGMLELPKELSEMFDPTRECMNSELLEELMSSEVFAPLLRLSPPPGDHDYIYNLDES

EGVCDLFDVPVLNL

>E2F5-Gg gi|71896455|ref|NP_001026113.1| E2F transcription factor 5 [Gallus gallus]

MAAAAEAAGGGGSSRHEKSLGLLTTKFVSLLQEAKDGVLDLKVAADALAVRQKRRIYDITNVLEGIDLIEKKSKNSIQWK

GVGAGCNTKEVVDRLRYLEAEIEDLELKEKELDQQKLWLQQSIKNVMDDSTNHQFSYVTHEDICNCFNGDTLLAIQAPCG

TQLEVPIPEMGQNGQKKYQINLKSSSGPIHVLLINKESNSSKPMVFPVPPPDDLAQTPSQPVAPVTPLKPTPASENPPEH

GLNQGQQLPETTVDTPSESSLQQNSTTPATPYSSLPDSVLYPSLSGDVTQATTSSNDYQALLPLDVNCILKPNSFDIAKM

EEPAGNIGGDIIDELMSSDGKYLASLLAVIKKVVAWFLYEQHSW

>E2F6-Gg ENSGALT00000031596 [Gallus gallus]

MAAASKWERLRPLRPDTLRLSATPMLDLNLDDDVPVVKKTLKVKRPRFDASLVYLTRKFMDLVKRAPDGVLDLNDVATAL

GVQKRRVYDITSVLDGIDLIQKRSKNHIQWVGSNLDQVVEMAAQRQNLKDELSDLSAMEEALDELIKDCAHQLFDLTDDK

ENAKYPYVTYQDIRSIQAFQKQIVIAIKAPEETRLEIPIPKEDCIKVHVKSTKGPI

>E2F1-Xt ENSXETT00000046332 [Xenopus tropicalis]

IVIISTPDVPAEAAGNPGLSEAELMLFTTPQGPSTSYTAQRPDLGRPPVKRKLDLETVSRYNQEALQTPRGKGKRPLKVC

HSSKKTVKSPGERSRYDTSLHLTTKRFLELLSQSSDGVVDLNWAAQVLNVQKRRIYDITNVLEGIHLITKKSKNHIQWLG

YTSYAEYNSRYQSTLKDCQKLEDQEKQLDKLIHMANTQLKLFKEDTQQCTFGYVTCQDLRSIADPSERMLMVIRYPPDTD

MCVSDPAEAFQMSLKSTQAPIDVFLCPDDSSVTSPTKTSQAEPSSINQAEPLPSVQQPFLPPENEIPLDSTLNCLPLSPS

NLLLDYRDAMPDFLPNDFISLSPASSQEYSFGLQTCEGAAELFNFDCDFSGLTALDFMHCM

>E2F3-Xt gi|58331835|ref|NP_001011109.1| E2F transcription factor 3 [Xenopus tropicalis]

MRKGTQGGHEKLLLPGLQGTDKGSGLAGGFIAGGGGVVGAGGGGYVQIISSPGPPLHHHGLCIPEQAASFIYSTPHGPAT

RAGQLQPALGRPPAKRRLELGDGSHQYLSDGLKTPKGKGRAVTHCPDSPKTPKSPLEKTRYDTSLGLLTKKFIQLLSQSS

DGVVDLNRAAEVLKVQKRRIYDITNVLEGIHLIKKKSKNNIQWMGCTLPDDGGNLAKSQELSKELSELAQEENKLDELIK

NCTLDLKHLTENAENQRLAYVTYQDIRKISGLKEQTVIVIRAPPETRLEVPDPVESLQIHLSSSQGAIEVYLCPEESESS

SPVKQCNQDHNGNVSKPKPHSKDSLSPNMENVNCSVEGISPLTSPTNLLQQTEDQISLNMDAPFVNLLPPLMQEDYLLSL

GDEEGISDLFDAYELEKLPSLDDFMCS

>E2F4-Xt gi|167560905|ref|NP_001107966.1| E2F transcription factor 4, p107/p130-binding [Xenopus tropicalis]

MADPAHLPVTPSRHEKSLGLLTSKFVSLLQEAEDGVLDLKAAADTLAVRQKRRIYDITNVLEGIGLIEKKSKNSIQWKGV

GPGCNTREIADKLIDLKAELADLEQREQELDQQRVWVQQSIKNVTDDVQNTGLAYLTHEDICRCFRGDTLLAIRAPSGTC

LEVPVPENTNGQKKFQIHLKSTTGPIEVLLVNKDTSSSPPVVVPVPPPEDLIQAPATVPSTPQRPALTPQNDTAASPVPA

MPDSPISNSESQDCTTDQTLSMENTASSRLSSIDTCPLQSSASLDNSTDLPDPSTSFQPIKSDLSDVLELPKDISDLFDQ

TKECITSDLLEELMSSEVFAPLLRLSPPPGDHDYVYNLDESEGVCDLFDVPINL

>E2F5-Xt gi|188528909|ref|NP_001120880.1| E2F transcription factor 5, p130-binding [Xenopus tropicalis]

MAEPAGASSRHEKSLGLLTSKFVSLLQEAKDGVLDLKVAADSLAVRQKRRIYDITNVLEGIGLIEKKSKNSIQWNGVGAG

CNTKEVLDRLRNLKAEIEDLELKEKELDQQKAWLQQSIKNVMDSSSNGMYSFVTHEDLCNCFNGDTLLAIQAPSGTQLEV

PIPEMGQNGQKKYQISLKSNSGPIQVLLINKESSSSKPVVFPVPPPDDLTQPNSQPEATASSNKQPAAQSSSLSVKEEQE

SNLTEQAAAGKLNPECPASDLQENTSSSYPELPESILYSALGTDVAQSSAASSDYSPLLPLDVNSILRPNAFDITKMDEQ

EGQISGDLIDELMSSDVFPLLRLSPTPDDYSFNLDDNEGVCDLFDVQILNY

>E2F3-Dr gi|220673319|emb|CAX13650.1| novel protein similar to vertebrate E2F transcription factor 3 (E2F3, zgc:158604) [Danio rerio]

MRKGGSSVPGQVMLTGVVRAEQHGVFTAVSDRRCAVYSNATRIQINTPPSTNNGCVPEASADSLYTTPLQGSTHGTGLRP

TLGRPPAKRRLELDITDHQYSEPAKTLRSRKGALKLKIPKAPKTPPEKTRYDTSLGFLTKKFCQLLAQSSDGVLDLNKAA

IVLNVQKRRLYDITNVLEGVRLIKKKSKNNIQWLGSSLPSDGGLPSPAMQSHSLAREMLALTQEERRLDELIQTCTRNVQ

QMTEEIHSQKYAYVTYQDIRRIKSLKDQTVIAIKAPSETKLEVPDPKESLQVHLSSSKGPIDVFLCTDGGDSGSPLQNGL

DVNGNHPAFLKVSQEAASDGPADNVKMNGNSNASVNGFGPVSGNAPMSPLSSSLSSILQQPEEAIPFVPLSPALLSDEYM

LGLGDEQGISDLFDSCDLDTLALDDLLRI

>E2F4-Dr gi|47087407|ref|NP_998597.1| E2F transcription factor 4 [Danio rerio]

MGESLQPQTPSRHEKSLGLLTTKFVTLLQEAKDGVLDLKAAADTLAVRQKRRIYDITNVLEGIGLIEKKSKNSIQWKGVG

PGCNTREIADKLIDLKLELEDLDRREHELDQQRVWVQQSIKNVTDDSLNSPLAYVTHQDLCNCFKGDTLLAIRAPSGTQL

EVPVPESHVNGQKKYQIHLKSSAGPIEVLLVNKDPSSSSPVVLPVPPPDDMLQNLSTPASTTSAAAAPTKPTANSTPSPA

STCQSPSTTTTSNTAITTTTVPPNTNSGPPAVADTDISSTSTPDTTANPTPSTDTQQLQSSASLDSSSLPDSSTLFEPIK

TDPSDLLDFPKELSEMFDPKEIMSTDLLEELMSSEVFSPLLRLSPPPGDHDYIYNLDETEGLCDLFDVPVANL

>E2F5-Dr gi|68533607|gb|AAH98598.1| Zgc:111879 protein [Danio rerio]

MAESNSASFPHSTPNGSSRHEKSLGLLTVKFVTLLQEAKDGVLDLKVAADSLAVKQKRRIYDITSVLEGIGLIEKKTKNT

IQWKGESTGCQPQEVLEQVELLKANIADLELQERELDMQKACLQQSIKQLNEDPYSCRYSYVMHEDICDAFSGDTLLAVM

APSGTQLEVPVPEMGHNGQKKYQVNLRSHSAPIQVMLINRETSCSKPVVVSVPPIDDISSMPTPPSTPAGLQRFPISSID

LCDQKHGLLKSPAAEHQLTPSSTSPDVHMECNPESPASQCLLMQSSLGGPEEQQRELGGQDLQSMLEEMRDEREGVSNLI

DELMSSDVFPLLRLSPNPGVDYSFNLDDNEGVCDLFDVQILNY

>E2F6-Dr gi|71892405|ref|NP_001025315.1| hypothetical protein LOC560495 [Danio rerio]

MVKCTVQGCINFSDLRPEEQPNRPRKRFFRFPKDKARVKVWLAALRDTEREITDLHRICEDHFLSHHITADGISPDAIPI

MPPLDGPVGNWSSFEEEQDEHTGTLEESGAAADDDLEDEDFEDDEEYEDEGYIPADNNSNSTISKPQEQSVTQSFRPIFS

SPTILPPVNVADDVFNSKAPHRSEVALGQLTKRFMQLLNAAPEGVLDLNEVSRKLGARKRRVYDITSVLAGIHLLKKTSK

NKIQWMSSTPLSSFGSQWSPKAKAELLHLKSTEEALDWLIKDCAQQLFALTDLKDNTSAYVTYEDICQIDVFKDQTIIAI

RAPEETKLEVPTPTEESIKIHLKGSRGPIHTLTCETEGPGDAEDSSNTESKLTKSDCFVSLEESRIQTQPLVPDVSNTVA

AVQSA

>E2F1-Tn ENSTNIT00000007876 [Tetraodon nigroviridis]

LITGQTSEDILADFETLLNSGSIDLGEDHQIVIITSPGNEGLHPGSAPTSTGEILLFATPQGPADVGLQDKRRPALGRPP

VKRKLDLDSDHQYVSTTRPCIGQAPPSTPAPPRVPRNLTEKSRYDTSLNLTTKRFLNLLSQSADGVVDLNWASQVLDVQK

RRIYDITNVLEGIHLISKKSKNHIQWLGNRVDTALVSRHKELQKEVCDLAEAEEQLDQLVSKCSLQLRLLTEDPQNNTWG

YVRCQDLKKSVDSPDQLLMVIRAPPETQMQVSEPSKGFEMSLKSTQGPIDVFLCPEDSSGACSPVTGSSPAKATGPSLVP

EQTTDQSKTRGSTALLEESSSSPASTSSTVTAASQQDSSSLPMGNDSGYVSDMPNFDLSPLSAADFLNGEGFPLPLDGFI

NLSPPHSHDYHFGLEDHEGISELFDCDFGDLTHVLE

>E2F2-Tn ENSTNIT00000020634 [Tetraodon nigroviridis]

MMRMPKGVSAASGRPAAGLSCSQTKMKLLSAGVKTEFFSAGLSSPLMNAVPAGYFTQICNTTAAEQRANSLYSTPHGPEA

KPIRASSGRMPAKRKLDLEDPLYMPEFRTPKGKCNIAARLTSPRTPKSPGERTRYDTSLGLLTKKFVGLIAESPDGVLDL

NWATEVLEVQKRRIYDITNVLEGVQLIRKKSKNHIQWLVGDVFEGGAGGGQKAGALRKELGDLERAEKCLDELILSSTAQ

LKQLTEYEDNQRLGYVTYQDIRSIGSFQDQTVIAVKAPADTKLEVPDTEGQGSLQIYLKSKNGPIEVYLCPEEALEDASP

VKSASTPKKDILQTICPPAPATSSTAPPSCSIKEEPVESKPSSAHVAAASSASILDVEGLLGLPPSLLQITEDQLPSASF

APDPNTPFVSFSPPLDHDDYLWSLDDGEGVSDFFDSYDLGDLLSN

>E2F3-Tn ENSTNIT00000012687 [Tetraodon nigroviridis]

MRRGISSAPEKVILTGVGGSSLDSNIILTTLADRLNPGQSNATFIQIITTPPPCNVTRTNVCLSEPQINNIYTTPQQGAA

NGAGQRPALGRPPAKRRLALDDSDHQHQSEPIRTPRGRGATANGTRIKAPRTPKSPPEKTRYDTSLGLLTKKFVELLGQS

SDGVLDLNLAAETLQVQKRRLYDITNVLEGIHLIKKKSKNNIQWMGCSLLEEEGSLSQRQSLTDEVSALGEEEQRLEQLI

QTCSKDMRCMSELSSNQKFSTYAYITYQDIKQLGNLKDQTVIVVKAPTDTKLEVTDPDESLSIHLTSTKGPIDVLLCPDD

DNDPKSPVKNGGTDINGNSPYPGSVAAAPSPGPPPPAAAAAAVSITTLSPISSPYTSLLQQTEDQIPTSLGPFLNLGPPL

LEQDDYLLGLGDDQGISDLFDACDFDKIRSLGLDDLLCS

>E2F4-1-Tn ENSTNIT00000002389 [Tetraodon nigroviridis]

LGAVGDSLQPQTPSRHEKSLGLLTTKFVTLLQEAKDGVLDLKAAADTLAVRQKRRIYDITNVLEGIGLIEKKSKNSIQWK

GVGPGCNTREIADKLIDLKAELDDLDFRERELDRQRSWVQQSIKNVTDDSNNSPPTLAYVKHEDLCGAFKGGDTLLAIRA

PFGTQLEVPVPEPILKGQRKYQIHLKSSAGPIEVLLVNKDPSSASPVVLPVPPPDEILQSLPTPAATPQPSVSQVLAVRP

PASSPLTQIITPVLAVLMHGYVTNQTFLLYLSREDTHLLSLPPVPDVSSTSSLTPTTDTPVTVTQQLLSSASLDGSASSS

ASAGFEPVKSDPSELLDFPKELSDMFDPTKEIISGDLLEDLMSSEVFSPLLRLSPPPSDHDYIYNLDETEGLCDLFDVPI

LNL

>E2F4-2-Tn ENSTNIT00000002758 [Tetraodon nigroviridis]

VGDSLQPQTPSRHEKSLGLLTTKFVTLLQEAKDGVLDLKAAADTLAVRQKRRIYDITNVLEGIGLIEKKSKNSIQWKGVG

PGCNTREIADKLIDLKAELDDLDFRERELDRQRSWVQQSIKNVTDDSNNSPYPTLAYVKHEDLCGAFKGGDTLLAIRAPF

GTQLEVPVPEPILKGQRKYQIHLKSSAGPIEVLLVNKDPSSASPVVLPVPPPDEILQSLPTPAATVLKTAAAASTRPAAA

AQPCRRPHPQSHLKKLKKRCSVRPPASSPLTQMLGPPPVGVPDVSSTSSLTPTTDTPVTVTQQLLSSASLDGSASSSASA

GFEPVKSDPSELLDFPKELSDMFDPTKEIISGDLLEDLMSSEVFSPLLRLSPPPSDHDYIYNLDETEGLCDLFDVPILNL

>E2F5-Tn ENSTNIT00000004791 [Tetraodon nigroviridis]

RAAPSRREKSLGVLTMKFVSLLQQAEDGVLDLKMAASSLAVKQKRRIYDITNVLEGVGLIEKKNKNIIQWRGKNSSGQTR

EVMEQVKYLKAQNSELEAQESELDNQKARLEENIQLLSHDPISRTYPFVTHEDVCNAFSDETLLAVLAPAGTQLEVPLPE

VGQSGQKYQVNLRSHSAPIQVLLINRDSASSVPVVFSVPPTTNWSQDSLCLDQQMALPERSGMLTPSRTPPDAHTGKDRD

FFRITALFSLSAGPVDLIEELISADYILILFLPPYAGLDYSFNLDDNEGVCDLFDVQVLNY

>E2F6-1-Tn ENSTNIT00000008935 [Tetraodon nigroviridis]

MDRGESRRTGDRLLPGDTCARGAPPDALPVIRFESSDEEGISSEPEGRSKARRSSDPQEHTEGAMMKKFSRSRASLQRLT

RRFLQLMQEAPGCCVDLSSASTRLRTKRRRLYDITNALYGVQVIEKESRNKVRWIGKSPISVFLLNKKKELEKLRQMEAT

LDGLIRRCAQQLFDLTDDDRHSACSLTWAYVTHQDLGLLQTFQEQTVIAVRAPEETKMEVPVPTEDSVQIHLKATQGPIT

VLSCEPGPGRTAEATACFLALEESRIRTNVHAPDRGSSAQDSW

>E2F6-2-Tn ENSTNIT00000013829 [Tetraodon nigroviridis]

MVKCVVSGCPNRLVSGENRSIFNRPPKRFFQFPKDPARVKVWLAALRETQQHDPAEPHFICEDHFLPEDISRNQVSSEAI

PIMPPCVDGGLGMMGPWGGVAEEEDDPWTMDADEEGEEEVGRPAGEAPPSGQRQSTRRHSCSVNKASAVSAWASFFGALT

PPSGDCASPHGFTACNTRRRHRGRCRFGKRLRRFLELMLASPDHLVDVRRLMAGTESSTDRMDDITGVLEDIRLIEKQSA

HRFKWIGKSHISSFLWKNQQEFQAEMEKLKLVESVLDGLIKSCSQQLFEVTDNLDSTLAYVSLADISRLKDFQQQTVMVV

KAPEETKLEVPAPREDSIQVHLKAEQGPILVLTCDIGGGEETYGCFLALEESRIKTAELHTEAPRCAVQSA

>E2F1/2/3-Sp gi|193788689|ref|NP_001123287.1| E2F transcription factor 3 [Strongylocentrotus purpuratus]

MPRGGAVTAGRRQPTEAKIRTILGVSPRGMLPDFSNQPMTTDITNISSNIKEEFDQEILDVVGFTPEFDSTKGMRPQLGR

PPAKRKLELDSAGVRPTNVASSTAFKTPSPRPSAKTKKPRAPTRSPMEKSRYDTSLGLLTKRFVGLLRGAPDGVLDLNRA

AEVLEVQKRRIYDITNVLEGIKLITKKSKNNIQWKGASSSVAIHPGDSQLSAETVNLHSELNDLEAQEKRLDELLRNAST

QLKTLTEDPDNARYAYVTYHDIRGIQSFEDQTVIAIKAPPETRLEVPDPKESTNIQIWLKSTRGQIEVYLCPDDNPDDSS

SSESEAGSKNSSPCSSKGDPALKATALEEDDLSALSRSLLLETEDQNGLDDDFVALSPPAVDDYLFALNDNEGISDLFDA

YDIF

>E2F4/5-Sp gi|115696783|ref|XP_799123.2| PREDICTED: similar to E2F transcription factor 4 [Strongylocentrotus purpuratus]

MATLVETPLSRHEKSLGLLTTKFVGLLQEAPDGVLDLKQAADTLAVRQKRRIYDITNVLEGIGLIEKKSKNSIQWKGGGP

GSNTKEATDRVEELKLELDQLDQIEQELDQQRSRVQQSIRNVTDDVENSRLAYVTHEDLCRCFKGDTLLAVQAPSGTQLE

VPVPERGPDNQKRYMVHLKSFNGPIYVLLVNKDETSNSPVVVPVPPHNLQPPAPAQRITRASRLVPAEPIAQAISAPSVV

VQQQQQQVRQPTPPTVVATPTPVSVIPVAEPMLTETKIKEEVTEESLNGASPESLKDLQTDMLFDPYKEGMYTDDFADPE

TYPLLRLSPAPNDQDYYFNLDDSEGAADLFDLFSQL

>E2F1/2/3-Ci gi|118343729|ref|NP_001071687.1| transcription factor protein [Ciona intestinalis]

MDNSRVFSTTTAREIFTNNLASVSPQCSSKSLPSNELHLTPSQDDTHQWLYGGHDMHGATDMSIVTVESQNNRNTAQVKR

KLELQGSSSGSMNSYLMSESQIQQLQTQNFVNNYNKPPSFKSPLKRAKSVSERKVLATNPPSRKRKMSTAGSVASGSPSE

KSRYDTSLGLLTKRFTQLMRNSSDGILDLNQAADILAVQKRRIYDITNVLEGIGLIEKRSKNNVQWVACPNTESDHSSEI

EKQETQNEVDALRNKEEELDQLIRKRQMELERLSESNTEHSYVTYQDIRGIKSFKEQIVICIKAPQDTKLEVPDPGEKIQ

MLLKSTKGEIDVFLCPDPQDHISPKKEPVEGDSGLLESTSVSDGSQRLSGSQYEENRPLFMQTEDQMPSDEVDSAFIDED

SFIALSPAMNPDDYLFSLDDSEGISELFDMNATSLSGARTS

>E2F4/5-Ci gi|118343737|ref|NP_001071688.1| transcription factor protein [Ciona intestinalis]

MADLASTSRHEKSLGLLTTKFVHLLKNAQNGVLDLKMAADELAVRQKRRIYDITNVLEGIGLIEKRSKNSIQWKGAGPEC

NDGDIAEKINILRQEIQSLEQTELDLDRQKTGVHQSLRNVTDDVDNNKLAYVTHEDLCRCFPGDTLLAIQAPSGTQLEVP

IPEMADPNKRYQVHLKSTSGPVSVLLVNKDTSSPAVVVPVPPPPQAEAAKPTELPLPSMQTDTNFLDDFPCSDTFEPLLS

LSPLPSDQDYYFNLDMSEGACDLFDISLP

>E2F1/2/3-Bf gi|260790430|ref|XP_002590245.1| hypothetical protein BRAFLDRAFT_184832 [Branchiostoma floridae]

KSPLEKTRYDTSLGLLTKKFVGLLGSSPDGIVDLNQAAEVLNVQKRRIYDITNVLEGINLIKKKSKNHIEWRVQSTMEKD

KERLNSAIGLFKTVDTEHIRENLLDELIRHSSTQLKHLTEDSENKKYPFQHGEHYCKCRNDIRSIKTFEEQTVIAIKAPP

ETRLEVPDPRESIQIWLKSSKGPIEVYLCPEEQNTEDAGSSSSDAASSEGCSSTSGSPCSVKGSELKTNEMARSLLPQTE

DQDQEIQDFVALSPPLMEDDYLFSLSPGEGISDLFDAYDL

>E2F4/5-Bf gi|260798626|ref|XP_002594301.1| hypothetical protein BRAFLDRAFT_57021 [Branchiostoma floridae]

MADASPAGPSRHEKSLGLLTTKFVTLLQEAKDGVLDLKVAADTLAVRQKRRIYDITNVLEGIGLIEKKSKNSIQWKGAGP

GCNTTEISNRLGELKDELEALERKEAELDQQRLWVQQSIKNVTEDVENHRLAYVTHEDLCRCFRGDTLLAVQAPSGTQLE

VPIPEAASQPQGKKYQIHLKSHSGPIYVLLVNKDTSSSSPVVVPVPPPDMPGVQQPGTPAKVPQQTVPLVQQGVSAPGKS

GLPQTVAASPMDTMSLPAPTQPSPVKPGLAPTLDPSMDGEFIDELMASEAFAPLLRLSPPPGDHDYHFNLDDSEGVCDLF

DVPMLDL

>de2f1-Dm gi|24648770|ref|NP_524437.2| E2F transcription factor CG6376-PA, isoform A [Drosophila melanogaster]

MSKFFVNVAPINNSNSSSSHTTTSSNTQRHQQHQQHYGGSGTTGHTMVARRLNYDLHGGTTSINNNNNIVIKNESVDLDY

DHVLSSSDSNSNGGVAAHLRDHVYISLDKGHNTGAVATAAAAATAGHTQQQLQQQHHHQNQQQRKATGKSNDITNYYKVK

RRPHAVSDEIHPKKQAKQSAHHQTVYQKHTASSAPQQLRHSHHQLRHDADAELDEDVVERVAKPASHHPFSLSTPQQQLA

ASVASSSSSGDRNRADTSLGILTKKFVDLLQESPDGVVDLNEASNRLHVQKRRIYDITNVLEGINILEKKSKNNIQWRCG

QSMVSQERSRHIEADSLRLEQQENELNKAIDLMRENLAEISQEVENSGGMAYVTQNDLLNVDLFKDQIVIVIKAPPEAKL

VLPNTKLPREIYVKAENSGEINVFLCHDTSPENSPIAPGAGYVGAPGAGCVRTATSTRLHPLTNQRLNDPLFNNIDAMST

KGLFQTPYRSARNLSKSIEEAAKQSQPEYNNICDIAMGQHHNLNQQQQQQQQQLLQQPEEDDVDVELNQLVPTLTNPVVR

THQFQQHQQPSIQELFSSLTESSPPTPTKRRREAAAAAIAAGSSTTATTTLNSHNNRNHSNHSNHSNHSSSNNSKSQPPT

IGYGSSQRRSDVPMYNCAMEGATTTSATADTTAATSRSAAASSLQMQFAAVAESNNGSSSGGGGGGGGYGSIAGAGANAD

PHQPYSHDRNSLPPGVADCDANSNSSSVTLQGLDALFNDIGSDYFSNDIAFVSINPPDDNDYPYALNANEGIDRLFDFGS

DAYGP

>de2f2-Dm gi|17137542|ref|NP_477355.1| E2F transcription factor 2 CG1071-PA [Drosophila melanogaster]

MYKRKTASIVKRDSSAAGTTSSAMMMKVDSAETSVRSQSYESTPVSMDTSPDPPTPIKSPSNSQSQSQPGQQRSVGSLVL

LTQKFVDLVKANEGSIDLKAATKILDVQKRRIYDITNVLEGIGLIDKGRHCSLVRWRGGGFNNAKDQENYDLARSRTNHL

KMLEDDLDRQLEYAQRNLRYVMQDPSNRSYAYVTRDDLLDIFGDDSVFTIPNYDEEVDIKRNHYELAVSLDNGSAIDIRL

VTNQGKSTTNPHDVDGFFDYHRLDTPSPSTSSHSSEDGNAPACAGNVITDEHGYSCNPGMKDEMKLLENELTAKIIFQNY

LSGHSLRRFYPDDPNLENPPLLQLNPPQEDFNFALKSDEGICELFDVQCS

>efl-1-Ce gi|17559226|ref|NP_507289.1| E2F-like (mammalian transcription factor) family member (efl-1) [Caenorhabditis elegans]

MEDSYNDMEDPGFRQLSDMELQKALEMTKQSSIKNNLMLGLDNELDFDFDFDEDEDLDQPQMGTRADKSLGLLAKRFIRM

IQYSPYGRCDLNTAAEALNVRQKRRIYDITNVLEGIGLIEKRSKNMIQWKGGDFMLNVKEGKRQSATTEEEDRMEQLKAE

IEQLNKEEELIEQRQRWLQQSLRNMTESVENNKLSYVLRSQLAEIQGSDLTIGIQTRVGTQVRLSDPEQVEIHGGPSWCY

LKDPSGPLRAAIVSNHELHDFVQRERAKRPGEEHVDADAPDEMMDDSRYRNRRTINDDEMFGFEQKVPAMKHLEPPPASD

DYVYSSTGDEYRGDSIVDLYGD

>efl-2-Ce gi|71998283|ref|NP_496825.3| E2F-like (mammalian transcription factor) family member (efl-2) [Caenorhabditis elegans]

MDPSSIDLGSIPGGSEQQQHEDGAAAAAGSPKEVFRSQQSLGLITQRFMSLRQRNEVLNLNEVAKELNISKRRVYDVINV

LEGLGYVEKVEKNNIRWIGDNNNSEEQNALEARVEMLRQQEKLLELMIRDAQAIIELHFEDPIERPYNYVSKEDIRKTAE

PNTKSIIVKSELDTSSSFEIQVTDPSTSGSHDMIVRNKNGVKSHALLFTNDPPEYGDEEDEDDVKKLSSQKRFETSEALR

VTLAEMLTDPTTISSSPKHIKLEEPEEIDTEDLETFAPPEFSENKWSQKIGGGLAILWAWLALMPRPQNGNFFSAKFFKF

FPETVARKTFVLPPSTSASQSNAPYLPETPGRGLFFSPFKSLIDPCILPSFSDDLTSGYINITTPSRATGAGAGASGQED

EEPVSVMDFFSD

>E2F-4/5-Nv gi|156368461|ref|XP_001627712.1| predicted protein [Nematostella vectensis]

MADFGSPGTPSRHEKSLGLLTTKFVSLLQEAKDGVLDLKVAADTLAVRQKRRIYDITNVLEGIGLIEKKSKNSIQWKGAG

PGCNTREISDKLVVLKKELEALDEEERKLDEQRAWVQQSLKNISEDPENEKLAFVTYDDVCKSFKGDTLLAIQAPSGTQL

EVPIPEQVPGMPKKYQIHLKSQNGPIHVLLVNKDAAGDSPVVTPVPPLAEENGNNDTNPEEPMEAETNGAASSCSSLENA

PAKVKTEVLDTSQAKELKDVANDIIFGNGKDPGKIADEVIDELMSAEVYAPLLRLSPPPADHDYYFNLDDNEGVCDLFDI

PNLDMNMSLQGTMAT

>E2F1/2/3-Nv gi|156371340|ref|XP_001628722.1| predicted protein [Nematostella vectensis]

MATTRRHSIGHSTRAPLRSQTSQINSSASKNGETVKSFTRLKAKEAANSEGYKHLQASFTPDSCTQKDRYASQQRPQVKR

RLDLDESPQKYVPAGFKTPKVRRQKRMRRESEPYPMPKLIATSPLEKTRYDTSLGILTKKFVGLIRASEDGVLDLNHAAE

VLSVQKRRIYDITNVLEGIGLIEKKSKNNIKWRGVNLHGEEMQAQISPQLMDLHTDLADLDAKENQLDQLIANCRAELKQ

LTEDPETSKYPFMHEKYITLNIM

>E2F4/5-Ta gi|221125072|ref|XP_002154174.1| PREDICTED: similar to predicted protein [Hydra magnipapillata]

MLISLQIQFQCHENTMASDYNSPGTPSRHEKSLGLLTAKFVGLLQEAKDGVLDLKVAADQLAVRQKRRIY

DITNVLEGIGLIEKKSKNSIQWKGAGPGCNSQEISDKLCELKGEISKLDAVETILDQQQLWVQQSLKNIS

EDPENERHAYVSHEDVCMCFKGETLLAIQAPSGTQLEVPPPDFVSSHTANYQMHLNSENGPICVLLVNHD

AKSDVPVVTNVSPESPNNCAECMVSTKDRDALKTNGEDMETDDEIEDMNLDQFCKENELFAPLLRLSPPP

GEHDYYFNLDDNEGVCDLFDVPALKIN

>E2F1/2/3-Ta gi|196010483|ref|XP_002115106.1| hypothetical protein TRIADDRAFT_58918 [Trichoplax adhaerens]

MTPTEFDNNTMRKQMHGSKFFVDNTHVIIAGVTSIQHRNRSPPTRGRNFLFLLGHLENPIKCWTIMELSK

KSKDDPARQQELLMQIKAKRRLDMDQTLNKNNQNVFKTPTSSGRGRRRGRKASTVKAAPVKTTTPPSESK

RYDTSLGLLTKKFVVLLREARDGVLNLNNAADNLTVQKRRIYDITNVLEGVGLIEKKSKNNVQWKGFQSW

KCGKINIPANSANETGLKNLHTADDFRCQIKKLREDEKTLDSMIAKLEEENKACKISDEALKYAYVTYND

ITSIKDFSNQTIIAIKASKDTLLETTEDRQVWLKSNTAPIDVYLCSDGSQSCGEVGQQYNQSNDHLTTFS

NAFEFLTSSDDSNDSSALVSDSNQSNSNHDMDYYDYGANTDAISKDRMLTSVASEHNISMGIPPTLHGSI

TAPPLTSYGNNPNLCDQLDVNNHGLSRCLYESEINNYQSDLSFCTLDHPNEFTTRASDYLYSLSENEGIA

DLFDCGDIVGSL

>E2F-Mb gi|167523471|ref|XP_001746072.1| hypothetical protein [Monosiga brevicollis MX1]

MASSEIPPTDQSVPVPFMPVGPSQGAPPQSLASAALTCAPGPASGVYYDPAAASRARPDLTPRGNSLPHAGAAPLTSPYT

MPHANATPLGYAPPLGTQPMPPHAHLTLTPGRGVNDITPLAHDYSDVYSAMTTPHQPAATPSTTTSRDRAPRSEKSLHLL

TTRFIDLLQNTPGGSLDLKDAAEKLDMRQKRRIYDITNVLEGVGLVEKTNKNVVRWRHDPSSDSSSSNAQTRAVQEEIAS

LDAEIQSLERLTHVMQDRLRNAVDEVEDPKLKALPYRDICKAKGLQDQTHFAIRAERGATMTVPEPQPIDNQRTQYCLYL

RGNAGSIKAFLVVDKGNGSDEEQPSTQPESQATSIVAAPGSTHDTSGPNASDRAAPAPKRAKQADIMYVNPPQSQAPYTF

GVRETDTVDEYFR

>E2F-Dd gi|66810101|ref|XP_638774.1| transcription factor E2F/dimerisation partner (TDP) family protein [Dictyostelium discoideum AX4]

MNTEIIRQNTIIPLTPDITTNNNIVHSNNNNNNNKMTKSKQDDNFDNSNNSNNSNNNKNNKNILPTTRNRNVNSQQKVGN

GNGNGNSLADEAASGYGTGNSLLSLDPLSQIQSMASLQSPTFLNKSSPNPTPNISLPQPTISTTANPTSPPSSAITAATT

GTTNTNTSTTSTTANNNNNNNNNNLSADKKIQNSLQLLRNSTKSNSVTSSSSSSSSSSSSSSSPPSTNNSDNTSPQEPSP

SSSNSSISPSTTPTSSQQLTSPQESNLSSSQGQTTTTTTTTTTTNTTSTTTTRKRRSTSSVNNTTVASQTNTTSTPPPPT

TTTTTTTTTKPKGAPKRQTKRSKSKGDLNSENESTPSSQLSSSSSSTTTTTQSSQPINDDQEDDDDDDDYDNSQASTTSN

GSGGGGGNKKKTKSTVGNRFDNSLVQLTKKFLDLIEKSPNGVLDLKVASEKLEISKRRIYDVTCVLEGVGLIEKCSKNQV

LWKGIGNDVNGQQNSNGQQQHQQQPLDPKHVDNFKKELKKLMEKEASLDNSIKKANKNIHNTLYEPKSSKLMFVTHDDLR

NIETLKGDTVIAIRAPSGTRLQIPDPDEGMEPGQRRYQILLDNETNAPIDVFLLNQTTMQPTDSITNNTNTNTNNNHYNN

NNNNNNNNNNNNNNNNNNNNNNNNNNNNNNNTTMNTTTEEYSYATNAITNALVGITNSNAYLDNNNNNNNNNNNNNNNNN

NNNNNNNNNNSILSPSKQIQQQTNYLQPSQNVSYWEPNSILPSYSPFDSTHNQQSTNSSSSSSSSSSSSSMVAPPNPQQH

ANNYQAFQKVGNNYQAFSEPMFEQSSEFYFESMIDSEGISELYTDDSFLTQSFDDFGNQSIES

>Os02g0537500-Os gi|115446539|ref|NP_001047049.1| Os02g0537500 [Oryza sativa (japonica cultivar-group)]

MAGSGRPPAAQKKILQSLRPPLPFAASSRSPFAAPNDYHRFPAGGAAAAAASGSGGIGAGGAGGGGDIEEGLVIRTPQKR

KAPEESDVAESSDCMITSPGFAVSPMLTPVSGKAVKTSKSKTKNNKAGPQTPTSNVGSPLNPPTPVGTCRYDSSLGLLTK

KFINLLKQAPDGILDLNNAAETLEVQKRRIYDITNVLEGIGLIEKTLKNRIRWKGLDDSGVELDNGLSALQAEVENLSLK

EQALDERISDMREKLRGLTEDENNQRWLYVTEDDIKGLPCFQNETLIAIKAPHGTTLEVPDPDEAGDYLQRRYRIVLRST

MGPIDVYLVSQFDEKFEDLGGGATPSGHANVPKHQPTEVFNTTNAGVGQCSNSVAVDNNIQHSQTIPQDPSASHDFGGMT

RIIPSDIDTDADYWLISEGDVSITDMWKTAPDVQWDESLDTDVFLSEDVRTPSSHNQQPSAVGGPQMQVSDMHKP

>Os12g0158800-Os gi|115487438|ref|NP_001066206.1| Os12g0158800 [Oryza sativa (japonica cultivar-group)]

MAAAGAGSSEVAARVLLQRYQPFAPPPGEYHQFGSGGAAAAGDMTEAVLIRTPLKRKHDREENEAAESNDWMMSPGYTNP

AGSPVPTPLSGKGSKAFAKSKAAKGQKSCPQTPLCASSPGNPVTPVGGCRYDSSLGLLTKKFLNLLKGAPGGIVDLNNAA

ETLEVQKRRIYDITNVLEGIGLIEKKLKNNIRWKGIDDSRPGEVSDDMSILQADIEALSLQEHSVDQQISEMRDKLRGLT

EDENNQKWLYVTEDDIKSLPCFQNQTLIAIKAPHGTTLEVPDPDEVNDYPQRRYRIVLRSTMGPIDVYLVSQFEEMSGME

TPPRTVQPVSMDSLENPRTPLAAEPNKAAESQPNIQDGLLMPSDAPSSSQDIGGMMKIVPSELDTDADYWLLSDAGVSIT

DMWKTAPEVEWEGIEKFNAEDFLEVSTPRQQDKPSSDIMDGDSCIS

>Os04g0416100-Os gi|115458326|ref|NP_001052763.1| Os04g0416100 [Oryza sativa (japonica cultivar-group)]

MSSGGGRPPAAQHIVRSVRQRFVPLPPPLARAPFAAAPGDYHRFAAASRGGEIEEGIVIRRTPLKRKTPCGESEAAESSE

RMMTSPGFTEGVGSPLMTPVSGKTSRTTKSMAKFNKAGPQTPISNAGSPGNPSTPASSRYDNSLGLLTRKFINLLKQTQD

GILDLNDAAKILDVRKRRIYDITNVLEGTGLIEKKLKNRIRWRGSDDSGTNLDSDISCLKTEVENLYIQEQALDRSISEI

REKMEELTEDESNHRWLFVTEDDIKGLPCFQNEALIAIKGPRGTTVEVPDPDEAGDYLQRRYRILLRSTMGPIDIYLVR

>ATE2F1-At gi|18420430|ref|NP_568413.1| E2F1; transcription factor [Arabidopsis thaliana]

MSEEVPQQFPSSKRQLHPSLSSMKPPLVAPGEYHRFDAAETRGGGAVADQVVSDAIVIKSTLKRKTDLVNQIVEVNELNT

GVLQTPVSGKGGKAKKTSRSAKSNKSGTLASGSNAGSPGNNFAQAGTCRYDSSLGLLTKKFINLIKQAEDGILDLNKAAD

TLEVQKRRIYDITNVLEGIGLIEKTLKNRIQWKGLDVSKPGETIESIANLQDEVQNLAAEEARLDDQIRESQERLTSLSE

DENNKRLLFVTENDIKNLPCFQNKTLIAVKAPHGTTLEVPDPDEAGGYQRRYRIILRSTMGPIDVYLVSQFEESFEDIPQ

ADEPSNVPDEPSNVPDVPSNLPSTSGLPENHDVSMPMKEESTERNMETQEVDDTQRVYSDIESHDFVDGIMKIVPPDLDM

GVDYWFRSEVGEVSITDMWPDESGPDWNQMITFDQDHAGPSDNKILEQPQTPSSPTPEESTATRSPTGS

>ATE2F3-At gi|42571075|ref|NP_973611.1| E2F3 (E2F TRANSCRIPTION FACTOR-3) [Arabidopsis thaliana]

MSGVVRSSPGSSQPPPPPPHHPPSSPVPVTSTPVIPPIRRHLAFASTKPPFHPSDDYHRFNPSSLSNNNDRSFVHGCGVV

DREEDAVVVRSPSRKRKATMDMVVAPSNNGFTSSGFTNIPSSPCQTPRKGGRVNIKSKAKGNKSTPQTPISTNAGSPITL

TPSGSCRYDSSLGLLTKKFVNLIKQAKDGMLDLNKAAETLEVQKRRIYDITNVLEGIDLIEKPFKNRILWKGVDACPGDE

DADVSVLQLQAEIENLALEEQALDNQIRQTEERLRDLSENEKNQKWLFVTEEDIKSLPGFQNQTLIAVKAPHGTTLEVPD

PDEAADHPQRRYRIILRSTMGPIDVYLVSEFEGKFEDTNGSGAAPPACLPIASSSGSTGHHDIEALTVDNPETAIVSHDH

PHPQPGDTSDLNYLQEQVGGMLKITPSDVENDESDYWLLSNAEISMTDIWKTDSGIDWDYGIADVSTPPPGMGEIAPTAV

DSTPR

>ATE2F2-At gi|15220994|ref|NP_175222.1| ATE2F2/ATE2FC/E2FC (ARABIDOPSIS HOMOLOG OF E2F C); DNA binding / protein heterodimerization/ transcription factor [Arabidopsis thaliana]

MAATSNSGEDPTLSYHHRSPFRFELLQSISSSDPRYSSLTPSSTNRPFSVSQSLPNSQLSPLISPHWDDSYSQITQKVQK

SRKNHRIQLGSIANMSGGESIDIAKVIVKQESSPQNVKRVYNKSKGGTKLLKAGKRMANGEVQNGGLNGASINCRYDSSL

GLLTKKFVKLIQEAEDGTLDLNYCAVVLEVQKRRIYDITNVLEGIGLIEKTTKNHIRWKGADNLGQKDLGDQISRLKSEV

ESMQSEESRLDDLIRERQEALRSLEEDDYCRRYMFMTEEDITSLPRFQNQTLLAIKAPTASYIEVPDPDEMSFPQQYRMV

IRSRMGPIDVYLLSKYKGDSAETSDKLGNESDQKAPVGVDTPSLKIVTSDTDLKADYWFESDAEVSLTDLWSNFNS

>E2F-Ot gi|55978010|gb|AAV68605.1| transcription factor E2F [Ostreococcus tauri]

MVEKHVLSSKRSLGRFERYARAHCLNSADTSCSSLGVLSAKFMKLLSETENGILDLNHAATSLSAQKRRVYDITNVLEGI

GLVSKLSKSKVALRRVDEDFVETTSGQHKEHKSLSRTVNIESSQTLPLASEDDASLHIETIRSFIRSTGIFISQADIIEQ

HALSSDMLIAVRAPTGAALLLPSPFTQERSPPHYRLFLRSNENSSAGVEVFTLGRRSGTCDAHDVIQILNGFSSLCREAQ

RAVWLLNSCDVLYFDINKDHVPVSFATILEFDNVHHNVTLIRQLPTFFLGVLFCHFLQVGNVFGTYSQGEKF

**Sequences for E2F7/8 family:**

>E2F7-Hs gi|145580626|ref|NP_976328.2| E2F transcription factor 7 [Homo sapiens]

MEVNCLTLKDLISPRQPRLDFAVEDGENAQKENIFVDRSRMAPKTPIKNEPIDLSKQKKFTPERNPITPVKFVDRQQAEP

WTPTANLKMLISAASPDIRDREKKKGLFRPIENKDDAFTDSLQLDVVGDSAVDEFEKQRPSRKQKSLGLLCQKFLARYPS

YPLSTEKTTISLDEVAVSLGVERRRIYDIVNVLESLHLVSRVAKNQYGWHGRHSLPKTLRNLQRLGEEQKYEEQMAYLQQ

KELDLIDYKFGERKKDGDPDSQEQQLLDFSEPDCPSSSANSRKDKSLRIMSQKFVMLFLVSKTKIVTLDVAAKILIEESQ

DAPDHSKFKTKVRRLYDIANVLTSLALIKKVHVTEERGRKPAFKWIGPVDFSSSDEELVDVSASVLPELKRETYGQIQVC

AKQKLARHGSFNTVQASERIQRKVNSEPSSPYREEQGSGGYSLEIGSLAAVYRQKIEDNSQGKAFASKRVVPPSSSLDPV

APFPVLSVDPEYCVNPLAHPVFSVAQTDLQAFSMQNGLNGQVDVSLASAASAVESLKPALLAGQPLVYVPSASLFMLYGS

LQEGPASGSGSERDDRSSEAPATVELSSAPSAQKRLCEERKPQEEDEPATKRQSREYEDGPLSLVMPKKPSDSTDLASPK

TMGNRASIPLKDIHVNGQLPAAEEISGKATANSLVSSEWGNPSRNTDVEKPSKENESTKEPSLLQYLCVQSPAGLNGFNV

LLSGSQTPPTVGPSSGQLPSFSVPCMVLPSPPLGPFPVLYSPAMPGPVSSTLGALPNTGPVNFSLPGLGSIAQLLVGPTA

VVNPKSSTLPSADPQLQSQPSLNLSPVMSRSHSVVQQPESPVYVGHPVSVVKLHQSPVPVTPKSIQRTHRETFFKTPGSL

GDPVLKRRERNQSRNTSSAQRRLEIPSGGAD

>E2F8-Hs gi|38505226|ref|NP_078956.2| E2F family member 8 [Homo sapiens]

MENEKENLFCEPHKRGLMKTPLKESTTANIVLAEIQPDFGPLTTPTKPKEGSQGEPWTPTANLKMLISAVSPEIRNRDQK

RGLFDNRSGLPEAKDCIHEHLSGDEFEKSQPSRKEKSLGLLCHKFLARYPNYPNPAVNNDICLDEVAEELNVERRRIYDI

VNVLESLHMVSRLAKNRYTWHGRHNLNKTLGTLKSIGEENKYAEQIMMIKKKEYEQEFDFIKSYSIEDHIIKSNTGPNGH

PDMCFVELPGVEFRAASVNSRKDKSLRVMSQKFVMLFLVSTPQIVSLEVAAKILIGEDHVEDLDKSKFKTKIRRLYDIAN

VLSSLDLIKKVHVTEERGRKPAFKWTGPEISPNTSGSSPVIHFTPSDLEVRRSSKENCAKNLFSTRGKPNFTRHPSLIKL

VKSIESDRRKINSAPSSPIKTNKAESSQNSAPFPSKMAQLAAICKMQLEEQSSESRQKVKVQLARSGPCKPVAPLDPPVN

AEMELTAPSLIQPLGMVPLIPSPLSSAVPLILPQAPSGPSYAIYLQPTQAHQSVTPPQGLSPTVCTTHSSKATGSKDSTD

ATTEKAANDTSKASASTRPGSLLPAPERQGAKSRTREPAGERGSKRASMLEDSGSKKKFKEDLKGLENVSATLFPSGYLI

PLTQCSSLGAESILSGKENSSALSPNHRIYSSPIAGVIPVTSSELTAVNFPSFHVTPLKLMVSPTSVAAVPVGNSPALAS

SHPVPIQNPSSAIVNFTLQHLGLISPNVQLSASPGSGIVPVSPRIESVNVAPENAGTQQGRATNYDSPVPGQSQPNGQSV

AVTGAQQPVPVTPKGSQLVAESFFRTPGGPTKPTSSSCMDFEGANKTSLGTLFVPQRKLEVSTEDVH

>E2F7-Cf gi|73978133|ref|XP_539692.2| PREDICTED: similar to E2F transcription factor 7 [Canis familiaris]

MQQHLTEAVAAARPVLKAVKIQFWFHVMAAVATPMEAQWHHRMEVNCLTLKDLINPRQSRLDFAIEDGDNAQKENIFVDR

SRMAPKTPIKNEPIDLSKQKIFTPERNPITPVKLVDQRQQTEPWTPTANLKMLISAASPDIRDREKKKGLFRPIENKDDA

FTDSLQLDVVGDGSVDEFEKQRPSRKQKSLGLLCQKFLARYPSYPLSSEKTTISLDEVAVSLGVERRRIYDIVNVLESLH

LVSRVAKNQYGWHGRHSLPKTLRSLQRLGEEQKYEEQMAHLQQKELELMDYKLGERKKDGYPDSQDQQLLDFSEPDYPSS

SANSRKDKSLRIMSQKFVMLFLVSKTKIITLDVAAKILIEESQDTPDHSKFKTKVRRLYDIANVLTSLALIKKVHVTEER

GRKPAFKWIGPVDFSSSDEELVDVSASVLPELKRETYGQIQVCAKQRLARHGSFNTVQASERIQRKVNSEPSSPYREEQG

SGYSLEIGSLAAVYQQKIEDGSQGKAVATSKRAMPPSGSLDPPAPLPGLSVDSEYCAHPLAHQVFSVAQLDLQAFPTQNS

LHGQVGVSAASAASDVESLKPALLASQPLVYVPSPSLLMLYGRLQEGPAPTSGSGVPADRPPTPSVQKRLGEERKPQAEE

PAPKRQSRDYEDSPLALVMPKKPSEATDLTSAKTLSNGRSGAPEDIHMEAQASAAEEASGKATANCLTSSEWGNPSRDTE

MEKSSKENESTKEPSLLQYLYVQSPAGLNGFNVLLPGSRNPQAGGAPSGQLPPLGIPCMVLPSPTLGPFPVLYSPTVPRP

VSSAAGALPGAGPVHFGVPSLGSTAHLLIGPTNVVNPKSSTLPSADPQLQRPRSLGLSPEVPGPHGIVQPGSPGCTAHPG

SAVKPQQCPGPATPATPVTPRSERRTQRETFFKTPGSLGEPVRRRRGGSAARHSRSAQRRLHIPGPPGPPSTQAD

>E2F8-Cf gi|73988917|ref|XP_534087.2| PREDICTED: similar to E2F family member 8 [Canis familiaris]

MENEKENLFFEPHKRGLMKTPLKESTAANIVLPDIQPDLGPLTTPTKPKEISQGEPWTPTANLKMLISAVSPEIRNRDQK

RGLFDSRNGLPEAKDCLHEHLSGDEYEKSQPSRKEKSLGLLCHKFLARYPNYPNPAVNNDICLDEVAEELNVERRRIYDI

VNVLESLHMVSRLAKNRYTWHGRHNLNKTLGTLKSVGEENKYAEQIMMIKKKEYEQEFDFSKTYSIEDHIIKSNTGQNGH

PDMCFVELPGVEFRAASVNSRKDKSLRVMSQKFVMLFLVSTPQIVSLEIAAKILIGEDHVEDLDRSKFKTKIRRLYDIAN

VLSSLNLIKKVHVTEERGRKPAFKWTGPEISPSPSGRGPVLPLPCSDLEAKHPSKDNCAKNLFSTRGKPNFTRHPSLIKL

VKSIESDRRKINSAPSSPIKTNKAESSQNSAPFPSKMAQLAAICKMQLEEQSSEPRKKVKVQLARSGHCKPVAPLDTPAN

AELELTAPSLIQPLGVVPLLPSPLSPAVPVILPQAPSGPSYAIYLQPAQAQTMTPPQGLSPTVCPTPSSKATRSKDATDA

TTEKAASDAAKASASTRPGSLLPVPERPGAKTRDKEPAGERGSKRASTLEDGGSKKKFKEDLKGLENVSATLFPSGYLIP

LTQCSSLGAESILSSKENSSTLSPNHRIYSSPITGVIPVTSSELTAVNFPSFHVTPLKLMVSPTSVAAVPVGNSPALAAS

HPVPGQTPSSAIVNFTLQHLGLLSPSMQVSASPGAVPVSPRLEGISVVPESAGTQQGRATRYDSPVPGQNQPNGQSVAVT

GSQQPVPMTPKGSQSVAESFFRTPGGPVKPTGPSCMDFDGAKKTSVGALFVPQRKLEVSTEDVH

>E2F7-Bt gi|194666654|ref|XP_604488.4| PREDICTED: similar to Transcription factor E2F7 (E2F-7) [Bos taurus]

MTRMNRIALKTEEKEGQRIFLTEQPAGLEACEMGLLDVPSQWTVKLNPGCKIFGKPRAAYTESALIIQQLLASEGEEGIF

VAGPFCLEMPGSVCYGYLGESKKGQECGISGQGIHELDGQPVQDFLCFTDGLCGRTWCVRAQGELSVAVTSTRRSVCSSP

GPRFLLQPEKGVERRRIYDIVNVLESLHLVSRVAKNQYSWHGRHSLPKTLRNLQRLGEKQKYEEQMAHLQQKELNPIDHK

SGERRRDGCPDSQDPQLLDFPEPDCPSSSANSRKDKSLKIMSQKFVMLFLVSKTKIVTLDVAAKILIEESQDIPDHSKFK

TGSIATLWEKWLDKIQFYQDWEEYCIWSESLMIQAHNQSPWLILALNSSGFPPSKVRRLYDIANVLTSLMLIKKVHVTED

RGRKPAFKWIGPVDFSSTEKWKSSWAFQFIKSVLQLKCASLELKKDSTDEQGRHDYLNNDDDLVDVSTPVLPELKKEIYG

HVQFCAKQKLARHSSFNSEQASERTQRKVNSEPSSPYRQKQGVYSLEIGSLAAVSRQKMEDNSETVAFASQNMMPLPSSL

DPAAPLPSPSVDSEYRVSPLCHQALSAAQTDLKALPAQNGLNGQGGVSLASMALDVEHQPQPLAAAQPLLYVPPAPLFML

CGGLQEGLSPGSGSGSGSVGGGSEVTAAEQPPMPSGQKRLSKERRLQEEEEEPATKRQCRDHEDGPLSLVMPKKPSDSAD

IASPKTSENRASAPHEDTHMNGQLSAAKAVSGKATTNGFVSSEWGNPCSNTEIEKPSEENESTKGPSPLQYLYVQPPAGL

NGLSVLLPSSQSPHAVGLPVGPLPSLSIQYMVLPSPALSGFPVLCSPTMPGPVSSAPSPLPNVGPVNFGLPGLGSTAHLL

IGPAAMVNPKSSTLPSTDPQLQGPCSLHLSPVMSRSHGSVQPGSPAYGSLPAATVKLQQSPVPVTPKSIRCTHQETFFKT

PGSLGDPVLRRKERNQSRSSSSAQRRLEISSGGTD

>E2F8-Bt gi|194679774|ref|XP_001254261.2| PREDICTED: similar to Transcription factor E2F8 (E2F-8) [Bos taurus]

MENEKENLFFEPHKRGLMKTPLKESTAANIVLADIQPDFGPLTTPTKPKEISQGEPWTPTANLKMLISAVSPEIRNRDQK

RGLFDNRNGLSDVKDCLHEHFSGDEYEKSQPSRKEKSLGLLCHKFLARYPNYPNPAVNNDICLDEVAEELNVERRRIYDI

VNVLESLHMVSRLAKNRYTWHGRHNLNQILETLKSVGEENKYAEQIMMIKKKEYEQEFEVSKSYNTEDPIIKSNTGQNGH

PDMCCAERPGVELRAASVNSRKDKSLKVMSQKFVTLFLVSTPQIVSLEIAAKILTWEDHVEDLDRSKFKTKIRRLYDIAN

VLSSLDLIKKVHVTEERGRKPAFKWTGPEISPNPSGLSPVLPCAASDLEARQSSKENCAKNLFSTRGKPNFTRHPSLIKL

VKSIESDRRKINSAPSSPIKTHKAESTQNSVPFRSKMAQLAAICKMQLEEQSSEPRKNVTVQLAGSGHCKSVAPLDTPAN

AEPEMMAPSLIQPLGVVPLLPSPLSPAVPVILPQTPSGTSYAIYLQPAQAQTITPPPGLSPTVCPTTSSNAMISEDSTDA

TGENADSDAPKSSVSTRPGSLLPGPERQGAKNREREPAREKGSKRASMLEDSGSKKKFKEDQKAPENVSTTLFPSGYLIP

LTQCSTLGAESILSSNENSGTLSPNHSIYSSPIAGVIPVTSSELTAVNFPSFQVTPLKLMVSPTSMAAVPVGNSPALSSS

HPLPIQNPSSAIVNFTLQHLGLISPGVQVSTSPGPGTIAVSPRIEAVSVTPENAGAEQGRATKCDASILSQNQTNGQSFA

GTGAQQPVPVTPKGSQPVAESFFRTPGGPTKPTGSPCTDFDGANYTSVGTLLVPQRKLEVSVEDVH

>E2F7-Mm gi|40254337|ref|NP_848724.2| E2F transcription factor 7 [Mus musculus]

MEVNCLTLKDLISPRQTRLDFAIEDAENAQKENIFVDRSRMTPKTPMKNEPIDLSKQRIFTPDRNPITPVKPVDRQPQVE

PWTPTANLKMLISAASPDIRDREKKKELFRPIENKEDAFVNSLQLDVAGDGAVDEYEKQRPSRKQKSLGLLCQKFLARYP

SYPLSTEKTTISLDEVAVSLGVERRRIYDIVNVLESLHLVSRVAKNQYGWHGRHSLPKTLRTLQRLGEEQKYEEQMACLQ

QKELDLMGYRFGERRKDGSPDPRDPHLLDFSEADYPSSSANSRKDKSLRIMSQKFVMLFLVSKTKIVTLDVAAKILIEES

QDTPDHSKFKTKVRRLYDIANVLTSLALIKKVHVTEERGRKPAFKWIGPVDFSSIDEELLDVSASILPELKKEAYGQIRV

CAKERLVRYGSFNTVHTSEKIQRKVSSEPSSPQGERQGSAYSLEIGSLAAIYRQKVEDNSQEEAFVSNTAVPPASILDPA

LSMDSEYCVKPLAQPVFSVAQTDLPAFSAQNGPSGQVGVPVPSAASDTENLKPALLAGQPLVYVPSTQLFMLYGSVQEGL

SPESRSEEDGGGSDVPADLSVTPSAQKRLCEERDPQEEEDEPAMKRQSQEFEDSPLSLVMPKKPSSSTDLACPVTMGNGS

SPPLEDACVKGQLPAAEEVTGKAAPNCYVASECGNPARNPDTEKPSNENEITKDPSLMQYLYVQSPAGLNGFNMVLPGTQ

TPHTVAPSPAQLPSFGVPCMFLQSPGLGPFPVLYSPAIPGPISSAPGTHPNPGPMNFGLSTLASASHLLISPAAMVNPKP

STLPCTDPQLRCQPSLNLNPVMPGSHGVIHPESPCYVRHPVSMVKAEQSPAPATPKSIQRRHRETFFKTPGSLGDPVFRR

KERNQSRNTSSAQRRLEISSSGPD

>E2F8-Mm gi|67972650|ref|NP_001013386.2| E2F transcription factor 8 [Mus musculus]

MENQKENLFSEPHKRGLMKSPLHPSSKANMVLAEIQPDLGPLTTPTKPKEVSQGEPWTPTANLKMLISAVSPEIRSRDQK

RGLSDNRSALPEARDCLHEHLSGDEFEKSQPSRKEKSLGLLCHKFLARYPKYPNPAVNNDICLDEVAEELNVERRRIYDI

VNVLESLHMVSRLAKNRYTWHGRHNLTKTLGTLKSVGEENKYAEQIMMIKRKEYEQEFDFIKSCGIEDHVIKSHTGQNGH

SDMCFVELPGVEFRAASVNSRKDKSLRVMSQKFVMLFLVSTPQIVSLEIAAKILIGEDHVEDLDKSKYKTKIRRLYDIAN

VLSSLDLIKKVHVTEERGRKPAFKWTGPEISPNNSGSSPIMPLPASLEAEQSAKENCAKNLFSTRGKPSFTRHPSLIKLV

KSIENDRRKISSAPSSPVKSNKAESSQNSPPVPNKMAQLAAICKMQLEEQSSEPRKKVKVNLARSGHYKPLAPLDPTVNT

ELELLTPSLIQPLGVVPLIPSPLSSAVPVILPQAPSGPSYAIYLQPAQAQMLTPPPGLSPTVCPTQPSNATGSKDPTDAP

AEKTATDAATTGSLQPAPERHGAKHRSKETTGDRGTKRMITAEDSGPSSVKKPKEDLKALENVPTPTPLFPSGYLIPLTQ

CSSLGPDSVLSNTENSGTPSPNHRIYGSPIAGVIPVASSELTAVNFPPFHVTPLKLMVSPTSMAAVPVGNSPALNSGHPA

PAQNPSSAIVNFTLQHLGLISPGVQMSASPGPGAGTVPVSPRVEADNLSSRQRRATNHDSPVLGQSQLNGQPVAGTGAQQ

PVPVTPKGSQLVAENFFRTPGGPTKPTSSPYTDFDGANKTSFGTLFVPQRKLEVSTEDIH

>E2F7-Rn NP_001101562.1 (ENSRNOT00000031986) E2F transcription factor 7 [Rattus norvegicus]

MEVNCLTLKDLISPRQTRLDFAVEDAETAQKENIFVDRSRMTPKTPMKNEPIDLSKQRIFTPERSPITPVKLVDRQPQVE

PWTPTANLKMLISAASPDIRDREKKKELFRPIENKGDAFVNSLQLDVVGDSAVDDYEKRRPSRKQKSLGLLCQKFLARYP

SYPLSTEKTTISLDEVAVSLGVERRRIYDIVNVLESLHLVSRVAKNQYGWHGRHSLPKTLRTLQRLGEEQKYEEQMACLQ

QKELDLMEYRFGERRKDGSPDPRDQHLLDFSESDYPSSSANSRKDKSLRIMSQKFVMLFLVSKTKIVTLDVAAKILIEES

QDTPDHSKFKTKVRRLYDIANVLTSLALIKKVHVTEERGRKPAFKWIGPVDFSSIDEELLDVSASVLPELKKETYGQIRV

CAKERLARYGSFNTVQTSEKIQRKVNSEPSSPQGGKQGPAYSLEIGSLAAIYRQKVEDSSQGEAFVNKRAAPPASVLDPT

LPVDSEYCVKPLAQPVFSVAQTDLQAFSAQNGLNGQVGVPVPSAASDAETLKSALLASQPLVYVPSTSLFMLYGSVQEAL

SPESRSEEDGSGSDVPADLSLAPTAQKRLCEERNPLEDDEPAVKRQSREFEDSPLSLVMPKKPSNSTDLAFPVTTGNGRA

TPLEDACVKGQLPAAEDASGRAVPNGFIASECGNPSRNPDTEKSSNDNEITKDPSLLQYLYVQSPAGLNGFNMLLPGGQT

PHAVAPSSAAMPSFGVPCMFLPSPGLGPFPVLYSPAIPGPISSAPGTLPNTGPMNFGLSTLASASHLLISPAAMVNPKSS

TLPSADPQLRCQPPLNPNPVMPGSHGVIHPESPGYMRHPVSMVKAEQSPAPATPKSIQRRHRETFFKTPGSLGDPAFRRE

RNQSRNTSSAQRRLEISSSGPD

>E2F8-Rn gi|109462000|ref|XP_001080259.1| PREDICTED: similar to E2f family member 8 isoform 1 [Rattus norvegicus]

MENQKENLFSEPHKRGLVKSPLQESSKANVVLAEIQPDLGPLTTPTKPKEVSQGEPWTPTANLKMLISAVSPEIRSRDQK

RGLSDNRSGLPEARDCLHEHLSGDEFEKSQPSRKEKSLGLLCHKFLARYPKYPNPAVNNDICLDEVAEELNVERRRIYDI

VNVLESLHMVSRLAKNRYTWHGRHNLTKTLGTLKSVGEENKYAEQIMMIKRKEHEQEFDFIKSCGLEDHVIKGDHVIKST

AGQNGHSDMCFVELPGVEFRAASANSRKDKSLRVMSQKFVMLFLVSTPQIVSLEIAAKILIGEDHVEDLDKSKFKTKIRR

LYDIANVLSSLDLIKKVHVTEERGRKPAFKWTGPEISPNNSGSSPVMPLTASLEAEQSAKENCAKNLFSTRGKPSFTRHP

SLIKLVKSIENDRRKISSAPSSPVKSSKAESSQNSPPVPNKMAQLAAICKMQLEEQSSEPRKRVKVNLTRSGHYKPLAPL

DPAVNTELELLAPSLIQPLGMVPLIPSPLSSAVPVILPQAPSGPSYAIYLQPAQAQMLTPPHGLSPTVCPTQSSNATGSK

DPTDAPTEKTATDATKSSASCRPGSLQPAPERQGAKNRSKETTGDRGTKRTGALEDGGPGPIKKPKEDLKALENVPTPTT

LFPSGYLIPLTQCPSLGPDPMLSNTENSGTLSPNHRIYGSPIAGVIPVASSELTAVNFPPFHVTPLKLMVSPTSMAAVPV

GNSPALSSSHPAPTQNPSSAIVNFTLQHLGLISPGVQMSASPGPGAGTVPLSPRVEAASVIPDNLSSRQGRATIHDSPVL

GQSQLNGQPVAGTGAQQPVPVTPKGSQLVAESFFRTPGGPTKPTSSSFMDFDGANKTSFGTLFVPQRKLEVSTEDVH

>E2F7-Gg gi|118082443|ref|XP_416110.2| PREDICTED: similar to E2F transcription factor 7 [Gallus gallus]

MSGCGRLAVEWMCASRSDAAAAPLRYLHTDGRLRWGNRGFSSGPLCSPAGTSRGEQKCDGKGTSCTADEVLIRCQMGTCK

NENILDRSRMAPKTPIKNDPVDLSKQKGCTPDRNPMTPVKLIDKPQADPWTPTANLKMLISAASPDMRDREKKKELFRPI

ENSEQNDIPDSLQYDMVDDGTVDEFEKQRPSRKQKSLGLLCQKFLARYPSYPLSTEKTTISLDEVASILGVGRRRIYDIV

NVLESLHLVSRVAKNQYCWHGRHHLSQTLKTLQEAGELQYGELVTSQCKEQDTEYKSGEQKNETVPDSQDRPLLDFAEPD

CTSASANSRKDKSLRIMSQKFVMLFLVSKTKIVTLDIAAKILIEENQDTVDYSKFKTKVRRLYDIANVLTSLCLIKKVHV

TEERGRKPAFKWIGPVDFPGKTDEPRGHCPTSGPPAEAQRGACAPYQICATGKQRFTRHASFNGAQPCEGTKRKVSSEPN

SPNRERQACIVNSDEYCSKMINLATVCRQKIEEDTRSKACATEAILNAVRNPSVTSPLSLLPVPGDSDFCANPLSQAVFP

VVQADMPSLLLPKGVSSQALHPSSAAASKTENGKPPLLPSQPFLCFPSSSFFMLCGGLQENLSRESGSTEKAVPPEAQGA

VPPANCQKRSSHRCTSPSAPTDDEEPAAKKQTVEQSDVPISLVVPKKPVESPKAGTAPGSGCASAVRLEAFHLDLSSPAA

PETADKKSTKPLDSQEQEKWSQNKDLDEPSPGKDHISKGNANQPFVPQYLYVQPAAGLSSFNFLFSANQAPSAIGLAASQ

LPSLSVPCVMVPSAALASFPLICSPAIPSPLSPVPDGSSAAASMNISMPGLASTTPVFIGTTAVVTPNASPSPSVDPQQT

AHPAAHPRPVLARSCSAVKLDSPVCVGHPVTLLKLQQPSSTPVTPKSVRPARHEAFFKTPGSLGDPGAWKKGEGNQTRSA

SSVQRRLEISSTSTD

>E2F8-Gg gi|118091079|ref|XP_420910.2| PREDICTED: similar to E2F family member 8 [Gallus gallus]

MPEQEEERLATEIKTQPPPPRIPSAYTGHTTSTKKRNPKQQHDLGEPFAQQPLRDTERSRKGRPQAVGNLRQSSGTGRAA

TGKASERSQPLCAARHNRPRPRPPPATWSFKDQWRAGAAAAQGGEETRAGREALPGRVGVRSPRSAPPRGGSVPGRPARP

RYSAALRPQRALRGRPPRVRALSDSGRCPARGGRTIPQAPRGPLRTPLKQAGGSQRVLAELQPGCQPLATPPKPGEALPA

EPWTPTANLKVLISAVSPEIRSREQSRGLQGARCCQEHLSGDEYERSQPSRKEKSLGLLCHKFLARYPDYPSAVESNYIC

LDEVAEELNVERRRIYDIVNVLESLHMVSRLAKNRYIWHGRHNLAETLQTLKKVGEENKYTQQIQMIKKREYEHEFDLDG

KRNEEVARSFISSEHSEMCFVELPGIEFRAASVNSRKDKSLRVMSQKFVMLFLVSTPQIVSLEVAAKILIGEDQLEDLDK

SKFKTKIRRLYDIANVLSSLELIKKVHVTEERGRKPAFKWTGPEVPPNTQGTYMCHFLFSHISESTTSKEQCSKNLFPSR

VKQSFTRHPSLIKLVKSIESDRRKIQSAPTSPVKISTTGTDENVPVYTSKMPQLAAVPQYQLEEPPKRQKDMKKNLSGSA

LEHSLPWPEAAPKANTHTVAPPPKSLLTQPLGALPPSHRSASPVILPQPHSGASYAIYLHPSQAHTVTAYSPSFMLQPLP

CANVTGIKSITAKALSEMTTKEGDNCTAAADSGKAMAAKERPVMESESSSKRCLKRSQALQENNLIKKHRSDEESLDTAV

GEPTKQERPPSISSQTNHQMDNFQEEKQNRSETLDQSMAGCSEQYKRECAPEDEDRTKTKQDIPVAFAIPAHETFFPSGY

LIPLTQCTHHSNKAGLPSREKTGTCSLHHTATHSSPTAGVIPMIASELKAVNIPAFHITSLNIMLSPTSIAAAPVLSNSH

LNSSSTSSVPNPSSSLLNFTLQHVGLIPAGVQVPANPVLQHIPIPSKPESINHSSENMTLQEEKPTVPKEPPEPQSVTES

FFRTPGGPNSAPLLSANSGGTDTISQGTSHIPQRKLEVSEN

>E2F7-Xt Transcription factor E2F7, ENSXETT00000031323, [Xenopus tropicalis]

MEVTSCLTLKDLISTKKNKSDPVADGRSAQKENMFDRFKISPRLPLRSEPIDLSKQKSFTPERIPVTPVKVAERPQADPW

TPTANLKMLISAASPEIRDREKKKELFRPIENNGIEETDTDLQLMDSVDDIDDLEKRPSRKQKSLGLLCQKFLARYPSYP

ISTEKMTISLDEAASSLGVERRRIYDIVNVLESLHLVSRVAKNQYCWHGQHNLNETLRNLQHIGEKQNYRAQIACFNLRD

MGMEYKCDEQEKGCHIDHLNTPLIELSEADCPSVSSSSRKDKSLRIMSQKFVMLFLVSTTKIITLEIAAKILIEESQDAA

DHSKFKTKVRRLYDIANVLTSLGLIKKVHVTDERGRKPAFKWIGPVDFTAEDQKMEVTTTIPSPDSKKDACNLSPASDRV

KQRLFRHSSFNIVQSFSAVKRKVCSHPCSPQKPQGVESSDSYASKMAHLATICKPKAEEDSKNGNIENSSLPFSVVVPMP

VDSDYRVKPVVHQVPLVSHKTVCEPLGIMPPSQSNEDCTNHGFVPNQPYMYLPSNSVFMLCGNLSEGKATDHLAMSLYPV

PGSDSPTLEETTMSKQERPTKRQLNDKDDAPLSLVLPKKSRVDNTQSLQKPICKTTTPEQLQHVSREEEYNTEPVTKHSN

VGETTEEVGSRILPHENVHLHPAVPPQFLYVPTTQGLNSFNFLLPANHSAGLSQSQLASLNVPYVMVPSSALTAFPFICS

PAVSSGASGSTLNGRMNFSQAGTSSPTRLIIGAPQMAVPQPPEPAVDQTKNLSPLSVSPVSAKCASSKADSHDSLSQSIH

TAKLHKSPTPSTPKSIRPLHKDAFFKTPGSLDVSSSRKPQRTQTRTSSSAQRKLDIDSSAGN

>E2F8-Xt (ENSXETG00000004436) [Xenopus tropicalis]

IQIAAANLKTPTKPQERNNADPWTPTANLKMLISAASPEIRNREREILEEQFSGDELEKTLPSRKEKSLGLLCHKFLARY

PSYPNPAVNNSICLDEVAGELSVERRRIYDIVNVLESLHMVSRLAKNKYIWHGRLNLSKTFDALKKVGEENRYGEQIQLL

RKREQEECDSQNSPNAETQKPLAKQPEVGFVELPGLEFRAASVNSRKEKSLRVMSQRFVMLFLVSDPQIVSLEVAAKILI

GEDQLEDLDKSKFKTKIRRLYDIANVLTSLNLIKKVHVTEEKGRKPAFQWTCPELCTDDQGSKNRSSPAALTPVAIDLSS

PKENCAKNLFASGGKTFTRHPSLIKLAKSIENDRRKINSAPSSPIKSGDGSSSAASKMAQLAAICKQQLQQSRDQTKVKL

KVSACKAKSTVKQPGGSDKNQTPTYCRAIPLLHPHPSAAPPYTVIVQPPQEQTLSRQSPPALAHSGGRHEGDGTSHSEDH

SAQERHPKRLPESDRGCTSKRMKSSAVDDVTETLYPSGYLIPIHLAPVAPEPSKENTGPSSENKLFTSPIPGVFPLKLMF

SPGPVTAVPVMSRGGQHVGGGSGSASRSPSPGMFTFALQNRELISAGLPQGATVSPRNGRGQEELSAASVLNCKHVSPVP

YHGQPFTVFALQQSAVPVTPKGYHSLQETFFRTPGGMGCSPPESARKLDVGTDD

>E2F7-Dr gi|169234759|ref|NP_001038612.2| hypothetical protein LOC567941 [Danio rerio]

MQEVKCLTLKDLLGVRTLVNKTGSDDAASMNDHKENICMDRRKMTPLKSESLTAALNGHGKISSPEITHITPIKLTEKAH

PDPWTPTANLKMLINAASPDIRDREMKKTLFKPIENKGKIAEEEEEEELDDSCQYEALDESERRPSRKQKSLGLLCQKFL

ALYPDYPESSESINISLDEVATCLGVERRRIYDIVNVLESLMLVSRKAKNMYVWHGRSRLPQTLQGLLQAGRDQHYDLLM

DQREGNGLHAVQHVQNAHAASSRRKDKSLRIMSQKFVMLFLVSKTQTVTLDMAAKILIEEGQEESYDSKYKTKVRRLYDI

ANVLTSLNLIKKIHMREEKTRKPVFKWIGPGNFQSSSNSDDLRGQISNSGTERREKMARHSSFQVITAPPVNQRLISSAP

STPHRYSTDEPVDYSRKSGNNSAVCRLQFGDGVHPSVSPAVPSALASLAMPLQADLMPVPASFSHPLAILPQTPLLMLYS

GNISDGASSLRKRERSEEDDHQTTKCRRRSASIESDTVESESLSSSTRRSPVCSPEGSPWDEASFGGLHEDDVAASSSKD

ALLSPHYLYVPNTAGLNSFNFLLPAGHAQGGVPAVAMPYFVVQSPLIAGAMPTSSTEGAAGFSVPTVLSPAQFVMAGGAY

GVTEILQSPEHHGNVPATTSSPRAEESPKPAQTQTPVTPKEASLGSKSFFETPGAFGSLVNQSAARKRGSAQRRLDIGHT

AAN

>E2F8-Dr gi|189521060|ref|XP_694311.3| PREDICTED: im:7149356 [Danio rerio]

MGPLTTPTKVLDAPSSDPWTPTSNLKMLISAASPEIRNREKERAVDSSESENSQETEQGEEVEKLHISRKDKSLGLLCYK

FLARYPNYPNPALNNGISLDDVAAELHVERRRIYDIMNVLESLNMVSRLAKNRYTWHGRVKLAQTLAVLKRAGKENRYEQ

LMQQIRQRSQEREEREFDLDGEEKENEEMSSFEVDGDSGLADLPGADSKAASANSRKDKSLRVMSQKFVMLFLVSSPPVV

SLDVAAKILIGEDHVVDQDKNKFKTKIRRLYDIANVLSSLELIKKVHVTEDKGRKPAFKWTGPEDIPSPKDLEISTTSSA

PKPLESRSSVENCAKNLFSSPGTKRGFTRHHSLVKLVKSIQDDRRKINSAPSSPIKMTGDSADSDFYTTKMAHLAAICKK

HLDEQSADGRPNNAVTDSSQSSKQPESTSASNHGPPGMQIPVLPAGAISYLPTKCSPIIPLLIPQHQTGGPYAVYMHPTS

LRPQPTSLAVRSMTFESPVGANAKTSPATLTSNNQTNQSSSYGKEQTSPVNLKRASGEKSSVGSPSKMQRTEPKSVSPKL

CEILQARLKARRGALTSNRPSARALHLEFSKPSESQPTVQTGTASLEHSLETFLEKEEKSQTSDNEAGLTPVRQPHSQPQ

KLSAPFQDMVLPSGPIHTETLIPAGYLIPISQQSIVNFREPQCSNESSKASTPTYNIYHTPTAGSRPAFPQEVTPTRLPL

HRIPPISPFPSHGHRLHSPSPAILNFTLQNLGLIPGSVTPNPHTPEQSSSLQSPHPGLPHQGMIFVKPMSPARALQQTSI

HGQPVTLISIPQALVTTPKGGQAFQQSFFHTPVSFPTVNTTAPKKIYIPQRKLDVSPEEI

>E2F7-Tn ENSTNIT00000007956 [Tetraodon nigroviridis]

MEVECLALKDLTSPRNSFMVQLEEEDGGEHKENICRERRRSTPMKSEAAAMLANRRGAAPDADHATPVKHQPLPEPWTPT

ANLKMLISVASPDIREREMKKVLFALADNDADKDKSADAAAEGSLGLLCQKFLALYPDYPPPHNPIWIPLDEVAASLGVE

RRRIYDIVNVLESLTIVGRIAKNCYTWYGRQRLEATLEELQQRGRKQGYHLHMEPGVEAPQGGLGREDEGAEGDHANGFV

AASNRKDKSLRIMSQKFVMLFLVSKTQTVTLDTAAKVLIEDGQDSSSHSKYKTKVRRLYDIANVLTSLNLIKKVHVREER

SRKPAFKWLGSVDFNRSAAAADKDLSKAKMTRHASFNAAPAAVSVPRQVSSAPCSPGPHQPVDYSRTAGHGSARGQEFGN

GADVCPSAHLLVPPLRGERALLAPSSSAPRCLAYLPALSRPPVVMLYGPDQAAEGQRSPGVEFALVKKRHMSGSEECDEV

GKRAETHKSRDARVYSRHRSLQKEAEGAECSSEGPGELPQTSHYLLIPNNAGLNGVNFLIPAGQPPASIALSPTLAVPYV

LVPSTALSHYPLVAGRLPPQVSDAQASHPSLSFSLPNVMPHPHYMVGAAPYGL

>E2F8-Tn ENSTNIT00000000955 [Tetraodon nigroviridis]

MGPLTTPKKGREVGSVDPWTPTSNLKMLISAASPDIRNREKELGLDASGRGCVDSAQDPEPGEDSERWISRKEKSLGLLC

RKFLARYPDYPNPARNNDICLDDVAIELNVERRRIYDIMNVLESLHIVSRSAKNRYAWHGRTKLAETLAILKEVGEEHRY

SQQMQQIRQRLREQGQKELFFVELPGVEFKAASVNSRKDKSLRVMSQKFVMLFLVSNPRVVSLDVAAKILIGEDHGADRD

KNKFKTKVRRLYDIANVLRSLKLIEKVHVTEERGRKPAFEWVGPQDFPPVQGCAPEGPPRNRAVLEPRAIKDNCAKQLFS

SPGTKRSFTRHPSLIKLAKSIQEDRRKINSAPSSPAKSSLGGSGETSSVDFPNKMAQLAAICKIELDQTGRAAQKSAAVN

PGRTSASEEPPPPPLLSSAAAHPAPRAPLAVLPAGSVAYIPAHCPSLIPVLLPQQQGGAPYAIYLPSPPAKPHPLARPQP

TSFAVRSMTFEDRAGQSPGARAEARRADLSPLVQKRVGPEPACESSPPKARRSGSAKFNGPQVAPGPQFAHLCSPFTLVP

AGYYIPIPHQVVSDKEIPDLAKDTKASTPTYNIYQTPTAGKDAAAARTLGSRPSPAQEMTPTSTRLLRPASAAASPHGAH

KAQRLHSPSPAILNFTLQNLGLISGSGPGGGYAASQTPERLGALASPLSLQRRGVVFVKPVSPGPLQQSGQVALISVQQP

LMTTPKGAALPQPSFFHTPVPLSPLAAVVPAGAHVATKTVYIPQRKLHVSTDE

>E2F7/8-Ci gi|198432739|ref|XP_002131779.1| PREDICTED: similar to Transcription factor E2F8 (E2F-8) [Ciona intestinalis]

MEALSPEKEGKGLDAAIKKIQSALMDGIDKEETDDWSDEMDELDSTNCPLPFSIQEENVDDSGLQCEEIVISASFNSENG

HESQSDLNIIATVTPTKSNTHTKVQTAALTPTANLKVLLHAASPEIRNYERRKKLFSRPCSTEQDQPQQAVETADCFSEQ

TMEIAEESEAEDLNENQAQDAPRRICKTFKRKEKSLGLLCRRFLRLFPENPKESISICLDDAAAKLCVGRRRIYDIINVL

ESIKVVTRLAKNNYTWRGRKGLSQTLCALRKEAEESGSADSIRASINGYLVYKVNIATQSVSSTPPERDTSVKRRDKSLG

ILSQKFVTLFLVQPNQLVSLDMAAKVLITDRNPQDNKYKTKVRRLYDIANILTSLRLITKVQNHGRKPAFRWIGPKAICT

STSAVSPKHRQIIVMSPVSNLWSSPKMQVIMQDKDEGDGGLFDLCQVVEQERKDLCLETPIKGGGFTLKTPLKGDQYRIV

TPVKHTSQKLAMSSSNDDSGVNLDSSFNSDEIFRQELESLRQRFPSPMSRLLSACNVEEALTEHKEKKRKLALKSESSSH

SITEPVTPTKVSLSYLKQQQIAISKTVDSVTNRSQINLRKPSTLESHALKSSTKVCQTVQHNMGVSKSLCSDQALRNIKC

LVPVSKENFGMQRNSGSLVQCIKVLPMKTQPLKIVMNPSNKSQEKSPEMSRKSSSKMTTIRVIAPSSTQERPTNNFVKLD

LSSTNFKTVTPKTKLPNSNERTGFCRPVLCNVTSSINNRAKPAQKISKVVTKLVTRLEETAAKKANISHASTSFGLSTMP

QVRFTDSKLPSSGLVALSKKISASLEVPPQPKKMRVSSRKLRMTDENEDPEVKHLLGWKSPPHPIKPIPKQLCLDANQSK

ILQENNITLAPTVKTTEIPSAENCSTNLNLSFGKMQSEDTLLQPISINNRIEQSTYIQLEHSIDSFSSLVSSKAFQISNY

NTPKAESSTVCILEAYQS

>E2F7/8-Mb gi|167517423|ref|XP_001743052.1| hypothetical protein [Monosiga brevicollis MX1]

MTQPTAMSTGCPVSPKRTSYGLELLSRLASPSLRALKAAEQQQHPEQPEPHLSPEPASPTTARRSRRLATPSTSHASSPQ

PASNTPTTPGPASSSTPTRTQIRTATSSRTSARALDLGQAAVDQLPRARGPRSSSQASHQSGAKTKLLEQAEANFTLNIP

SKPIRRMDKTLGRIALGFIEFCKDRQDPEVNLSDAAAALEVERRRIYDVINVFEALELVSRKAKNTYTWRGLDALRTTLG

KLKTLTTTEANTPKRTNSDPDSPNTRADRSLGVLTQRFIMMFLVSSTGSVQLDTAADRLIFGLDCPPEKKNKNQLRRLYD

IANILSSLDLVKKDSGSQKGKTKFVWCGEDPAKLPAINTDEFLSPDRQPTKRSQRQSLLAGRPDSSACTPVAKRARQSAE

SPPKSDETPTSSQPLSGGPSRTLPFSFPTPIAQPNFEDDKADLPSTSATSSMPSSDIACTTSADASGPFTTTSCAPASSL

ASSTLTPFPMHALTSLPPNAQIHIVVHHQSTPQDLIGLTRSALPSSSQVFVVSPSALQLNPNGPIHVSHG

>E2F7/8-Nv gi|156344376|ref|XP_001621163.1| hypothetical protein NEMVEDRAFT_v1g222302 [Nematostella vectensis]

MDFESQKDGLSCSIAPVGWEKATESSDSEESECNNSQPVPLARHSSLAEITTISNNCKTSPLAKSQLSVDEGDLQKSVSL

ELDSVLDLAVRSNQPSGTKMQLKSRVPSTPTKQQPESRTPSKHSTDEPLTPTANLKMLVSAASPAIRDREIKKRELFTDS

PGSPAVVPSFALPVFDSKVVIRNGSFTRVEMENGSEKIAISRKDKSLGLLCQRFLAKYPDYPTSDESIEISLDETRQVTL

DDAANVLIDSSEEGQAKYKTKVRRLYDIANILSSLQLIQKVHIHNIQHGRKPGFRWIGMDLDTLDDKRRQDPVPAVKTEK

LTPKSHQSLLAEIEDKEESLFGRLISRRPSQQQIQRPRHDSSKSKLPRSWSDCRHASKKRALSSDKEKANSEDGYSIQEL

ADAAAAESDVASSPTAVKFQSELKKLQEQYPDHILSSFELFPCEKASIIYLHLFYYIPRRTVGHVNRLPDASGAS

>Os02g0739700-Os gi|115448579|ref|NP_001048069.1| Os02g0739700 [Oryza sativa (japonica cultivar-group)]

MATAAVMAAVPSSSPADAAEAVVMTEAVPSLPQRQQPVFVEGRGGKLRDHAYSRKQKSLGLLCSNFVALYNRDDVESIGL

DDAARRLGVERRRIYDIVNVLESVGILVRKAKNRYSWIGFGGVPMALRELKERALREKSGLAPLPVEEPSAAIMSDDEDE

DKMGDADGDTESEKLSQPVDNPSDNKPGAPRCRLRSDHRKEKSLGLLTQNFVKLFLTMEVDTISLDEAAKLLLGEGHAEN

SMRTKVRRLYDIANVLSSLNFIDKIQQADSRKPAFRWLGSAGKPKAENGVTIAVPPPGKTISNKRAFGTELTNIDINRSR

LDSTIPKKAKLTLSGGEILKNCKLSVQKQLGQGSKGGFVYGPFHPAGARKQELDNGNKGHTDNVQNWESLAASFRPQYQN

QALGDLFAHYVEAWKSWYSEFAQGSSMMQQHFGMPVINQFL

>Os06g0245900-Os gi|115467360|ref|NP_001057279.1| Os06g0245900 [Oryza sativa (japonica cultivar-group)]

MAAAADAPPPPPEVAPPAPAPAPAPAPYQPPRLAVADGAGGGGGGGGKPCRHHAYSRKQKSLGLLCTNFVALYDREDVES

VGLDDAARRLGVERRRIYDIVNVLESIGMLVRRAKNRYTWIGFGGVPAALAKLKEMSLRAVSSVASPSLDETSAANVSDD

EDDDKLDDAEGDAESEKLSLSQSIDNPSDKPDAPPCKLRSEHRKEKSLGLLTQNFVKLFLTMEIETISLDEAAKRLLGEG

HAANNMRTKVRRLYDIANVLSSLNLIEKTQQADSRKPAFRWLGQAKRNEGVTVALPPTKTLPNKRAFGTDLTNIDNKRGK

LDSTMENRGKPTQDGGNLFNNLQRQLGQENRSDFVYGPFHPAVARKQEHGNRTVQEKERKSIQDWENLASSFRPQYQNPG

LNDLFGHYMEAWRSWYSDLRRDRAS

>DEL1-At gi|30692988|ref|NP_851012.1| DEL1 (DP-E2F-like 1); transcription factor [Arabidopsis thaliana]

MSDLSPERFKLAVTSPSSIPESSSALQLHHSYSRKQKSLGLLCTNFLALYNREGIEMVGLDDAASKLGVERRRIYDIVNV

LESVGVLTRRAKNQYTWKGFSAIPGALKELQEEGVKDTFHRFYVNENVKGSDDEDDDEESSQPHSSSQTDSSKPGSLPQS

SDPSKIDNRREKSLGLLTQNFIKLFICSEAIRIISLDDAAKLLLGDAHNTSIMRTKVRRLYDIANVLSSMNLIEKTHTLD

SRKPAFKWLGYNGEPTFTLSSDLLQLESRKRAFGTDITNVNVKRSKSSSSSQENATERRLKMKKHSTPESSYNKSFDVHE

SRHGSRGGYHFGPFAPGTGTYPTAGLEDNSRRAFDVENLDSDYRPSYQNQGAYILFTSI

>DEL2-At gi|15242176|ref|NP_197000.1| DEL2/E2FD/E2L1 (DP-E2F-LIKE 2); DNA binding / transcription factor [Arabidopsis thaliana]

MDSLALAPQVYSRKDKSLGVLVANFLTLYNRPDVDLFGLDDAAAKLGVERRRIYDVVNILESIGLVARSGKNQYSWKGFG

AVPRALSELKEEGMKEKFAIVPFVAKSEMVVYEKEGEESFMLSPDDQEFSPSPRPDNRKERTLWLLAQNFVKLFLCSDDD

LVTFDSATKALLNESQDMNMRKKVRRLYDIANVFSSMKLIEKTHVPETKKPAYRWLGSKTIFENRFIDGSASLCDRNVPK

KRAFGTELTNVNAKRNKSGCSKEDSKRNGNQNTSIVIKQEQCDDVKPDVKNFASGSSTPAGTSESNDMGNNIRPRGRLGV

IEALSTLYQPSYCNPELLGLFAHYNETFRSYQEEFGREK

>DEL3-At gi|22330789|ref|NP_186782.2| DEL3 (DP-E2F-like 3); transcription factor [Arabidopsis thaliana]

MSSAIVVSQDAESLGLQIYSRKEKSLGVLVSNFLRLYNRDDVDLIGLDDAAGQLGVERRRIYDVVNILESIGIVARRGKN

QYSWKGFGEIPRSLDELKEEGMRERLGYSSSNNSDKVSNGCEREEPLTLTPDDQENSSSSKMDQKKEKSLWLLAQNFVKM

FLCSDDDLITLDSAAKALLSDSPDSVHMRTKVRRLYDIANVFASMNLIEKTHIPVTRKPAYRWLGSKSIAERGLSLFNSG

EPKRVFGTEITNLRAKRNKTYCSSIRKQIGYKKHDEENTEQESKPAASKYVFGPFSPIGASKTNNDKVGKGRLLEIEALA

STYQPQYCNQEITGLLGHFTEAWKKWYAEVDRNK

**RB family proteins:**

>Rb1-Hs gi|108773787|ref|NP_000312.2| retinoblastoma 1 [Homo sapiens]

MPPKTPRKTAATAAAAAAEPPAPPPPPPPEEDPEQDSGPEDLPLVRLEFEETEEPDFTALCQKLKIPDHVRERAWLTWEK

VSSVDGVLGGYIQKKKELWGICIFIAAVDLDEMSFTFTELQKNIEISVHKFFNLLKEIDTSTKVDNAMSRLLKKYDVLFA

LFSKLERTCELIYLTQPSSSISTEINSALVLKVSWITFLLAKGEVLQMEDDLVISFQLMLCVLDYFIKLSPPMLLKEPYK

TAVIPINGSPRTPRRGQNRSARIAKQLENDTRIIEVLCKEHECNIDEVKNVYFKNFIPFMNSLGLVTSNGLPEVENLSKR

YEEIYLKNKDLDARLFLDHDKTLQTDSIDSFETQRTPRKSNLDEEVNVIPPHTPVRTVMNTIQQLMMILNSASDQPSENL

ISYFNNCTVNPKESILKRVKDIGYIFKEKFAKAVGQGCVEIGSQRYKLGVRLYYRVMESMLKSEEERLSIQNFSKLLNDN

IFHMSLLACALEVVMATYSRSTSQNLDSGTDLSFPWILNVLNLKAFDFYKVIESFIKAEGNLTREMIKHLERCEHRIMES

LAWLSDSPLFDLIKQSKDREGPTDHLESACPLNLPLQNNHTAADMYLSPVRSPKKKGSTTRVNSTANAETQATSAFQTQK

PLKSTSLSLFYKKVYRLAYLRLNTLCERLLSEHPELEHIIWTLFQHTLQNEYELMRDRHLDQIMMCSMYGICKVKNIDLK

FKIIVTAYKDLPHAVQETFKRVLIKEEEYDSIIVFYNSVFMQRLKTNILQYASTRPPTLSPIPHIPRSPYKFPSSPLRIP

GGNIYISPLKSPYKISEGLPTPTKMTPRSRILVSIGESFGTSEKFQKINQMVCNSDRVLKRSAEGSNPPKPLKKLRFDIE

GSDEADGSKHLPGESKFQQKLAEMTSTRTRMQKQKMNDSMDTSNKEEK

>Rbl1-Hs gi|34577079|ref|NP_002886.2| retinoblastoma-like protein 1 isoform a [Homo sapiens]

MFEDKPHAEGAAVVAAAGEALQALCQELNLDEGSAAEALDDFTAIRGNYSLEGEVTHWLACSLYVACRKSIIPTVGKGIM

EGNCVSLTRILRSAKLSLIQFFSKMKKWMDMSNLPQEFRERIERLERNFEVSTVIFKKYEPIFLDIFQNPYEEPPKLPRS

RKQRRIPCSVKDLFNFCWTLFVYTKGNFRMIGDDLVNSYHLLLCCLDLIFANAIMCPNRQDLLNPSFKGLPSDFHTADFT

ASEEPPCIIAVLCELHDGLLVEAKGIKEHYFKPYISKLFDRKILKGECLLDLSSFTDNSKAVNKEYEEYVLTVGDFDERI

FLGADAEEEIGTPRKFTRDTPLGKLTAQANVEYNLQQHFEKKRSFAPSTPLTGRRYLREKEAVITPVASATQSVSRLQSI

VAGLKNAPSDQLINIFESCVRNPVENIMKILKGIGETFCQHYTQSTDEQPGSHIDFAVNRLKLAEILYYKILETVMVQET

RRLHGMDMSVLLEQDIFHRSLMACCLEIVLFAYSSPRTFPWIIEVLNLQPFYFYKVIEVVIRSEEGLSRDMVKHLNSIEE

QILESLAWSHDSALWEALQVSANKVPTCEEVIFPNNFETGNGGNVQGHLPLMPMSPLMHPRVKEVRTDSGSLRRDMQPLS

PISVHERYSSPTAGSAKRRLFGEDPPKEMLMDKIITEGTKLKIAPSSSITAENVSILPGQTLLTMATAPVTGTTGHKVTI

PLHGVANDAGEITLIPLSMNTNQESKVKSPVSLTAHSLIGASPKQTNLTKAQEVHSTGINRPKRTGSLALFYRKVYHLAS

VRLRDLCLKLDVSNELRRKIWTCFEFTLVHCPDLMKDRHLDQLLLCAFYIMAKVTKEERTFQEIMKSYRNQPQANSHVYR

SVLLKSIPREVVAYNKNINDDFEMIDCDLEDATKTPDCSSGPVKEERGDLIKFYNTIYVGRVKSFALKYDLANQDHMMDA

PPLSPFPHIKQQPGSPRRISQQHSIYISPHKNGSGLTPRSALLYKFNGSPSKSLKDINNMIRQGEQRTKKRVIAIDSDAE

SPAKRVCQENDDVLLKRLQDVVSERANH

>Rbl2-Hs gi|172072597|ref|NP_005602.3| retinoblastoma-like 2 (p130) [Homo sapiens]

MPSGGDQSPPPPPPPPAAAASDEEEEDDGEAEDAAPPAESPTPQIQQRFDELCSRLNMDEAARAEAWDSYRSMSESYTLE

GNDLHWLACALYVACRKSVPTVSKGTVEGNYVSLTRILKCSEQSLIEFFNKMKKWEDMANLPPHFRERTERLERNFTVSA

VIFKKYEPIFQDIFKYPQEEQPRQQRGRKQRRQPCTVSEIFHFCWVLFIYAKGNFPMISDDLVNSYHLLLCALDLVYGNA

LQCSNRKELVNPNFKGLSEDFHAKDSKPSSDPPCIIEKLCSLHDGLVLEAKGIKEHFWKPYIRKLYEKKLLKGKEENLTG

FLEPGNFGESFKAINKAYEEYVLSVGNLDERIFLGEDAEEEIGTLSRCLNAGSGTETAERVQMKNILQQHFDKSKALRIS

TPLTGVRYIKENSPCVTPVSTATHSLSRLHTMLTGLRNAPSEKLEQILRTCSRDPTQAIANRLKEMFEIYSQHFQPDEDF

SNCAKEIASKHFRFAEMLYYKVLESVIEQEQKRLGDMDLSGILEQDAFHRSLLACCLEVVTFSYKPPGNFPFITEIFDVP

LYHFYKVIEVFIRAEDGLCREVVKHLNQIEEQILDHLAWKPESPLWEKIRDNENRVPTCEEVMPPQNLERADEICIAGSP

LTPRRVTEVRADTGGLGRSITSPTTLYDRYSSPPASTTRRRLFVENDSPSDGGTPGRMPPQPLVNAVPVQNVSGETVSVT

PVPGQTLVTMATATVTANNGQTVTIPVQGIANENGGITFFPVQVNVGGQAQAVTGSIQPLSAQALAGSLSSQQVTGTTLQ

VPGQVAIQQISPGGQQQKQGQSVTSSSNRPRKTSSLSLFFRKVYHLAAVRLRDLCAKLDISDELRKKIWTCFEFSIIQCP

ELMMDRHLDQLLMCAIYVMAKVTKEDKSFQNIMRCYRTQPQARSQVYRSVLIKGKRKRRNSGSSDSRSHQNSPTELNKDR

TSRDSSPVMRSSSTLPVPQPSSAPPTPTRLTGANSDMEEEERGDLIQFYNNIYIKQIKTFAMKYSQANMDAPPLSPYPFV

RTGSPRRIQLSQNHPVYISPHKNETMLSPREKIFYYFSNSPSKRLREINSMIRTGETPTKKRGILLEDGSESPAKRICPE

NHSALLRRLQDVANDRGSH

>Rb1-Cf gi|73989274|ref|XP_534118.2| PREDICTED: similar to Retinoblastoma-associated protein (PP110) (P105-RB) (RB) [Canis familiaris]

MELDLGFPLTGHVLKLVFEETEEPDFTALCQKLKIPDHVRERAWLTWEKVSSVDGVLEGYVQRKKELWGICIFIAAVDLD

EMPFTFTELQKTIEISVYRFFDLLKEIDTSTKVDNAMSRLLKKYDVLCALYSKLERTCELIYLTQPSSLISTEINSVLVL

KVSWITFLLAKGEVLQMEDDLVISFQLMLCVLDYFIKLSPPALLKEPYKTAVIPFNGSPRTPRRGQNRSARIAKQLENDT

RIIEVLCKEHECNIDEVKNVYFKNFIPFMNSLGIVASNGLPEVESLSRQYEEIYLKNKDFDARLFLDHDKTLQIDPTDSF

EMQRTPQKSNSDEEVNVILPQTPVRTVMNTIQQLMMILNSASDQPSENLISYFNNCTVNPKESILKRVKDVGYIFKEKFA

KAVGQGCMEIGSQRYKLGVRLYYRVMESMLKSEEERLSIQNFSKLLNDDIFHMSLLACALEVVMATYSRSTSQNLDTGTD

LSFPWILNVLNLKAFDFYKVIESFIKAEANLTREMIKHLERCEHRIMESLAWLSDSPLFDLIKQAKDREGPADHLESACT

LNLPLQSNHTAADMYLSPVRSPKKKGSTIRVNSTVNTEAQATSAFQTQKPLKSTSLSLFYKKVYRLAYLRLNTLCARLLS

DHPELEHIIWTLFQHTLQNEYELMRDRHLDQIMMCSMYGICKVKNIDLKFKIIVTAYKDLPHAVQETFKRVLIREEEYDS

IIVFYNSVFMQRLKTNILQYASTRPPTLSPIPHIPRSPYKFSSSPLRIPGGNIYISPLKNPYKISEGVPTPTKMTPRSRI

LVSIGESFGTSEKFQKINQMVCNSDRVLKRSAEGSNPPKPLKKLRFDIEGSDEADGSKHLPGESKFQQKLAEMTSTRTRM

QKQKLNDSMDTSNREEK

>Rbl1-Cf gi|73992372|ref|XP_534412.2| PREDICTED: similar to retinoblastoma-like protein 1 isoform a [Canis familiaris]

MFEDDPHAEGAAVVAAAGEALQALCQELNLDEGSAAEALDDFTAIRGNYSLEGEVIHWLACSLYVACRKSIIPTVGKGIM

EGNCVSLTRILRSAKLSLIQFFSKMKKWMDMSNLPQEFRERVERLERNFEVSTVIFKKFEPIFLDIFQNPYEEQPKFPRS

RKQRRIPCSVKDLFNFCWTLFVYTKGNFRMIGDDLVNSYHLLLCCLDLIFANAIICPNRRDLLNPTFKGLPSDFHTADFK

ASEEPPCIIAVLCELHDGLLVEAKGIKEHYFKPYISKLFDRKVLKGECLLDLSSFTDNSKAVNKEYEEYVLTVGDFDERI

FLGADAEEEIGTPRKFTGETPLGKLTAQANVDCSLQQHFEKKTSFAPSTPLTGRRYLREKEAVITPVASATQSVSRLQSI

VAGLKNAPSEQLINIFESCIRNPMENIMKIVKGIGETFCQHYTQSTDEQPGSHIDFAINRLKLAEILYYKILETVMVQET

RRLHGMDMSVLLEQDIFHRSLMACCLEIVLFAYSSPRTFPWIIEVLNLRPFYFYKVIEVVIRSEEGLSRDMVKHLNSIEE

QILESLAWSHDSALWEALQASANKVPTCEEVIFPNNFETGNGGNVQGHLPMMPMSPLMHPRVKEVRTDSGSLRRDMQPLS

PISVHERYSSPTAGSAKRRLFGEDPPKEMLVDRIITEGTKLKIAPSSSITAENISISPGQSLLTMATAIVTGTTGHKVTI

PLHGIANDAGEITLIPISMNTAQESKVESPISLTAQSLIGASPKQTHLTKAQEAHPTGISKPKRTGSLALFYRKVYHLAS

VRLRDLCLKLDVSNELRRKIWTCFEFTLVHCPDLMKDRHLDQLLLCAFYIMAKVTKEERTFQEIMKSYRNQPQANSHVYR

SVLLKSIPREVVAYNKNINGDFEMTDCDLEDATKTPDCSSGPVKEERGDLIKFYNTIYVARVKSFALKYDLSNQDHVMEA

PPLSPFPHIKQQPGSPRRISQQHSIYVSPHKNGSGLTPRSALLYKFNGSPSKSLKDINNMIRQGEQRTKKRAITIDGDAE

SPAKRLCQENDDVLLKRLQDVVSERANH

>Rbl2-Cf gi|73949852|ref|XP_535303.2| PREDICTED: similar to Retinoblastoma-like protein 2 (130 kDa retinoblastoma-associated protein) (PRB2) (P130) (RBR-2) isoform 1 [Canis familiaris]

MPSGGDQSPPPPPPPPAAAASDEEEEDDGEAEDAAQPARSPAPQTQQRFDELCSRLNMDEAARAEAWDSYRNMSESYTLE

GNDLHWLACALYVACRKSVPTVSKGTVEGNYVSLTRILRCSEQSLIEFFNKMKKWEDMANLPPHFRERTERLERNFTVSA

VIFKKYEPIFQDIFKYPQEEQPRQQRGRKQRRQPCTVSEVFHFCWVLFIYAKGNFPMISDDLVNSYHLLLCALDLVYGNA

LQCSNRKELVNPNFKGLSEDFHAKDSKPSSDPPCVIEKLCSLHDGLVLEAKGIKEHFWKPYIRKLYEKKLLKGKEENLTG

FLEPGNFGESFKAINKAYEEYVLSVGNLDERIFLGDDAEEEIGTLSKCLNSGSGTETAERVQMKNILQQHFDKSKALRIS

TPLTGVRYMKDNSPCVTPVSTATHSLSRLHTMLTGLRNAPSEKLEQILRTCSRDPTQAIANRLKEMYEIYSQHCQPDEDF

SNSKEIASKHFRFAEMLYYKVLESVIEQEQKRLGDMDLSGILEQDAFHRSLLACCLEVVTFSYKPPGNFPFITEIFDVPL

YHFYKVIEVFIRAEDGLCREVVKHLNQIEEQILDHLAWKPESPLWDRIRDNENRVPTCEEVMPPQNLERADDICIAGSPL

TPRRVSEVRADTGGLGRSITSPATLYDRYSSPTASSTRRRLFVENDSPSDGGTPGRIPPQPLVNAVPVQNVSGETVSVTP

VPGQTLVTMATATVTANNGQTVTIPVQGIANENGGITFFPVQVNVGGQAQAVTGSIQPLSAQALAGSLSSQQVTGTTLQV

PGQVAIQQISPGGHQQKQGPPLTGSSIRPRKTSSLSLFFRKVYHLAGVRLRDLCAKLDISDELRKKIWTCFEFSIIQCPE

LMMDRHLDQLLMCAIYVMAKVTKEDKSFQNIMRCYRTQPQARSQVYRSVLIKGKRKRRNSGSSDSRSHQNSPTELNKDRT

SRDSSPVMRSSSTLPVPQPSSAPPTPTRLTGANSDMEEEERGDLIQFYNNIYIKQIKTFAMKYSQANIMDAPPLSPYPFV

RTGSPRRIQLSQNHPVYISPHKNETMLSPREKIFYYFSNSPSKRLREINSMIRTGETPTKKRGILLEDGSESPAKRICPE

NHSALLRRLQDVANDRGSH

>Rb1-Bt gi|116004031|ref|NP_001070375.1| retinoblastoma 1 [Bos taurus]

MPPKTPRRAAAAAAAAAAEPPPPPLPPPPEEDPEQDSGSEDLPLARLEFEETEEPDFTALCQKLKLPDHVRERAWLTWEK

VSSVDGVLEGYVQKKKELWGICIFIAAVDLDEMPFTFTELQKNIETSVYKFFDLLKEIDTSTKVDNAMSRLLKKYNVLCA

LYSKLERTCELIYLTQPSSSISTEINSMLVLKVSWITFLLAKGQVLQMEDDLVISFQLMLCVLDYFIKLSPPALLKDPYK

TAVIPINGSPRTPRRGQNRSARIAKQLENDTRIIEVLCKEHECNIDEVKNVYFKNFIPFMNSLGIVASNGLPEVENLSKQ

YEEIYLKNKDLDARLFLDHDKTLQADPTDSFEMQRTPRKSNPDEEVNMILPQTPVRTVMNTIQQLMMILNSASDQPSENL

ISYFNNCTVNPKDSILKRVKDIGDVFKEKFAKAVGQGCMEIGSQRYKLGVRLYYRVMESMLKSEEERLSIQNFSKLLNDN

IFHMSLLACALEVVMATYSRSMSQSLDTGTDLSFPWILNVFNLKAFDFYKVIESFIKAEANLTREMIKHLERCEHRIMES

LAWLSDSPLFDLIKQAKDREGPVDHFEPACTLNLPLQNNHTAADMYLSPVRSPKKKGPTPRVNSTPNSEAQATSAFQTQK

PLKSTSLSLFYKKVYRLAYLRLNTLCARFLSDHPELEHIIWTLFQHTLQNEYELMRDRHLDQIMMCSMYGICKVKNIDLK

FKIIVTAYKDLPHAVQETFKRVLIREEEYDSIIVFYNSVFMQRLKTNILQYASTRPPTLSPIPHIPRSPYKFSSSPLRIP

GGNIYISPLNNPYKISEGLPTPTKMTPRSRILVSIGESFGTSEKFQKINQMVCNSDRVLKRSAEGSNPPKPLKKLRFDIE

GSDEADGSKHLPGESKFQQKLAEMTSTRTRMQKQKMNESVDTSNREEK

>Rbl1-Bt gi|119905907|ref|XP_603207.3| PREDICTED: similar to retinoblastoma-like protein 1 [Bos taurus]

MDEDDPHAEGAAVVAAAGEALQALCQELNLDEGSAAEALDDFTAIRGNYSLEGEVIHWLACSLYVACRKSIIPTVGKGIM

EGNCVSLTRILRSAKLSLIQFFSKMKKWMDMSNLPQEFRERIERLERNFEVSTVIFKKFEPIFLDIFQNPYEEPPKLPRS

RKQRRIPCSVKELFNFCWTLFVYTKGNFRMIGDDLVNSYHLLLCCLDLIFANAIMCPNRQELLNPSFKGLPSNFQTADFR

ASEEPPCIIPVLCELHDGLLVEAKGIKEHYFKPYISKLFDRKILKGECLLDLCSFTDNSKAVNKEYEEYVLTVGDFDERI

FLGADAEEEIGTPRKFTGDGPLGKLTAQANVECNLQHHFEKKTSFAPSTPLTGRRYLREKEAVITPVASATQSVSRLQSI

VAGLKNAPSEQLINIFESCMRNPMENIMKIVKGIGETFCQHYTQSTDEQPGSHIDFAVNRLKLAEILYYKILETVMVQET

RRLHGMDMSVLLEQDIFHHSLMACCLEIVLFAYSSPRTFPWIIEVLNLRPFYFYKVIEVVIRSEEGLSRDMVKHLNSIEE

QILESLAWSHDSALWEALQASENRVPTCEEVIFPNNFETGSGGNVQGHLPMMPMSPLMHPRVKEVRTDSGSLRKDMQPLS

PISVHERYSSPTAGSAKRRLFGEDPPKEILMDRIITEGTKLKIAPSSSITAENISISPGHSLLTMATAIVAGTTGHKVTI

PLHGIANDAGEITLIPISMNTTQESKVESPVSLTAQSLIGASPKQTHLTKAQEVHPIGISKPKRTGSLALFYRKVYHLAS

VRLRDLCLKLDVSNELRRKIWTCFEFTLVHCPDLMKDRHLDQLLLCAFYIMAKVTKEERTFQEIMKSYRNQPQANSHVYR

SVLLKSIPREVVAYSKNLNGDFEMTDCDLEDATKTPDCSSGPVKEERGDLIKFYNTIYVGRVKSFALKYDLSNQDHVMEA

PPLSPFPHIKQQPGSPRRISQQHSIYVSPHKNGSGLTPRSALLYKFNGSPSKSLKDINNMIRQGEQRTKKRAITIDGDAE

SPAKRLCQENDDVLLKRLQDVVSERANH

>Rbl2-Bt gi|148225699|ref|NP_001091542.1| retinoblastoma-like 2 (p130) [Bos taurus]

MPSGGDQSPPPPPPPPAAAASDEEEEDDGEAEDAAQPSRSPAPQTQQRFDELCSRLNMDEAARAEAWESYRSMSESYTLE

GNDLHWLACALYVACRKSVPTVSKGTVEGNYVSLTRILRCSEQSLIEFFNKMKKWEDMANLPPHFRERTERLERNFTVSA

VIFKKYEPIFQDIFKYPQEEQPRQQRGRKQRRQPCTVSEVFHFCWVLFIYAKGNFPMISDDLVNSYHLLLCALDLVYGNA

LQCSNRKELVNPNFKGLSEDFHAKDSKPSSDPPCVIEKLCSLHDGLVLEAKGIKEHFWKPYIRKLYEKKLLKGKEENLTG

FLEPGNFGESFKAINKAYEEYVLSVGNLDERIFLGEDAEEEIGTLSRCLNTGSGAETAERVQMKNILQQHFDKSKALRVS

TPLTGVRYIKDSSPCVTPVSTATHSLSRLHTMLTGLRNAPSERLEQTLRSCSRDPTQAIANRLKEMYEIYSQHFQSEEDG

SNCAKDIASKHFRFAEMLYYKVLESVIEQEQKRLGDMDLSGILEQDAFHRSLLACCLEVVTFSYKPPGNFPFITEIFDVP

LYHFYKVIEVFIRAEDGLCREVVKHLNQIEEQILDHLAWKPESPLWDRIRDNENRVPTCEEVMPPQNLERADEICIAGSP

LTPRRVSEVRADSGGLGRSISSPTTLYDRYSSPTASSTRRRLFVENDSPTDGGTPGRTPPQPLVNAVPVQNVAGEAVSVT

PVPGQTLVTMATATVTANNGQTVTIPVQGIANENGGITFFPVQVNVGGQAQAVTGSIQPLSAQALAGSLSSQQVTGTTLQ

VPGQVAIQQISPGGPQQKQGLPLTSSSIRPRKTSSLSLFFRKVYHLAGVRLRDLCAKLDISDELRKKIWTCFEFSIIQCP

ELMMDRHLDQLLMCAIYVMAKVTKEDKSFQNIMRCYRTQPQARSQVYRSVLIKGKRRRRNSGSSDSRSHQNSPTELNKDR

TSRDSSPVMRSSSTLPVPQPSSAPPTPTRLTGANSDVEEEERGDLIQFYNNIYIRQMKTFAMKYSQANAMDAPPLSPYPF

VRTGSPRRIQLSQNHPVYISPHKNEAMLSPREKIFYYFSNSPSKRLREINSMIRTGETPTKKRGILLEDGSESPAKRICP

ENHSALLRRLQDVANDRGSH

>Rb1-Mm gi|188528630|ref|NP_033055.2| retinoblastoma 1 [Mus musculus]

MPPKAPRRAAAAEPPPPPPPPPREDDPAQDSGPEELPLARLEFEEIEEPEFIALCQKLKVPDHVRERAWLTWEKVSSVDG

ILEGYIQKKKELWGICIFIAAVDLDEMPFTFTELQKSIETSVYKFFDLLKEIDTSTKVDNAMSRLLKKYNVLCALYSKLE

RTCELIYLTQPSSALSTEINSMLVLKISWITFLLAKGEVLQMEDDLVISFQLMLCVVDYFIKFSPPALLREPYKTAAIPI

NGSPRTPRRGQNRSARIAKQLENDTRIIEVLCKEHECNIDEVKNVYFKNFIPFINSLGIVSSNGLPEVESLSKRYEEVYL

KNKDLDARLFLDHDKTLQTDPIDSFETERTPRKNNPDEEANVVTPHTPVRTVMNTIQQLMVILNSASDQPSENLISYFNN

CTVNPKENILKRVKDVGHIFKEKFANAVGQGCVDIGVQRYKLGVRLYYRVMESMLKSEEERLSIQNFSKLLNDNIFHMSL

LACALEVVMATYSRSTLQHLDSGTDLSFPWILNVLNLKAFDFYKVIESFIKVEANLTREMIKHLERCEHRIMESLAWLSD

SPLFDLIKQSKDGEGPDNLEPACPLSLPLQGNHTAADMYLSPLRSPKKRTSTTRVNSAANTETQAASAFHTQKPLKSTSL

ALFYKKVYRLAYLRLNTLCARLLSDHPELEHIIWTLFQHTLQNEYELMRDRHLDQIMMCSMYGICKVKNIDLKFKIIVTA

YKDLPHAAQETFKRVLIREEEFDSIIVFYNSVFMQRLKTNILQYASTRPPTLSPIPHIPRSPYKFSSSPLRIPGGNIYIS

PLKSPYKISEGLPTPTKMTPRSRILVSIGESFGTSEKFQKINQMVCNSDRVLKRSAEGGNPPKPLKKLRFDIEGADEADG

SKHLPAESKFQQKLAEMTSTRTRMQKQRMNESKDVSNKEEK

>Rbl1-Mm gi|213417847|ref|NP_035379.2| retinoblastoma-like protein 1 isoform 1 [Mus musculus]

MFEDEPHAEGAAAVAAAREALQALCQELNLDEGSAAEALDDFTAIRGNYSLEGEVIHWLACSLYVACRKSIIPTVGKGVM

EGNCVSLTRILRSAKLSLIQFFSKMKKWMDMSNLPQEFRERIERLERNFEVSTVIFKKFEPIFLDIFQNPYEEPPKLPRS

RKQRRIPCSVKDLFNFCWTLFVYTKGNFRMIGDDLVNSYHLLLCCLDLIFANAIMCPNRRDLLNPSFKGLPSDFHAPDFK

AAEEPPCIIAVLCDLHDGLLVEAKGIKEHYFKPYISKLFDKKILKGECLLDLSSFTDNSKAVNKEYEEYVLTVGDFDERI

FLGADAEEEIGTPRKFTADTPFGKLTSQASVECNLQQHFEKKRSFAPSTPLTGRRYLQEKEAVTTPVASATQSVSRLQSI

VAGLKSAPSEQLLNIFESCMRNPMGNIIKIVKGIGETFCQHYTQSTDKQPGSHIDFAVNRLKLAEILYYKILETIMVQET

RRLHGMDMSVLLEQDIFHKSLMACCLEIVLFAYSSPRTFPWIIEVLDLQPFYFYKVIEVVIRSEEGLSRDMVKHLNSIEE

QILESLAWTNNSALWEALHASANRVPSCEEVIFPNNFEIGNGGNVQGHLPMMPMSPIIHPRVKEVRTDSGSLRQDMQPLS

PISVHERYSSPAAGSAKRRLFGDDPPKDTLMDKIMAEGTKLKIAPSSVTAESLSISPGQALLTMATTTVTGTTGRKVTVP

LHGIANDAGEITLVPISMNPTQESTAESPVSLTAQSLIGTSPKQTHLTKAQDAHLTGVSKPKRTGSLALFYRKVYHLASV

RLRDLCLKLDVSNELRRKIWTCFEFTLVHCPDLMKDRHLDQLLLCAFYIMAKVTKEERTFQEIMKSYRNQPQANSHVYRS

VLLKSIPGGVVVYNGDCEMTDGDIEDATKTPNCSSEPVKEERGDLIKFYNTVYVGRVKSFALKYDLSNQDHIMDAPPLSP

FPHIKQQPGSPRRISQQHSLYVSPHKNGAGLTPRSALLYKFNGSPSKSLKDINNMIRQGEQKTKKRVIAISGDADSPAKR

LCQENDDVLLKRLQDVVSERANH

>Rbl2-Mm gi|170932488|ref|NP_035380.3| retinoblastoma-like 2 [Mus musculus]

MASGGNQSPPPPPAAAASSEEEEEDGDAADRAQPAGSPSHQIQQRFEELCSRLNMDEAARAEAWSSYRSMSESYTLEGND

LHWLACALYVACRKSVPTVSKGTAEGNYVSLTRILRCSEQSLIEFFNKMKKWEDMANLPPHFRERTERLERNFTVSAVIF

KKYEPIFQDIFKYPQEEQPRQQRGRKQRRQPCTTSEIFHFCWVLFIYAKGNFPMISDDLVNSYHLLLCALDLVYGNALQC

SNRKELVNPNFKGLSEDCHPKDSKASSDPPCVIEKLCSLHDGLVLEAKGIKEHFWKPYIRKLFEKKLLKGKEENLTGFLE

PGNFGESFKAVNKAYEEYVLAAGNLDERVFLGEDAEEEVGTLSRCLSAASGTESAERTQMRDILQQHLDKSKALRVCTPL

TGVRYVQENSPCVTPVSTAAHSLSRLHTMLSGLRNAPSEKLERILRSCSRDPTQAIADRLKEMYEIYSQHFQPDENFSNC

AKEIANKHFRFAEMLYYKVLESVIEQEQKRLGDMDLSGVLEHDAFHRSLLACCLEVVAFSHKPPGNFPFIAEIFDVPHYH

FYKVIEVFIRAEDGLCREVVKHLNQIEEQILDHLAWKTKSPLWDRIRDNENRVPTCEEVMPPQNLERTDEIYIAGSPLTP

RRVGEVRADAGGLGRSITSPTTLYDRYSSPTVSTTRRRLFENDSPSEGSTSGRIPPQPLVNAVPVQNVPGETVSVTPVPG

QTLVTMATATVTANNGQTVTIPVQGIANENGGITFFPVQVNVGGQAQAVAGSIQPLSAQALAGSLSSQQVTGTTLQVPGP

VAIQQISPGGQQQNPGQPLTSSSIRPRKTSSLALFFRKVYYLAGVRLRDLCIKLDISDELRKKIWTCFEFSIIQCTELMM

DRHLDQLLMCAIYVMAKVTKEDRSFQNIMRCYRTQPQARSQVYRSVLIKGKRRNSGSSESRSHQNSPTELNTDRASRDSS

PVMRSNSTLPVPQPSSAPPTPTRLTGASSDVEEEERGDLIQFYNNIYRKQIQAFAMKYSQANAQTDTPPLSPYPFVRTGS

PRRVQLSQSHPIYISPHNNEAMPSPREKIFYYFSNSPSKRLREINSMIRTGETPTKKRGILLDDGSESPAKRICPENHSA

LLRRLQDVANDRGSQ

>Rb1-Rn gi|109501744|ref|XP_344435.3| PREDICTED: similar to Retinoblastoma-associated protein (PP105) (RB) [Rattus norvegicus]

MPPKAPRRTAAAEPPPPPPPPPEDDPAQDSDPEELPLIRLEFEKIEEPEFIALCQKLKVPDHVRERAWLTWEKVSSVDGI

LEGYIQKKKELWGICIFIAAVDLDEMPFTFTELQKSIETSVYKFFDLLKEIDTSTKVDNAVSRLLKKYNVLCALYSKLER

TCGLIYLTQPSSGLSTEINSMLVLKVSWITFLLAKGEVVQMEDDLVISFQLMLCVLDYFIKLSPPALLREPYKTAATPIN

GSPRTPRRGQNRSARIAKQLESDTRTIEVLCKEHECNVDEVKNVYFKNFIPFISSLGIVSSNGLPELESLSKRYEEVYLK

SKDLDARLFLDHDKTLQTDTIDSFETERTPRKSNPDEEANMVTPHTPVRTVMNTIQQLMVILNSASDQPSENLISYFSNC

TVNPKENILKRVKDVGHIFKEKFASAVGQGCIDIGAQRYKLGVRLYYRVMESMLKSEEERLSIQNFSKLLNDNIFHMSLL

ACALEVVMATYSRSMLQNLDSGTDLSFPWILNVLNLKAFDFYKVIESFIKVEANLTREMIKHLERCEHRIMESLAWLSDS

PLFDLIKQSKDGEGPDHLESACSLSLPLQSNHTAADMYLSPIRSPKKRTSTTRVNSAANTETQAASAFHTQKPLKSTSLS

LFYKKVYRLAYLRLNTLCARLLSDHPELEHIIWTLFQHTLENEYELMKDRHLDQIMMCSMYGICKVKNIDLKFKIIVTAY

KDLPHAAQETFKRVLIREEEFDSIIVFYNSVFMQRLKTNILQYASTRPPTLSPIPHIPRSPYKFSSSPLRIPGGNIYISP

LKSPYKISEGLPTPTKMTPRSRILVSIGESFGTSEKFQKINQMVCNSDRVLKRSAEGGNPPKPLKKLRFDIEGSDEADGS

KHLPAESKFQQKLAEMTSTRTRMQKQKLNDSMEISNKEEK

>Rbl1-Rn gi|109469134|ref|XP_001055763.1| PREDICTED: similar to Retinoblastoma-like protein 1 (107 kDa retinoblastoma-associated protein) (PRB1) (P107) [Rattus norvegicus]

MFEDEPHAEGAAAVAAAREALQALCQELNLDEGSAAEALDDFTAIRGNYSLEGEVIHWLACSLYVACRKSIIPTVGKGVM

EGNCVSLTRILRSAKLSLIQFFSKMKKWMDMSNLPQEFRERIERLERNFEVSTVIFKKFEPIFLDIFQNPYEELPKLPRS

RKQRRIPCSVKDLFNFCWTLFVYTKGNFRMIGDDLVNSYHLLLCCLDLIFANAIMCPNRRDLLNPSFKGLPSDFHAVNFK

AAEEPPCIIAVLCDLHDGLLVEAKGIKEHYFKPYISKLFDKKILKGECLLDLSSFTDNSKAVNKEYEEYVLTVGDFDERI

FLGADAEEEIGTPRKFAADTQFGKLTSQASVDCNLQQHFEKKRSFAPSTPLTGRRYLQEKEAVTTPVASATQSVSRLQSI

VAGLKSAPSEQLLTIFESCMRNPMGNIVKIVKGIGETFCQHYTQSTDKQPGSHIDFAVNRLKLAEILYYKILETIMVQET

RRLHGMDMSVLLEQDIFHRSLLACCLEIVLFAYSSPRTFPWIIDVLGLQPFYFYKVIEVVIRSEEGLSRDMVKHLNSIEE

QILESLAWTNNSALWEALRASANKVPSCEEVIFPNNFEIGNGGSVQGHLPMMPMSPIIHPRVKEVRTDSGSLRKDMQPLS

PISVHERYSSPAAGSAKRRLFGDDPPKETLMEKIMAEGTQLKIAPSSVTAESLSISPGQALLTMATTTVTGTTGRKVTVP

LHGIANDAGEITLVPISMNTTQDSTAESLVSLTAQSLIGASPKQTHLTKTQDAPLTGISKPKRTGSLALFYRKVYHLASV

RLRDLCLKLDVSNELRRKIWTCFEFTLVHCPDLMKDRHLDQLLLCAFYIMAKVTKEERTFQEIMKSYRNQPQANSHVYRS

VLLKSIPGEVVAYNGDYEMTDGDIEDATKTPNCSSEPVKEERGDLIKFYNAIYVGRVKSFALKYDLSNQDHIMDAPPLSP

FPHIKQQPGSPRRISQQHSIYVSPHKNASGLTPRSALLYKFNGSPSKSRKVLRNMVNNGEKRAKKRVIAISGDAESPAKR

LCQENDDVLLKRLQDVVSERANH

>Rbl2-Rn gi|13592041|ref|NP_112356.1| retinoblastoma-like 2 [Rattus norvegicus]

MASGGNQSSPPPPAAAASSEEEEEDGDTADRAQPAGSPSHQIQQRFEELCSRLNMDEAARAEAWSSYRSMSESYTLEGND

LHWLACALYVACRKSVPTVSKGTAEGNYVSLTRILRCSEQSLIEFFNKMKKWEDMANLPPHFRERTERLERNFTVSAVIF

KKYEPIFQDIFKYPQEEQPRQQRGRKQRRQPCTTSEIFHFCWVLFIYAKGNFPMISDDLVNSYHLLLCALDLVYGNALQC

SNRKELVNPNFKGVSEDGHPRDSHPSSDPPCVIEKLCSLHDGLVLEAKGIKQHFWKPYIRKLFEKKLLRGKEENLTGFLE

PGNFAESFKAVNKAYEEYVLATGSLDERIFLGEDAEEEVGTFSRCVSAASGTESAERTQMRDILQQHLDKSKTLRVCNPL

TGVRYVQENSPCVTPVSTATHSLNRLHTMLAGLRNAPSEKLEQILRSCSRDPTRAIADRLREMYEIYSQHFQPDENVSNC

AKEMANKHFRFAEMLYYKVLESVIEQEQKRLGDMDLSGVLEQDAFHKSLLACCLEVVAFSYKPPGNFPFIAEIFDVPHYH

FYKVIEVFIRAEDGLCREVVKHLNQIEEQILDHLAWKTKSPLWDRIRDNENRVPTCEEVTPPHNLERTDEIYIAGSPLTP

RRVGEVRTDAGGLGRSVTSPTTLYDRYSSPTVSTTRRRLFESDSPSEGSTAGRIPPQPLVNAVPVQNVSGETVSVTPVPG

QTLVTMATATVTANNGQTVTIPVQGIANENGGITFFPVQVNVGGQAQAVTGSIQPLSAQALAGSLSSQQVTGTTLQVPGP

VAIQQISPGGQQQNQGQPLTSSSIRPRKTSSLSLFFRKVYYLAGVRLRDLCTKLDISDELRKKIWTCFEFSIVQCPELMM

DRHLDQLLMCAIYVMAKVTKEDKSFQNIMRCYRTQPQARSQVYRSVLIKGKRRNSGSCENRSHQNSPTELNTDRASRDSS

PVMRSNSTLPVPQPSSAPPTPTRLTGANSDIEEEERGDLIQFYNNIYRKQIQTFAMKYSQANSQMDTPPLSPYPFVRTGS

PRRVQLSQSHPIYISPHKNEAMLSPREKIFYYFSNSPSKRLREINSMIRTGETPTKKRGILLDDGSESPAKRICPENHSA

LLRRLQDVANDRGSH

>Rb1-Gg gi|45383327|ref|NP_989750.1| retinoblastoma 1 (including osteosarcoma) [Gallus gallus]

MPPKPLRRAGAARSQRTSPEGGAGTASPPGGTRLEVGEAEFVALCDALKAPDSVREKAWMTYQSLAAADGASAYNKKKKE

TWGVCIFIVAIDLDEMTFTFTELLKSLSISVCTFFQFLKEVDVNMDTVSTKVDSTVSRLKKKYDVLLALYHKFERTCGLI

YLEQPSSEISAELSSVLVLKNYWITFLLAKGKVLQMEDDLVISFQLLLCVLDYFIKLSPPAMLKEPYKSAVTALTVNGST

RTPRRGQNRNARASKQIDTDTKVIEILCKEHDCNLDEVKNVYFTSFIPFLNSLGVVASNGLPEVDVLSKQYDELYLKNKD

IDARLFLDHDETLQPDVIACSQLERTPRKNNPDEEVNHVLPQTPVRAAMNTIQQLMMILNSATDKPSDTLIAYFNNCTVN

PEDSILKRVESLGHIFKKKFAEAVGQGCAEIGSQRYQLGVRLYYRVMESMLKSEEERLSVHNFSKLLNDNIFHTSLLACA

LEIVMATYGRTASQSDGTSAETDLSFPWILNVFDLKAFDFYKVIESFIKVEPSLTRDMIKHLERCEHRIMESLAWQSDSP

LFDLIKQSKEREGQTDQPEPTSTLNLPLQHNHTAADLYLSPVRSPKKKASGHPQSGTSNPDAQPSATSQTQKPQKSTSLS

LFYKKVFRLAYLRLHTLFFRLLSEHPDLEPLIWTLFQHTLQNESELMRDRHLDQIMMCSMYGICKVKNVDLRFKTIVSAY

KELPNTNQETFKRVLIREEQYDSIIVFYNLVFMQKLKTNILQYASNRPPTLSPIPHIPRSPYQFSNSPRRVPAGNNIYIS

PLKSPYKFSDGFHSPTKMTPRSRILVSIGETFGTSEKFQKINQMVCNSESHVKRSAEPSDAPKPLKRLRFDIEGQDEADG

GKHLPQESKFQQKLAEMTSTRTRMQKQKLNDGNDTSANEEK

>Rbl1-Gg gi|118100471|ref|XP_417312.2| PREDICTED: similar to retinoblastoma-like protein 1 [Gallus gallus]

MSRAEPLPEPQPELELGAAIERLCRELNLDAGSAAEALRDFTALRGTYSLEGDAQHWLACALYAACRRSVLPTVGSGVME

GNCVSLTRILRSARLSLIQFFSKMKKWMDMSNVPQEFRERVERLERNFEVSTVIFRKFEPIFLDIFQNPNEETSKPQRSR

KQRRVLCGVKDLFNFCWTLFVYTKGNFRMIGDDLVNSYHLLLCCLDLVFANAILCPNRRDLLNPSFKGLPADFHAPEMKA

SEDPPCIIATLCELHDGLLVEAKGIKEHYFKPYISKLFDRKILKGECLLDLCNFTENNKALNKEYEEYVLTVGDFDERVF

LGADAEEEIGTPRKFPADLQVGKTAARAHVECHLQQHFEKKRSFAPSTPLTGRRYLREKEAVITPVASATQSVSRLQNIV

AGLKNAPSELLMAIFESCARNPAESIVNRVREIGETFCRSYTQPTDELPGSHIDFAVNRLKLAEILYYKILETIMVQEMR

RLHGKDMTALLEQDVFHRSLMACCLEIVLFAYSSPRTFPWIIEVLDLRPFYFYKVIEVLIRSEEGLSRDMVKHLNSIEEQ

ILESLAWTRDSALWTALQASENRVPTCEEVIFPNNFEASNGGSGLGHLPMMPISPLVHPRVKEVRTDLGGSLRRDTQPLS

PISVHERYSSPTAGSAKRRLFGDDGPKEMQMEKILTEGTKLTIAPASSIAAENVSISPGQTVLTVTTATVAGKPGQKVTI

PLHGIAGELGGITLIPIAMNLGQAPKIEAQDPCHPQVNQDQEVHLSSGNKPKKTGSLALFYRKVYHLASVRLRDLCLKLD

VSNDLRRKIWTCFEFTLVHCADLMKDRHLDQLLLCAFYIMAKVTKEERTFQDIMKSYRNQPQANSHVYRSVLLRNTSANV

LLDRNANQDVQMTEDSSVKTGNSLGRSAAENSTELGTEERGDLIKFYNAVYVGRVKSFALKYDITNQDHMEAPPLSPFPS

IKQQPVSPRRISQQHSVYVSPHKNGACLTPRTALLYKFNGSPSKSLKDINNMIKQGEHRSKKRAITIDSDTESPMKRLCQ

ENDDVLLKRLQDVVSERANH

>Rbl2-Gg gi|118123617|ref|XP_414087.2| PREDICTED: similar to Retinoblastoma-like 2 (p130), partial [Gallus gallus]

LIEFFNKMKKWEDMANLPSQFRERTERLERNFTVSAVIFKKYEPIFQDIFRYPQEDQPRQQRGRKQRRQPCTVTEVFQFC

WVLFVHAKGNFPMISDDLVNSYHLLLCALDLVYGNALQCPNHKELLNPNFKGLPEDFHSKDYKVSSDPPCIIEKLCSLHY

GLVLEAKGIKEHFWKPYIRKLFDKKLLKGKDENLTGFLDPGNFGDSFKAINKAYEEYVLSVGNLDERIFLGEDADEEIGT

LTRCLNTPSGMETAERVQVKHNLQQHFDRSKSPRVTTPLTGRKYIKESNPYVTPVSIATYSLSRLHTMLAGLKNAPSENL

EQIFRACSRDPSQSIASRVKEMYEVYCQSMQAEEEFSNLSKDVASKHFRRAEVLYYKVLESVIEQERRRLGDTDLSAVLE

QDVFHRSLLACCLEIVTFTYKLPGSFPFITEIFDIPVYHFYKVIEVFIRAEDGLCREVVKHLNHIEEQILESMAWKQESI

LWDRIRDNDNKVPSCEEVMPPQYFERSAANSVVGSPLTPRRLNEVRAESGGLGKGISSSPTTLYDRYSSPTAHPTRRRLF

VDNDNTSDNGTPVRVSQQPVVNTVPVQNVNPEAVSVTPVPGQTLVTVATATVTANNGQTVTIPVQGIANENGGITFFPVQ

VNVGAQPQAVSGSIQPLSAQALTGTLSTQMSGATLQLPGQVTVQHITSGEPRPAQQLTATTATRSRKMGSLSLFFRKVYH

LASVRLRDLSVKLDISDELRKKVWTCFEYSLMHCPEIMMDRHLDQLLMCAIYVMAKVTKEDRSFQNIMRCYRTQPQAKSH

VYRSVLIKGRRRRRQSGSSDSSSQQNSPTGRSKERTSRDSSPVMRSSSTLPVPHPSSAPPTPTRLTGANSDTEEEERGDL

IQFYNSIYIEQIKEFALKYTSSGTDTPPLSPYPSVRISSPRRVQLSQNHAVYISPHKNVSALSHREKIFYYFSSSPSKVR

LKGLSVSRSKQLESNEVSK

>Rbl1-Xt gi|213982839|ref|NP_001135587.1| hypothetical protein LOC100216140 [Xenopus (Silurana) tropicalis]

MQREETDTEASNSSDGGPGEAVQEQLETLCRELNLDQETGAETIRDFTRTWSTYSLEGEVKHWLACALYAACRKGVIPTV

GKGIMEGNGVSLTRILRSAKLSLIQFFNKMKKWIDMSNLPQEFRERVERLERNFEVATVIFKKFEPIFREIFQNPHEEPP

RLPRSRKQRRLQCSAKDLFSFCWTLFVYSKGNFCLIGDDLVNSYHLLLCCLDLIYAHVLQCPNKEDLLNPSFTAKLPALD

PSSEDAPCIIRALCELYDGIFVEAKGIKEHYFKPYISKLYDKKILKGACLLDVSSFTDNNKSLNKEYEEYVLTKGDFDER

VFLGVDAEEEIGSPRKFVVEFPMGICTPRKQMESNLQQHFERKRSFAPSTPLTGRGYLKEEPTVITPVSSATQCVSRLQS

MVAGLRNAPSEQLIEVFKSCVRNPLENVLNIVRDIGRRFCHHYTQSTEDQPGSHIDFAVNRWKLAEILYFKVLETIVVQE

SKRLHGKDLTALLEQDIFHQSLIACCLEIVLFAYSSPRTFPWTIEVLNLSAFYFYKVIEVFIRSEDGLSRDMVKHLNTIE

EQILESRAWTSDSVLWVHLDAVDNVPTCEEVIIPSNFETGNEAGVGHLPMMPASPIVHPRLKEVRTDLGGSFRRDCQPLS

PISVHDRYSSPAAGSAKRRLFGDDCPREQTVDKITTPDITRIRIALSPSPDKTAQSIGQTIAIPVQGLANESVKSLFLPM

KTSPPQSENYVPVPLTAQALISPLTKRQLGRMPESCPTSARKPKKTGSLALFFRKVYHLASVRLRDLCLKLDVSNDLRRK

IWTLFVYSLVHCTDLMKDRHLDQLLLCAVYIMAKISKEERLFHDIMKCYRNQPQANSHVYRSVLLKRIPQASGDETSTSQ

DVEMTARNSSDSHPTPSGFTGSPCGNAERGDLITFYNNVYVERVRSFALKFASTQDHLMDAPPLSPFPSIKQQAISPRRV

SQQHSIYVSPHKNSGSLTPHTALLYKFNGSPSKSLQDINSMLKQEQRSRKRIIAIDSDTDSPAKRLCQENDDALLKRLQD

VVSERANH

>Rbl2-Xt ENSXETP00000000730 [Xenopus (Silurana) tropicalis]

GNYVSTSSSSNKKKWDMANTRTRTKKRNTVSAVKKYDVRSRHRGRKSYRRCTVSVYCWVHTKGNMSDDVNSYHCADVYGN

ACNRRNNDCSRYKAACTCGHDGVAKGKHWKYRKDKKKGKSDSVGDGNADSVKANKAYGYVSAGNDRVMDVGRGCNSGMSV

RAKVTYNNDKKSRVSTTSKYRCSCTTSSAMYCVSRTTGKASRMRSCSRDTKTRRKMSTCNTDSVAKCRDSDTKKYRAVYY

KVSVDKKRGDDMSSDVHRSMSCCTVSYKGNSVMAYYKVVVKAGCRVVKHNNCMAWTHDSWDKRNDGKCVVNHTGSHCCDK

MAAHYYNSAGVSSATCDRYSSAAGTARRRVDDSSSAGSAASATDTVATVGTVTMATATVTANNGTVTVGANNGGTVVNVS

SHSVTSSVTAAAGNGVASGSKNTSKKKASSTCRRVRHKASRKDCKDSDRRKWTCSVHCKDRHDMCAVYVTKVTKDTSHNM

KCYRVHASSHVCDSCKTRCSRSVMRSSSTVNSATTRTAAGSNVRGDVYNNYKKSAMRSSNTKSHNATSYSRAVSRRSHHS

YVSTHNDDATTVCRKCYYDSAKRNSMRTGTTKKRGDGSSAKRCNHSARRDVANDRGTH

>Rb1-Dr gi|118150572|ref|NP_001071248.1| retinoblastoma 1 [Danio rerio]

MPPKKRSSGTPQKKELKGSLKSRSPDSGDNAVLSPERHKDKDPEFVFLSEELQSTNSICDHAWRIWEREIRSMDKTNMPY

SNRQQWGACLFIAGMELEGINLTFTQFLKAVGLSVKQFISLVRKMDVNVDTISPKVNSAVTRLENKYDVTLALYQRFVKT

CEKIFAEPDNAKRKELWESSWTMFLLAKGTFLQMEDDLVISFQLLLCVLEFFAKRLSPSLLQSPYNSVVSSSTLSPPTRT

SRRNQGKSKPRPAEMDMQLLETLCKEGDCSVDEVKNVYQSTFCAFLDSVGLLGLQGLPPMEALSKQYEELYHKSKDFDAR

LFLSDDETLSPNKIEVSKVEVTPRKNLFAEDIAIPVPQTPIRAAMTSIQQLRGDLTSGSDQPSSNLLVYYKNCTVDPSGE

IKKRVEELGEVFIQRFAQAVGQHCEGLGRKRFYLGAQLYYKVMESMLKSEEKRLSVQNFSKLLNNAAFHTSLLACALEVV

IATYVGSSLKNGGFGRSSGASDSVESDLCFPWILSVFQLPAFDFYKVIESFIKAEPTLKHDMVKHLEQCEHVIMESLAWR

TDSPLFDLLKQSREEGPGEQAEPPATLNQPLHHNHTAADLYLSPVRPCRQPPVMEAEPPTPGTRAPRSNSLSLFYKKLYR

MAYLRLKMLFSNLLTSHPEMEPIIWTLLQHTLQNEYELMRDRHLDQLIMSAMYAICKVKNVDLRFKTIVTAYKELPNTNQ

ETFKRVLIREGQYDSIIVFYNLVFMQKLKTNILQYSSPRPPPLSPIPHIPCSPYKNSPLRVPGSNNVYVSPLKSSRVSPL

VMTPRSRILISIGESFGSADKFQKINQMLSSSDWSLKRSLDGGSAPKPLKRLRFDMDGQDEADGSKSSGESALIQKLAEM

SSTRSRMQEQKLKEESDKDHPEP

>Rbl1-Dr gi|194578849|ref|NP_001124082.1| retinoblastoma-like 1 (p107) [Danio rerio]

MRGDETDTESVRSADGSIRSNFEALCQELNMDEETAAEALQNFRSIWNTYTLEGDVMHWLACSLYAACRKSSIPTVGRGV

MEGNCVSLTRILRSAKLSLIQFFSKMKKWSDMANLCQDFRSRIGRLERNFEVSTVIFRKFEPIFLDMFQNPQSEPPRMPR

SRKHRRLPCHISDVFKFCWTLFVYTKGNFHMIGDDLVNSYHLLLCCLDLVFCNALMCSNRKDLINQNFRGLPKDTENLKE

MPCVIDKLCELHDGLVVEAKGIKEHYFKPYIKTLFEKRMLKGDVETLTELLDTPNFQDNNKAINRDYEEFVLTVGDFDER

VFLGADADEEIGTPRKSPAEPPSGHLSARMQVENNLQQHFEKTRSLAPSTPLTGRRYLKEKEVLVTPVSSATQSVSRLQS

MVSGLRNAPSDALLQIFNSCSRNPTESILNRVKTLGEKFKQAYTNPTDDLPGAHMDFAEKRLKLAEILYFKILENVMTQE

MKRLQGKDWAVLLEQEVLHCSLLACCLEVVLFSYSSQRTFPWILEIFQIPPFYFYKVIEVFIRSEEGLSRDMVKHLNSIE

EQVLESRAWTRDSALWNALNKDNNKVPTVEEVNFPSNFDTGNNAGGPTHLPLVALSPIIHPRLREVRTGLGSSARKDVPQ

SPISLHDRYSSPAAGSAKRRLFGDDAPPQSPVKRISVTPIKIIPSGMENQTTTTTVLSMATGNGQQLTIPLPVVKNEAGG

ITVIQLQASDMNPLTAQVLLTASPSRPALAAPAISSDSQPPPANKPRRTGSLALFFRKVYHLASVRLRDLCLKLDISSEL

RGKIWTCFEHSLLHCTDLMKDRHLDQLLLCAVYIISRITKEEHTFQDIMKCYRTQPQANSHVYRSVLIKRRPKVQQADEN

MEVDPPADQSNERTDQAANERTDQADRTQTVFESEERGDLIQFYNNIYVSKMRSFAFKYALSTLDNRMEAPPLSPFPSVR

SHTLSPRRVSQKHSIFVSPHKNSSSLTPSTAYTYRFTGSPSKELSDINQMIRQGGVSKKRAFTMEGDETESPSKCLRQEN

DDVLLKRLQDVVSERASL

>Rbl2-Dr gi|189537876|ref|XP_001922168.1| PREDICTED: similar to Retinoblastoma-like protein 2 (130 kDa retinoblastoma-associated protein) (p130) (PRB2) (RBR-2) [Danio rerio]

MLHTYTLICCDVWSLSGIVVSSSLIEFFSKMKKWQDMANLPRDFQQSTEKLERNFTVTSVIFKKYLPLFRDIFKTPTDEL

PRTHRGRKQRRHPCTVTEVFNFCWILFIHAKGNFPMISDDLVNSYHLLLCALDLVYCNALLCSSRKDLLNPAFKGLPEDF

NSKDYKPGPGSLCFIEQLCELHDGLVLEAKGAKEHFWKPFIKKLFERKILKGKEDTLTGFLEPVNFGDSLASLNRVYEEH

VLSSGSLDERIFLGDGAREDIGTPGPYEGIENQDRANNPLQNSMTALDVSVSQASALRVSTPLTGRRYVPESIVGTPVSS

AMQSVGRLHTLLTGFKYRPSVKLRDIFRSCVRDPTESVAARLKEMSEIFLQNYEGTGDDNKSLARGLQSLHLYISHICSL

RNMSSHVSQGVLEQDVFHRSLIACCLEIVIFSYRPPGEFPRVLQIFDLPAYHFYKVIEVLVRAEEGLFREVVKHLNHVEE

QVLESLAWKGNSPLWERIREAKNCVPTCQEVMPPQHLEDGSGSNANTPSSKTEFSGNIAAGAGKGVSPSPTSLHDRYSSP

PTGTAVRRLFVDGDTTPEPAPPVKVAPPSIVSSIPAGQTVVTMATATVTANNGQTVTIPVQGIANESGGITFIPVQVSVT

SQAGATLPTLSAQTLTGTLTTQQLTTTPMGAVQNNPSPPKQDKKTPQQGTPTKRAQKTGSLCLFFRKVYHLASVRLRDLC

AKLDISADLRRKIWTCFEYSLVHCTDLMMDRHLDQLLMCAIYVMTKVTKEDKSFQNIMKCYRSQPQASSSVYRSVLISGR

KRRHSGNTENTHRQSSPTEGAQEQASGESSPVSMRSSSTLPIPQPSSAPSTPTRAPGGPQEQEEERGDLIRFYNHVYIKQ

IKPFALRYSSNSPKNGQAETPPLCPYPSLRIGSPRRVLLSHNHSIYISPHKTGSPISPRDKIFYYVSSSPSHRLREINTM

IRTGETPTKKRSIALEEEQQSPAKRLCQENQTALLRRLQDVANDRSSSH

>Rb1-Tn ENSTNIT00000014938 [Tetraodon nigroviridis]

MPPKKRNTVVSQSKEPKPSAASVSPDKKEIAELSFKKHRDKDTEFVTLCKSLHVTDLVCDNAWMLWKTIQDSVEELNDSQ

KSLWGSCLFASVTDMDIACFTLTQLLKAVSLNVKEFLALVRKLDVNMDTISTKVNAALSSLEKKYDVMLALYQRFEKTCK

SVFASVSDKEKEIIRSCWTMFLLAKGRALQMEDDLVISFQLLLCTLEFCIKRCPPELLQPLYKSAISKVHNPPARAARRS

QSKAKSRPPESEVDLQLLKTLCKENDCNSEEVKNVYQTSFSAFLDSLDLSGAPHFPQGKGLDQQYEQHYLRSRDFDGRLF

LDGDATVLAPKVDLSQVERTPKKMSEEDNTLIPPQTPIRAAMTSIQQLRGDLTSSGDQPSTHLATYFKNCTVDPTQDVMK

RLETLGEAFSQRFGQAVGPHCVTLGRQRFNLGVKLYYKIMEAMLKSEEKRLSVQNFSKLLNDSTFHTSLLACALEVVMAT

YGESSFKTGGYNQGSGDAAERNMCFPWILDVVNLAAFDFYKVIESFIKADPTLSKDIVKHLETCENLIMERIAWRTGSPL

FELLRQEHEGGTAEQVETPASFSQPLQHNHTAADLYLSPVRPGLRVLPPETSPTSTTQASSQPPAQPASQPARPLKSNSL

SLFYKKLYRLAYTRLKMLCSHLLFSHPELEPIIWTLFQHTLQHEHELMRDRHLDQLMMCAMYAICKVKIVDLRFKSIVTA

YKNMPNTSQDLTDFQASVLTTEGNYDSIIGFYNLVFIAEVKNATSMQDLTVPQHSSPIPQIPRSPYKFPNSPLRVPVSSN

VYISPMKSPRMSPGIMTPRSRMLVSIGESFGVSNRFQKINQMVNSGDRSFKRSLDLGSTPKPLKRLRFDMDGQDEADGSK

SGGDSALIQKLAEMTSTRSRVQEQKMKEDAESRREKP

>Rbl1-Tn ENSTNIT00000009683 [Tetraodon nigroviridis]

SLDQLCQELNMDEQTATEAMRNFTAIWNTHTLEGEVVHWLACSLYAACRKGSTPTVGKGLMEGNCVSLTRILRSSRLSLI

QFFSKMRKWADMSNLSQDFRLRLKQLERNFEVSMVIFRKFEHIFVDMFQNPQGEEPPRKSRTRKHRRLPCHTSDVFRFCW

TLFVYAKGNFCMIGDDLVNSYHLLLCCLDLVFGNALLCSNRKELVNPSFKGTASGYPADGHAALDQPPCVLERLCELHDG

LVVEAKGIKQHYFRPYIQKLFEKKILKGKEEHLTQLLDPQNFLENNKALNKEYEEYVLTVGDFDERVFLGADADEEIGTP

RKLVGDSSACQKNAQQHLEKSASLVPSTPLTGHAYLKENNTLGTPISSATQSVSRLQSMVAGLRTAPSENLMQIFRSCSR

NPSEAITARVRRLGQTFKEHYSLDSEDTPASHIDFAEKRLKLAEILYYKVLENVMVQETKRLHGKDMSILLEQDIFHCSL

MACCLEVVLFSYSSQRTFPWIINVFKLASFYFFKVIEVFIRSEEGLSRDMVKHLNQIEEQVLESQAWSRDSALWGALQAA

GSKVPSVEEVNFSSSLDSATAGCQSPLPLLTLSPIIHPRIREFRSGLSGARKGGLMCLLLRLSVHERYSSPAAGSAKRPL

FGEEHPPPGLAHGLALISPAKRLMIGPSSTLKISAPGSAPALAVPLQGRGPSGAGVAPPGENAPNFSRAILPEPINAQFL

LTASLNPTLPPEPEAQAGRPRRTGSLALFFRKVYHLASVRLRDLCWKLDISAELRARIWTCLEHTLIHCTELMRGRHLDQ

LLLCSVYIMSKVTKETHFFHDIMKCYRSQPQASSHVYRNVLLRHSPGECVSEEKMEVDAAPAADGDECLQRLGRCAPAGA

GSSQSGEEQRGDLIQFYNTVFVLKVKNFAVRYAVSDSEGGAPPLSPFPSLRAQPLSPRRVSQRHSLYVSPHKSSSGGPAL

NSYSYRINSSPSDELTAINRIIQQGCVSRKRVFASDGDELPSASPSPSKRACSQGSSSPDVLLKRLQDVVSERQSL

>Rbl-Ci gi|198437827|ref|XP_002124769.1| PREDICTED: similar to predicted protein [Ciona intestinalis]

MSSGDESDEDEQRFRDLCLAVNMDKPTMESAWRSYENAKENYTLEGDCLHWLTCTLYSACRTNLSSVKTIAGKTAPSNCI

SLTKLLREAKLSFISFLKKMDKWLQMNQNDPLSSHVSALERSFHVSTVVFKKYQVVFKYVFLDPDDSKYQRPVRGSAKNP

SRPRRNRQYRKLVCNPHDVFNFCWTLFVHARATEFRSISDEIVLSYQLLLSAINLCYNNALNCSVDHRAQLLNPKFPGLP

ADWSTRTEPLTQNQDIIALLVEQQPGTGQLDEKFLIETRVVNVHNWGKHVNSLLESEGLGLSGEQNSLFSPQNFETNSSA

ISKLYEEYVITEGDFDERVFLHDEVDSELGTPIKCPPPAQTNLKQEVAEHLAKTNSMVQKTPLTNREYLHGKGHNSSLNG

SLTPVSSTCHDVMKLKKLLQSKTDGPSEKLAAIFRECTEDPSEEVKKRTDRLREIIKKKYMERGGKGGGIPAGTPAIAEQ

RATMATVLYYHILEGSMIQEKKRLNGKSDLSELLKRDDFHTLLFACCVEIVLCSYKAERMFPWILSALDLSPYYFYKVIE

LVIRAEDGLWGPCIKHLNHIEEQILESLAWKSDSTLWQVLGNGESPAPLCEEVNHSQHIEPSSADKSTGVNKGLLGPGTP

KSPPTHPRVKAVSEGEIGNLRKFEPGTARDRFSSPSPGSAKRRLFVKDHLSSTPGTLKVVSMDVPNSPGAKTYVVQQTSP

GGKPITVVSVANHADNVKPRKTGSLALFFRKVYHLASVRLRHLCEQLVIPPDLRAKIWTCFEYSVSKHAYSLMRDRHIDQ

MLMCAVYVIAKITQHDHSFQEIMRCYRSQPQATSNVYRNVLIHRSSSPRLIPAQPPSRSMRSNSTIPRSDPSSPLPGSTG

GGDGERDDLIQFYNKVYVPVMREYVKKFNSGDVSHLEVPLSPMPRPRILPQSPRKVTDNIYISPLKNPSLLMATNTDRHL

DYNFHRSPAKRLRDINSMMQRHAPTLNGKRTLEFSKDEDGSEPKRANVSRRVQLMQRERSLPN

>Rb-Sp gi|115660767|ref|XP_790323.2| PREDICTED: similar to p104 chicken Rb, partial [Strongylocentrotus purpuratus]

MDESVANVLSHLASPVNASVRDQAEKLLQIWTASDRVEFRENESTWAVCAIYIATVNVKYGKGAPVSSGHEAPESSSLPT

VSHLLRASNINIKTFFEHMGKVEEIFSFSDTVKSSLMALKQKYLITYAIFCKFEKLFSKLFKSPDEIPCSTDGSCDVSCD

KRKVCWLLFLIAKGSMLSECSELVLPLHLMLSCVDYTLRRALPFTVRETVSSPRYDSGSEDKTNERVLRVVCEEGGVGGA

DAEDEVTMVKRSILLPFINSISSENTNQDVEGFPKMEHLESSYHQMYQAAGDINEVSFLTLDPTLTQPEAVKPGVPPSPD

STVKSPVFLTPVRRAVTTVQALKSLLTGARDTPSNVLCSYFEGCSINPQSAVEQRVEKLKELFVGHYVGEVGVQASELAC

TRFKLGLRFYYRIMESMLIKEEKRLSTSDFSTLLNNDAFHRSLLACALEVVMVTFGYTASPINSGMSALSSKLLFPWILD

VFDLHSFNFIKVIESFVKNEPRLTEEARKHLQGIETQIVECLAWSVNSPLFDALKRVRGIEVNGSHTQSPSSHGVSHASA

ADMFLSPVRPGHYSSTHRPNEATPPRRTHAPTCASRLEYPSPKSSRLGIQQCPSPRVGLQQTCPSPRPSYGGVGGGQEKL

GSVTLNMFFNKALQLAYHRLHTMCNLLDLSKDLNRLMWTCVEHCITNKPWLLQGRHLDQVIMCCMYGICKVRDCERKFKE

IVNMYRGLPHALSQTYKNVYMGEGKESASIIVFYNQIFILHTKEYIHKFQLSIS

>RBL-Sp gi|115968799|ref|XP_001194030.1| PREDICTED: hypothetical protein, partial [Strongylocentrotus purpuratus]

ALDEGNAGSPQDFAKRRLKLAENLYYKVLENIMLAEKRRLKNLTHLLEHELFHRSLFACCLEVVIFAYNSQRTFPWILEA

FEIPAFHFYKVIELILRAEEGLSRDIVKHLNFIEEAILQDYAWRSDSPLWDLLQESNQGVPTCEDATIPGQTDGGVAGGA

GGGGGGGKGATTSQATSSSGTTNSSQGQSLLSPIVHKRVVQLGGRTEAILQSPTSAHDRFSSPSPGSARRRLFTADAPTT

SSSTSTSSSPTETTIKITSSSSSSVTRAMTFTDVQPTTGTTSSPTTSATSTSNSTVVVSPRKQQFVVVHGIQGKDSTGNA

RLIVSPVKVIYTSSPAKAKTAEGEAGSSGTTAVPVPRPKRTGSIGIFFRKVYHLMNVRLLDLCHRLKLSDDLRRKIWTCL

EHAVVHKTEMMRDRHLDQIIMCTMYIICRVTQNPCSFQEIMRSYRFQPQASSHVYRSVLIQSNRKKDANGNGSVNSSRGS

PEPKTMETPILMRSASTLPVPQPSSAPPTPTRMAGTASTFDDEHRGDLIKFYNSVFLQSIQAFALRFENSPNGVTERLEA

PPLSPLPVPRPHMLSPRKVSNRHQLYVSPHKHMQSPKSGMTYVIQKSPAKVLHNMNNMVKARECTKRQLLSESLESSKRL

CVDNRNDFLQTRLATVKSERESASS

>Rb-Bf gi|260800656|ref|XP_002595214.1| hypothetical protein BRAFLDRAFT_135823 [Branchiostoma floridae]

SNLLNKDTFHRSLLACSVEVIMTTYGSASINTYFISGTPMLYTMATTDTNTSFPWILDVFELKAYDFFKVLETFILAEPK

LTKEVIKHLQHIENKILERVAWREGSPLYEAILTSEMPRPSFQTSPGSRVTNGNIPHSSTASEMYLSPTRFSAAQQSPYR

SPARLHCRSPGPHRRKSQSLQLFYNKVCKLAYMRLQSLCKLLCVSAEMEHRIWTALEFCLLHRPELLKNRHLDQVIMCSV

YAICKVSGCDTKFKSIVAAYKNLPTASQTVYKEAWISGEEYDSIIGFYNRVYMQTLKNYILRFAS

>Rbl-Bf gi|260834765|ref|XP_002612380.1| hypothetical protein BRAFLDRAFT_218889 [Branchiostoma floridae]

MAGPDEETPSPEQRYQDLCMDLNMDKIATEEAWQSFEKMSTNYTLEGDDLHWLACALYVACRKSLTPTVGRGTVEGNCVS

LTRLLRSAKLSLIQFFNKMKKWADMASLPQDFRDKVDRLERNFAVSTVIFKKFEPIFLDLFRNPGDDQPRAPRGRKQRRL

PCTSSDVFSFCWTLFVQVKGNFPAISDDLVNSYHLLLCCLDLFFGNALCAGRKDLLNTEFPALPEGFTNRDWRSPSEPPC

IIDKLCELHDGLVLEAKGIKEHWWKPYIKKLFDKKTLTGLLDLATFEINNKSINKEYEEWVLTVGDFDERIFLGEDADVE

IGTPAKPAPNITGELGERMVQSSHLKQHMAETKSLAPSTPLTGRRYLKEKDPTVTPVSTATQSVSRLQALLAGLKTSPSD

NLTKLFSECSRNPQESISSRIKEMGEQFCTSYTQPSEDHPGSHIEFAQKRLRLAETLYYKVLENVMLQEKKRLKGKLDLV

SLMEQDVFHRSLMACCLEIIIFSYNSQRTFPWIIDVFGLSPYHFYKVIEVLIRAEDGLSRDVVKHLNFIEEQILESLAWK

SESPLWEAMKMNENAVPSCEEVTLPSQLENGHSNTAVRMGQSPPVHPQVRRIARDTGVMLKKVESPLSPLSAHDRFSSPA

AGSLGTAKRRLFSNDQTNNAATTTSAQSQPATVQVVTTATDQGVQQTITQQTQIQLQAVPHGQQVPGAGVLGAMAQAVAT

GQQQAAPSQTAAVPHGTAAAAAPASGEKAAAGEQHPHKRIGSLGLFFRKVYHLASVRLRDLCEKLEVGDELRRKMWTCFT

HTLKEHVELMRDRHLDQIVMCSIYVMAKVTQHDKSFQDIMKCYRTQPQASSHVSLTFLNRYLNHKHSIDNQHFSRMYLQQ

ELCISSAPPTPTRLAGLGATVDGDERGDLIQFYNTIFVTKLKSYVRKFSPTGDQKVGLEQKNLSPIPLPRTHMQSPRRVS

VRHPIYISPHKNGRPLTPTSAMLYCFGKSPAKNLQDITSMIKSGEQPTKKRALNYDTNENPAKRLCSAGGADRTLFQKLK

DIDTVRQAANANQ

>RBF1-Dm gi|24638969|ref|NP_525036.2| Retinoblastoma-family protein [Drosophila melanogaster]

MSEPDPQELGAEVVSGLVATSDDRLEMINAEYTNLCRDLNMDRQTELQGYETYLEVSQRCSMEGTASHWMCCAIYTACRR

TSTPTVTGQNAVVKGNCVSLNNLLRCCKMSIYEFKTKIKQWCDMANLPQEFVNEIEDLDRKFSITFMLHKRFRIIMDMIF

SCPPNEKKHSKYISLHGNHAHGKCSYIKLDDICWRLFLCAKNQKPSNTVDLVTSYNLMICCIDLIYNNVLAEKRTDLINP

KFEGLPSNWTELDFRHNPHCILSNFCDMTEEAKAMKATTFRQIMSSFFQASTIYGNKDTMLGLLANENFERNLKSLNISY

EQYVLSVGEFDERILSAYDAGEHTALNDQSLRPPVTPLTRKQDLPAQPAMAGDKFEPVRNATNNVKQLSAFGRITEPTDF

VKQAGEEVIAKLLSIIEEIEQKFLAKYPSTEAKSRFQLAKSFFFYLLDQILQAEIRNKPDIDLKRLLVQKVSLVIFNITL

MACCVELVLEAYKTELKFPWVLDCFSISAFEFQKIIEIVVRHGSHEGCLNRSLIKHLNSIEETCLERLAWARNSTVWEMI

ASAQLPLPTWLMVNLDRAAGPLQIFLRKVYLLGWLRIQKLCSELSLCEKTPESIWHIFEHSITHETELMKDRHLDQNIMC

AIYIYIRVKRMEDPKFSDIMRAYRNQPQAVNSVYREVFIDINEDGEPKVKDIIHFYNHTYVPLMRQFVIDYLNVTPDVSG

RASDLQLSPHPKERAAQPKKVTQSHSLFVSQMSKNEIQQSPNQMVYSFCRSPAKDLQAMNEKVRGGKRMLSFGDEPGLGT

MAETKRSKISQVKAVMDDPELQSAEQQTAVTTEGCVGGEGGEHET

>RBF2-Dm gi17737995|ref|NP_524372.1| Retinoblastoma-family protein 2 [Drosophila melanogaster]

METCEVEGEAETLVRRFSVSCEQLELEARIQQSALSTYHRLDAVNGLSTSEADAQEWLCCAVYSELQRSKMRDIRESINE

ANDSVAKNCCWNVSLTRLLRSFKMNVSQFLRRMEHWNWLTQNENTFQLEVEELRCRLGITSTLLRHYKHIFRSLFVHPGK

GADPGAANHYQALYEFGWLLFLVIRNELPGFAITNLINGCQVLVCTMDLLFVNALEVPRSVVIRREFSGVPKNWDTEDFN

PILLNKYSVLEALGELIPELPAKGVVQMKNAFFHKALIMLYMDHSLVGDDTHMREIIKEGMLDINLENLNRKYTNQVADI

SEMDERVLLSVQGAIETKGDSPKSPQLAFQTSSSPSHRKLSTHDLPASLPLSIIKAFPKKEDADKIVNYLDQTLEEMNRT

FTMAVKDFLDAELSGKRFRQARGLYYKYLQKILGPELVQKPQLKIGQLMKQRKLTAALLACCLELALHVHHKLVEGLRFP

FVLHCFSLDAYDFQKILELVVRYDHGFLGRELIKHLDVVEEMCLDSLIFRKSSQLWWELNQRLPRYKEVDAETEDKENFS

TGSSICLRKFYGLANRRLLLLCKSLCLVDSFPQIWHLAEHSFTLESSRLLRNRHLDQLLLCAIHLHVRLEKLHLTFSMII

QHYRRQPHFRRSAYREVSLGNGQTADIITFYNSVYVQSMGNYGRHLECAQTRKSLEESQSNVGILTETTSNELSMRANIS

ISSPPPPRVCQSGSCSSHPPSSPAASPLSLQSSPNVKRAASSNDLMREIKRPNILRRRQLSVI

>lin35-Ce gi|17508261|ref|NP_491686.1| abnormal cell LINeage family member (lin-35) [Caenorhabditis elegans]

MPKRAADEPGTSTTDPFHEQSPFDAVLAGTETTDTICEEPPAKRIDLDIKQEFNGGVQSGGLIKNESELTQMTIKQETEG

NINEARREEEDEEQDEDSRTSMPPALGEDDDYEEDDADSFIDKTNTPPPSQSFLEGCRAANLPNDIVTGAWETYNHAVQR

VSLEGSESAWQLSAIYYYLLSKGIKRRGKTIRILIQPFPVSILTIANSFDISVAEMLDKTARFVEIIHSRKIRRYQEYIR

RIQEGLAVSCVIFKKFCRIFCKIFEEIKVGSENCPSSHELFTVLWTSFLVMKSRMTVDDLISNYQLLFSILDQVYTEMCS

MKEGIVHHLNQKFVEDLLENDCTIIRALCTQFGGSVLDARHFSDHTFKKMEKTGIPSTWNFQEFRDLIMNVPKTAYENYL

LQRGSIDERIFIPSVEDFSKIFQSPDTYSVADILKVSYSGRRFRDAEFLTKISNNHCLEKLALGGKVASEKLVTQSKEQP

RVPCVEYNLELGNYPDDLESNNQSLYNRLTKIIGSWKLENSKLEEVCGTMSDSPMATILLKSDEMTNKFERTLSAELGET

INENIPKYHYNVRKELELVFLIFMEKIIVAELKKKVREEDLLNVIRREEFLDSVFCFCVELILVSNGYDRPFPWSAELCG

VHPFMFHKVIDLMITHEKQLSRQMVQHFSRIEETVIEYFSWKSDSPLWPMVVRCPFAHFQEFGEDWADKLNSYSPIKFTP

IKKPDDLRDELGRPIVPQNQTSRTLRIFLKRTYFTAARRLQDLTDRVSMGARAKSQCWSLFDYLLRNDTLIFMDRHLDQI

LLCCVFVIMKINESSMLFTEIMAQYRRQSANSLLVYRSVTVFQEQLNPENPQAVNTKETILERLEGPQKEKTTVDIIKYY

NIEFRDRIKYIIGQIDSASDEDLMEMPVATESGLMPVRVYLTHKLSIQTLPKTKHGESKQERAIANLEKSGITIAMERSG

D

>Rb-Nv gi|156399369|ref|XP_001638474.1| predicted protein [Nematostella vectensis]

MASSNEDEDTITKERFDNVCSELNMDEETAESAWSSYETIRTNYTLEGDSLHWLACALYVACRRSVVPTVDSSGTVEGNC

VSLTRLLRASKLSLIQFFSKMKKWLDMSNASSEFRKKIEHLERNFQVSTVIFNKYRPIFLDLFKNPSEDTPKAQRSRKSR

KQPCSISDVFSFCWTLYVEAKGNFPAISDDLVNSYHLLLCCLDLMYSNALAARHRRDLINTEFEGLPSDFNTRDVRGPSD

APCIVDVLCTKYQGIVLEAKSIKEHYWKPFVQKLFEKRVLRGHEESLSGILDLGNFEANWKAINKEYEEYVLSAGDFDER

IFLGEEAHIEIGTPAKLFGSSEVDECQKSGLKKNLQEHFSKTRSLAPQTPLTGRRYLKEKDPCITPVSTATQSVSRLQAM

CSGLKPTPSERLLTIFKECSRDPLQSIIDRVENLGEIFLKEYVQPSAERPNSPCSTREFALRRLKLAEILYYKVLENITL

SEKRRLQGHLDLTTLLEQDVFHRSLLACCLEIVIFSYNSQRTFPWVISILGLAPYYFYKVIESLIKAEDGLSRDVVKHLN

HIEEQVLESLAWSGDSPLWMTIEQAGGVPSCEDVTIPNHVEAAHPNQNLLASPIMHPRLQRICGEEGMPRRIMSPTSPTL

QDHFGSPGASGARRNLMANFGGAPARTVAMATASNGHFTRSAGAAQAASNSTASVSQVHPGTTPTLLITQAPASGGGFVV

NGIAQAVVLPSSTATTVQVSLSPRSGSTLATSVIPPGSPVRSPVRLTPQAAVATSHSASTTPAKPKKTGSLALFFRKVYH

MASVRLRDLCARLDVNEELRGKMWTCFEHSLMHLPEIAKDRHLDQILMCCVYVMGKVTNSELSFQNIMKCYRTQPQAASH

VYRSVMIKNRRRAPIPNTGSESSRGDSGSTRTSPIRSTSTIPSTPPPPGGSSSTENSPVLETERGDLIEFYNKVFIKTIK

NFALKFSSSDRSLESPPLSPLPIVRNQGHSPRRQVSARHPVYISPHKNGVPMTPTTRMLYCFSESPAKNLRDINHMLRQG

ENRKRALLQDDIGAPQAKRSTSEEHLQRRLTSMIEERQANASR

>RBL-Ta gi|196011866|ref|XP_002115796.1| hypothetical protein TRIADDRAFT_59859 [Trichoplax adhaerens]

MTFHGKNPPDDRPIRSEYDELCLSLNMDKDTAQTAWQRYQDIKLKFTLEGNHLHWLTCALFVAGKQSRLP

TVGEGIQVGNCISLSRLIKAANLSLIEFFNKIDKWIDMACLDEDFRGQINVIERNFEVVSVIYKNKFEPI

FQHLFQSNVNDSVPRRKLRHSKICTYQQVFSYCWSLYLLIKGKYPDIRTDLVNSYHLLLCCIDLIYANIV

VANRRDLLNPQLESDALVESESNSSPVSVIEYLCKRYEGISIEVKAIKQHLLWLYLENLFDKNHLRGNKK

TFCGLLDQGNFDYNNKFVSNSYDEFVLSSGDFDERIFLGSNAYLDIGTPAKLPKAAHTTAEARQNVTRNL

QDRFQNSTMIPQTPLTGRNFLNQKEERTPVSSATLSVGRLQALLAKWTVSPSNKLLAIFGDCSNDLLDII

QSRINELKETFYDSYTKIDDERSKSPIDFAENRWNLGIKLYYKTLESIILAEKRRNKTSGELKILLEKDV

FHRSLLACCLEITIFSYGSQKTFPWIVQVFGLSHYYFYKVIEIVIRTEHGLPREVVKHLNYIEESILDHL

AWVSGSPLFTASDNGLIAIPSYEEVCIPSNEEGHSNKPAITNIVHPQMRRLNPALAGPIESKGFPVAERY

SSPMKKSIEARSITDAKDTSKPKRTGSLGLFFRKFYYLASVRLKDLCRSLHLGEDLTSKIWTCFEYSVMH

CHELIIDRHLDQLLMCAIYVICKVSRKNTKFLEIKDCYNRQPQAKKHLYRNVLVRKRKGKSDNKSVESSR

NSSPVSPPIIISGSTTIYQEQNETAMRSSSTLSQTPTPSCSDVDVENGELEDVRGDLVQFYNEVYLDAMK

EFVLRFLPENGNSQVSGRHSVFVSIFGCDSPPLSPKPKMLYSFQESPDTILKDINDAVKQSERTGDKSKA

FTARGRAKRAILQNDEPDILPKRSRQSEVLTKRLSNLTSDKEKVKRSQGE

>RB1-Ta gi|196012646|ref|XP_002116185.1| hypothetical protein TRIADDRAFT_60210 [Trichoplax adhaerens]

MASSLAVTHCANHANNDEDSSYTDQTIRDLFKDLHSNLNITDPLVLEEGWNMWKIISETFKIVKNDISQW

VCAINYIAIYTARMSSKASILKPIPKVLNHLMAARINILDFIDKLQKLGEIISVENHAAKKQIYLLSRKY

LIDAALFAKCDLLIELVFRKDIDKSIHKFKDLLDNPRSYIWLLFLVAKGTLIGTDTESVSHLIYDDKIAI

LFETVDFMKLKNIFIINIGQQVRDFHNNCWTRFQVTGSSILNPLKIDNLNREYEAAFIKNGDFDERAFLD

ESIFDSMLKPKSTGKEDRGSYRKDPPPTPVKSTMKSIANLLALLTSTEHLKMVITYRIDFVLSTTPGSLL

CDYFKKCCPNPFDSILARLERYRDVFVQKYAKQTSSAYSSISDKYFDLAKRLYFRVLENMLKGEQRRLGN

SDFSVLLNHDVFHSSLLACSLEVVLFTYNSPHTSNSNKYGYEFSFPWILDILDIKAYHFGKVIESFIRSE

LSLDNNLVQHLNFIESMILESLAWQASSPLYAALESSKIDLRYRQLVTENKTEVAGSLLETCLSPVKKSH

TSEKQSCGDETQKKLHSVDLFYRKVFQLAYLRLKNLCDALKIDEKTINRTWTCYHYALTYKTKLFMDRHI

DQVCRNVLLLSNQYGTIIAFYNQIFICEMKEFVLKLSSVNAALQLSPIPTYNSRSPRSDGVGKRIPWVSN

VYLSPLKRKDILTPRSKALYCFGDALESSRKLSEINEIVHKGSMKSSKRLKFDDTTVTADVDVTSRCAEA

RPGLSNLDQDENPTDNPTNNYKAVMLENRLADFGGSSFAVIDISRKSNTR

>Rb-Mb Mb|A9UZQ5|A9UZQ5_MONBE Predicted protein [Monosiga brevicollis ]

MEVQTEAREIVDLLVLALDPAQTWTGLESTYWSAVAMLLAARQPTASGFMLLATGGLTVSDLLQRCELNAVTFFDHCRQA

LAALRTSANVDADFSLTRLNLFEAYCNHMHRSFVVMCQLRHQLERYLYNLFSTDQHNSEIHARIKQFVWITFQAARISHI

HDTVLHTGCESLLHLLICVVHHAVVRCDAPLLRPNLAVELIKQKPSTSDPCLEQLDTICRTFKFNRDEILVIRRSFWEPF

LRDYENLLFFVDGQPQPMAMTYLSQIYEQQYYRQPQYAGLDDRLYIDQVPHLQLPPSIASRGSSTVDLLQAGTRDVLHRA

RRNLLGELRQTADPVNPEDSSAADRGHLENETGRAAVSKSDAGAAACAMLNMNCSASAPPQPQPQPQPQPQVRPPAPSSA

TTPRSRARPMPQPTPSHDALQRLRATASGTNMPSTPSSPLRQAAQQLREFMEELPQHTDGPSFELAKCFYECDQTRDTAT

TQQECKQLLSVVQDQVVRALRGHQYAYFWETHEKSLITESEKLFWWLLHDILAKSLNTSLLGRAAFLRAVMALSYEVVLQ

VHRREWFQVPVPSRLRFPWILHVAHVPPENVLKVIEPCIVHTLSAYPVITQYLADLEAGVLQQQLWAVGSSFLARMRRDG

VYLPTVHESGTIDRPGRRHPPQPHRASPARRNLSSLFADEGEPLAKRAARELAPVDNNATPSASSRSGSQASLPHTLLTQ

VAYKLYPLVIDRLSELIHRLKEVPGAVVTSITKMAWNLLLDVFHNHIQLLEGMHTDQLIICCVYASLKRRFPRQIAFKYI

REQYESMRGVNERLASTASVAWGDDLQGLQAMLQKRRIDIQDVAIYCRQRLTPMNLTVRLCVCALAGQEGALVPLPVFPE

CKRIQLTPQRHSQSPGPFGRNVFVSPLLYRSEEAAALRVTRNTPMACKTPTSRLRASDSIRTSLQDLSKAM

>Rb-Dd gi|60463973|gb|EAL62136.1| Rb-like protein [Dictyostelium discoideum AX4]

MMAHNKNDLTNINTKTTAPTTTTTEQQPEQQQQQPEQQQQEKQNNNNNNNNNNNNNNNINNNENNENNNNDIINNKINLL

DELVAAAELKGRYTQIQLEELKSKLDRTSSALNVDQDTIKLAWCLLENMQLVSVDTSEQFQKQIVACSLFICGNKKFSSL

LYNLNQPSSSSFQPDNNSKIKGRKIRKTNNSKNKNNDSNEEEEETTTDTEEEEEEDTLLNENNNSINKNSSNNNELLVSI

RSCINESGGDSQFLQNSVFLSQLLKHFEGEIKLSQFFDTLRSFISNLRLGNAFEEQGRQLEQSFNTLNNLYRKYEQIFFT

LFHPTENYILKTTQQHNYNNNSNNNNNNNNNNNNNNNNNRTNEDYLLSIGWLIFLFCKNKLFTKDSPDFVQSLHLLLCII

NFIYVNTSISELKKIPQGVQIIDTATGDILLYLAKSIPCNLDDLSNVNQNIFSTYSNLLINNSILKLFDNNNNNNNNNNN

NNNNSDGIKSIFNNSSIIKDNYNSLNKSYELYYYANGDIDERLFYSFDHDIYVLNNNNNNQNNNNNNNNHNNNYYNNNNN

NNNNNNNNNHLNYQYSSLLPPSTPTTPSRYGSSSSSGGGGGGNLRKNIYGTPSKSLSAGNLGSVIPQTPISLTLTLESWL

KVEIQNYRDPNPSLNLINILKSADQPQQTSEVDKMIERVSSLTNNTDSLFSIGGIDDHQQQQQQQQEVKQRRKNMAIQLY

YCLLEKLIKFEQNTSSNINVLLNHEDFHKSLLSNSFEIIAYVYKMEGLYFPHFINVFKLHPFSYLRLIDLVLKVDADLPK

LLAQHFSQIEEKILEKYVWSNNSTVFSTIKSHDFQNIFNSNGVGAAITTHLRPTQVFSTPTKNQNNNPFRNQQLINTNIP

NTPTKKNPFIQNFIKKVSTLIIAKCRKLIHAMGLSSDYVVQIYQVMIKILIDETLLFKNREIDVLLICSIYAICKVNSKN

ITFKAIIDKSCISAKVYKEVYIGNDENNNNNNNNNNNNNNNNNNNNNNINNNNNNNENNNNNNNPVKGDIILFYNKIFLT

KMDPYIHEVFSKYQQQQQHQYQQPLQNLPPPLLNQNSPKKWNPLFHTPTKNQNNNPYNSNNNTPNKFSSSIPLSPQKGVN

YNSSFSVNSMVTISPMKQHTPISSSYTYIVAKSPSKELFQINQCLNKKKNDEMVPPPPTTPSSINNNNNNNNNNNNNNNN

NNNNNSNDNNNNNNNNNNNNNNNNSNNNSNNNNNTEKKMVGKRLLFDYEESPSTPSSSSSPTILNNNKKNNNNNKSENVD

DPGNNSPSSSPLSSSSSSSSSSSSGGRKRLKS

>Os08g0538700-Os gi|115477553|ref|NP_001062372.1| Os08g0538700 [Oryza sativa (japonica cultivar-group)]

MEGAAPPASSGSEVTGAGSGKVDAGGGAAMEERFADLCKSKLGLDESITRQAMQLFKESKSILLSSMSSLGSGSPEEIER

FWSAFVLYCVSRLGKAGKGKEDGGISLCQILRAFSLNIVDFFKEMPQFCIKVGSVLAGLYGSDWEKRLELKELQANVVHL

SLLSRYYKRAYQELFLLNDAKPPENSAEPNAQASDYYRFGWLLFLVLRIQTFSRFKDLVTSTNGLVSVLAVLIVHIPVRL

RNFNIKESSSFAKKSDKGVNLIASLCEKYHTSEDELSKAIEKTNTLIVDILKKKPCPAASECQQDRLSFIDPEGLTYFKN

LLEEDSLKLSLLMLEKEYENAINTKGELDERMFANDEDSLLGSGSLSGGAINLPGTKRKYDVMASPAKSITSPSPMSPPR

FCASPTGNGYCSSKMAPITPVSTAMTTAKWLRSTISPLPSKPSGELLRFFSACDKDVTDDITRRAGIILGAIFTSSSFGE

RICTSVRSTNRIDAIWTEQRKMEALKLYYRVLESMCRAETQILSGNNLTSLLSNERFHRCMIACSAELVLATHKTVTMMF

PAVLEKTGITAFDLSKVIESFVRHEDTLPRELKRHLNSLEERLLESMAWEKGSSMYNSLIVARPTLSAEINRLGLLAEPM

PSLDAIAAHHNISLEGLPPLPFQKQEHSPDKDEVRSPKRACTERRNVLVDNNSFRSPVKDTLKSKLPPLQSAFLSPTRPN

PAAGGELCAETGIGVFLSKIAKLAAIRIRGLCERLQLSQQVLERVYSLVQQIIIQQTALFFNRHIDQIILCSIYGVAKIS

QLALTFKEIIFGYRKQSQCKPQVFRSVYVHWASRSRNGKTGEDHVDIITFYNEVFIPTVKPLLVELGSGTSPNKKNEEKC

AADGPYPESPRLSRFPNLPDMSPKKVSAAHNVYVSPLRTSKMDTLLSPSSKSYYACVGESTHAFQSPSKDLKVINNRLNS

GKKVSGRLNFDVVSDLVVARSLSDQNSASAAATTADIATKTPVKLEQPDC

>Os11g0533500-Os gi|115485753|ref|NP_001068020.1| Os11g0533500 [Oryza sativa (japonica cultivar-group)]

MASQPPAAVEARLADLCKELGVDEGVAGEAAAVLEEGKGALLASPSFGSKSPEDAEKLCFAFVLYCVSKLKETKAGSSGV

RLWEILKGCKLKYDDFFKESQRLASRIDQVLGSRYGSDWEARLELKQLENLVNLLADASRFYCKAFNELFLSPSTDQEPG

STTNIPDYIRFGWLLFLILRSKSPELFKDLVSCIHGLVAILAILLIHVPAKFRSFTIEGSSHLIKQTEKGVDLLPSLCHN

YHTSEDRLKEMMGKSYKVIEVFFSRKAINASEFKTVNLDKIDTDGLMYFKDLVDDEIFQSNLEKLEKLSSTTGCQGELDL

EMFLTSNDYVLNAENSSGSSANFGCSKRVFETLASPTKTIKNMLAAPSSPSSPANGGSIKIVQMTPVTSAMTTAKWLRDV

ISSLPDKPSSKLEEFLSSCDTDLTSDVVKRVSIILEAIFPTKSIDRGTSIGLNCANAFDIPWAEARKMEASKLYYRVLEA

ICRAESQNNNVNNLTPLLSNERFHRCLIACSAELVLATHKTVIMMFPAVLESTGLTAFDLSKIIENFVRHEETLPRELKR

HLNSLEEQLLESMSWEKGSSLYNSLVVARPSLSTEINSLGLLAEPMPSLDGIVARQSIHPDGLPPTPSKRWPSAGPDGNC

YPQSPKRLCTESRNSLVERNSQTPPPKQSQTGLSILKAKYHPLQATFASPTVSNPVSGNEKCAVVGVQIFFSKILKLAAI

RIRNLCERLRHEELTVSVYNIFKQILDQQTALFFNRHVDQIILCCLYGVAKVSQLSLTFKEIVNNYKREPQCKPEVFRSI

FVGSTNRNGGFGSRHVDIIVFYNQVFVPTVKPLLVALMPSSTRPEDKRNTNSQIPGSPKSSPFSNLPDMSPKKVSSSHNV

YVSPLRQTKMDALLSPSSRSFYACIGESTQAFQSPSKDLAAINSRLNYPTRRINTRINFDMVSDSVVAGSLGQPNGGSAS

SDPAAAFSPLSKKSKTDS

>RBR1-At gi|30682129|ref|NP_566417.3| RBR1 (RETINOBLASTOMA-RELATED 1) [Arabidopsis thaliana]

MEEVQPPVTPPIEPNGKRSEASLLDICEKVLSLDGSTCDEALKLFTETKRILSASMSNIGSGTREEVERFWFAFILYSVK

RLSVRKEADGLSVSGDNEFNLCQILRALKLNIVDFFKELPQFVVKAGSVLGELYGADWENRLQAKEVQANFVHLSLLSKY

YKRGFREFFLTYDANAEKNSANSSTYLLDSYRFGWLLFLALRNHAFSRFKDLVTCSNGVVSILAILIIHVPCRFRNFSIQ

DSSRFVKKGDKGVDLVASLCKIYDASEDELRIVIDKANNLVETILKKKPSPASECQTDKLDNIDPDGLTYFEDLLEETSI

STSLITLEKDYYDGKGELDERVFINEEDSLLGSGSLSAGAVNITGVKRKIDALSSPARTFISPLSPHKSPAAKTNGISGA

TKLAATPVSTAMTTAKWLRTVISPLLPKPSPGLEHFLKSCDRDITNDVTRRAHIILEAIFPNSSLGAQCGGGSLQAVDLM

DDIWAEQRRLEACKLYYRVLEAMCKAEAQILHANNLNSLLTNERFHRCMLACSAELVLATHKTITMLFPAVLERTGITAF

DLSKVIESFIRHEDSLPRELRRHLNSLEERLLESMVWEKGSSMYNSLIVARPSLALEINQLGLLAEPMPSLDAIAALINF

SDGANHASSVQKHETCPGQNGGIRSPKRLCTDYRSILVERNSFTSPVKDRLLALGNVKSKMLPPPLQSAFASPTRPNPGG

GGETCAETGINIFFTKINKLAAVRINGMVERLQLSQQIRESVYCFFQHVLAQRTSLLFSRHIDQIILCCFYGVAKISQMS

LTFREIIYNYRKQPQCKPLVFRSVYVDALQCRRQGRIGPDHVDIITFYNEIFIPAVKPLLVELGPVRNDRAVEANNKPEG

QCPGSPKVSVFPSVPDMSPKKVSAVHNVYVSPLRGSKMDALISHSTKSYYACVGESTHAYQSPSKDLSAINNRLNNSSSN

RKRTLNFDAEAGMVSDSMVANSLNLQNQNQNQNGSDASSSGGAAPLKTEPTDS

>Rb-Ot gi|116055560|emb|CAL58228.1| retinoblastoma protein (IC) [Ostreococcus tauri]

MRDVLDAARGTLSARGNESDGAGEGTRAACGALAALGLLDAEDAVRDGGATGAAGEGGDVTGVLEDCETTLVEVLDAMPE

FLSAGARALATRLKSPSLGSSAVAEAERAMRLREMRSAFAFNKVIAKKFHEFIRVHFDADSRGGRTVARLGWAVYQCAKC

EALPKFPDLYSCYHLLVAVEAFLLVNAPRELLRTSLKNMVSMTAKDAVTGLPDPLASLSSSSKTKVETVRAMSVAVINVL

KATFPSAVFDVTSHFEDVAPSDACVRGVFDGDADVASPVMERYRRVAAETFGRLAVDETLYLYTEMEGEDSRGRKIIGDL

ASCATETTHGSMATPTRRKRAAAPFSPYRPKKISTIQEDGGMSMSPMRAIRGPLPAGVVPPTPISQAMASASWLQDIVCA

SSDLETKLAELRRFVPGEETVIDKLHKKVDVLGQRLGQAIREDALVTTMRTDISPHSMVMDELVRQRTTELVHIFFFFLN

RILRAESQVKKDANIVALLQSSRFTKSLLACCMEVIVATYKTSTLTFPATTHLLGIHPFDLTTIIEPFVRADMDLPREIV

RHFNSLEEKTLERLAWCKGSALFDFLQSFQESVNRGGTTSTSTSNPRATSIPKRPVSPMFNVAAIAEVAAMGEPARDAAA

EKDLVMPQCHSPVRRPPTSAFTVFSSPLRGATTPRRKLPGGQPLPERFAAVRNHDVEQPIGCDVCAFKALQIFFAKVMQL

AARRLGDLASRLKLSPEVTRDVYALIEHVIYEQTNLVYNRHVDQIILASVYGVCKVNGGCGGAVQFKDIIYQYSKQPQCT

EEIFWTVVIEQTDPELEVSTRGDIISFYNKVFVSRVRTFLLALRERAEALAAQKTTDGEKVSVDEPFPFGISSPRRRLPV

ENQNIYVSPMRPEREAAMLQDAAVQGEPSTPRTRSLFATIGESIHGDPSSANDFEAINKHLAMKATSTPSRLGARVPEFS

KR

**Sequences for DP family:**

>DP1-Hs gi|6005900|ref|NP_009042.1| transcription factor Dp-1 [Homo sapiens]

MAKDAGLIEANGELKVFIDQNLSPGKGVVSLVAVHPSTVNPLGKQLLPKTFGQSNVNIAQQVVIGTPQRPAASNTLVVGS

PHTPSTHFASQNQPSDSSPWSAGKRNRKGEKNGKGLRHFSMKVCEKVQRKGTTSYNEVADELVAEFSAADNHILPNESAY

DQKNIRRRVYDALNVLMAMNIISKEKKEIKWIGLPTNSAQECQNLEVERQRRLERIKQKQSQLQELILQQIAFKNLVQRN

RHAEQQASRPPPPNSVIHLPFIIVNTSKKTVIDCSISNDKFEYLFNFDNTFEIHDDIEVLKRMGMACGLESGSCSAEDLK

MARSLVPKALEPYVTEMAQGTVGGVFITTAGSTSNGTRFSASDLTNGADGMLATSSNGSQYSGSRVETPVSYVGEDDEED

DDFNENDEDD

>DP2-Hs gi|5454112|ref|NP_006277.1| transcription factor Dp-2 (E2F dimerization partner 2) [Homo sapiens]

MIISTPQRLTSSGSVLIGSPYTPAPAMVTQTHIAEATGWVPGDRKRARKFIDSDFSESKRSKKGDKNGKGLRHFSMKVCE

KVQRKGTTSYNEVADELVSEFTNSNNHLAADSQAYDQKNIRRRVYDALNVLMAMNIISKEKKEIKWIGLPTNSAQECQNL

EIEKQRRIERIKQKRAQLQELLLQQIAFKNLVQRNRQNEQQNQGPPALNSTIQLPFIIINTSRKTVIDCSISSDKFEYLF

NFDNTFEIHDDIEVLKRMGMSFGLESGKCSLEDLKLAKSLVPKALEGYITDISTGPSWLNQGLLLNSTQSVSNLDLTTGA

TLPQSSVNQGLCLDAEVALATGQFLAPNSHQSSSAASHCSESRGETPCSFNDEDEEDDEEDSSSPE

>DP3-Hs gi|189409125|ref|NP_057605.3| transcription factor Dp family, member 3 [Homo sapiens]

MAKYVSLTEANEELKVLMDENQTSRPVAVHTSTVNPLGKQLLPKTFGQSSVNIDQQVVIGMPQRPAASNIPVVGSPNPPS

THFASQNQHSYSSPPWAGQHNRKGEKNGMGLCRLSMKVWETVQRKGTTSCQEVVGELVAKFRAASNHASPNESAYDVKNI

KRRTYDALNVLMAMNIISREKKKIKWIGLTTNSAQNCQNLRVERQKRLERIKQKQSELQQLILQQIAFKNLVLRNQYVEE

QVSQRPLPNSVIHVPFIIISSSKKTVINCSISDDKSEYLFKFNSSFEIHDDTEVLMWMGMTFGLESGSCSAEDLKMARNL

VPKALEPYVTEMAQGTFGGVFTTAGSRSNGTWLSASDLTNIAIGMLATSSGGSQYSGSRVETPAVEEEEEEDNNDDDLSE

NDEDD

>DP1-Cf gi|73989562|ref|XP_849597.1| PREDICTED: similar to Transcription factor Dp-1 (E2F dimerization partner 1) (DRTF1-polypeptide-1) (DRTF1) [Canis familiaris]

MSAISMGQGWQHSWHSSERPAGLIEANGELKVFIDQNLSPGKGVVSLVAVHPSAVNTLGKQLLPKTFGQSNVNIAQQVVI

GTPQRPAAPNTIVVGSPHTPNTHFVSQNQPSDPSPWSAGKRHRKGEKNGKGLRHFSMKVCEKVQRKGTTSYNEVADELVA

EFSAADNHILPNESAYDQKNIRRRVYDALNVLMAMNIISKEKKEIKWIGLPTNSAQECQNLEVERQRRLERIKQKQSQLQ

ELILQQIAFKNLVQRNRQAEQQASRPPPPNSVIHLPFIIVNTSKKTVIDCSISNDKFEYLFNFDNTFEIHDDIEVLKRMG

MACGLESGSCSAEDLKMARSLVPKALEPYVTEMAQGSIGGVFVTSSVSTSNGTRLSASDLTNGADGMLATSSSGSQYSGS

RVETPVSYVGEDEEEDDDFNENEEED

>DP2-Cf gi|73990311|ref|XP_852372.1| PREDICTED: similar to Transcription factor Dp-2 (E2F dimerization partner 2) isoform 2 [Canis familiaris]

MTAKNVGLTSTNAELRGFIDQNLSPTKGNISFVAFPVSSTNSPTKILPKTLGPINVNVGPQMIISTPQRLTSSASVLIGS

PYTPAPAMVTQTHIAEATGWVPGDRKRAREFIDSDFSESKRSKKGDKNGKGLRHFSMKVCEKVQRKGTTSYNEVADELVS

EFTNSNNHLAADSAYDQKNIRRRVYDALNVLMAMNIISKEKKEIKWIGLPTNSAQECQNLEIEKQRRIERIKQKRAQLQE

LLLQQIAFKNLVQRNRQNEQQNQGPPALNSTIQLPFIIINTSRKTVIDCSISSDKFEYLFNFDNTFEIHDDIEVLKRMGM

SFGLESGKCSLEDLKLAKSLVPKALEGYITDISTGPSWLNQGLLLNSTQSVSNLDLTTGATLSQSSVNQGLCLDAEVALA

TGQFLAPNSHQSSSAASHCSESRGETPCSFNDEDEEDDEEDSSSPE

>DP1-Bt gi|115496726|ref|NP_001069497.1| transcription factor Dp-1 [Bos taurus]

MAKDAGLIEANGELKVFIDQNLSPGKGVVSLVAVHPSTVNSLGKQLLPKTFGQSNVNITQQVVIGTPQRPAAPNTIVVGS

PHTPNTHFVSQNQPSDPSPWSAGKRNRKGEKNGKGLRHFSMKVCEKVQRKGTTSYNEVADELVAEFSAADSHILPSESAY

DQKNIRRRVYDALNVLMAMNIISKEKKEIKWIGLPTNSAQECQSLEVERQRRLERIKQKQSQLQELILQQIAFKNLVQRN

RQVEQQASRPPPPNSVIHLPFIIVNTSKKTVIDCSISNDKFEYLFNFDNTFEIHDDIEVLKRMGMACGLESGSCSPEDLR

VARSLVPKALEPYVTEMAQGSLGGVFVASAVSTSNGTRLSASDLANGADGALATSSSGSQYSGSRVETPVSCVGEDDEDD

EDFNENEEED

>DP2-Bt ENSBTAP00000008169 [Bos taurus]

MIISTPQRLTSSGSVLIGSPYTPAPAMVTQTHIAEAAGWVPGDRKRAREFIDSDFSESKRSKKGDKNGKGLRHFSMKVCE

KVQRKGTTSYNEVADELVSEFTNSNNHLAADSAYDQKNIRRRVYDALNVLMAMNIISKEKKEIKWIGLPTNSAQECQNLE

IEKQRRIERIKQKRAQLQELLLQQIAFKNLVQRNRQNEQQNQGPPALNSTIQLPFIIINTSRKTVIDCSISSDKFEYLFN

FDNTFEIHDDIEVLKRMGMSFGLESGKCSLEDLKLAKSLVPKALEGYITDISTGPSWLNQGLLLNSTQSVSNLDLTTGAT

LSQSSVNQGLCLDAEVALATGQLLAPNSHQSSSAASHCSESRGETPCSFNDEDEEEDEEDSSSPE

>DP1-Mm gi|6678305|ref|NP_033387.1| transcription factor Dp 1 [Mus musculus]

MAKDASLIEANGELKVFIDQNLSPGKGVVSLVAVHPSTVNTLGKQLLPKTFGQSNVNITQQVVIGTPQRPAASNTIVVGS

PHTPNTHFVSQNQTSDSSPWSAGKRNRKGEKNGKGLRHFSMKVCEKVQRKGTTSYNEVADELVAEFSAADNHILPNESAY

DQKNIRRRVYDALNVLMAMNIISKEKKEIKWIGLPTNSAQECQNLEVERQRRLERIKQKQSQLQELILQQIAFKNLVQRN

RQAEQQARRPPPPNSVIHLPFIIVNTSRKTVIDCSISNDKFEYLFNFDNTFEIHDDIEVLKRMGMACGLESGNCSAEDLK

VARSLVPKALEPYVTEMAQGSIGGVFVTTTGSTSNGTRLSASDLSNGADGMLATSSNGSQYSGSRVETPVSYVGEDDDDD

DDFNENDEED

>DP2-Mm gi|182765448|ref|NP_848782.2| transcription factor Dp-2 [Mus musculus]

MIISTPQRIANSGSVLIGNPYTPAPAMVTQTHIAEAAGWVPSDRKRAREFIDSDFSESKRSKKGDKNGKGLRHFSMKVCE

KVQRKGTTSYNEVADELVSEFTNSNNHLAADSAYDQKNIRRRVYDALNVLMAMNIISKEKKEIKWIGLPTNSAQECQNLE

IEKQRRIERIKQKRAQLQELLLQQIAFKNLVQRNRQNEQQNQGPPAVNSTIQLPFIIINTSRKTVIDCSISSDKFEYLFN

FDNTFEIHDDIEVLKRMGMSFGLESGKCSLEDLKIAKSLVPKALEGYITDISTGPSWLNQGLLLNSTQSVSNLDPTTGAT

VPQSSVNQGLCLDAEVALATGQLPASNSHQSSSAASHFSESRGETPCSFNDEDEEDEEEDPSSPE

>DP2L-Mm gi|149260219|ref|XP_001481321.1| PREDICTED: similar to Transcription factor Dp-2 (E2F dimerization partner 2) (Dp-3) [Mus musculus]

MTAKNVGLPSTNAELRGFIDQNFSPTKGNISLVAFPVSSTNSPTKILPKTLGPINVNVGPQMIISTPQRIANSGSVLIGN

PYTPAPAMVTQTHIAEAAGWVPSDRKRAREFIDSDFSESISQXKKNKNNGKSQEAHSNSVCESLQSGAGECFSHRTEELA

SEIKDKQAKGRISQAYDQKNIRRRVYDALNVLMAMNIISKEKKEIKWIGLPTNSAQECQNLEIEKQRRIERIKQKRAQLQ

ELLLQQIAFKNLVQRNRQNEQQNQGPPAVNSTIQLPFIIINTSRKTVIDCSISSDKFEYLFNFDNTFEIHDDIEVLKRMG

MSFGLESGKCSLEDLKIAKSLVPKALEGYITDISTGPSWLNQGLLLNSTQSVSNLDPTTGATVPQSSVNQGLCLDAEVAL

ATGQLPASNSHQSSSAASHFSESRGETPCSFNDEDEEDEEEDPSSPE

>DP1-Rn gi|71043770|ref|NP_001020889.1| hypothetical protein LOC361178 [Rattus norvegicus]

MAKDASLIEANGELKVFIDQNLSPGKGVVSLVAVHPSTVNTLGKQLLPKTFGQSNVNITQQVVIGTPQRPAAANTIVVGS

PHTPNTHFVSQNQTSDSSPWSAGKRNRKGEKNGKGLRHFSMKVCEKVQRKGTTSYNEVADELVAEFSAADNHILPNESAY

DQKNIRRRVYDALNVLMAMNIISKEKKEIKWIGLPTNSAQECQNLEVERQRRLERIKQKQSQLQELILQQIAFKNLVQRN

RQAEQQARRPPPPNSVIHLPFIIVNTSRKTVIDCSISNDKFEYLFNFDNTFEIHDDIEVLKRMGMACGLESGSCSAEDLK

VARSLVPKALEPYVTEMAQGSIGGVFVTTTGSTSNGTRLSASDLSNGADGMGHELQWVSVQRLQGGDPCVLRWGG

>DP2-Rn gi|157818883|ref|NP_001100317.1| transcription factor Dp-2 (E2F dimerization partner 2) [Rattus norvegicus]

MIISTPQRITNSGSVLIGSPYTPAPAMVTQTHIAEAAGWVPSDRKRAREFIDSDFSESKRSKKGDKNGKGLRHFSMKVCE

KVQRKGTTSYNEVADELVSEFTNASNHLAADSAYDQKNIRRRVYDALNVLMAMNIISKEKKEIKWIGLPTNSAQECQNLE

IEKQRRIERIKQKRAQLQELLLQQIAFKNLVQRNRQNEQQNQGPPAVNSTIQLPFIVINTSRKTVIDCSISSDKFEYLFN

FDNTFEIHDDIEVLKRMGMSFGLESGKCSLEDLKIAKSLVPKALEGYITDVSTGPSWLNQGLLLNSTQSVSNLDPTTGAT

VPQSSVNQGLCLDAEVALATGQFLAPNSHQSSTAASQFSESRGETPCSFNDEEEEDEEEDPSSPE

>DP1-Gg gi|50730508|ref|XP_416938.1| PREDICTED: similar to transcription factor [Gallus gallus]

MAKDAGLIEANGELKVFIDQNLSPGKGVVSLLAVHPSTVNTLGKQLLPKTFGRSNVNIAQQVVIGTPQRPSVPNTILVGS

PHTPNTHFVSQNQTADSSPWSAGKRNRKGEKSGTGLRHFSMKVCEKVQRKGTTSYNEVADELVAEFTTPDDHISPNESAY

DQKNIRRRVYDALNVLMAMNIISKEKKEIKWIGLPTNSAQECQNLEVEKQRRLERIKQKQSQLQELILQQIAFKNLVQRN

RQAEQQANRPPPSNSVIHLPFIIVNTSKKTVIDCSISNDKFEYLFNFDNTFEIHDDIEVLKRMGMACGLESGSCSAEDLK

IARSLVPKALEPYVTEMAQGSISSVYVTSSSGSTSNGTRFSASDFSNGGDGMLATSSNGSQYSGSRVETPVSYVGDDDDD

DDDFNENDDDD

>DP2-Gg gi|118095071|ref|XP_422598.2| PREDICTED: similar to Transcription factor Dp-2 (E2F dimerization partner 2) [Gallus gallus]

MTAKNVGVTSTNGDLKGFIDQNQSPTKGNISVITLPVSSTNSPTKILPKTLGPINVNVGPQMIISTSQRLTNSGGVLIGS

PYNPAPTMVTQTHITEATGWIPGERKRTREFIESDFSESKRSKKGDKNGKGLRHFSMKVCEKVQRKGTTSYNEVADELVS

EFTNSNSHLAADSAYDQKNIRRRVYDALNVLMAMNIISKEKKEIRWIGLPTNSAQECQNLEIEKQKRIERIKEKRAQLQE

LLLQQIAFKNLVQRNQQNEQQNRGPPASNSTIQLPFLIVNTSKRTVIDCSISSDKFEYLFNFDNAFEIHDDSEVLKRMGM

SFGLEEGKCSAEDLRTAKSLVPKALEGYITDMSTGLSWMNQGLLSAQAVSHSEIAGGTSDSKSSENPGLCLDTEVALATG

QFLAPSSQQSSSATSRYSESRGETPCSFNEEEEEDEDEDDPSSPE

>DP1-Xt gi|58332126|ref|NP_001011211.1| hypothetical protein LOC496643 [Xenopus tropicalis]

MAKDVGLIEANGELKVFVDQNLSPEKGVVSLLTVHPSSVSSIGKQLLPKTFGQSNVNITQQVVLGTPQRQSAPNTILIGS

PHTPNTHFVSQNQVTDSSPWSAGKRNKKGEKNGKGLRHFSMKVCEKVQKKGTTSYNEVADELVAEFSSADNHISPNESQA

YDQKNIRRRVYDALNVLMAMNIISKEKKEIKWIGLPTNSAQECQNLEVERQRRLERIKQKQSQLQELILQQIAFKNLVQR

NRLTEQKANRPPPPNSVIHLPFIIVNTSKKTVIDCSISNDKFEYLFNFDNTFEIHDDIEVLKRMGMACGLESGSCSAEDL

KTARSLVPKALEPYVTEMAQGSISSVYISSSSGSVSNGRRFSSSDLTGCTDSMLATSSNGSQYSSSRVETPVSYVGEEDD

DDEDDFNDDDD

>DP2-Xt gi|166158068|ref|NP_001107442.1| hypothetical protein LOC100135290 [Xenopus tropicalis]

MMANTIGAAALCGDLKAFLNQNLSKGNISLVTPSSYSPANKITLGTVNSNVGAQMILSTPQRVSHSGNILIGSPFTPHTT

PVAMVTQAHPPEGNEWTPGNKKRTHDYIDSYYSDRDGLCSSDSSSSKRLKRADKNGKGLRHFSMKVCEKVQKKGTTSYNE

VADELVAEFTHASSLMPADSQVCDQKNIRRRVYDALNVLMAMNIISKEKKEIRWIGLPTNSAQECRNLELEKQKRLERIR

QKRAQLEELILQQVAFKNLVQRNQASEASSRSSPPAGSVIQLPFIILNTDVRTVIDCSISSDKCEYLFNFDNTFEIHDDV

EILKRMGMSLGLENGTCSPQNLTLAKSLVPKSLEAYITSTCKTFWVPHNRFLLG

>DP1-Dr gi|41152118|ref|NP_957070.1| transcription factor Dp-1 [Danio rerio]

MAKDAGLKETNGEIKLFVNQSPGKAAGVLQLLTVHPASITTVKQILPKTLTVAAGAHVLPHMVVSTPQRPTIPVLLTSPH

TPTAQTQQESSPWSSGHCRRADKSGKGLRHFSMKVCEKVQKKVVTSYNEVADELVQEFSSADHSSISPNDAVTSCHVYDQ

KNIRRRVYDALNVLMAMNIISKDKKEIKWIGFPTNSAQECEDLKAERQKRQERIKQKQSQLQELIVQQIAFKNLVQRNRE

VEQQSKRSPSANTIIQLPFIIINTSKKTIIDCSISNDKFEYLFNFDSMFEIHDDVEVLKRLGLALGLESGRCSAEQMKIA

TSLVSKALQPYVTEMAQGSVNQLMDFSHVAAERRASSSTSSRVETPTSLMEEDEEDEEEDYEEEDD

>DP2-Dr gi|38016161|ref|NP_937851.1| transcription factor Dp-2 [Danio rerio]

MMANTIGAAALCGDLKAFLNQNLSKGNISLLTPSSYSPANKITLGTVNSNVGAQMILSTPQRVSHSGNILIGSPFTPHTT

PVAMVTQAHPPEGNEWTPGNKKRTHDYIDSYYSDRDGLCSSDSSSSKRLKRADKNGKGLRHFSMKVCEKVQKKGTTSYNE

VADELVAEFTHASSLMPADSQVYDQKNIRRRVYDALNVLMAMNIISKEKKEIRWIGLPTNSAQECRNLELEKQKRLERIR

QKRAQLEELILQQVAFKNLVQRNQASEASSRSSPPAGSVIQLPFIILNTDVRTVIDCSISSDKCEYLFNFDNTFEIHDDV

EILKRMGMSLGLENGTCSPQNLTLAKSLVPKSLEAYITNMARNSTRTPLSLTSSTALSHTSESRVLTPSSFNEADDEDDD

DDDSPSSPE

>DP3-Dr gi|189523624|ref|XP_001918928.1| PREDICTED: hypothetical protein [Danio rerio]

MAKDAGLIEANGELKVFIDQNLSPGKGVLSLVTVHPQALTVGKQLLPKTLGPSNVNIAPHMVISTPQRPSGSNVILMNSP

HTPSSQFITQSQPSEASPWSSGKRGKKGEKNGKGLRHFSMKVCEKVQRKGVTTYNEVADELVAEFSSGDNHISPNDAHVY

DQKNIRRRVYDALNVLMAMNIISKEKKEIKWIGLPTNSAQECQNLEVERQRRLERIKQKQSQLQELILQQIAFKNLVQRN

RQREQQTKRPPPANSVIHLPFIIVNTSKKTVIDCSISNDKFEYLFNFDSMFEIHDDIEVLKRMGMACGLEVGKCSAEDLK

TARSLVPKALEPYVTEMAQGPISNVYMTGASSANGGGRYHVGGDSGADATMASSSNDSHYSGSRVETPVSYMGEDDDDDD

EFDENDDED

>DP-Tn ENSTNIT00000017108 [Tetraodon nigroviridis]

MAKDAGLIETNGELKIFVDQNMSPSKEVYSLVTVQPAAVAKQLLPKTLGPSNVNVAAHVHLQVIGTPQRPTVSNAILVNS

PHTPSSQFLTQTQQSDASPWSSGKRGKKGEKNGKGLRHFSMKVCEKVQKKGVTTYNEVADELVAEFSSSDNHMSPNDAHV

YDQKNIRRRVYDALNVLMAMNIISKEKKEIKWIGLPTNSAQECQNLEVERQRRLERIKQKQSQLQELILQQIAFKNLVQR

NKQTELQANRPPPPNSIIHLPFIIVNTSKKTVIDCSISSDKFEYLFNFDSMFEIHDDIEVLKRMGMACGLEVGKCSAEDL

KVARSLVPKALEPYVIEMAKGPISNVYVTGGSSANGARYPGSDGCTDGTMASSSNDSHYSGSRVETPVSYMGVDDDDRKK

KRQQKKAR

>DP-Sp gi|72048148|ref|XP_798717.1| PREDICTED: similar to MGC81762 protein [Strongylocentrotus purpuratus]

MESSASLTSLWGPEGQGSPLKTQPSTPSKSSGKDIVGDEVISFYDDAGLGGIAREAKLIDPSGILRRDTNLQQVTRVIQL

PQFISPKPSPMKTTTQILPKVATIVTTTNVSGLPMILTPQRPCTTVLAASPSQSQFTPVSLQSPSWSGSSQKRKADFSDE

FDNSNKRKKREKESKGLRHFSMKVCEKVQQKGITSYNEVAEELVREFSQPAHQFLPSESHQYDQKNIRRRVYDALNVLMA

MNIISKEKKEIKWIGLPTNSRQECDKLETEKRKRLDSIRQKKSQLQELLLQQIAFKNLVTRNKRLEDTGASPPTSNSAIH

LPYIIVNTSKKTVIDCSISNDKYEYLFNFNDTFQIHDDIEVLKRMGMAYGLEKGQCSHSDLERAKTMIPKTLEPYLAEMA

QQSQSSSSTTIIAGPSGIQTQGASKTHLPGDPQFSPLAVPLALAHSVDAEVALATADLAISQSSSLSSYSDAGPSRGATD

TPSSLLDSESDESSRESFQV

>DP-Ci gi|118343721|ref|NP_001071681.1| transcription factor protein [Ciona intestinalis]

MEVEQNAKSTCATIRPHAPTISVVHTTPVKGQSVFLTTPSSGSGIVRTAGSQVRVVQTTPQQTPQVVRIIKAGEAVTGSP

NVINIRAPLASPVKGGAIINIPTGSKLLTHPGPKLKVGDTITIQKSNIINENQSSSNDPNDVIQPSGTKLVKVVTSNQTP

VFIAPNTNNMKQMTPSTPQNKSFFSQQMASPVRINNMSPTWNASSPSYKRPSMFNDPAQMVESKRRRTPTTDKGSKGLRH

FAMLVCEKVKQKVTTTYNEVADELVSEFADHQRQVTDQYDQKNIRRRVYDALNVLMAMDIIYKDKKDIHWVGLPTNSAQE

VQTLQTEKKNRQQRIQQKTLQLHELILQQIAFKNLVQRNKRIEQTQGFPADNSSIQLPFIIVNTSKKTVIDCSISNDKYE

YMFNFDNTFEIHDDIEVLKRMGMAYGLEAGVCSNDNLRTAMTLVPPVLKPYLEQMAGKTSGEVSEASVGTPEVKIEPQTI

DVSSFTPDDEVAMVIDGGVGCAGSSRSSSAVYSGTPLSGDIESGSESRGSTPLF

>DP-BF gi|260816838|ref|XP_002603294.1| hypothetical protein BRAFLDRAFT_207856 [Branchiostoma floridae]

VGLIDTNGELAGFVRSHQDQNTSPTKGTVQLLCFPSRIHTIGETIILPKTLGPANTPASPMILQQSPARPTPVAQLVQVQ

QSPARAQQTTYTTVVTDPNLIQVGGQVRKRTHDFIEADFSESKRKKGEKGGKGLRHFSMKVCEKVQRKGTTSYNEVADEL

VAEFSDPQRHLSPSDQIGNAYDQKNIRRRVYDALNVLMAMNIISKEKKEIKWIGLPTNSAQECQNLELEKQRRIDRIKQK

QQQLQELILQQIAFKNLVQRNKRMEQAGTGPPNQNSAIQLPFIIVNTSKKTVIDCSISNDKYEYLFNFDNTFEIHDDIEV

LKRMGMAYGLESGKCAPENLKIAKTLVPRALEPYVEGKS

>DP-Nv gi|156375187|ref|XP_001629963.1| predicted protein [Nematostella vectensis]

QVVGTPPSQSLIAVSNSPRTPAMIAAPIIPSPSNTWPSASRSAVGDKSGKGLRHFSMKVCEKVQQKGTTSYNEVADELVR

EFSDPEKHMSPTDQAYDQKNIRRRVYDALNVLMAMNIISKEKKEIKWIGLPTNSAQECQSLEVEEKKDKRDRIKQKTAQL

QELILQQIAFKNLVQRNRSSEKQQGLPAANTTIHLPFIIVNTSKKTIIDCSISNDKFEYLFNFDNTFEIHDDIEVLKCMG

MAFGLEKKSCSVENLKTSRTMVPKALEPYVVGKESITEYV

>DP-Ta gi|196015549|ref|XP_002117631.1| hypothetical protein TRIADDRAFT_32839 [Trichoplax adhaerens]

MKVCEKVQQKGTTTYNEVVADELVREFSGDPDRGMPSNDQTYDQKNIRRRVYDALNVLMAMNIISKEKKE

IKWIGLPTNSAQECDTLEKELGTLRDRVQEKKERLRELIIQQYAYKNLVKRNRERENENALKENSCIQLP

FVVINTSKQTVIDCSISNDKFEYLFNFDNTFEIHNDIEILKRMGIVSSDVLDTTGNLNVKELEEIKTIVP

KSVGDNVPGTFNNFTSVDNISL

>DP-Mb gi|167516980|ref|XP_001742831.1| hypothetical protein [Monosiga brevicollis MX1]

MATSMSNGSPNTKRKKVDRSSRGLKHFATMVCAKVQEKNVTTYSEVADELVVQHQRELQSNPDPNDDGEPKNIRRRVYDA

LNVLMALNIISKDKKCIRWEGLPTNVTQEARTYAATKRKKEEQVQKLKKQLQALVLQHIAFQNLIKRNQARMQENPSTEE

HDSIQLPFVIVSTRQSAVIDCQMAADQSEYFFDFNEPFVIHDDLQVLSQLGLTFGLETGAISDEQVALARDLLPAEMGHI

LDEMVQEGRARAPQAPATAAAPAPAATASTAATAATASATASIGTVSQSATAAASNKTAAAARSAPTSSS

>DP-Dm gi|17136994|ref|NP_477039.1| DP transcription factor CG4654-PA, isoform A [Drosophila melanogaster]

MAHSTGGTVKTDEVNFFFRNEHGQISKMLKPAQNKSEMEGGKPVAVVYATGSSARNSGSASGIGNVGRMGAFSQMGSQGQ

FIRLQDNGLSIPKTEAGTTYTTVSAQKTSGAGSGHYDLPLKGDRYVKFTPNPIKMKSKLHAIQSNSLHSMSASSSSVQRK

RKPDKAGKGLRHFSMKVCEKVEEKGKTTYNEVADDLVSEEMKNNAYDNNCDQKNIRRRVYDALNVLMAINVISKDKKEIR

WIGLPANSTETFLALEEENCQRRERIKQKNEMLREMIMQHVAFKGLVERNKRNESQGVVPSPNASIQLPFIIVNTHKSTK

INCSVTNDKSEYIFKFDKTFEMHDDIEVLKRMGFLLGLDKGECTPENIERVKSWVPPNLAKYVEAYGTGKTGENMYESDD

EDNEFNGYLESANESQGFAQHSAQHTTDGEFKLEMDDDELDDDID

>dpl-1-Ce gi|17532739|ref|NP_495957.1| vertebrate transcription factor DP-Like family member (dpl-1) [Caenorhabditis elegans]

MNPTNYDPRIGQPAQSRPQVSLSMGRRIVQMPTGLPRSYQDESHNEPVGWDEPSGVGGSSGAGGQQSDKPTGLRHFSTKV

CEKVKEKGLTNYNEVADELVADYFQNNLIKQIDVVKQEYDMKNIRRRVYDALNVLLAMNIITKSKKDIRWIGLPASASQE

ISRLEEEKSRREASISSKKQALEEMVLQIVSYKNLVERNRKNEHKNGRPENDTVLHLPFLIINTDKEANVECSVSSDKSE

FLFSFDKKFEIHDDFEILKKLNLACSLETTNPTAEEVKTAKSFLPTLHQHYVDEIIANRKKVEAEKEEKRKQQQLIADQM

SMNLSQAQYVEPTSSLAQMSYSSRFNRQLQEHLINDGSEDRSAAAGIMERDYDMDKNVNQGSASRGPMYNTYSPQKIRAQ

VNTPLQVPPVTKRYYVQKTQGPMKHDMTPVVRTVNRPYSTVPPDRRLSTGATSVNSGPVKYYVPQGHQPMHQPVGQRYRV

RPQQPQMSHMGQPHQVQQRVVYPAGSISGHQLQPGQRIVTQRIVAPGGPHPPGTIVRKVIRKIVVNNPKQSPAQQVIQKK

MMEQDMCTFERKTEQPMTSAQAAALIQHPQPEEYDYFQ

>DP-Dd gi|66818451|ref|XP_642885.1| [Dictyostelium discoideum AX4]

MESTNQSQIANIPSSTASTINNNKNNNNNNNNNNNNNNNNNNNNNNNNNNNNNNNNNNNNNNNNNQSNSQHDDTINNTQE

IEDESPSQSPTYPNNSNNNNINSTSTNNNNNNNHNNNINSNSINEANNKLNESDDLNNSTFVNNLLTSLKTRSQSDLKQF

QQQQQQQQQQQQQQQQQQQNNNNINSHSPILSSQHSFSIPNQPIITPTQSQYINNSRNMQFDINDVHPEYFYSQLNSITQ

NNNNNNNNNNNNNNNNNVNNNNVNNNLSNQNNESEINNTASSSKKRSSSSLNTNNNNNNTNNSNNNNNNNNNNNNNNNNN

NNNNNNNNNNNNNNNNLSNDENDMDDKAKDEKSKGLRYLSYKVCQKVQSKKTTSYVEVSNELLSEYIEENRRNMGAGVSD

ASFKTNTVKRRIYDVLNVFQAMNIITKDKQKISWVGLPSGLPSQQLQQLRNSQEDNNNNNSNNNSNNTNNNNNNNNNNNN

NNNNNNNNNNNNNNNNNSTITPTPVIQTNQSIAKQLEDIKERIQAKKKQLKDLNQQEKDYQELHSRNTLLEQRKDQSLKN

KIFLPFIVLSTQNSTVIDCEVENERSKYFFNFSQSFNIIDDDSLVKSVLENPLSNNPSLGNNLPTTITNRSLSTPILPSQ

VSNEPKSNGRKKRKKDD

>Os03g0152100-Os gi|115450779|ref|NP_001048990.1| Os03g0152100 [Oryza sativa (japonica cultivar-group)]

MVSGVAHRPDDDGGRAASTFQRPPQPAGARPSLATPPPSGGAQSASTSGGSAGSPSSRSEQHVPAAAGMAAGAAAASTPI

SENTFLRLNDLDIHGDDAPSSQAPTSKKKKRGARAVGPDKGGRGLRQFSMKVCEKVESKGRTTYNEVADELVAEFADPNN

SILPPDPDNPNAQQYDEKNIRRRVYDALNVLMAMEIISKDKKEIQWKGLPRTSINDIEDLQTELVGLKSRIEKKNTYLQE

LQDQFVGMQKLIQRNEQLYGSGNIPSGGVALPFILVQTRPHATVEVEISEDMQLVHFDFNSTPFELHDDSFVLKAMSSCG

EEQIDGIHDLISNGGESSSMPNIYRQQVQQPARSTNGTARLPSSPPIPGILKGRVKHEH

>Os10g0440100-Os gi|115482194|ref|NP_001064690.1| Os10g0440100 [Oryza sativa (japonica cultivar-group)]

MVSGAAHSASTSGGGGGSEGSPTGRAAPGMQGGGSAATPAASASASTPASETTVARRLDGLDIQGDDAPSSQPATSKKKK

RGPGTRATGPDKGGRGLRQFSMKVCEKVESKGRTTYNEVADELVAEFADPNNNFASPDPDNPNTPQFDEKNIRRRVYDAL

NVLMAMDIISKDKKEIQWKGLPRTSMSDVEELKTEIIGLKGRIDKKNAYLQELEDQFVGLQNLAQRNEQLYGSGNAPSGG

VALPFILVQTRPHATVEVEISEDMQLVHFDFNSTPFELHDDSFVLKALGFSGKEPDDTQAWVGNGGECSTTPIYHQSPQV

ARPNGVRLPTSPPIPGILKGRVKHEH

>Os01g0678700-Os gi|115439161|ref|NP_001043860.1| Os01g0678700 [Oryza sativa (japonica cultivar-group)]

MAPPCGDAAAAASAAPGLANLLIREGAGLPSRPERYPPFRPCTSDSFAPISREGDDIPPQKKSVSLRSGGGGNAAEREEG

GANRNGKKEKTGAQRITGWGLREFSKIVSKKVEAKGRTTYNEVADEIFAELKSITQNGLEFDEKNIRRRVYDAFNVLIAI

RVIAKDKKEIKWMGLTNYRYEKIQKLEEVHKELITRIKNKKKLLQEIEKQFDDLQNITLRNQASQRPAESVNGILLPFLL

IKTSRKARVEIEISEDSKFARFDFNGAPFTMHDDVSILEAIRRNKGRAGLSIHP

>DPA-At gi|22326573|ref|NP_195867.2| DPA; transcription factor [Arabidopsis thaliana]

MSMEMELFVTPEKQRQHPSVSVEKTPVRRKLIVDDDSEIGSEKKGQSRTSGGGLRQFSVMVCQKLEAKKITTYKEVADEI

ISDFATIKQNAEKPLNENEYNEKNIRRRVYDALNVFMALDIIARDKKEIRWKGLPITCKKDVEEVKMDRNKVMSSVQKKA

AFLKELREKVSSLESLMSRNQEMVVKTQGPAEGFTLPFILLETNPHAVVEIEISEDMQLVHLDFNSTPFSVHDDAYILKL

MQEQKQEQNRVSSSSSTHHQSQHSSAHSSSSSCIASGTSGPVCWNSGSIDTR

>DPB-At gi|30680032|ref|NP_850757.1| DPB [Arabidopsis thaliana]

MTTTGSNSNHNHHESNNNNNNPSTRSWGTAVSGQSVSTSGSMGSPSSRSEQTITVVTSTSDTTFQRLNNLDIQGDDAGSQ

GASGVKKKKRGQRAAGPDKTGRGLRQFSMKVCEKVESKGRTTYNEVADELVAEFALPNNDGTSPDQQQYDEKNIRRRVYD

ALNVLMAMDIISKDKKEIQWRGLPRTSLSDIEELKNERLSLRNRIEKKTAYSQELEEQYVGLQNLIQRNEHLYSSGNAPS

GGVALPFILVQTRPHATVEVEISEDMQLVHFDFNSTPFELHDDNFVLKTMKFCDQPPQQPNGRNNSQLVCHNFTPENPNK

GPSTGPTPQLDMYETHLQSQQHQQHSQLQIIPMPETNNVTSSADTAPVKSPSLPGIMNSSMKPEN

>DP-Ot gi|116060017|emb|CAL56076.1| E2F dimerization partner 1 (IC) [Ostreococcus tauri]

MGEPRVGVAHAGASASTARYDRDAAEASMQAAAKYVIEARASIADARASLRARGDASARDEVDAMYSTPKASTSARKTPA

GQTPTTPSAQTPGEREQKGLRHFSMRVCEKVEEKMHTSYNEVANELVEELRLAAQQANTEFDEKNVRRRVYDALNVIEAV

GIITKKKKEIFWSGYPPGCMKPGELPPKRAGTTGTPEATTPRTAAPTWATSEAGIEEFEKKVNHLAELVEQHDAIIALVD

RNREAMAKSNGAPITGIQLPFILIQTKADAEVDVEISEDQRHVHLDFNQTPFQIHDGFHVLTKMIKQHPIRIPREDAGPS

GLDTTGKSTETNEEPAVKRKTTAASPLEENTGVTGKRTKKS
