# Supplementary figures and images for: The ancient function of RB-E2F Pathway: insights from its evolutionary history
Source: Biol Direct. 2010 Sep 20;5:55. doi: 10.1186/1745-6150-5-55 (PMC3224931; doi:10.1186/1745-6150-5-55)

A. E2F1-6 family

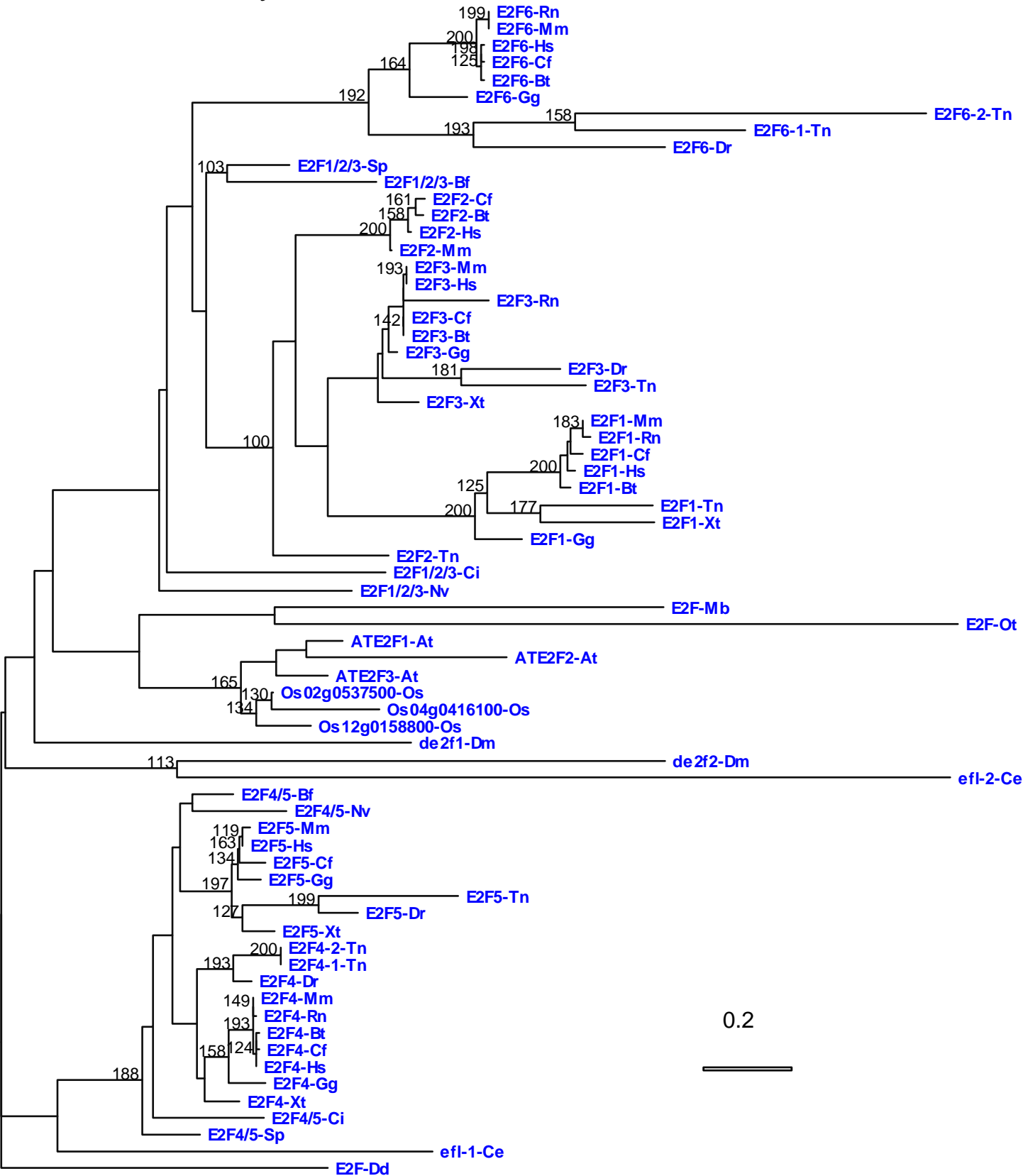

## B: E2F7/8 family

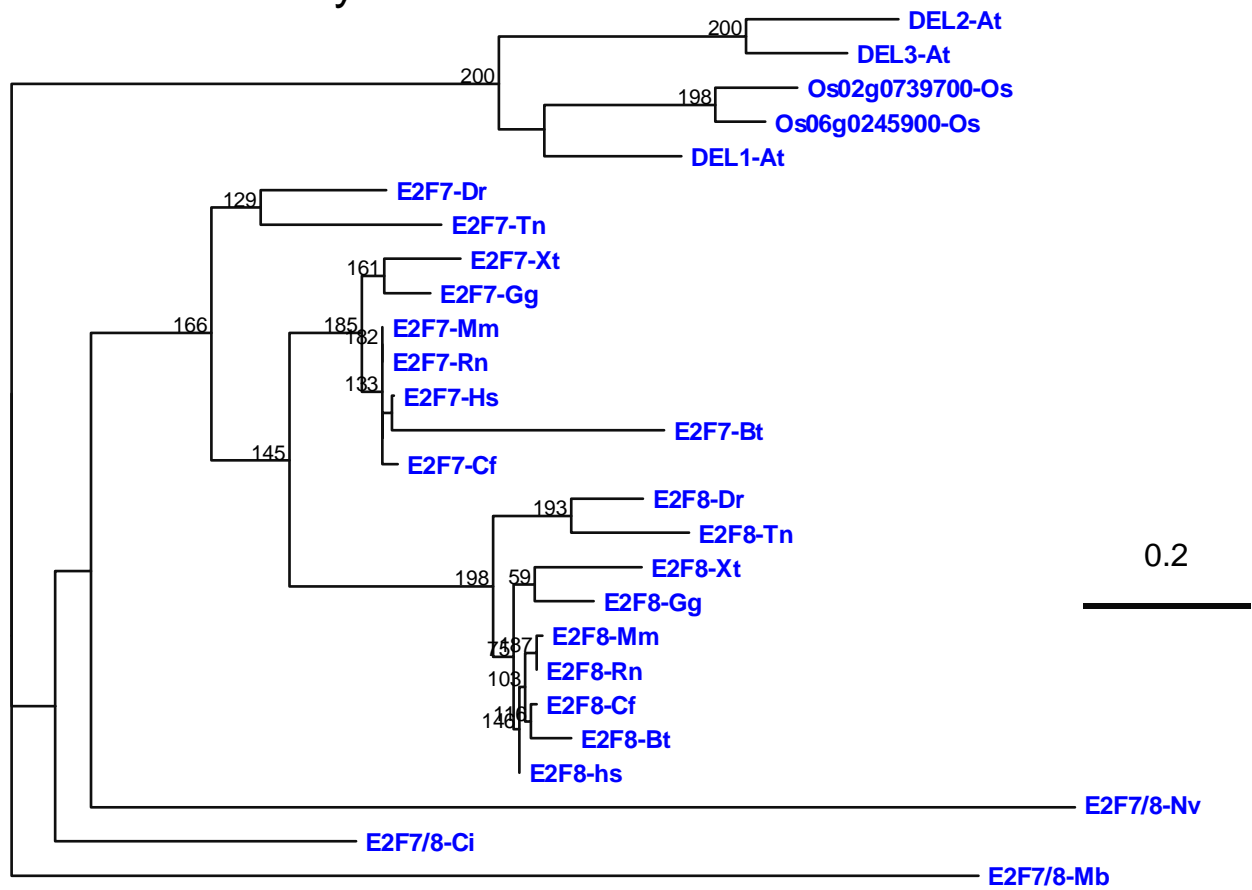

## C: RB family

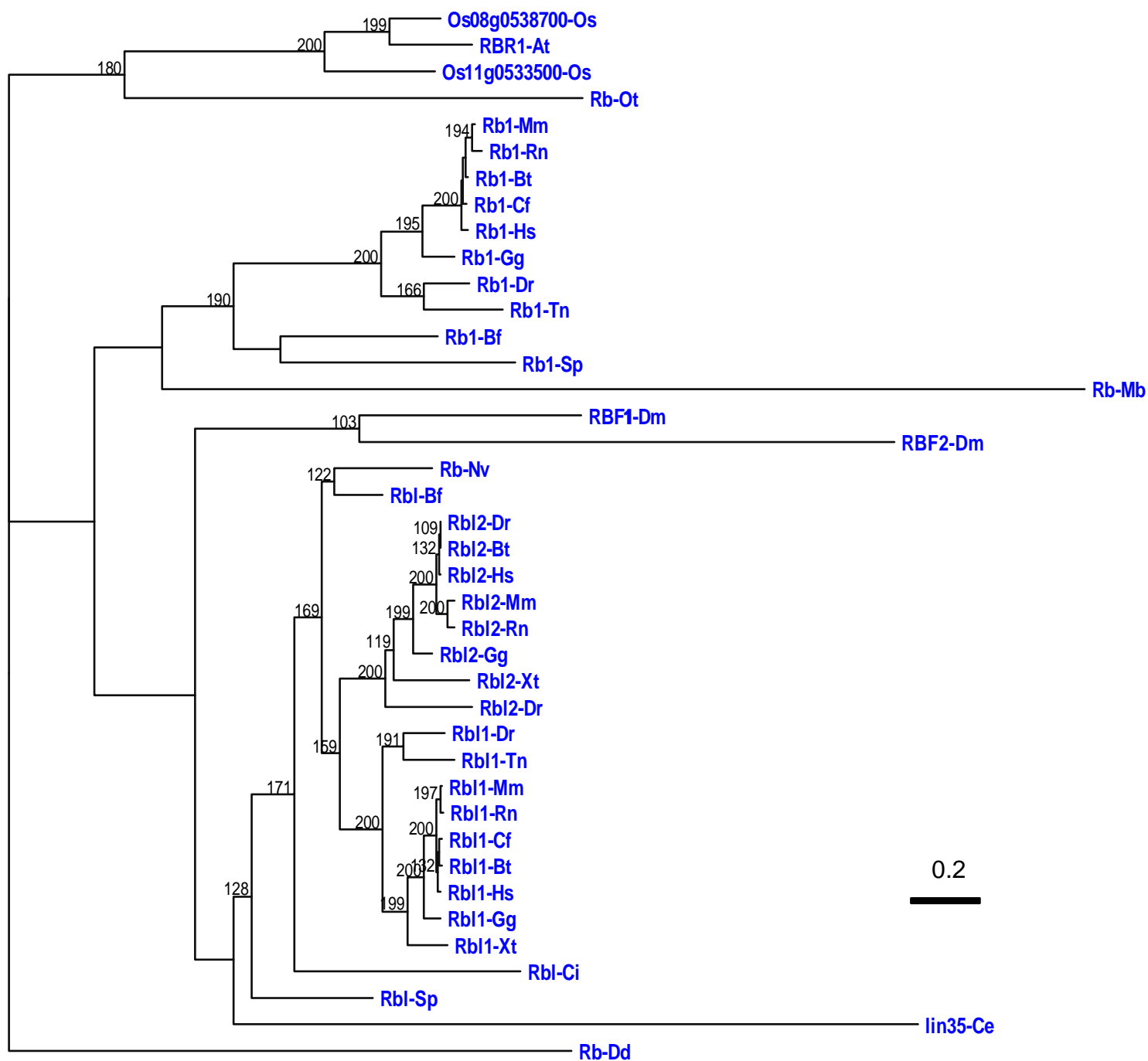

D: DP family

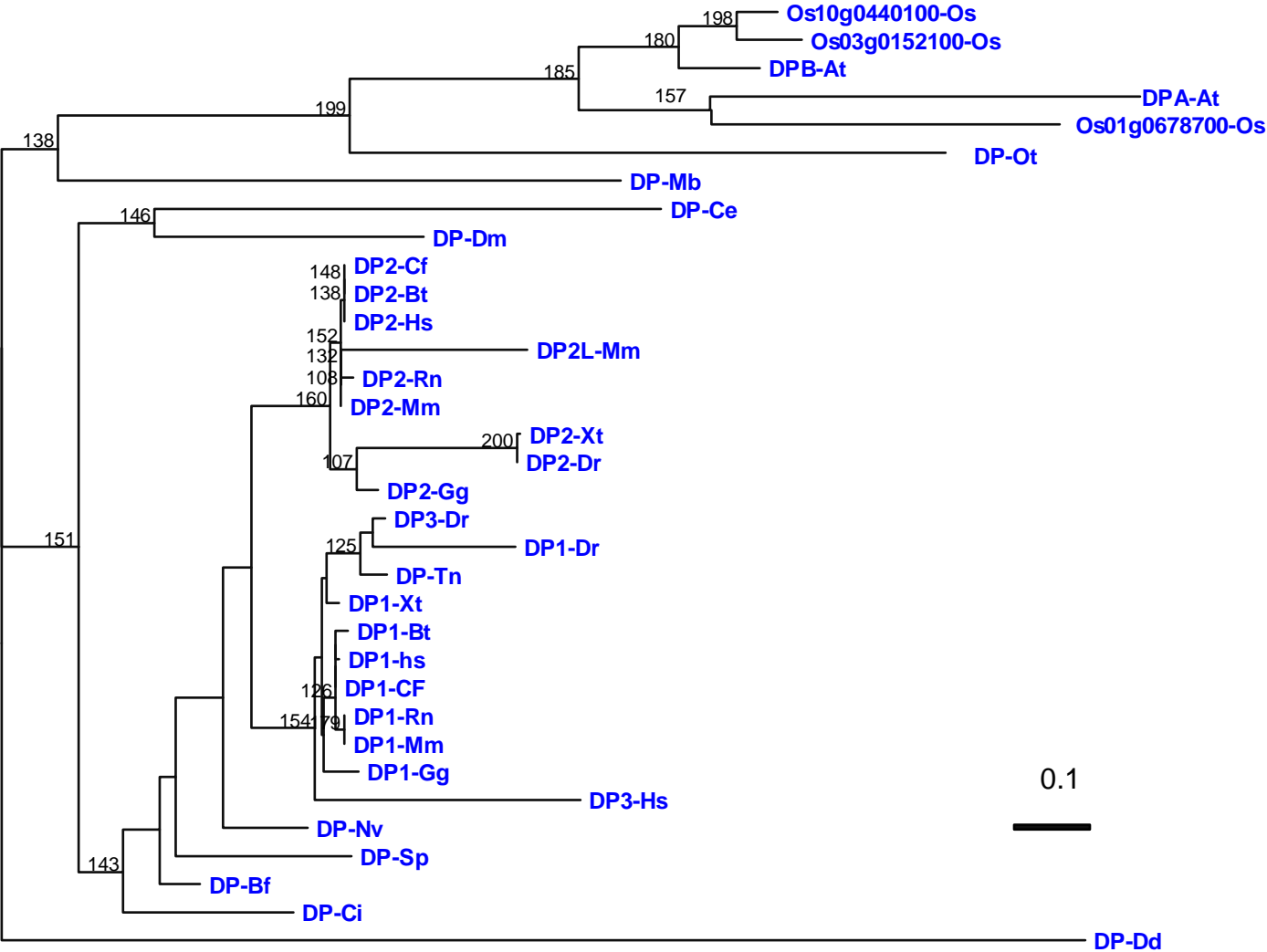

Supplement: Additional file 2 — Phylogenetic analyses of E2F1-6, E2F7/8, RB, and DP family in eukaryote. Maximum likelihood (ML) trees were constructed by using PHYML V.2.4 for E2F1-6, E2F7/8, RB, and DP family in eukaryota, with 200 bootstrap resamplings and JTT setting, [file 1745-6150-5-55-S2.PDF]
