# Supplementary material for: The ancient function of RB-E2F Pathway: insights from its evolutionary history
Source: Biol Direct. 2010 Sep 20;5:55. doi: 10.1186/1745-6150-5-55 (PMC3224931; doi:10.1186/1745-6150-5-55)
Supplement: Additional file 3 — Exon intron structures. The exon and intron structures of E2F and RB family genes from human, fly, and worm. [file 1745-6150-5-55-S3.PDF]

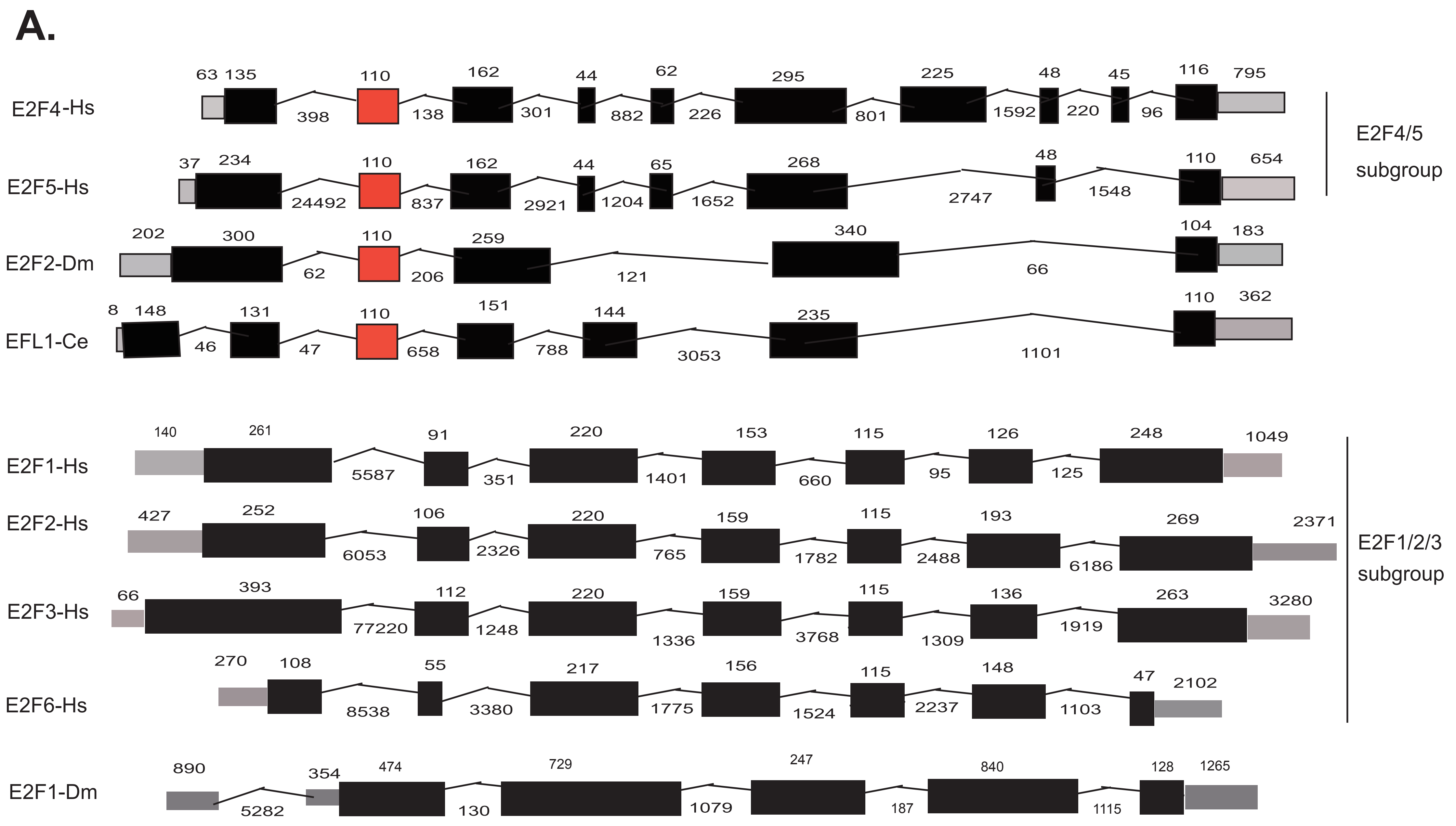
A.
E2F4-Hs
63 135 110 162 44 62 295 225 48 45 116 795
398 138 301 882 226 801 1592 220 96
E2F5-Hs
37 234 110 162 44 65 268 48 110 654
24492 837 2921 1204 1652 2747 1548
E2F2-Dm
202 300 110 259 340 104 183
62 206 121 66
EFL1-Ce
8 148 131 110 151 144 235 110 362
46 47 658 788 3053 1101
E2F4/5
subgroup
E2F1-Hs
140 261 91 220 153 115 126 248 1049
5587 351 1401 660 95 125
E2F2-Hs
427 252 106 220 159 115 193 269 2371
6053 2326 765 1782 2488 6186
E2F3-Hs
66 393 112 220 159 115 136 263 3280
77220 1248 1336 3768 1309 1919
E2F6-Hs
270 108 55 217 156 115 148 47 2102
8538 3380 1775 1524 2237 1103
E2F1/2/3
subgroup
E2F1-Dm
890 354 474 729 247 840 128 1265
5282 130 1079 187 1115

B

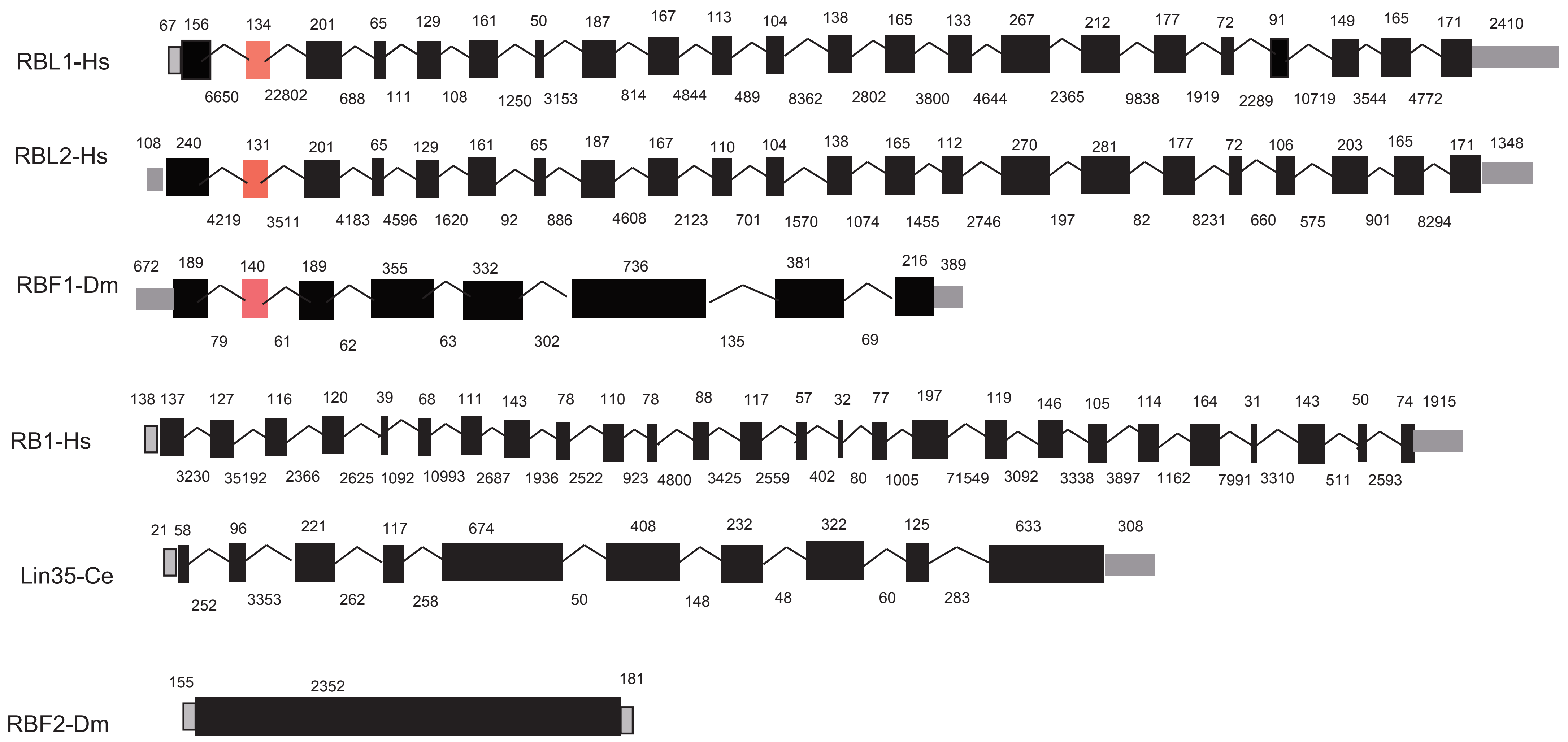
RBL1-Hs
67 156 134 201 65 129 161 50 187 167 113 104 138 165 133 267 212 177 72 91 149 165 171 2410
6650 22802 688 111 108 1250 3153 814 4844 489 8362 2802 3800 4644 2365 9838 1919 2289 10719 3544 4772
RBL2-Hs
108 240 131 201 65 129 161 65 187 167 110 104 138 165 112 270 281 177 72 106 203 165 171 1348
4219 3511 4183 4596 1620 92 886 4608 2123 701 1570 1074 1455 2746 197 82 8231 660 575 901 8294
RBF1-Dm
672 189 140 189 355 332 736 381 216 389
79 61 62 63 302 135 69
RB1-Hs
138 137 127 116 120 39 68 111 143 78 110 78 88 117 57 32 77 197 119 146 105 114 164 31 143 50 74 1915
3230 35192 2366 2625 1092 10993 2687 1936 2522 923 4800 3425 2559 402 80 1005 71549 3092 3338 3897 1162 7991 3310 511 2593
Lin35-Ce
21 58 96 221 117 674 408 232 322 125 633 308
252 3353 262 258 50 148 48 60 283
RBF2-Dm
155 2352 181

The exon and intron structures of E2F and RB family genes from human, fly, and worm

(A). E2F1-6 family; (B). RB family, Boxes correspond to exons. Non-coding exons are shown in grey. The size of introns and exons in nucleotides is shown. Introns are not drawn to scale. The intron and exon structure information are got from ensemble data base, the detail of transcripts used for analysis are: E2F1-Hs: ENST00000343380, 7 exons; E2F2-Hs: ENST00000361729, 7 exons; E2F3-Hs: ENST00000346618, 7exons; E2F4-Hs: ENST00000379378, 10 exon; E2F5-Hs: ENST00000416274, 8 exon; E2F6: ENST00000381525, 7 exons; E2F1-Dm:FBtr0084119 6 exons; E2F2-Dm: FBtr0081501, 5 exons; EFL1-Ce: Q9XX87_CAEEL (Y102A5C.18) 7 exon; RBL1-Hs: ENST00000373664 22 exons; RBL2-Hs: ENST00000262133 22 exons; RB1-Hs: ENST00000267163, 27 exons; RBF1-Dm: FBtr0070146, 8 exons; RBF2-Dm: FBtr0083213, 1 exon; Lin35-Ce: O01682_CAEEL (C32F10.2), 10 exons;
