# Supplementary material for: The ancient function of RB-E2F Pathway: insights from its evolutionary history
Source: Biol Direct. 2010 Sep 20;5:55. doi: 10.1186/1745-6150-5-55 (PMC3224931; doi:10.1186/1745-6150-5-55)
Supplement: Additional file 4 — Predicted ancestral sequences of E2F and RB. Detail ancestral sequences of E2F and RB predicted by Gapped Ancestral Sequence Prediction program (GASP) program [file 1745-6150-5-55-S4.DOC]

**Predicted acestral sequeces of E2F and RB**

>Predicted acestral sequeces of E2F

MNTEIIRQNTIIPLTPDITTNNNIVHSNNNNNNNKMTKSKQDDNFDNSNNSNNSNNNKNNKNILPTTRNRNVNSQQKVGN

GNGNGNSLADEAASGYGTGNSLLSLDPLSQIQSMASLQSPTFLNKSSPNPTPNISLPQPTISTTANPTSPPSSAITAATT

GTTNTNTSTTSTTANNNNNNNNNNLMEDKYIDMSLPGFRQLKKSELQKALSSSSSAGTTSSSMPPSTDNADNYSPQEPSP

SSSNSSISPSTTPTSSQQLTSPQESNLSMAKGQTTKTALTLGTTNLTSTTTTRKRRSTSSVNNTTGAEQQKTEDGPPPPA

GTTTTTTTTKPKGAPKRQTKRSKFKFDLNSEEESDPSSQLSSSSSSTTTTTQSSQPINDDQEDDDDDDDYDNSQASTTSN

GPGGGGGNKKKTKSTVGNRFDKSLGLLTKKFLDLIEYSPNGVLDLKVAAEKLEIQKRRIYDITCVLEGIGLIEKCSKNQI

LWKGGGFDLNGKEGKRGQQQHQKQPLDPKEQDNFKKELKKLEEKEESLDQRIKKAQKNIRNMLYEPKNSKLMYVTHDDLR

NIEGFKGDTVIAIQAPSGTQLQIPDPDEGEEPGQRRYQILLKNETNGPIDVFLLNQTELHPTDSIKLPTPPDNLQPEYDD

QDIELNLRLPPAENQNPNERNKEPGNNPEEAATMDTTEEEYSYARNRITNWLGGMPNSPAYLDNNNNNNNNNNNNNNNNN

NNNNNNNNNNSILSPSKQIQQQTNYLQPSQNVSYWGGNFFLGKYFPFFPPYNHQKTFLPPGSASSSLSSNMLFPPYPGQF

ANFYPAFPFIYNPYLLCLEPPFEQSDEYYFESLGDSEGISELYDGESFLPPSFDDFGNQSIES

>predicted acestral sequeces of RB

MMAHNKNDTNNTKTTATTTTTANNNNNMNGNNNGKDDGGGMNARGDCDGKGDCGAAGRYTSAVTGSGAVDDTKAGDCKSV

TSANKVACSCGNKKSSYNNSSSSDNNSKKGRKRKTNNSKNKNNDSNTTTDTDTNNNNSNKNSSNNNVSRSCNSGGDSDNS

VSSKAYKSDRSVSNRNAYRSNKVYRKYRTHACNGKTTHNYNNYSNNNNNNTNNNNNNNNNNRTNGRDRGWCKNKKKSDVS

SHCAYVNARKTSKNVMKTGDVGAKSKDDGKTVNCYVAKANNKDKANNACRNNNNGSDDTASGMYYRVDNYSNKSYYYAGD

DRSDDGDYTNNHGNNNNNNNNNKMDAYYRCNNNNNNYRTDSTTSRYSSRGGGGGGNRKNYGTSKSSAGNGKVVTSATASW

KDGRDNSNASCTSDKVKRVSTNRGCSGGDTTMVDVVRRKNMAVKYYKKASVSNNANHDHKSACSMAVYKTWYWNVGHDKV

KVDMDRVHSKRYAWSKGSTDKSSRGSNTGAAATRSRVSMNDGNRNNTNNTKRNAVAGGVRCAKKRCTMGSSDYVVYVMKD

TKNRCHRVRNSATVSSTGTRRSSAVTRNNGNNGNNNANGNAKVNKANRRNNCNRKSYRKYAHVHVNKTNRHDCCVYGVKV

NGGCKGAKTYNYRNNSNNVTNKRSVVWGSTKGVNYNSSSRNGGVSMKHGDSYNKVKSVKNRANKKKARGSNNNSRRNNKN

NHNVYVSRNKNANDSNSNNYSCTGNTKTGGKHGGRSSSSSSSTNDANNVDKGVDDDGGKNSSSRSSSSSSSSRRSKGRKT

DKS
