# Supplementary material for: The ancient function of RB-E2F Pathway: insights from its evolutionary history
Source: Biol Direct. 2010 Sep 20;5:55. doi: 10.1186/1745-6150-5-55 (PMC3224931; doi:10.1186/1745-6150-5-55)
Supplement: Additional file 5 — The codoning sequences alignments for E2F and RB family proteins. The codoning sequences alignments for E2F and RB family proteins used in computing Ka/Ks in Figure 5 and Figure 6. [file 1745-6150-5-55-S5.DOC]

**The Codon sequences alignments for E2F and RB family proteins**

**1. The Codon sequences alignment For E2F1 proteins (Nuc format)**

5 1311

E2F1-Hs

AUGGCCUUGGCCGGGGCCCCUGCGGGCGGCCCAUGCGCGCCGGCGCUGGAGGCCCUGCUC

GGGGCCGGCGCGCUGCGGCUGCUCGACUCCUCGCAGAUCGUCAUCAUCUCCGCCGCGCAG

GACGCCAGCGCCCCGCCGGCUCCCACCGGCCCCGCGGCGCCCGCCGCCGGCCCCUGCGAC

CCUGACCUGCUGCUCUUCGCCACACCGCAGGCGCCCCGGCCCACACCCAGUGCGCCGCGG

CCCGCGCUCGGCCGCCCGCCGGUGAAGCGGAGGCUGGACCUGGAAACUGACCAUCAGUAC

CUGGCCGAGAGCAGUGGGCCAGCUCGGGGCAGAGGCCGCCAUCCAGGAAAAGGUGUGAAA

UCCCCGGGGGAGAAGUCACGCUAUGAGACCUCACUGAAUCUGACCACCAAGCGCUUCCUG

GAGCUGCUGAGCCACUCGGCUGACGGUGUCGUCGACCUGAACUGGGCUGCCGAGGUGCUG

AAGGUGCAGAAGCGGCGCAUCUAUGACAUCACCAACGUCCUUGAGGGCAUCCAGCUCAUU

GCCAAGAAGUCCAAGAACCACAUCCAGUGGCUGGGCAGCCACACCACAGUGGGCGUCGGC

GGACGGCUUGAGGGGUUGACCCAGGACCUCCGACAGCUGCAGGAGAGCGAGCAGCAGCUG

GACCACCUGAUGAAUAUCUGUACUACGCAGCUGCGCCUGCUCUCCGAGGACACUGACAGC

CAGCGCCUGGCCUACGUGACGUGUCAGGACCUUCGUAGCAUUGCAGACCCUGCAGAGCAG

AUGGUUAUGGUGAUCAAAGCCCCUCCUGAGACCCAGCUCCAAGCCGUGGACUCUUCGGAG

AACUUUCAGAUCUCCCUUAAGAGCAAACAAGGCCCGAUCGAUGUUUUCCUGUGCCCUGAG

GAGACCGUAGGUGGGAUCAGCCCUGGGAAGACCCCAUCCCAGGAGGUCACUUCUGAGGAG

GAGAACAGGGCCACUGACUCUGCCACCAUAGUGUCACCACCACCAUCAUCUCCCCCCUCA

UCCCUCACCACAGAUCCCAGCCAGUCUCUACUCAGCCUGGAGCAAGAACCGCUGUUGUCC

CGGAUGGGCAGCCUGCGGGCUCCCGUGGACGAGGACCGCCUGUCCCCGCUGGUGGCGGCC

GACUCGCUCCUGGAGCAUGUGCGGGAGGACUUCUCCGGCCUCCUCCCUGAGGAGUUCAUC

AGCCUUUCCCCACCCCACGAGGCCCUCGACUACCACUUCGGCCUCGAGGAGGGCGAGGGC

AUCAGAGACCUCUUCGACUGUGACUUUGGGGACCUCACCCCCCUGGAUUUC

E2F1-Bt

AUGGCCGUGGCCGGGGCCCCCGCGGGCGGCUCUUGCGCGCCGGCGCUGGAGGCCCUGCUC

GGGGCCGGCGCGCUGCGGCUGCUCGACUCCUCGCAGAUCGUCAUCAUCUCCACCGCGCAG

GACGCCAGCGCCCCGCCGGCCCCCGCCGGCCCCGCCGCACCCGCCGCCGGCCCCCGGGAC

CCUGACCUGCUGCUCUUCGCCACGCCGCAGGCGCCCCGGCCCACACCCAGCGCGCCGCGC

CCCGCGCUCGGCCGCCCGCCGGUGAAGCGGAGGCUGAACCUGGAAACUGACCAUCAAUAC

CUGGCUGAGAGCAGCGGGCCAGCUCGGGGCAGAGGCCGCCACCCAGGAAAAGGUGUGAAG

UCCCCAGGGGAGAAGUCACGCUACGAAACAUCAUUGAACCUGACCACCAAACGCUUCUUG

GAAUUACUGAGCCGCUCGGCUGAUGGAGUGGUCGACUUGAACUGGGCGGCAGAGGUGCUG

AAGGUGCAGAAACGGCGCAUCUACGACAUCACCAACGUCCUGGAGGGCAUCCACCUCAUC

GCGAAGAAGUCCAAGAACCACAUCCAGUGGCUAGGCAGCCAUGCAACGGUGGGGAUCAGC

GGGCGGCUUGAAGGAUUGACCCAGGACCUCCAGCAGCUGCAGGAGAGUGAGCAGCAGCUG

GAUCACCUGCUCCACACCUGCAGCACCCAGCUGCGUCUGCUCUCGGAGGACGCUGACAGC

CAGCGCCUGGCCUAUGUGACCUGCCAGGACCUUCGUAGCAUCGCAGACCCUGCAGAGCAG

AUGGUCAUGGUGAUCAAGGCCCCCCCUGAGACCCAGCUCCAAGCCGUGGACUCCUCGGAG

AACUUUCAGAUCUCCCUUAAGAGCAAACAAGGCCCCAUCGACGUUUUCCUGUGCCCUGAG

GAGAGUGUGGGUGGAACCAGCCCUGGAAAGACCCCGUCCCAGGGGGCAGCUUCGGGGGAG

GAGGACAGGACAGCUGACCUUGCCACUGCGGUGCCACCACCACCAUCAUCACCCCGCUCA

UCCCCUGCCACAGAUCCCAGUCAGUCCCUGCUCAGCCUGGAGCAAGAACCUCUGCUUUCC

CGGAUGGGCGGCCUGCGGGCCCCCGUGGACGAGGACCGCCUAUCCCCGCUCGUGGCGGCC

GACUCACUCCUGGAGCACGUGAAGGAGGACUUCUCCAGCCUCCUCCCCGAGGAGUUCAUC

ACCCUGUCCCCCCCCCACGAGGCCCUCGACUACCACUUUGGCCUCGAGGAGGGUGAGGGC

AUCAGAGACCUCUUCGACUGUGACUUUGGGGACCUCACUCCCCUGGAUUUC

E2F1-Cf

AUGGCCGUGGCCGGGGCCCCCGCAGGCGGCCCAUGCGCGCCGGCGCUGGAGGCCCUGCUC

GGGGCCGGCGCGCUGCGGCUGCUCGACUCCUCGCAGAUCGUCAUCAUCUCCACGGCGCAG

GACGCCAGCGCCCCGCCGGCCCCCGCCGGCCCGGCCGCGCCGGCCGCUGGCCCCCGCGAC

CCCGACCUGCUGCUCUUCGCCACGCCGCAGGCGCCCCGGCCCACACCCAGCGCGCCGCGC

CCUGCGCUCGGCCGCCCGCCGGUAAAGCGGAGGUUGGACCUGGAAACUGACCAUCAGUAC

CUGGCUGAGAGCAGUGGGCCAGCCCGGGGCAGAGGCCGCCACCCAGGAAAAGGUGUGAAA

UCCCCAGGGGAGAAGUCACGCUAUGAGACCUCACUGAAUCUGACCACAAAACGCUUCCUG

GAGCUGCUGAGCCGCUCGGCUGAUGGCGUUGUUGAUCUGAACUGGGCGGCUGAGGUGUUG

AAGGUGCAGAAACGGCGCAUCUAUGACAUCACCAACGUCCUCGAGGGCAUCCAGCUCAUU

GCCAAGAAGUCUAAGAACCACAUCCAGUGGCUAGGCAGCCAUGCAGCAGUGGGGAUCAGU

GGGCGGCUUGAAGGACUGACUCAGGACCUCCGGCAGCUGCAGGAGAGUGAGCGGCAGCUG

GACCACCUGAUCCAUAUCUGCACCACACAACUGCGGCUGCUCGCUGAGGACUCCGACAGC

CAGCGCCUGGCCUACGUGACCUGCCAGGACCUUCGUAGCAUCGCAGACCCUGCAGAGCAG

AUGGUCAUGGUGAUCAAGGCCCCUCCUGAGACCCAGCUCCAAGCCAUAGACUCCUCAGAG

ACCUUUCAGAUCUCCCUUAAGAGCAAACAAGGCCCCAUUGACGUUUUCCUGUGCCCUGAG

GAGAGUGCAGGCGGGAUCAGCCCUGGGAAGACCCCAUCCCAGGGGACAGCUUCUGGGGAG

GAAGACAGGCCAGUUGACCCUGCCACCACAGUG---CCACCACCAUCAUCUCCCCCCUCA

UCCCCUGCUUCGGAUCCCAGCCAGUCCCUGCUCAGCCUGGAGCAAGAACCUCUGCUUUCC

CGGAUGGGUGGCCUGCGGGCCCCCGUGGACGAGGACCGCCUGUCCCCGCUGGUGGCGGCC

GACUCGCUCCUGGAACAUGCACGGGAGGAUUUUUCCGGCCUCCUCCCUGAGGAGUUCAUC

AGCCUGUCCCCACCCCACGAGGCUCUCGACUACCACUUUGGCCUUGAGGAGGGUGAGGGC

AUCAGAGACCUCUUCGACUGUGACUUUGGGGACCUUACCCCUCUGGAUUUC

E2F1-Rn

AUGGCCGUAGCC------CCCGCGGGCGGCCAGCACGCGCCGGCGCUGGAGGCCCUGCUC

GGGGCGGGCGCGCUGCGGCUGCUCGACUCCUCGCAGAUCGUCAUCAUCUCCACCGCGCCC

GAUGUCGGCGCCCCGCAGGUCCCCACCGGCCCCGCCGCGCCGCCCGCUGGCCCUCGAGAU

CCUGACGUGCUGCUCUUCGCAACGCCGCAGGCGCCCCGACCCGCGCCUAGUGCACCGCGC

CCGGCUCUCGGCCGCCCGCCGGUGAAACGGAGGCUGGAUCUGGAAACUGACCAUCAGUAC

CUUGCUGGUAGCAGCGGGCCAUUCCGGGGCAGAGGCCGCCACCCAGGGAAAGGUGUGAAA

UCUCCAGGGGAGAAGUCACGCUAUGAGACCUCACUAAAUCUGACCACCAAACGCUUCUUG

GAGCUGCUGAGCCACUCAGCUGAUGGUGUUGUUGAUCUGAACUGGGCAGCCGAGGUGCUG

AAGGUGCAGAAACGACGCAUCUAUGACAUCACCAAUGUCCUGGAGGGCAUCCAACUCAUU

GCCAAGAAGUCCAAGAAUCAUAUCCAGUGGCUAGGCAGCCGCACCAUGGUGGGGAUCGGU

CAGCGGCUUGAAGGCCUGACCCAGGACCUGCAACAACUGCAGGAGAGUGAGCAGCAGCUG

GAUCACCUGAUGCACAUCUGUACCACUCAGCUGCAACUGCUUUCUGAGGACUCAGACAUC

CAGCGCCUGGCCUAUGUGACCUGCCAAGAUCUCCGCAGCAUUGCAGACCCUGCAGAACAA

AUGGUCAUAGUGAUCAAGGCCCCUCCUGAGACCCAACUACAAGCUGUGGAUUCUGCAGAG

ACAUUUCAGAUCUCCCUUAAGAGCAAACAAGGCCCCAUCGAUGUUUUCCUGUGCCCUGAG

GAAAGUGCAGAGGGGAUUAGCCCUGGGAGGACCUCAUACCAGGAGACAUCU---GGGGAG

---GACAGGAAUGCUGACUCUGGCACAGCAGGG---CCUCCACCAUCACCUCCCUCCACA

UCCCCAACCUUGGAUCCCAGCCAGUCCCUGUUAGGCCUGGAGCAAGAAGCUGUAUUGCCU

CGAAUAGGCAACCUGAGGGCCCCCAUGGAAGAAGACCGGUUGUCACCACUGGUGGCUGCU

GACUCACUCCUGGAGCAUGUUAAAGAAGACUUCUCUGGGCUCCUCCCUGGGGAGUUCAUC

AGCCUUUCCCCACCCCAUGAGGCUGUUGACUAUCACUUUGGUCUCGAGGAGGGUGAGGGC

AUUAGAGAUCUCUUUGACUGUGACUUUGGGGACUUGACCCCUCUGGAUUUC

E2F1-Mm

AUGGCCGUAGCC------CCCGCGGGCGGCCAGCACGCGCCAGCGCUGGAGGCCCUGCUC

GGGGCGGGCGCGUUGCGGCUGCUCGACUCCUCGCAGAUCGUCAUCAUCUCCACCGCGCCC

GAUGUCGGCGCCCCGCAGCUCC---------CCGCCGCGCCGCCCACUGGCCCUCGCGAU

UCUGACGUGCUGCUCUUCGCCACGCCGCAGGCGCCCCGACCCGCGCCUAGUGCACCGCGC

CCGGCUCUCGGCCGCCCGCCGGUGAAACGGAGGCUGGAUCUGGAGACUGACCAUCAGUAC

CUCGCUGGUAGCAGUGGGCCAUUCCGGGGCAGAGGCCGCCACCCAGGGAAAGGUGUGAAA

UCUCCGGGGGAGAAGUCACGCUAUGAAACCUCACUAAAUCUGACCACCAAACGCUUCUUG

GAGCUGCUGAGCCGCUCAGCUGACGGUGUCGUUGACCUGAACUGGGCAGCUGAGGUGCUG

AAGGUGCAGAAACGGCGCAUCUAUGACAUCACCAAUGUCCUGGAGGGCAUCCAGCUCAUU

GCCAAGAAGUCCAAGAAUCAUAUCCAGUGGCUAGGCAGCCACACCAUGGUGGGGAUUGGU

AAGCGGCUUGAAGGCCUGACCCAGGACCUGCAGCAACUGCAGGAGAGUGAGCAGCAGCUG

GAUCACCUGAUGCACAUCUGUACCACACAGCUGCAACUGCUUUCGGAGGACUCCGACACC

CAGCGCCUGGCCUAUGUGACCUGCCAGGACCUUCGCAGCAUUGCAGACCCUGCAGAACAG

AUGGUCAUAGUGAUCAAGGCCCCUCCUGAGACCCAACUACAAGCUGUGGAUUCUUCAGAG

ACAUUUCAGAUCUCCCUUAAGAGCAAACAAGGCCCCAUUGAUGUUUUCCUGUGCCCGGAG

GAGAGUGCAGACGGGAUUAGCCCUGGGAAGACCUCAUGCCAGGAGACAUCCUCUGGGGAG

---GACCGGACUGCAGACUCUGGCCCAGCAGGG---CCUCCACCAUCACCUCCCUCCACA

UCCCCAGCCUUGGAUCCCAGUCAAUCCCUGUUGGGCCUGGAGCAAGAAGCAGUAUUGCCA

CGGAUGGGCCACCUGAGGGUCCCUAUGGAAGAGGACCAACUGUCACCACUGGUGGCUGCU

GACUCACUCCUGGAGCAUGUUAAAGAAGACUUCUCUGGGCUCCUCCCUGGGGAGUUCAUC

AGCCUCUCCCCACCCCACGAGGCCCUUGACUAUCACUUUGGUCUCGAGGAGGGUGAGGGC

AUUAGAGAUCUCUUUGACUGUGACUUUGGGGACCUGACCCCUCUGGAUUUC

**2. The Codon sequences alignment For E2F2 proteins (Nuc format)**

4 1335

E2F2-hs

ATGCTGCAAGGGCCCCGGGCCTTGGCTTCGGCCGCTGGGCAGACCCCGAAGGTGGTGCCC

GCGATGAGCCCCACAGAGCTGTGGCCATCCGGCCTCAGCAGCCCCCAGCTCTGCCCAGCT

ACT------GCTACCTACTACACACCGCTGTACCCGCAGACGGCGCCTCCCGCAGCGGCG

CCAGGCACCTGCCTCGACGCCACTCCCCACGGACCCGAGGGCCAAGTTGTGCGATGCCTG

CCGGCAGGCCGGCTGCCGGCCAAAAGGAAGCTGGATCTGGAGGGGATTGGGAGGCCCGTC

GTCCCTGAGTTCCCAACCCCCAAGGGGAAGTGCATCAGAGTGGATGGCCTCCCCAGCCCC

AAAACCCCCAAATCCCCCGGGGAGAAGACTCGGTATGACACTTCGCTGGGGCTGCTCACC

AAGAAGTTCATTTACCTCCTGAGCGAGTCAGAGGATGGGGTCCTGGACCTGAACTGGGCC

GCTGAGGTGCTGGACGTGCAGAAGCGGCGCATCTATGACATCACCAACGTGCTGGAAGGC

ATCCAGCTCATCCGCAAGAAGGCCAAGAACAACATCCAGTGGGTAGGCAGGGGAATGTTT

GAAGACCCCACCAGACCTGGGAAGCAGCAACAGCTGGGGCAGGAGCTGAAGGAGCTGATG

AACACGGAGCAGGCCTTGGACCAGCTCATCCAGAGCTGCTCTCTGAGCTTCAAGCACCTG

ACTGAGGACAAGGCCAACAAGAGGCTGGCCTATGTGACTTACCAGGATATCCGTGCTGTT

GGCAACTTTAAGGAGCAGACAGTGATTGCCGTCAAGGCCCCTCCGCAGACGAGACTGGAA

GTGCCCGACAGGACTGAGGACAACCTGCAGATATATCTCAAGAGCACCCAAGGGCCCATC

GAAGTCTACCTGTGCCCAGAGGAGGTGCAGGAGCCGGACAGTCCTTCCGAGGAGCCTCTC

CCCTCTACCTCCACCCTCTGCCCCAGCCCTGACTCTGCCCAGCCCAGCAGCAGCACCGAC

CCTAGCATCATGGAGCCCACAGCATCCTCA------GTGCCAGCACCAGCGCCAACCCCC

CAGCAGGCCCCACCGCCT------------CCATCCCTGGTCCCCTTGGAGGCTACTGAC

AGCCTGCTGGAGCTGCCGCACCCACTCCTGCAGCAGACTGAGGACCAGTTCCTGTCCCCG

ACCCTGGCGTGCAGCTCCCCTCTGATCAGCTTCTCCCCATCCTTGGACCAGGACGACTAC

CTGTGGGGCTTGGAGGCGGGTGAGGGCATCAGCGATCTCTTCGACTCCTACGACCTTGGG

GACCTGTTGATTAAT

E2F2-Mm

ATGCTGCGCGCGCCGCGGACCCTGGCTCCGGCCACGGCGCAACCTACAAAGAGCTTGCCG

GCGCTGAACCCCACCGAGCTGTGGCCTTCGGGTCTGAGCAGCCCCCAGCTCTGCCCGGCC

ACCACCGCCACCACCTACTACACTTCGCTTTACACGCAGACGGTGCCTTCCTCTGTGGCG

CTGGGCACCTGCCTCGACGCCACTCCCCACGGACCCGAGGGCCAAATTGTGCGATGTGCA

CCCGCAGGCCGGCTGCCGGCCAAAAGGAAGTTGGACCTGGAGGGCATTGGGAGGCCTACG

GTCCCTGAATTCCGGACCCCCAAGGGGAAGTGCATCCGCGTGGATGGTTTGCCAAGCCCC

AAAACCCCCAAGTCTCCTGGGGAGAAGACACGCTATGACACGTCGCTGGGGCTCCTGACC

AAGAAGTTCATTTACCTCCTGAGCGAGTCGGAGGATGGAGTCCTGGACCTGAACTGGGCA

GCCGAGGTGCTGGATGTGCAAAAGCGGCGCATCTATGACATCACCAACGTGCTGGAGGGT

ATCCAGCTCATCCGCAAGAAGTCCAAAAACAACATCCAGTGGGTAGGCAGGGAACTATTT

GAAGACCCCACCCGACCCTCCAGGCAGCAGCAGTTGGGGCAGGAGCTGAAGGAGCTGATG

AATGCCGAGCAGACCTTGGACCAGCTCATTCAGAGTTGCTCCCTGAGCTTCAAGCACCTG

ACCGAAGATAATGCCAACAAGAAACTGGCCTATGTGACCTACCAGGATATCCGTGCCGTA

GGCAACTTCAAGGAGCAGACAGTGATTGCGGTCAAGGCCCCACCACAGACAAGATTGGAA

GTGCCGGACAGGGCCGAGGAGAACCTGCAGATTTATCTAAAGAGTACCCAAGGCCCCATT

GAAGTCTACCTGTGCCCAGAGGAGGGGCAGGAGCCAGACAGTCCTGCCAAGGAGGCGCTC

CCCTCCACCTCTGCCCTCAGCCCCATTCCTGACTGCGCTCAGCCGGGCTGCAGCACTGAC

TCTGGGATCGCAGAGACCATAGAGCCTTCA------GTACTGATACCCCAGCCGATACCA

CCGCCTCCTCCACCACCACTGCCGCCAGCCCCATCCCTCGTCCCCTTGGAAGCCACTGAC

AACATGCTGGAGCTGTCACACCCTCTTCTACAACAGACTGAGGACCAGTTCCTGTCCCCA

ATCCTGGCGGCCAACTCCCCCCTGATCAGCTTCTCCCCGCCCTTGGACCAGGACGAATAC

CTGTGGGGCATGGACGAGGGGGAAGGCATCAGTGACCTCTTCGACTCCTATGACCTTGGG

GACCTGTTGATTAAT

E2F2-Cf

CTGCACCGAGGGACACAAACCAGCCTCCCAGCCCGGTGGGCGGGCTGCAAGGCACTGCCC

GCGATGAGCCCTACCGAGCCGTGGCCGCCCGGCCTCAGCAGCCCCCAGCTCTGCCCGGCC

ACC------GCCACCTACTGCACCTCGCTGTACCCGCAGACCGTGCCTCCCCCTGCGGCG

GCCGGCACCTGCCTCGACGCCACCCCGCACGGACCCGAGGGCCAAGCGGTGCGCTGCGTG

CCGGCTGGCCGGCTGCCGGCCAAAAGGAAGCTGGACCTGGAGGGGATCGGGAGGCCAGCC

ATCCCTGAATTCCGGACTCCCAAGGGGAAGTGCATTAGAGTGGAGGGCCTTCCCAGCCCC

AGAACCCCCAAGTCCCCTGGAGAGAAGACTCGGTATGACACGTCGCTGGGGCTGCTCACG

AAGAAGTTCATTTACCTCCTGAGTGAATCCAAGGATGGGGTCCTGGACCTGAACTGGGCG

GCCGAGGTGCTGGACGTGCAGAAGCGGCGCATCTACGACATCACCAATGTGCTGGAGGGT

ATCCAGCTCATCCGTAAGAAGGCCAAGAACAACATCCAGTGGGTAGGCAGGGGAATGTTC

GAAGACCCCACCCGGCCTGGGAAGCAGCAGCAGCTGGGGCAGGAGCTGAAGGAGCTGATG

AGCATGGAGCAGGCCTTGGACCAGCTCATCCAGAGCTGCTCCCTGAACTTCAAGCACCTG

ACTGAGGACAAGGCCAACAAGAGACTGGCCTATGTGACTTACCAGGACATCCGTGCCGTT

GGCAACTTTAAGGAACAGACGGTGATTGCTGTCAAGGCCCCTCCACAGACAAGACTGGAA

GTGCCTGACAGGAACGAGGAGAACCTGCAGATCTATCTGAAGAGCACCCAGGGGCCCATC

GAAGTCTACCTGTGCCCAGAGGAGGTTCAGGACCCGGACAGTCCTGCCAAGGAGCGCCTC

CCCCCAGCCTCCACCCTTGGCCCCAGCCCTGACTCCACCCAGCCCAGCAGCAGCACTGAC

CCTGGGATGACAGACCCCGTGGCATCTTCAGCATGCGTGTTCCAACCCAGTTTTAAAGCC

CCGCCACCGGGGCTGCCCCCCCCCAAGTCACCCTCGCTTGTCCCCCTGGAGGCCACCGAC

AGCATGCTGGAGCTGCCACACCCACTTCTGCAGCAGACTGAGGACCAGTTCCTGTCCCCA

ACCCTGCCATGCAGCTCTCCCCTGATCAGCTTCTCCCCGCCCTTGGACCAGGACGACTAC

CTGTGGGGCCTGGATGGGGGTGAGGGCATCAGTGACCTCTTCGACTCCTATGACCTCGGG

GACCTGCTGATTAAT

E2F2-Bt

------------------------------------------------------------

---------------------------------------------------------ATG

CTT------CTCAAACTCTGCCTCTCTCTCCTCTCTCTCCCC------------------

------------------------------------------------------------

---------------AAGGCCAAAAGGAAGCTGGACCTGGAGGGGATCGGGAGGCCCACG

GTCCCTGAATTCCGGACTCCCAAGGGGAAGTGCATTAGAGTGGATGGCCTCCCCAGCCCC

AAAACCCCCAAGTCCCCCGGAGAAAAGACTCGCTATGACACATCGCTGGGGCTGCTCACC

AAGAAATTCATTTACCTCCTGAGTGAGTCTGAGGATGGGGTTCTGGATCTGAACTGGGCT

GCCGAAGTGCTGGACGTGCAGAAGCGGCGCATCTACGACATCACCAACGTGCTGGAGGGC

ATCCAGCTCATCCGCAAGAAGGCCAAGAACAACATCCAGTGGGTGGGCAGGGGACTGTTT

GAAGACCCCACGCGGCCTGGGAAGCAGCAGCAGCTGGGGCAGGAGCTGAAGGAGCTGATG

AACATGGAGCAGGCCCTGGACCAGCTCATCCACAGCTGCTCTCTGAACTTCAAGCACCTG

ACGGAGGACAAGGCCAACAAGAGACTGGCCTACGTGACGTACCAGGACATCCGTGCTGTG

GGCAACTTTAAGGAGCAGACGGTGATTGCGGTCAAGGCCCCTCCGCAGACTAGACTGGAG

GTGCCTGACAGGAGTGAGGAGAACCTGCAGATACATCTGAAGAGCACACAGGGGCCCATC

GAGGTTTACCTGTGCCCAGAAGAGGTGCAGGAGCCCCACAGTCCTGCCAAGGAGCCCCTC

CCCTCCACTTCCGCCCTCAGCCCCAGCCCTGACTCCACCCAGCTCAACAGCAACAGCGAC

CCTGGGATCACGGAACCCACGGCATCCTCA------------GAGCCAGCACTGACGTCC

CCG------CAGGTCCCGCCGCCACCCCCGCCGCCCCTTGTCCCCCTGGAGGCCACGGAG

AACATGCTGGAGCTGCCGCATCCGCTGCTGCAGCAGACGGAGGACCAGTTCCTGTCCCCA

ACGCTGCCCTGCAGCTCCCCTCTGATCAGCTTCTCCCCGCCCTTAGACCAGGATGACTAC

CTGTGGGGCCTGGACGGCGGGGAGGGCATCAGCGACCTCTTTGATACCTACGATCTCGGG

GACCTGCTGATTAAC

**3. The Codon sequences alignment For E2F3 proteins (Nuc format)**

5 1527

E2F3-hs

ATGAGAAAGGGAATCCAGCCC------------------------GCTCTGGAGCAGTAC

CTGGTGACCGCCGGGGGTGGGGAGGGGGCGGCTGTCGTCGCCGCCGCCGCTGCAGCCTCC

ATGGACAAAAGGGCACTGCTAGCCAGCCCCGGCTTCGCCGCCGCCGCCGCCGCTGCC---

------GCCGCCCCGGGCGCGTACATCCAGATCCTCACCACG------------------

---------AACACTTCCACCACCTCCTGTTCCTCCTCCCTCCAAAGCGGCGCCGTAGCC

GCCGGCCCCCTCCTCCCCAGTGCCCCCGGCGCGGAGCAGACCGCCGGCAGCCTCCTCTAC

ACCACGCCGCACGGACCCTCCAGCAGAGCCGGGCTGCTGCAGCAGCCA------------

---------------CCAGCGCTGGGACGCGGCGGCAGCGGCGGCGGCGGCGGCCCTCCG

---------------------------------------------GCAAAGCGAAGGCTG

GAGCTAGGAGAAAGCGGTCATCAGTACCTCTCAGATGGTTTAAAAACCCCCAAGGGCAAA

GGAAGAGCTGCACTACGAAGTCCAGATAGTCCAAAAACTCCAAAATCTCCCTCAGAAAAA

ACGCGGTATGATACGTCTCTTGGTCTGCTCACCAAGAAGTTCATTCAGCTCCTGAGCCAG

TCACCCGATGGGGTATTGGATTTGAACAAGGCAGCAGAAGTGCTAAAAGTGCAAAAGAGA

AGGATTTATGATATCACCAACGTTCTGGAAGGCATCCACCTCATTAAGAAGAAGTCTAAA

AACAACGTCCAATGGATGGGCTGCAGTCTGTCTGAGGATGGGGGCATGCTGGCCCAGTGT

CAAGGCCTGTCAAAAGAAGTGACCGAGCTCAGTCAGGAAGAGAAGAAATTAGATGAACTG

ATCCAAAGCTGCACCCTGGACCTCAAACTGTTAACCGAGGATTCAGAGAATCAAAGGTTA

GCTTATGTTACATATCAAGATATTCGAAAAATTAGTGGCCTTAAAGACCAAACTGTTATA

GTTGTGAAAGCCCCTCCAGAAACAAGACTTGAAGTGCCTGACTCAATAGAGAGCCTACAA

ATACATTTGGCAAGTACCCAAGGGCCCATTGAGGTTTACTTATGTCCAGAAGAGACTGAA

ACACACAGTCCAATGAAAACAAACAACCAAGACCACAATGGGAATATCCCTAAACCCGCT

TCCAAAGACTTGGCTTCAACCAACTCAGGACATAGCGATTGCTCAGTTTCTATGGGAAAC

CTTTCTCCTCTGGCCTCCCCAGCCAACCTCTTACAGCAGACTGAGGACCAAATTCCTTCC

AACCTAGAAGGACCGTTTGTGAACTTACTGCCTCCCCTGCTGCAAGAGGACTATCTCCTG

AGCCTCGGGGAGGAGGAAGGCATCAGCGATCTCTTCGATGCTTACGATTTGGAAAAGCTC

CCACTGGTGGAAGACTTCATGTGTAGT

E2F3-Mm

ATGAGAAAGGGGATCCAGCCC------------------------GCCCTGGAGCAGTAC

CTGGTGACCGCCGGGGGTGGGGAGGGGGCGGCTGTCGTCGCCGCCGCCGCTGCAGCCTCC

ATGGACAAAAGGGCACTGCTAGCCAGCCCCGGCTTCGCCGCCGCCGCCGCC---------

------------CCGGGCACGTACATCCAGATCCTCACTACG------------------

---------AACCCTTCCACCACGTCCTGTGCCACCTCCCTCCAAAGTGGCGCCCTGACC

GCCGGCCCCCTTCTCCCCAGTGTCCCCGGCACGGAGCCGGCCGCCAGCAGC---CTCTAC

ACCACGCCACAAGGACCCTCCAGCAGAGTCGGGCTGCTGCAGCAGCCA------------

---------------CCAGCACCAGGACGCGGCGGC---------GGCGGTGGCCCACCG

---------------------------------------------GCAAAGCGAAGGCTG

GAACTGGGCGAGAGTGGCCATCAGTACCTCTCAGATGGTCTAAAGACCCCCAAGGGCAAA

GGAAGAGCTGCACTACGGAGTCCCGATAGTCCAAAAACTCCAAAATCTCCCTCAGAAAAA

ACGCGGTATGATACGTCCCTCGGTCTGCTCACCAAGAAGTTCATTCAGCTCCTGAGCCAG

TCTCCTGATGGGGTCCTGGATCTGAACAAGGCAGCAGAGGTGCTCAAGGTGCAGAAGAGG

AGGATTTACGACATCACCAACGTGCTGGAAGGCATCCACCTCATTAAGAAGAAGTCTAAG

AACAACGTCCAGTGGATGGGCTGCAGTCTGTCTGAGGATGGGGGCATGCTGGCCCAGTGT

CAAGGCCTGTCCAAAGAAGTGACTGAGCTCAGTCAGGAAGAGAAGAAATTAGATGAGCTG

ATCCAAAGCTGTACCCTGGACCTCAAACTGTTAACCGAGGATTCAGAGAATCAAAGGTTA

GCTTATGTTACATATCAAGATATTCGAAAAATTAGTGGCCTTAAAGACCAAACTGTTATA

GTTGTGAAAGCCCCTCCAGAAACGAGACTTGAAGTGCCTGACTCAATAGAGAGCCTACAA

ATCCATTTGGCAAGTACCCAAGGGCCCATTGAGGTTTACTTGTGTCCAGAAGAGACGGAA

ACACACAGACCCATGAAAACAAATAACCAAGACCACAATGGGAATATCCCCAAGCCCACT

TCCAAAGACTTGGCTTCTAACAACTCAGGACATAGTGACTGCTCGGTTTCTACAGCAAAC

CTCTCTCCTCTGGCCTCCCCAGCCAACCTTTTACAGCAGACTGAGGACCAAATCCCGTCC

AACCTTGAAGGACCTTTTGTGAACTTACTGCCTCCCCTGCTCCAAGAGGACTACCTGCTG

AGCCTGGGGGAGGAAGAGGGCATCAGTGACCTCTTCGATGCCTATGATTTGGAAAAGCTG

CCTCTGGTGGAGGACTTCATGTGTAGT

E2F3-Rn

ATGAGAAAGGGGATCCAGCCC------------------------GCCCTGGAGCAGTAC

CTGGTGACCGCCGGGGGTGGGGAGGGGGCGGCTGTCGTCGCCGCCGCCGCTGCAGCCTCC

ATGGACAAAAGGGCACTGCTAGCCAGCTCCGGCTTTGCCGCCGCCGCCGCC---------

------------CCGGGCACGTACATCCAGATCCTCACCACG------------------

---------AACCCTTCCACCACGTCCTGTGCCACCTCCCTCCAAAGCGGCGCCCTGGCC

GCCGGCCCCCTTCTCCCCAGTGTCCCCGGCACGGAGCCGGCCGCCAGCAGCCTCCTCTAC

ACCACGCCACAAGGACCCTCCAGCAGAGCCGGGCTGCTACAGCAGCCA------------

---------------CCAGCAGCAGGACGCGGCGGC---------GGCGGTGGCCCACCG

---------------------------------------------GCAAAGCGAAGGCTG

GAGCTGGGCGAGAGTGGCCATCAGTACCTCTCAGATGGTCTAAAAACCCCCAAGGGCAAA

GGAAGAGCTGCACTACGGAGTCCTGATAGTCCAAAAACTCCAAAATCTCCCTCAGAAAAA

ACGCGGTATGATACGTCACTCGGTCTGCTCACCAAGAAGTTCATTCAGCTCCTGAGCCAG

TCACCTGATGGGGTCCTGGATCTGAACAAAGCAGCAGAGGTGCTCAAGGTGCAGAAGAGG

AGGATCTACGACATCACCAACGTGCTGGAAGGCATCCACCTCATTAAGAAGAAATCTAAG

AACAACGTCCAGTGGATCTTATGTTACATATCAAGA------------------------

------TATTCGAAAAAT------------------------------------------

------------------------------------------------------------

------------------------------------------------------------

------------------------------------------------------------

------------------------------------------------------------

------------------------------------------------------------

------------------------------------------------------------

------------------------------------------------------------

------------------------------------------------------------

------------------------------------------------------------

---------------------------

E2F3-CF

ATGCCCTCCCCAGTAGCTAGTGATGTCCCTGTGCCCCGACATGGCCACGTCCAGCAGCTG

ACACCAGCATCTGTCCATGTCCGTGACCTCGAAATGGTCCATTACAGTCCTGCTGCCTCC

AGCAGCCCGAACGTTGTTTCTGCCTCCGGGGGAAGAACCAGCCGTGTCAGCCGTGGTCAA

CCTGTCTCTGCTTCTACTCCATACATTCTGGAGAGGAAGGCAAGGCAAGGGTGCTGCTGC

CCTGAAGAAGACAAGACCGTTACTCGTACATCAGCAGAGAACAGGTCTGGTTGCAAGAGG

TACTCGCCTCATCAGGACCAGAAGAGGGTATTACAAGCCTTGATGATTGAGCAGAAAGAT

CTTAGGACAGCTGGTCAAAACAAGCGTGTCCTGCTTTGTGGGAGGGACAGAGCCTTCCAA

ACGATCTTTTGGTATGAGGCAGTGGTGAGAAAGCAGGAAGGTGCAGGGAACATGCTGCCC

ATAATTGAAGCTCCTGACTTCAGCGCTTGTGCTTTCACGTTGCAGGCAAAGCGAAGGCTG

GAGCTGGGAGAAAGCGGTCATCAGTACCTCTCAGATGGCTTAAAAACCCCCAAGGGCAAA

GGAAGAGCCACACTGCGAAGTCCAGATAGTCCAAAAACTCCAAAATCTCCCTCAGAAAAA

ACACGGTATGACACATCACTTGGTCTGCTCACCAAGAAGTTCATTCAGCTGCTGAGCCAG

TCCCCTGATGGGGTCTTGGATTTGAACAAGGCAGCGGAGGTGCTGAAAGTGCAAAAGAGA

AGAATCTACGACATCACCAACGTGCTGGAAGGCATCCACCTCATTAAGAAGAAGTCGAAA

AACAACGTGCAGTGGATGGGCTGCAGTCTGTCTGAGGATGGGGGCATGCTGGCCCAGTGT

CAAGGCCTCTCAAAGGAAGTGACCGAGCTCAGCCAGGAAGAGAAGAAATTAGATGAACTG

ATCCAAAGCTGCACCCTGGACCTCAAGCTGCTAACCGAGGATTCAGAGAATCAAAGGTTA

GCTTATGTTACATATCAAGATATTCGAAAAATTAGTGGCCTTAAAGACCAAACTGTTATA

GTTGTCAAAGCCCCTCCAGAAACAAGACTTGAAGTGCCTGACCCAATAGAGAGCCTACAA

ATACATTTGGCAAGTACCCAAGGGCCCATTGAGGTTTATTTGTGTCCAGAAGAGACAGAA

ACACACAGTCCAATGAAAACAACCAACCAAGACCACAATGGGAATATCCCGAAACCCCCT

TCCAAAGACTTGGCTTCAACCAACTCAGGACATAGCGATTGTTCAATTTCTATGGCAAAC

CTTTCTCCTTTGGCCTCCCCAGCCAACCTCTTACAGCAGACTGAGGACCAAATTCCTTCC

AACTTAGAAGGACCATTTGTGAACTTACTGCCTCCCCTGCTCCAAGAAGACTATCTCCTA

AGCCTTGGGGAGGAAGAAGGCATCAGTGATCTCTTTGATGCTTATGATTTGGAAAAGCTC

CCACTGGTGGAAGACTTTATGTGTAGT

E2F3-Bt

ATGAGAAAGGGGATCCAGCCC------------------------GCCCTGGAGCAGTAC

CTGGTGACCGCCGGGGGTGGGGAGGGGGCGGCTGTCGTCGCTGCGGCCGCTGCAGCCTCC

ATGGACAAAAGGGCACTGCTAGCCAGCCCCGGCTTCCCCGCCGCCGCCGCCGCC------

---------GCCCCGAGCGCGTACATCCAGATCCTCACCACG------------------

---------AACACTTCCACCACCTCCTGTTCCTCCTCCCTCCAAAGCGGCGCCGTCGCC

GCCGGCCCCCTCCTCCCCAGTGCCCCCGGCGTGGAGCAGACCGCTGGCAGCCTCATCTAC

ACCACGCCGCACGGACCCTCCGGCAGAGCCGGGCTGCTGCAGCAGCCA------------

---------------CCAGCGCTGGGACGCGGCGGCAGCGGCGGCGGCGGCGGCCCTCCG

---------------------------------------------GCAAAGCGCAGGCTG

GAGCTAGGAGAAAGCGGCCAGCAGTACCTCTCAGATGGTTTAAAAACCCCCAAGGGCAAA

GGAAGAGCTGCACTCCGGAGTCCAGACAGCCCAAAAACTCCAAAATCTCCCTCAGAAAAA

ACACGGTATGATACATCCCTTGGTCTGCTCACCAAGAAGTTCATTCAGCTCCTGAGCCAG

TCACCTGATGGGGTCTTGGATTTGAACAAGGCGGCAGAGGTGCTGAAGGTGCAAAAGAGA

AGGATTTACGACATCACCAACGTCCTGGAAGGCATCCACCTCATTAAGAAGAAGTCTAAA

AACAACGTGCAGTGGATGGGCTGCAGTCTGTCTGAGGACGGGGGCATGCTGGCCCAGTGT

CAAGGCCTGTCAAAAGAAGTGACCGAGCTCAGTCAGGAAGAGAAGAAATTAGATGAACTG

ATCCAAAGCTGCACCCTGGACCTCAAACTGTTAACCGAGGATTCAGAGAATCAAAGGTTA

GCTTATGTTACATATCAAGATATTCGAAAAATTAGTGGCCTTAAAGACCAAACTGTTATA

GTTGTGAAAGCCCCTCCAGAAACAAGACTTGAAGTGCCTGACCCAATAGAGAGCCTACAA

ATACATTTGGCAAGTACCCAAGGACCCATTGAGGTTTATTTGTGTCCAGAAGAGACTGAA

ACACACAGTCCAATGAAAACAAACAACCAAGACCACAATGGGAATATCCCTAAACCCACT

TCCAAAGACTTGGCTTCAACCAACTCAGGACATAGTGATTGCTCGATTTCTATGGCAAAC

CTTTCTCCTCTGGCCTCCCCGGCCAACCTTTTACAGCAGACTGAGGACCAAATTCCTTCC

AACCTAGAAGGACCATTTGTGAACTTACTGCCTCCCCTGCTCCAAGAAGACTATCTCCTA

AGCCTCGGGGAGGAAGAAGGCATCAGCGATCTCTTTGATGCTTACGATTTGGAAAAGCTC

CCGCTGGTGGAAGACTTCATGTGTAGT

**4. The Codon sequences alignments For E2F4 proteins (Nuc format)**

5 1242

E2F4-Hs

AUGGCGGAGGCCGGGCCACAGGCGCCGCCGCCCCCGGGCACUCCAAGCCGGCACGAAAAG

AGCCUGGGACUGCUCACCACCAAGUUCGUGUCCCUUCUGCAGGAGGCCAAGGACGGCGUG

CUUGACCUCAAGCUGGCAGCUGACACCCUAGCUGUACGCCAGAAGCGGCGGAUUUACGAC

AUUACCAAUGUUUUGGAAGGUAUCGGGCUAAUCGAGAAAAAGUCCAAGAACAGCAUCCAG

UGGAAGGGUGUGGGGCCUGGCUGCAAUACCCGGGAGAUUGCUGACAAACUGAUUGAGCUC

AAGGCAGAGAUCGAGGAGCUGCAGCAGCGGGAGCAAGAACUAGACCAGCACAAGGUGUGG

GUGCAGCAGAGCAUCCGGAACGUCACAGAGGACGUGCAGAACAGCUGUUUGGCCUACGUC

ACUCAUGAGGACAUCUGCAGAUGCUUUGCUGGAGAUACCCUCUUGGCCAUCCGGGCCCCA

UCAGGCACCAGCCUGGAGGUGCCCAUCCCAGAGGGUCUCAAUGGGCAGAAGAAGUACCAG

AUUCACCUGAAGAGUGUGAGUGGUCCCAUUGAGGUUCUGCUGGUGAACAAGGAGGCAUGG

AGCUCACCCCCUGUGGCUGUGCCUGUGCCACCACCUGAAGAUUUGCUCCAGAGCCCAUCU

GCUGUUUCUACACCUCCACCUCUGCCCAAGCCUGCCCUAGCCCAGUCCCAGGAAGCCUCA

CGUCCAAAUAGUCCUCAGCU---CACUCCCACUGCUGUCCCUGGCAGUGCAGAAGUCCAG

GGAAUGGCUGGCCCAGCAGCUGAGAUCACAGUGAGUGGCGGCCCUGGGACUGAUAGCAAG

GACAGUGGUGAGCUCAGUUCACUCCCACUGGGCCCAACAACACUGGACACCCGGCCACUG

CAGUCUUCUGCCCUGCUGGACAGCAGCAGCAGCAGCAGCAGCAGCAGCAGCAGCAGCAGC

AACAGUAACAGCAGCAGUUCGUCCGGACCCAACCCUUCUACCUCCUUUGAGCCCAUCAAG

GCAGACCCCACAGGUGUUUUGGAACUCCCCAAAGAGCUGUCAGAAAUCUUUGAUCCCACA

CGAGAGUGCAUGAGCUCGGAGCUGCUGGAGGAGUUGAUGUCCUCAGAAGUGUUUGCCCCU

CUGCUUCGUCUUUCUCCACCCCCGGGAGACCACGAUUAUAUCUACAACCUGGACGAGAGU

GAAGGUGUCUGUGACCUCUUUGAUGUGCCUGUUCUCAACCUC

E2F4-Cf

AUGGCGGAGGCCGGGCCGCAGGCGCCGCCGCCCCCGGGCACCCCAAGCCGGCACGAGAAG

AGCUUGGGACUUCUCACCACCAAGUUCGUGUCGCUUCUGCAGGAGGCCAAGGACGGCGUG

CUUGACCUCAAGCUGGCAGCUGACACCCUUGCUGUGCGCCAGAAGCGGCGGAUUUACGAC

AUUACCAACGUCCUGGAAGGUAUCGGGCUGAUCGAGAAAAAAUCCAAGAAUAGCAUCCAG

UGGAAGGGUGUGGGGCCUGGCUGCAACACCCGGGAGAUUGCGGACAAGCUGAUUGAGCUC

AAGGCUGAAAUCGAGGAGCUGCAGCAGCGGGAGCAAGAACUGGACAAGCACAAGGUGUGG

GUGCAGCAGAGCAUCCGGAAUGUCACAGAGGACGUGCAGAACAGCUGCUUGGCCUACGUG

ACUCAUGAGGACAUCUGCAGAUGCUUUGCUGGAGAUACCCUCUUGGCCAUCCGGGCCCCA

UCGGGAACCAGCCUCGAGGUGCCCAUCCCAGAGGGCCUCAAUGGACAGAAGAAAUACCAG

AUUCACCUGAAGAGUGUGAGUGGCCCUAUUGAAGUGCUGCUGGUAAACAAGGAGGCAUGG

AGCUCACCGCCUGUGGCAGUGCCUGUGCCACCACCAGAAGAUCUGCUCCAGAGCCCACCU

GCUGUCUCUACCCCUCCACCUCUGCCCAAGCCUACCCUGGCUCAGCCCCAUGACACCUCA

CGCCCAAGCAGUCCCCAGCUGACCACCCCCACCCCUGUCACUGGCAUCACUGAAGCCCAG

GGGGUGCCCGGUCCAGCAGCUGAGAUCGCAGUGAGUGGUGGCCCUGGAACGGACAGCAAG

GACGGUGGUGAGCUUGGCUCCCUCCCACCGGGCCUGGCAGCCCUGGACACUCGGCCGCUG

CAGUCCUCUGCCCUACUAGACAGCAGCAGCAGCAGCAGCAGCAGCAGCAGC---------

------AGCAACAGCAGCUCAUCCGGACCCAACCCUUCUACCUCCUUUGAGCCCAUCAAG

GCAGACCCCACAGGAGUUCUGGAACUCCCCAAAGAGCUGUCAGAAAUCUUUGAUCCCACA

AGAGAAUGCAUGAGCUCAGAGCUGCUGGAGGAGCUGAUGUCCUCAGAAGUGUUUGCCCCC

CUCCUCCGCCUAUCUCCACCCCCCGGAGACCACGAUUACAUCUACAACCUGGACGAGAGU

GAAGGUGUCUGUGACCUCUUCGAUGUGCCUGUUCUCAACCUC

E2F4-Bt

AUGGCGGAGGCCGGGCCACAGGCGCCGCCGCCCCCAGGCACCCCAAGCCGGCACGAGAAG

AGCUUGGGACUUCUCACUACCAAGUUCGUGUCGCUUCUGCAGGAAGCCAAGGACGGCGUG

CUUGACCUCAAGCUGGCAGCUGACACCCUAGCUGUGCGCCAGAAGCGGCGGAUAUAUGAC

AUUACUAACGUACUGGAAGGUAUCGGGUUGAUCGAGAAAAAAUCCAAGAAUAGCAUCCAG

UGGAAGGGUGUGGGGCCUGGCUGCAAUACCCGGGAGAUUGCGGACAAGCUGAUCGAGCUC

AAGGCAGAGAUCGAGGAGCUGCAGCAGCGGGAGCAAGAGCUAGACCAGCACAAGGUGUGG

GUGCAGCAGAGCAUCCGGAACGUCACAGAGGACGUGCACAACAGCUGCUUGGCCUACGUG

ACUCAUGAGGACAUCUGCAGAUGCUUUGCUGGAGACACCCUCCUUGCCAUCCGGGCCCCG

UCGGGCACCAGCCUGGAGGUGCCCAUCCCAGAGGGCCUCAAUGGGCAGAAGAAGUACCAG

AUUCACUUGAAGAGUGUAAGCGGCCCCAUUGAGGUGCUUCUGGUGAACAAGGAGGCAUGG

AGCUCACCGCCUGUGGCAGUGCCUGUGCCACCACCCGAAGACCUGCUCCAGAACCCGCCU

GCUGUCUCCACCCCUCCGCUUCUACCCAAGCCUUCCCUGGCCCAGCCCCAGGAUGCCUCA

CGCCCGAGCAGUCCCCAGGCGACCACCCCCAACCCUGUCCCCAGCAGCACUGAGGCCCAG

GGGGUGGCUGGUCCAGCAGCUGAGAUUCCAGUGAGUGGAGGCCAUGGAACCGAGAGCAAG

GACAGUGGUGAGCUCAGCUCCCUCCCGCUGGGCCUGGCAGCUCUGGACACCCGGCCGCUG

CAGUCCUCUGCCCUGUUGGACAGCAGCAGCAGCAGC------------------------

------AGCAACAGCAGUUCAUCUGGACCCAAUCCUUCUACCUCCUUUGAGCCCAUCAAG

GAAGACCCCACAGGUGUUUUGGAGCUCCCGAAAGAGCUGUCAGAAAUCUUUGAUCCCACU

CGAGAAUGCAUGAGCUCAGAGCUCUUGGAGGAACUGAUGUCCUCAGAAGUGUUUGCACCC

CUCCUCCGCCUUUCUCCACCCCCUGGAGACCACGAUUACAUCUACAACCUGGACGAGAGU

GAAGGUGUCUGUGACCUCUUUGAUGUGCCUGUUCUCAACCUC

E2F4-Mm

AUGGCGGAGGCCGGGCCACAGGCGCCGCCGCCCCCGGGGACUCCAAGCCGGCACGAGAAG

AGUCUGGGACUUCUCACCACCAAGUUCGUGUCGCUCUUGCAGGAAGCCAAGGACGGCGUG

CUUGACCUCAAGCUGGCAGCCGACACUCUUGCUGUGCGCCAGAAACGGCGGAUCUACGAC

AUCACCAACGUGCUGGAAGGCAUCGGUCUGAUCGAGAAGAAAUCCAAGAACAGCAUCCAG

UGGAAGGGCGUCGGGCCAGGUUGCAAUACCCGGGAGAUCGCUGACAAGCUGAUUGAGCUC

AAGGCAGAGAUCGAGGAGCUGCAGCAACGAGAGCAAGAACUGGACCAGCACAAGGUGUGG

GUGCAGCAGAGCAUCCGGAAUGUCACUGAGGACGUCCAGAACAGCUGCUUGGCCUACGUG

ACUCAUGAAGACAUCUGCAGAUGCUUUGCUGGAGAUACCCUCCUUGCCAUCCGGGCGCCA

UCGGGCACCAGUCUAGAGGUGCCCAUCCCAGAGGGUCUCAAUGGCCAGAAGAAGUACCAG

AUUCACUUAAAGAGCAUGAGUGGGCCUAUCGAGGUGCUGCUAGUGAACAAGGAGGCCUGG

AGUUCACCACCUGUGGCUGUGCCUGUCCCUCCCCCUGACGAUCUACUCCAGAGUCCACCU

GCUGUUUCUACACCUCCACCUCUGCCCAAGCCUGCCUUAGCUCAACCCCAAGAAUCCUCU

CCUCCAAGCAGCCCCCAGCUAACUACCCCCACUCCUGUCCUUGGCAGCACUCAAGUCUCA

GAGGUGGCAUGCCAGACAUCUGAGAUUGCAGUGAGUGGUAGCCCUGGAACUGAGAACAAG

GACAGUGGUGAAGUCAGCUCACUCCCACUGGGCCUGACAGCACUGGACACUCGGCCUCUG

CAGUCCUCUGCACUGCUGGAUAGUAGUAGCAGCAGCAGCAGCAGCAGCAGUAGC------

------AGCAGCAGCAGUUCAUCUGGACCCAACCCUUCUACCUCCUUUGAGCCCAUCAAA

GCAGACCCCACAGGCGUUCUGGAUCUCCCCAAAGAGCUGUCAGAAAUCUUCGACCCCACG

AGAGAGUGCAUGAGCUCCGAGCUGCUGGAAGAACUGAUGUCUUCAGAAGUGUUUGCCCCC

CUCCUCCGACUUUCUCCACCUCCCGGAGACCACGAUUACAUCUACAACCUGGACGAGAGU

GAAGGUGUCUGUGAUCUCUUUGAUGUUCCUGUUCUCAAACUC

E2F4-Rn

AUGGCGGAGGCCGGGCCACAGGCGCCGCCGCCCCCGGGUACUCCAAGCCGGCACGAGAAG

AGUCUGGGACUUCUCACCACCAAGUUCGUGUCGCUUCUGCAGGAAGCCAAGGACGGCGUG

CUUGACCUCAAGUUGGCAGCCGACACUCUUGCUGUACGCCAGAAACGGCGGAUUUACGAC

AUCACCAACGUGUUGGAAGGUAUUGGUCUGAUCGAGAAAAAAUCCAAGAACAGCAUCCAG

UGGAAGGGCGUCGGGCCAGGUUGCAAUACCCGGGAGAUUGCCGACAAACUGAUUGAGCUC

AAGGCAGAGAUCGAGGAGCUACAGCAGCGGGAGCAAGAACUUGACCAGCACAAGGUGUGG

GUGCAGCAGAGCAUCCGGAAUGUCGCAGAGGACGUCCAGAACAGCUGCUUGGCCUACGUG

ACUCAUGAAGACAUCUGCAGGUGCUUUGCUGGAGAUACCCUCCUUGCUAUCCGAGCCCCA

UCGGGCACCAGCCUGGAGGUGCCCAUCCCAGAGGGUCUCAAUGGUCAGAAGAAGUACCAG

AUUCACUUAAAGAGCAUGAGUGGUCCCAUUGAGGUCCUGCUAGUGAACAAGGAGGCCUGG

AGUUCACCACCUGUGGCUGUGCCCGUACCUCCCCCUGACGAUCUACUCCAGAGCCCACCU

GCUGUGUCUACACCUCCACCUCUGCCCAAGCCUGCCUUAGCUCAUCCCCAAGAAACCUCU

CGUCCAAGCAGCCCCCAGAUAACUACCCCCACUCCUGUCCUUGGCAGCACUGAAGUCUCA

GAUGUGGCAGGCCAGACAGCUGAGAUUGCAGUGAGUGGUAGCCCUGGAACUGAGAACAAG

GGCAGUGGUGAACUCAGUUCACUCCCACUGGGCCUGACAGCACUGGACACUCGACCUCUG

CAGUCCUCUGCACUACUGGAUAGUAGCAGCAGCAGCAGCAGCAGUAGCAGCAGCAGCAGC

------AGCAGCAGCAGUUCAUCUGGACCCAACCCUUCUACCUCCUUUGAGCCCAUCAAG

GCAGACCCCACAGGCGUUCUGGAUCUCCCCAAAGAGCUGUCAGAAAUCUUCGACCCCACA

AGAGAGUGCAUGAGCUCCGAGCUGCUGGAAGAAUUGAUGUCUUCAGAAGUGUUUGCCCCC

CUCCUCCGACUUUCUCCACCUCCCGGAGACCACGAUUACAUCUACAACCUGGACGAGAGU

GAAGGUGUCUGUGAUCUCUUUGAUGUUCCUGUUCUCAAACUC

**5. The Codon sequences alignment For E2F5 proteins (Nuc format)**

3 1038

E2F5-Hs

ATGGCGGCGGCAGAGCCCGCGAGCTCGGGCCAGCAGGCGCCGGCAGGGCAGGGGCAGGGC

CAGCGGCCGCCGCCGCAGCCTCCGCAGGCGCAAGCCCCGCAGCCGCCCCCGCCGCCGCAG

CTCGGGGGCGCCGGGGGCGGCAGCAGCAGGCACGAGAAGAGCCTGGGGCTGCTCACTACC

AAGTTCGTGTCGCTGCTGCAGGAGGCCAAGGACGGCGTTCTGGATCTCAAAGCGGCTGCT

GATACTTTGGCTGTGAGGCAAAAAAGGAGAATTTATGATATCACCAATGTCTTAGAGGGA

ATTGACTTGATTGAAAAAAAGTCAAAAAACAGTATCCAGTGGAAAGGTGTAGGTGCTGGC

TGTAATACTAAAGAAGTCATAGATAGATTAAGATATCTTAAAGCTGAAATTGAAGATCTA

GAACTGAAGGAAAGAGAACTTGATCAGCAGAAGTTGTGGCTACAGCAAAGCATCAAAAAT

GTGATGGACGATTCCATTAATAATAGATTTTCCTATGTAACTCATGAAGACATCTGTAAT

TGCTTTAATGGTGATACACTTTTGGCCATTCAGGCACCTTCTGGTACACAACTGGAGGTA

CCCATTCCAGAAATGGGTCAGAATGGACAAAAGAAATACCAGATCAATCTAAAGAGTCAT

TCAGGACCTATCCATGTGCTGCTTATAAATAAAGAGTCGAGTTCATCTAAGCCCGTGGTT

TTTCCTGTTCCCCCACCTGATGACCTCACACAGCCTTCCTCCCAGTCCTTGACTCCAGTG

ACTCCACAGAAATCCAGCATGGCAACTCAAAATCTGCCTGAGCAACATGTCTCTGAAAGA

AGCCAGGCTCTGCAGCAGACATCAGCTACAGATATATCTTCAGCAGGATCTATTAGTGGA

GATATCATTGATGAGTTAATGTCTTCTGACGTGTTTCCTCTCTTAAGGCTTTCTCCTACC

CCGGCAGATGACTACAACTTTAATTTAGATGATAACGAAGGAGTTTGTGATCTGTTTGAT

GTCCAGATACTAAATTAT

E2F5-Mm

ATGGCGGCGGCGGAGCCCACGAGCTCTGCTCAGCCCACGCCGCAGGCCCAGGCTCAG---

------CCGCCGCCGCATGGG------------GCGCCATCCTCGCAGCCGTCCGCGGCG

CTCGCG---------GGGGGCAGCAGCCGGCACGAGAAGAGCCTGGGCTTGCTTACCACC

AAATTCGTGTCGTTGCTGCAGGAGGCGCAGGACGGCGTCCTGGATCTCAAAGCGGCTGCA

GATACCTTGGCTGTGAGGCAAAAGCGAAGAATTTATGATATCACCAATGTCTTAGAGGGA

ATTGATCTAATTGAAAAAAAATCAAAGAACAGTATCCAGTGGAAGGGTGTAGGTGCTGGC

TGTAATACTAAAGAAGTTATCGATAGATTAAGGTGTCTTAAAGCTGAAATTGAAGATCTC

GAATTGAAGGAAAGAGAACTTGACCAGCAGAAGTTGTGGCTACAGCAAAGCATCAAAAAT

GTGATGGAAGACTCCATTAATAACAGATTTTCTTATGTAACTCACGAAGACATCTGCAAT

TGCTTTCATGGTGATACACTGTTGGCCATTCAGGCACCTTCTGGTACACAGCTGGAAGTA

CCTATTCCAGAAATGGGACAGAATGGACAAAAGAAATACCAGATAAATCTGAAGAGTCAC

TCAGGGCCTATCCATGTGCTACTTATAAATAAAGAGTCCAGTTCATCTAAGCCAGTGGTT

TTTCCTGTTCCCCCACCTGATGACCTCACACAGCCTTCCTCCCAGTCCTCAACTTCAGTG

ACTCCACAGAAATCCACCATGGCTGCTCAAAACCTGCCTGAGCAGCATGTTTCCGAAAGA

AGCCAGACTTTCCAGCAGACACCAGCTGCAGAAGTATCTTCA---GGATCTATTAGTGGA

GACATCATTGATGAACTGATGTCTTCTGATGTGTTTCCTCTTTTACGGCTTTCTCCTACC

CCAGCAGATGACTACAACTTTAATTTAGATGATAATGAAGGAGTTTGTGATCTGTTTGAT

GTTCAGATACTAAATTAT

E2F5-Cf

ATGGGTGTGAAGGCTTTTGTGCGTTTCAGAGCTATTGTTCCCAAG---------------

------------------------------------------------------------

------------------------------------------------CAACTGACCAGG

TCAAATTTGGGATTGGGCTCTCATGCTGTCAACTTAATAATTAATCTTTTTTTGGCTGCA

GATACTCTGGCTGTGAGACAAAAGAGAAGAATTTATGATATCACCAATGTCTTGGAGGGA

ATTGACCTGATTGAAAAAAAGTCAAAAAACAGTATACAATGGAAAGGTGTAGGTGCTGGC

TGTAATACTAAAGAAGTCATAGATAGATTAAGATATCTTAAAGCTGAAATTGAAGATCTA

GAACTGAAGGAAAGAGAACTTGATCAGCAGAAGTTGTGGCTACAGCAAAGCATCAAAAAT

GTGATGGACGACTCCATTAATAATAGATTTTCCTATGTAACTCATGAAGACATCTGTAAT

TGCTTTAATGGTGATACACTTTTGGCCATTCAAGCACCTTCTGGTACTCAGCTGGAGGTA

CCTATCCCAGAAATGGGTCAGAATGGACAAAAGAAATACCAGATCAATCTAAAGAGTCAT

TCAGGACCTATCCACGTGCTGCTTATAAATAAAGAGTCCAGTTCGTCTAAGCCTGTGGTT

TTTCCTGTCCCCCCACCTGATGACCTCACACAACCCTCCTCTCAGCCCTCCACTCCAGTG

ACTGCACAGAAATCCAACATAGCAACCCAGAATCTGCCTGAA---CATGTCTCCGAAAGA

AGCCAAAATCTTCAACAGACACCAGCCACAGACTTATCATCAGCAGGATCTATTAGTGGA

GATATCATTGATGAGTTAATGTCTTCTGATGTGTTTCCTCTCTTACGGCTTTCTCCTACC

CCGGCAGATGACTACAACTTTAATTTAGATGATAATGAAGGAGTTTGTGATCTGTTTGAT

GTCCAGATACTAAATTAT

**6. The Codon sequences alignment For RB1 proteins (Nuc format)**

5 2787

Rb1-Hs

ATGCCGCCCAAAACCCCCCGAAAAACGGCCGCCACCGCCGCCGCTGCCGCCGCGGAACCC

CCGGCACCGCCGCCGCCGCCCCCTCCTGAGGAGGACCCAGAGCAGGACAGCGGC---CCG

GAGGACCTGCCTCTCGTCAGGCTTGAGTTTGAAGAAACAGAAGAACCTGATTTTACTGCA

TTATGTCAGAAATTAAAGATACCAGATCATGTCAGAGAGAGAGCTTGGTTAACTTGGGAG

AAAGTTTCATCTGTGGATGGAGTATTGGGAGGTTATATTCAAAAGAAAAAGGAACTGTGG

GGAATCTGTATCTTTATTGCAGCAGTTGACCTAGATGAGATGTCGTTCACTTTTACTGAG

CTACAGAAAAACATAGAAATCAGTGTCCATAAATTCTTTAACTTACTAAAAGAAATTGAT

ACCAGTACCAAAGTTGATAATGCTATGTCAAGACTGTTGAAGAAGTATGATGTATTGTTT

GCACTCTTCAGCAAATTGGAAAGGACATGTGAACTTATATATTTGACACAACCCAGCAGT

TCGATATCTACTGAAATAAATTCTGCATTGGTGCTAAAAGTTTCTTGGATCACATTTTTA

TTAGCTAAAGGGGAAGTATTACAAATGGAAGATGATCTGGTGATTTCATTTCAGTTAATG

CTATGTGTCCTTGACTATTTTATTAAACTCTCACCTCCCATGTTGCTCAAAGAACCATAT

AAAACAGCTGTTATACCCATTAATGGTTCACCTCGAACACCCAGGCGAGGTCAGAACAGG

AGTGCACGGATAGCAAAACAACTAGAAAATGATACAAGAATTATTGAAGTTCTCTGTAAA

GAACATGAATGTAATATAGATGAGGTGAAAAATGTTTATTTCAAAAATTTTATACCTTTT

ATGAATTCTCTTGGACTTGTAACATCTAATGGACTTCCAGAGGTTGAAAATCTTTCTAAA

CGATACGAAGAAATTTATCTTAAAAATAAAGATCTAGATGCAAGATTATTTTTGGATCAT

GATAAAACTCTTCAGACTGATTCTATAGACAGTTTTGAAACACAGAGAACACCACGAAAA

AGTAACCTTGATGAAGAGGTGAATGTAATTCCTCCACACACTCCAGTTAGGACTGTTATG

AACACTATCCAACAATTAATGATGATTTTAAATTCAGCAAGTGATCAACCTTCAGAAAAT

CTGATTTCCTATTTTAACAACTGCACAGTGAATCCAAAAGAAAGTATACTGAAAAGAGTG

AAGGATATAGGATACATCTTTAAAGAGAAATTTGCTAAAGCTGTGGGACAGGGTTGTGTC

GAAATTGGATCACAGCGATACAAACTTGGAGTTCGCTTGTATTACCGAGTAATGGAATCC

ATGCTTAAATCAGAAGAAGAACGATTATCCATTCAAAATTTTAGCAAACTTCTGAATGAC

AACATTTTTCATATGTCTTTATTGGCGTGCGCTCTTGAGGTTGTAATGGCCACATATAGC

AGAAGTACATCTCAGAATCTTGATTCTGGAACAGATTTGTCTTTCCCATGGATTCTGAAT

GTGCTTAATTTAAAAGCCTTTGATTTTTACAAAGTGATCGAAAGTTTTATCAAAGCAGAA

GGCAACTTGACAAGAGAAATGATAAAACATTTAGAACGATGTGAACATCGAATCATGGAA

TCCCTTGCATGGCTCTCAGATTCACCTTTATTTGATCTTATTAAACAATCAAAGGACCGA

GAAGGACCAACTGATCACCTTGAATCTGCTTGTCCTCTTAATCTTCCTCTCCAGAATAAT

CACACTGCAGCAGATATGTATCTTTCTCCTGTAAGATCTCCAAAGAAAAAAGGTTCAACT

ACGCGTGTAAATTCTACTGCAAATGCAGAGACACAAGCAACCTCAGCCTTCCAGACCCAG

AAGCCATTGAAATCTACCTCTCTTTCACTGTTTTATAAAAAAGTGTATCGGCTAGCCTAT

CTCCGGCTAAATACACTTTGTGAACGCCTTCTGTCTGAGCACCCAGAATTAGAACATATC

ATCTGGACCCTTTTCCAGCACACCCTGCAGAATGAGTATGAACTCATGAGAGACAGGCAT

TTGGACCAAATTATGATGTGTTCCATGTATGGCATATGCAAAGTGAAGAATATAGACCTT

AAATTCAAAATCATTGTAACAGCATACAAGGATCTTCCTCATGCTGTTCAGGAGACATTC

AAACGTGTTTTGATCAAAGAAGAGGAGTATGATTCTATTATAGTATTCTATAACTCGGTC

TTCATGCAGAGACTGAAAACAAATATTTTGCAGTATGCTTCCACCAGGCCCCCTACCTTG

TCACCAATACCTCACATTCCTCGAAGCCCTTACAAGTTTCCTAGTTCACCCTTACGGATT

CCTGGAGGGAACATCTATATTTCACCCCTGAAGAGTCCATATAAAATTTCAGAAGGTCTG

CCAACACCAACAAAAATGACTCCAAGATCAAGAATCTTAGTATCAATTGGTGAATCATTC

GGGACTTCTGAGAAGTTCCAGAAAATAAATCAGATGGTATGTAACAGCGACCGTGTGCTC

AAAAGAAGTGCTGAAGGAAGCAACCCTCCTAAACCACTGAAAAAACTACGCTTTGATATT

GAAGGATCAGATGAAGCAGATGGAAGTAAACATCTCCCAGGAGAGTCCAAATTTCAGCAG

AAACTGGCAGAAATGACTTCTACTCGAACACGAATGCAAAAGCAGAAAATGAATGATAGC

ATGGATACCTCAAACAAGGAAGAGAAA

Rb1-Mm

ATGCCGCCCAAAGCCCCGCGCAGA------------------GCCGCGGCCGCCGAGCCC

CCGCCACCGCCGCCGCCGCCGCCTCGGGAGGACGACCCCGCGCAGGACAGCGGC---CCC

GAAGAGCTGCCCCTGGCCAGGCTTGAGTTTGAAGAAATTGAAGAACCCGAATTTATTGCA

TTATGTCAAAAGTTAAAGGTACCCGATCATGTCAGAGAAAGAGCTTGGCTAACTTGGGAG

AAAGTTTCATCCGTGGATGGAATCCTGGAAGGATATATTCAAAAGAAGAAGGAACTCTGG

GGCATCTGCATCTTTATCGCAGCAGTTGATCTAGATGAGATGCCATTCACTTTTACTGAG

CTACAGAAAAGCATAGAAACCAGTGTCTATAAATTCTTTGACTTATTAAAAGAAATCGAT

ACCAGTACCAAGGTTGATAATGCTATGTCAAGACTATTGAAGAAGTATAATGTGTTATGT

GCACTCTACAGCAAATTAGAACGGACGTGTGAACTTATATATTTGACACAACCCAGCAGT

GCGTTATCTACTGAAATAAATTCTATGTTGGTGCTAAAAATTTCTTGGATCACTTTTTTA

CTAGCTAAAGGAGAAGTATTACAGATGGAAGATGACCTGGTAATCTCATTTCAGCTAATG

TTGTGTGTAGTTGACTATTTTATTAAGTTCTCACCTCCTGCACTACTCAGAGAGCCATAC

AAAACAGCTGCAATCCCCATTAATGGTTCACCTCGAACACCCAGAAGAGGTCAGAACAGG

AGCGCTCGGATAGCAAAACAACTAGAAAATGATACGAGGATTATCGAGGTTCTCTGTAAA

GAACACGAGTGTAATATAGATGAGGTGAAAAATGTTTATTTCAAAAATTTTATCCCTTTT

ATAAATTCACTTGGAATTGTATCATCTAATGGACTTCCAGAGGTTGAAAGTCTTTCTAAA

CGCTATGAAGAAGTTTATCTTAAAAACAAAGATTTAGATGCAAGACTGTTTTTGGATCAT

GATAAAACACTTCAGACTGATCCTATAGACAGTTTTGAAACAGAGAGAACGCCACGAAAA

AACAACCCTGATGAAGAGGCAAACGTGGTTACTCCACACACTCCAGTTAGGACTGTTATG

AATACTATTCAACAATTAATGGTGATTTTAAATTCTGCAAGTGATCAGCCATCAGAAAAT

CTGATTTCCTACTTCAATAATTGCACAGTGAATCCAAAAGAAAATATCCTAAAGAGAGTA

AAGGATGTTGGGCACATCTTTAAAGAGAAGTTTGCTAACGCTGTGGGCCAGGGCTGTGTT

GACATCGGAGTACAGCGATATAAACTTGGAGTCCGATTGTATTACCGTGTGATGGAATCC

ATGCTTAAATCAGAAGAAGAACGTTTGTCCATTCAGAATTTTAGCAAACTCCTAAATGAC

AACATCTTTCATATGTCTTTACTGGCCTGTGCTCTTGAAGTTGTAATGGCTACGTATAGC

AGAAGTACATTGCAGCATCTTGATTCTGGAACAGATTTGTCCTTCCCGTGGATTCTGAAC

GTACTTAATTTAAAAGCCTTTGATTTTTACAAAGTGATTGAAAGTTTTATCAAAGTGGAA

GCCAACTTGACAAGAGAAATGATAAAACATTTAGAAAGATGTGAGCATCGAATCATGGAA

TCCCTTGCATGGCTTTCAGATTCACCTTTATTTGATCTCATTAAGCAGTCCAAGGATGGA

GAAGGACCT---GATAACCTTGAACCTGCTTGTCCTCTCAGCCTGCCTCTCCAGGGTAAC

CATACTGCAGCAGATATGTATCTTTCTCCTCTAAGATCTCCAAAGAAAAGAACTTCCACT

ACACGTGTAAATTCTGCTGCAAATACAGAGACACAAGCAGCCTCAGCCTTCCATACTCAG

AAGCCATTGAAATCTACCTCCCTTGCCCTGTTTTACAAAAAAGTGTACCGTCTAGCATAT

CTCCGACTAAATACACTCTGTGCACGCCTTCTGTCTGACCACCCAGAGCTAGAGCACATC

ATCTGGACTCTGTTTCAGCATACATTGCAAAATGAGTATGAGCTCATGAGAGACCGACAT

TTGGACCAGATTATGATGTGCTCTATGTATGGCATCTGCAAGGTGAAGAACATCGACCTC

AAGTTCAAAATCATCGTCACTGCCTACAAGGATCTTCCTCACGCTGCCCAGGAGACCTTT

AAACGTGTTTTGATCAGAGAAGAGGAGTTTGATTCCATTATAGTATTCTATAACTCCGTT

TTCATGCAGAGACTAAAAACAAATATTTTACAGTATGCCTCCACCAGGCCTCCTACCTTG

TCACCAATACCTCACATTCCTCGAAGCCCTTACAAGTTTTCTAGTTCACCCTTACGGATT

CCTGGAGGTAACATCTATATATCACCCCTAAAGAGTCCTTATAAAATTTCAGAAGGTCTG

CCAACACCCACAAAAATGACTCCGAGATCAAGAATCTTGGTCTCAATTGGTGAATCATTT

GGGACATCTGAAAAGTTCCAGAAAATAAACCAGATGGTGTGTAATAGTGACAGAGTGCTC

AAAAGAAGTGCTGAAGGCGGCAACCCCCCCAAACCACTGAAAAAGCTGCGCTTTGACATC

GAGGGAGCCGATGAAGCAGATGGGAGTAAACATCTCCCAGCGGAGTCCAAATTCCAACAG

AAACTGGCAGAAATGACTTCCACTCGAACACGAATGCAAAAGCAGAGAATGAATGAGAGC

AAGGATGTCTCAAACAAGGAGGAAAAG

Rb1-Cf

------------------------------------------------------------

------------------------------------ATGGAGTTAGACTTGGGTTTCCCA

CTTACTGGCCATGTCCTCAAGCTTGTGTTTGAAGAAACCGAAGAACCCGATTTTACTGCA

TTATGTCAGAAATTAAAGATACCAGATCATGTCAGAGAAAGAGCTTGGTTAACTTGGGAG

AAAGTTTCATCTGTGGATGGAGTATTGGAAGGTTATGTTCAAAGGAAAAAGGAGCTATGG

GGAATCTGTATCTTTATTGCAGCAGTTGACCTAGATGAGATGCCATTCACTTTTACCGAG

CTACAAAAAACCATAGAAATCAGTGTCTACAGATTCTTTGATTTACTGAAAGAAATTGAT

ACCAGTACCAAAGTTGATAATGCTATGTCAAGACTATTGAAGAAGTACGATGTATTGTGT

GCACTCTACAGCAAATTAGAAAGGACATGTGAACTTATATATTTGACACAACCCAGCAGT

TTGATATCTACTGAAATAAACTCTGTGTTGGTGCTAAAAGTTTCTTGGATCACATTTTTA

CTAGCTAAAGGGGAAGTGTTACAAATGGAGGATGATCTCGTCATCTCATTTCAGTTAATG

CTCTGTGTCCTTGACTATTTTATTAAACTCTCACCTCCTGCATTGCTTAAAGAACCATAC

AAAACAGCTGTAATACCTTTTAATGGTTCACCTCGAACACCAAGGCGAGGTCAGAACAGG

AGTGCACGGATAGCAAAACAACTAGAAAATGATACAAGAATTATTGAAGTTCTCTGTAAA

GAACATGAATGTAATATAGATGAGGTGAAAAATGTTTATTTCAAAAATTTCATACCTTTT

ATGAATTCTCTTGGAATTGTAGCTTCTAATGGACTTCCAGAGGTGGAAAGTCTCTCTAGA

CAATATGAAGAGATTTACCTTAAAAACAAAGATTTTGATGCAAGATTATTTCTGGATCAT

GATAAAACTCTTCAGATTGATCCTACAGACAGTTTTGAAATGCAGAGAACACCACAAAAA

AGTAACTCTGATGAAGAGGTAAATGTGATTCTTCCACAGACTCCAGTTAGGACCGTAATG

AATACTATCCAACAATTAATGATGATCTTAAATTCAGCAAGTGATCAACCATCAGAAAAT

CTGATTTCCTATTTTAATAACTGCACAGTGAATCCAAAGGAAAGTATCCTGAAAAGAGTG

AAGGATGTTGGATACATCTTTAAAGAGAAATTTGCTAAAGCTGTGGGACAGGGATGTATG

GAAATTGGATCACAGCGATACAAACTTGGAGTCCGATTGTATTATCGAGTAATGGAATCC

ATGCTTAAATCAGAAGAAGAACGATTATCCATTCAAAATTTTAGCAAACTCCTGAATGAC

GACATCTTTCATATGTCTTTGTTGGCATGTGCTCTTGAGGTTGTAATGGCTACATATAGC

AGAAGTACATCTCAGAATCTTGATACTGGAACAGATTTGTCCTTCCCATGGATTCTGAAT

GTACTTAATTTAAAAGCCTTTGATTTTTACAAAGTGATTGAAAGTTTTATCAAAGCAGAA

GCCAACTTGACCAGAGAAATGATAAAACATTTAGAACGATGTGAACATCGAATCATGGAA

TCACTTGCATGGCTCTCTGATTCACCTTTATTTGATCTGATTAAACAAGCAAAAGACCGA

GAAGGACCAGCTGATCACCTTGAATCTGCTTGTACTCTCAACCTTCCTCTCCAGAGTAAT

CACACTGCAGCAGATATGTATCTTTCTCCTGTAAGATCCCCAAAGAAAAAAGGATCAACT

ATACGTGTAAATTCTACTGTAAACACAGAGGCACAAGCAACCTCAGCCTTCCAGACTCAG

AAGCCATTGAAATCTACCTCCCTTTCACTTTTTTACAAAAAAGTGTACCGACTAGCTTAT

CTTCGACTAAATACGCTGTGTGCACGCCTCCTATCTGACCACCCAGAACTAGAACACATC

ATCTGGACCCTTTTTCAGCACACACTGCAAAATGAGTATGAACTCATGAGAGACAGGCAT

TTGGATCAGATTATGATGTGTTCCATGTATGGCATATGCAAAGTGAAGAATATAGACCTT

AAATTCAAAATCATTGTAACAGCATATAAGGATCTTCCTCATGCTGTTCAGGAGACATTC

AAACGTGTTTTGATCAGAGAAGAGGAGTATGATTCCATTATAGTATTCTATAATTCGGTC

TTCATGCAGAGACTGAAAACAAATATTTTACAGTATGCTTCTACCAGGCCCCCTACCTTG

TCACCAATACCTCACATTCCTCGAAGCCCTTACAAGTTTTCTAGTTCACCTTTGCGGATT

CCTGGAGGGAACATCTACATATCACCCCTGAAGAATCCATATAAAATTTCAGAAGGTGTG

CCAACGCCAACAAAAATGACTCCAAGATCAAGAATCTTAGTGTCAATTGGTGAATCATTT

GGGACTTCTGAGAAGTTCCAGAAAATAAATCAGATGGTGTGTAACAGCGACCGTGTGCTC

AAAAGAAGTGCTGAAGGCAGCAACCCTCCTAAACCATTGAAAAAACTACGCTTTGATATC

GAAGGATCAGATGAAGCAGATGGAAGTAAACATCTCCCAGGGGAGTCTAAATTTCAACAG

AAACTGGCAGAAATGACATCCACCCGAACGCGAATGCAGAAGCAAAAACTGAATGATAGC

ATGGATACATCAAACAGAGAAGAGAAA

Rb1-Bt

ATGCCGCCCAAAACCCCCCGGAGAGCAGCAGCAGCCGCCGCCGCCGCCGCCGCGGAACCC

CCGCCGCCGCCCCTGCCGCCGCCCCCTGAGGAGGACCCCGAGCAGGACAGCGGC---TCC

GAGGACCTGCCTCTGGCCAGGCTTGAGTTTGAAGAAACTGAAGAACCTGATTTTACTGCA

TTATGTCAGAAATTAAAGCTACCAGATCATGTCAGAGAAAGAGCTTGGTTAACTTGGGAA

AAAGTTTCATCTGTGGATGGAGTATTGGAAGGTTATGTTCAAAAGAAAAAGGAACTGTGG

GGAATCTGTATTTTTATCGCAGCAGTTGACTTAGATGAGATGCCGTTCACTTTCACTGAG

CTACAGAAAAACATTGAAACCAGTGTCTATAAATTCTTTGATTTACTAAAAGAAATCGAT

ACCAGTACCAAAGTTGATAATGCTATGTCAAGACTGTTGAAGAAGTATAATGTGTTGTGT

GCACTCTACAGCAAATTAGAAAGGACATGTGAGCTTATATATTTGACACAACCCAGCAGT

TCGATATCTACTGAAATAAATTCTATGTTGGTGCTAAAAGTTTCTTGGATCACATTTTTA

TTAGCTAAAGGACAAGTATTACAAATGGAAGATGATCTGGTGATCTCATTTCAATTAATG

CTGTGTGTCCTTGACTATTTTATCAAACTCTCACCTCCTGCATTGCTCAAAGACCCATAC

AAAACAGCTGTAATACCTATTAATGGTTCACCTCGAACACCAAGGCGAGGTCAGAACAGG

AGTGCACGGATAGCAAAACAACTAGAAAATGATACAAGAATTATTGAAGTTCTCTGTAAA

GAACATGAATGTAATATAGATGAGGTGAAAAATGTTTATTTCAAAAATTTTATACCTTTT

ATGAATTCTCTTGGAATTGTAGCTTCTAATGGACTTCCAGAGGTGGAAAATCTCTCTAAA

CAATATGAAGAAATTTACCTTAAAAACAAAGATCTAGATGCAAGATTATTTCTGGATCAT

GATAAAACTCTTCAGGCTGATCCTACAGACAGTTTTGAAATGCAAAGAACACCACGAAAA

AGTAACCCTGATGAAGAGGTGAATATGATTCTTCCACAGACTCCAGTTAGAACTGTTATG

AATACTATCCAACAATTAATGATGATCTTAAATTCAGCAAGTGATCAACCATCGGAAAAT

CTGATTTCCTATTTTAATAATTGTACAGTGAATCCAAAGGACAGTATCCTGAAAAGAGTG

AAGGATATTGGGGACGTCTTTAAAGAGAAATTTGCTAAAGCTGTGGGACAAGGATGTATG

GAAATTGGATCACAGCGATACAAACTTGGAGTCCGATTGTATTACCGAGTAATGGAATCC

ATGCTTAAATCAGAAGAAGAACGATTATCCATTCAAAATTTTAGCAAACTCCTGAATGAC

AACATCTTTCATATGTCTTTGTTGGCATGCGCTCTTGAGGTTGTAATGGCTACCTATAGC

AGAAGTATGTCTCAGAGCCTGGATACTGGAACAGATCTATCCTTCCCATGGATTCTGAAT

GTATTTAACTTAAAAGCTTTTGATTTTTACAAAGTGATTGAAAGTTTCATCAAAGCAGAA

GCCAACCTGACCAGAGAAATGATTAAACATTTAGAACGATGTGAACATCGAATCATGGAG

TCCCTTGCCTGGCTCTCAGATTCACCTTTATTTGATCTTATTAAACAAGCAAAGGACCGA

GAAGGACCAGTTGATCACTTTGAACCTGCCTGTACTCTCAACCTTCCTCTCCAGAATAAT

CACACTGCAGCAGATATGTATCTTTCTCCTGTAAGATCCCCAAAGAAAAAAGGGCCAACT

CCACGTGTAAATTCTACTCCAAATTCAGAGGCACAAGCAACCTCAGCCTTCCAGACTCAG

AAGCCATTGAAATCTACCTCCCTTTCACTCTTCTACAAAAAAGTGTATCGACTAGCATAT

CTTCGACTGAACACCCTGTGTGCACGCTTTCTGTCTGACCACCCAGAACTAGAGCATATC

ATCTGGACCCTTTTCCAGCACACACTGCAAAATGAGTATGAACTCATGAGAGACAGGCAT

TTGGACCAGATTATGATGTGTTCTATGTATGGCATATGCAAAGTGAAGAATATAGACCTT

AAATTCAAAATCATTGTCACAGCATACAAAGATCTTCCTCATGCTGTTCAGGAGACATTC

AAACGTGTTTTGATTAGAGAAGAGGAGTATGATTCCATTATCGTATTCTATAATTCGGTC

TTCATGCAGAGACTGAAAACAAATATTTTGCAGTACGCTTCCACCAGGCCCCCTACCTTG

TCACCGATTCCTCACATTCCTCGAAGTCCTTACAAGTTTTCTAGTTCACCTTTACGGATT

CCTGGGGGGAACATCTATATATCACCCCTAAATAATCCATATAAAATTTCAGAAGGTCTG

CCGACACCAACAAAAATGACTCCAAGATCAAGAATCTTAGTATCAATCGGTGAATCATTT

GGGACTTCAGAGAAGTTCCAGAAAATAAATCAAATGGTATGTAACAGCGACCGCGTGCTC

AAAAGAAGTGCTGAAGGAAGCAACCCTCCTAAACCACTGAAAAAACTGCGCTTTGACATC

GAAGGATCCGATGAAGCAGATGGAAGTAAACATCTCCCAGGGGAATCCAAATTTCAACAG

AAACTGGCAGAAATGACATCTACTCGAACACGAATGCAAAAGCAGAAAATGAATGAGAGC

GTGGATACCTCAAACAGGGAAGAAAAG

Rb1-Rn

ATGCCGCCCAAAGCCCCGCGCAGA------------------ACCGCGGCCGCCGAGCCC

CCGCCGCCGCCGCCGCCGCCTCCG---GAGGACGACCCCGCGCAGGACAGTGAC---CCC

GAAGAGCTGCCCCTGATCAGGCTTGAGTTTGAAAAAATTGAAGAACCTGAATTTATTGCA

TTATGTCAGAAGTTAAAGGTGCCCGATCATGTCAGAGAGAGAGCTTGGCTAACGTGGGAG

AAAGTTTCATCTGTGGATGGAATCCTGGAAGGATATATTCAGAAGAAGAAGGAACTCTGG

GGCATCTGCATCTTCATTGCAGCAGTTGACCTGGATGAGATGCCATTCACTTTTACTGAG

CTTCAGAAAAGCATAGAAACCAGTGTCTATAAATTCTTTGACTTATTAAAAGAAATTGAT

ACCAGTACCAAAGTTGATAATGCTGTGTCAAGGCTATTGAAGAAGTACAATGTGTTATGT

GCACTCTACAGCAAATTAGAACGGACATGTGGACTTATATATTTGACACAGCCCAGCAGT

GGGTTATCTACTGAAATAAATTCTATGTTGGTGCTAAAAGTTTCTTGGATCACCTTTTTA

CTAGCTAAAGGAGAAGTAGTACAGATGGAAGATGATCTGGTGATCTCATTTCAGTTAATG

TTGTGTGTACTTGACTATTTTATCAAGCTCTCGCCTCCTGCGCTGCTCAGAGAGCCGTAC

AAAACAGCTGCAACACCCATTAATGGTTCACCTCGGACACCCAGAAGAGGTCAGAACAGG

AGTGCTCGGATAGCAAAACAACTAGAAAGTGATACAAGAACTATTGAAGTTCTCTGTAAG

GAGCACGAGTGTAATGTAGATGAGGTGAAAAATGTTTATTTCAAAAATTTTATCCCTTTT

ATAAGTTCACTTGGAATTGTATCATCTAATGGACTTCCAGAGCTTGAAAGTCTTTCTAAA

CGCTACGAAGAAGTTTATCTTAAAAGCAAAGATTTAGATGCAAGACTATTTTTGGATCAT

GACAAAACACTTCAGACTGATACTATAGACAGTTTTGAAACAGAGAGAACTCCACGAAAA

AGCAACCCTGATGAAGAGGCAAACATGGTTACTCCACACACTCCAGTTAGGACTGTTATG

AATACTATTCAACAGTTAATGGTGATTTTAAATTCTGCAAGTGATCAGCCATCAGAAAAT

CTGATTTCCTACTTCAGTAATTGCACAGTGAATCCAAAAGAAAATATCCTAAAAAGAGTA

AAGGATGTTGGACACATCTTTAAAGAGAAGTTTGCTAGCGCTGTGGGGCAGGGATGTATT

GACATTGGAGCACAGCGATATAAACTTGGGGTTCGATTGTATTATCGTGTGATGGAATCG

ATGCTTAAATCAGAAGAAGAACGTTTGTCCATTCAGAATTTTAGCAAACTCCTAAATGAC

AACATCTTTCATATGTCTTTATTGGCCTGTGCTCTTGAAGTTGTAATGGCTACATATAGC

AGGAGTATGTTACAGAATCTTGATTCTGGAACAGATTTGTCCTTCCCGTGGATTCTGAAT

GTACTTAATTTAAAAGCCTTTGATTTTTACAAAGTGATTGAAAGTTTTATCAAAGTGGAA

GCCAACTTGACAAGAGAAATGATAAAACATTTAGAAAGATGTGAGCATCGAATCATGGAA

TCCCTTGCATGGCTTTCTGATTCACCTTTGTTTGATCTCATAAAGCAGTCCAAGGATGGG

GAAGGACCT---GATCACCTTGAGTCTGCTTGTTCTCTCAGCCTCCCTCTCCAGAGTAAC

CACACTGCAGCAGATATGTATCTTTCTCCTATAAGATCTCCAAAGAAAAGAACTTCTACT

ACACGTGTAAATTCTGCGGCAAATACAGAGACGCAAGCAGCCTCAGCCTTCCATACTCAG

AAGCCATTGAAATCTACCTCCCTTTCCCTGTTTTACAAAAAAGTGTACCGTCTAGCATAT

CTCCGACTAAATACACTGTGTGCGCGCCTTCTGTCTGATCACCCAGAGCTAGAACACATC

ATCTGGACTCTGTTTCAGCATACATTGGAAAATGAGTATGAGCTCATGAAAGACCGACAT

TTGGACCAGATTATGATGTGTTCTATGTATGGCATCTGCAAGGTGAAAAACATTGACCTT

AAATTCAAAATCATCGTAACTGCGTATAAGGATCTTCCTCACGCTGCCCAGGAGACCTTT

AAACGTGTTTTGATCAGAGAAGAGGAGTTTGATTCCATTATAGTGTTCTACAACTCAGTT

TTCATGCAGAGACTAAAAACAAATATTTTACAGTATGCCTCCACCAGGCCTCCTACCTTG

TCACCAATACCTCACATTCCTCGAAGCCCTTACAAGTTCTCTAGTTCACCCTTGCGGATT

CCTGGAGGTAACATCTATATATCACCCCTAAAGAGTCCTTATAAAATTTCAGAAGGTCTG

CCAACACCCACAAAAATGACTCCAAGATCAAGAATCTTGGTCTCAATCGGTGAATCATTT

GGGACATCCGAGAAGTTCCAGAAAATAAACCAGATGGTGTGTAACAGTGACAGAGTGCTC

AAAAGAAGTGCTGAAGGCGGCAATCCCCCCAAACCCCTGAAGAAGCTGCGCTTTGACATC

GAAGGATCCGATGAAGCAGACGGGAGTAAACATCTCCCAGCGGAGTCCAAATTCCAACAG

AAACTGGCAGAAATGACTTCTACTCGAACACGAATGCAAAAGCAGAAACTGAATGATAGC

ATGGAAATCTCAAACAAGGAGGAAAAG

**7.The Codon sequences alignment For RBL1 proteins (Nuc format)**

5 3204

Rbl1-Hs

ATGTTCGAGGACAAGCCCCACGCTGAGGGGGCGGCGGTGGTCGCCGCAGCCGGGGAGGCG

CTACAGGCCCTGTGCCAGGAGCTGAACCTGGACGAGGGGAGCGCGGCCGAAGCCCTGGAC

GACTTTACTGCCATCCGAGGCAACTACAGCCTAGAGGGAGAAGTTACACACTGGTTGGCA

TGTTCATTATATGTTGCATGCCGCAAAAGCATTATTCCCACGGTTGGAAAGGGTATCATG

GAAGGCAACTGTGTTTCACTTACCAGAATACTACGTTCAGCTAAATTAAGTTTAATACAA

TTTTTTAGTAAAATGAAGAAATGGATGGACATGTCAAATCTACCACAAGAATTTCGTGAA

CGTATAGAAAGGCTAGAGAGAAATTTTGAGGTGTCTACTGTAATATTCAAAAAATATGAG

CCAATTTTTTTAGATATATTTCAAAATCCATATGAAGAACCACCAAAGTTACCACGAAGC

CGGAAGCAGAGGAGGATTCCTTGCAGTGTTAAGGATCTGTTTAATTTCTGTTGGACACTT

TTTGTTTATACTAAGGGTAATTTTCGGATGATTGGGGATGACTTAGTAAACTCTTATCAT

TTACTTCTATGCTGCTTGGATCTGATTTTTGCCAATGCGATTATGTGCCCAAATAGACAA

GACTTGCTAAATCCATCATTTAAAGGTTTACCATCTGATTTTCATACTGCTGACTTTACG

GCTTCTGAAGAGCCACCCTGCATCATTGCTGTACTGTGTGAACTGCATGATGGACTTCTC

GTAGAAGCAAAAGGAATAAAGGAGCACTACTTTAAGCCATATATTTCAAAACTCTTTGAC

AGGAAGATATTAAAAGGAGAATGCCTCCTGGACCTTTCAAGTTTTACTGATAATAGCAAA

GCAGTGAATAAGGAGTATGAAGAGTATGTTCTAACTGTTGGTGATTTTGATGAGAGGATC

TTTTTGGGAGCAGACGCAGAAGAGGAAATTGGAACACCTCGAAAGTTCACTCGTGACACC

CCATTAGGGAAACTGACAGCACAGGCTAATGTGGAGTATAACCTTCAACAGCACTTTGAA

AAAAAAAGGTCATTTGCACCTTCTACCCCACTGACCGGACGGAGATATTTACGAGAAAAA

GAAGCAGTCATTACTCCTGTTGCATCAGCCACCCAAAGTGTGAGCCGGTTACAGAGTATT

GTGGCTGGTCTGAAAAATGCACCAAGTGACCAACTTATAAATATTTTTGAATCTTGTGTG

CGTAATCCTGTGGAAAACATTATGAAAATACTAAAAGGAATAGGAGAGACTTTCTGTCAA

CACTATACTCAATCAACAGATGAACAGCCAGGATCTCACATAGACTTTGCTGTAAACAGA

CTAAAGCTGGCAGAAATTTTGTATTATAAAATACTAGAGACTGTAATGGTTCAGGAAACA

CGAAGACTTCATGGAATGGACATGTCAGTTCTTTTAGAGCAAGATATATTTCATCGTTCC

TTGATGGCTTGTTGTTTGGAAATTGTGCTCTTTGCCTATAGCTCACCTCGTACTTTTCCT

TGGATTATTGAAGTTCTCAACTTGCAACCATTTTACTTTTATAAGGTTATTGAGGTGGTG

ATCCGCTCAGAAGAGGGGCTCTCAAGGGACATGGTGAAACACCTAAACAGCATTGAAGAA

CAGATTTTGGAGAGTTTAGCATGGAGTCACGATTCTGCACTGTGGGAGGCTCTCCAGGTT

TCTGCAAACAAAGTTCCTACCTGTGAAGAAGTTATATTCCCAAATAACTTTGAAACAGGA

AATGGAGGAAATGTGCAGGGACATCTTCCCCTGATGCCAATGTCTCCTCTAATGCACCCA

AGAGTCAAGGAAGTTCGAACTGACAGTGGGAGTCTTCGAAGAGATATGCAACCATTGTCT

CCAATTTCTGTCCATGAACGCTACAGTTCTCCTACCGCAGGGAGTGCTAAGAGAAGACTC

TTTGGAGAGGACCCCCCAAAGGAAATGCTTATGGACAAGATCATAACAGAAGGAACAAAA

TTGAAAATCGCTCCTTCTTCAAGCATTACTGCTGAAAATGTATCAATTTTACCTGGTCAA

ACTCTTCTAACAATGGCCACAGCCCCAGTAACAGGAACAACAGGACATAAAGTTACAATT

CCATTACATGGTGTCGCAAATGATGCTGGAGAGATCACACTGATACCTCTTTCCATGAAT

ACAAATCAGGAGTCCAAAGTCAAGAGTCCTGTATCACTTACTGCTCATTCATTAATTGGT

GCTTCTCCAAAACAGACCAATCTGACTAAAGCACAAGAGGTACATTCAACTGGAATAAAC

AGGCCAAAGAGAACTGGGTCCTTAGCACTATTTTACAGAAAGGTCTATCATTTGGCAAGT

GTACGCTTACGTGATCTATGTCTAAAACTGGATGTTTCAAATGAGTTACGAAGGAAGATA

TGGACGTGTTTTGAATTCACTTTAGTTCACTGTCCTGATCTAATGAAAGACAGGCATTTG

GATCAGCTCCTCCTTTGTGCCTTTTATATCATGGCAAAGGTAACAAAAGAAGAAAGAACT

TTTCAAGAAATTATGAAAAGTTATAGGAATCAGCCCCAAGCTAATAGTCACGTATATAGA

AGTGTTCTGCTGAAAAGTATTCCAAGAGAAGTTGTGGCATATAATAAAAATATAAATGAT

GACTTTGAAATGATAGATTGTGACTTAGAAGATGCTACAAAAACACCTGACTGTTCCAGT

GGACCAGTGAAAGAGGAAAGAGGTGATCTTATAAAATTTTACAATACAATATATGTAGGA

AGAGTGAAGTCATTTGCACTGAAATACGACTTGGCGAATCAGGACCATATGATGGATGCT

CCACCACTCTCTCCTTTTCCACATATTAAACAACAGCCAGGCTCACCACGCCGCATTTCC

CAGCAGCACTCCATTTATATTTCCCCGCACAAGAATGGGTCAGGCCTTACACCAAGAAGC

GCTCTGCTGTACAAGTTCAATGGCAGCCCTTCTAAGAGTTTGAAAGATATCAACAACATG

ATAAGGCAAGGTGAGCAGAGAACCAAGAAGCGAGTAATAGCCATCGATAGTGATGCAGAA

TCCCCTGCCAAACGCGTCTGTCAAGAAAATGATGACGTTTTACTGAAACGACTACAGGAT

GTTGTCAGTGAAAGAGCAAATCAT

Rbl1-Mm

ATGTTCGAGGACGAGCCCCACGCCGAGGGGGCGGCGGCGGTCGCCGCGGCCAGGGAGGCG

CTGCAGGCCCTGTGCCAGGAACTGAACCTGGACGAGGGAAGCGCGGCCGAAGCCCTGGAT

GACTTCACGGCCATCCGCGGCAACTACAGCCTAGAGGGAGAAGTTATACACTGGCTGGCA

TGCTCTTTATATGTTGCTTGCCGCAAGAGCATCATTCCTACTGTGGGAAAGGGCGTCATG

GAAGGAAACTGTGTCTCACTCACCAGAATACTGCGCTCAGCTAAGTTAAGCTTAATACAG

TTTTTTAGTAAAATGAAGAAATGGATGGACATGTCAAACCTACCACAAGAGTTTCGTGAG

CGGATAGAAAGGCTAGAAAGAAATTTTGAAGTATCTACTGTAATTTTTAAAAAATTTGAG

CCCATTTTTTTAGATATCTTTCAAAATCCATATGAAGAGCCACCAAAGTTGCCCCGGAGC

CGAAAACAGAGGAGGATCCCCTGCAGTGTTAAGGACCTCTTTAACTTCTGCTGGACACTC

TTTGTTTACACCAAGGGGAATTTCCGTATGATTGGTGATGATTTAGTAAACTCCTATCAT

TTACTTCTGTGCTGCTTGGACCTGATCTTTGCCAATGCTATAATGTGCCCAAATAGACGA

GACTTGTTAAATCCATCATTTAAAGGCTTACCATCTGATTTCCATGCTCCGGACTTCAAA

GCCGCAGAAGAGCCTCCGTGCATCATTGCTGTACTTTGTGATCTGCATGACGGACTTCTA

GTAGAAGCAAAAGGGATAAAGGAGCATTACTTTAAGCCATATATTTCAAAGCTCTTTGAT

AAGAAGATTTTAAAAGGTGAATGCCTCTTGGATCTTTCCAGCTTTACTGATAATAGCAAA

GCCGTGAACAAGGAATATGAAGAGTATGTCCTAACTGTTGGGGACTTCGATGAGAGGATC

TTTTTGGGAGCAGATGCAGAGGAGGAGATTGGAACACCTCGAAAGTTCACTGCTGACACC

CCATTTGGGAAACTGACATCACAGGCCAGTGTGGAATGCAACCTTCAACAACACTTTGAA

AAAAAACGGTCATTTGCACCTTCTACCCCACTTACTGGACGGCGGTATTTACAAGAAAAA

GAGGCAGTCACCACGCCTGTAGCTTCAGCCACTCAAAGTGTAAGCCGGTTACAGAGCATC

GTCGCTGGATTAAAAAGCGCACCAAGTGAGCAGCTTCTGAATATTTTTGAATCTTGTATG

CGGAATCCTATGGGAAACATTATAAAAATTGTGAAAGGAATAGGAGAGACTTTCTGCCAA

CACTATACCCAGTCCACAGATAAACAGCCAGGATCTCACATAGACTTTGCTGTAAACAGA

CTAAAACTGGCAGAAATTTTGTATTACAAAATACTGGAGACTATAATGGTCCAGGAAACA

CGACGGCTTCATGGAATGGACATGTCGGTTCTTTTAGAGCAAGATATATTTCATAAATCC

TTGATGGCTTGTTGTTTGGAAATTGTGCTCTTTGCATATAGCTCACCCCGTACTTTCCCG

TGGATTATTGAAGTTCTCGATTTGCAGCCATTTTATTTTTATAAGGTTATTGAGGTGGTG

ATCCGCTCAGAGGAGGGCCTTTCCAGAGACATGGTGAAACATCTGAACAGCATTGAAGAA

CAGATTTTGGAGAGTTTGGCTTGGACTAATAATTCTGCACTGTGGGAGGCTCTGCATGCT

TCTGCAAACAGAGTCCCTTCCTGTGAAGAAGTTATATTCCCTAATAACTTTGAAATAGGT

AATGGAGGAAATGTGCAAGGCCATCTCCCCATGATGCCAATGTCTCCAATAATACATCCA

AGAGTCAAGGAAGTTCGCACTGACAGTGGGAGCCTTCGACAGGATATGCAACCATTGTCT

CCGATTTCTGTCCATGAGCGCTACAGCTCCCCTGCTGCAGGAAGTGCTAAGAGGAGACTG

TTTGGAGATGACCCACCAAAGGACACACTGATGGATAAGATTATGGCAGAAGGAACAAAG

CTGAAAATTGCTCCT---TCGAGTGTCACTGCTGAAAGCTTATCAATTTCCCCTGGGCAA

GCTCTTCTGACAATGGCCACAACCACAGTCACAGGGACGACGGGACGGAAAGTTACAGTC

CCTTTGCATGGTATCGCCAATGATGCTGGAGAAATCACACTGGTTCCTATCTCCATGAAT

CCAACTCAGGAGTCCACAGCTGAGAGCCCTGTGTCACTGACTGCCCAGTCACTAATTGGT

ACTTCTCCGAAACAGACCCATCTGACTAAAGCACAAGATGCTCATCTGACCGGAGTAAGC

AAACCCAAGAGGACTGGGTCCTTAGCACTGTTCTATAGGAAGGTCTATCATTTGGCAAGT

GTGCGCCTACGTGACTTATGTCTAAAACTTGATGTTTCAAATGAATTACGAAGGAAGATC

TGGACATGTTTTGAATTCACTTTAGTTCACTGCCCTGATTTAATGAAAGATAGGCATTTG

GATCAGCTCCTTCTGTGTGCATTTTACATCATGGCAAAAGTAACAAAAGAAGAAAGAACT

TTTCAAGAAATAATGAAAAGTTATAGGAATCAGCCCCAAGCTAATAGTCACGTATACAGG

AGTGTTCTCTTGAAGAGTATTCCAGGAGGAGTCGTGGTGTAC------------AATGGT

GACTGTGAAATGACCGATGGTGACATAGAAGATGCCACAAAGACTCCCAACTGTTCCAGT

GAACCAGTGAAAGAGGAAAGAGGTGATCTTATCAAATTTTACAATACAGTATATGTAGGA

AGAGTGAAGTCATTTGCATTGAAGTATGATTTATCCAATCAGGACCATATAATGGATGCT

CCCCCGCTCTCTCCTTTCCCACACATTAAGCAGCAGCCGGGTTCCCCACGCCGCATCTCC

CAGCAGCATTCCCTCTATGTGTCTCCACACAAGAACGGAGCGGGCCTCACCCCCAGGAGC

GCACTGCTGTACAAGTTCAACGGCAGCCCTTCTAAGAGTTTGAAAGATATCAACAACATG

ATAAGGCAAGGCGAGCAGAAAACCAAGAAGCGCGTGATAGCCATCAGTGGAGATGCAGAC

TCACCTGCCAAACGTCTCTGCCAAGAGAACGATGATGTTTTACTTAAGCGACTACAAGAT

GTTGTCAGTGAGAGAGCAAATCAT

Rbl1-Cf

ATGTTCGAAGACGACCCGCACGCCGAGGGGGCGGCGGTAGTCGCCGCGGCCGGGGAGGCG

CTGCAGGCCCTGTGCCAGGAGCTGAACCTGGACGAGGGGAGCGCGGCCGAAGCCCTGGAC

GACTTCACCGCCATCCGGGGCAACTACAGTCTAGAGGGAGAAGTTATACACTGGTTGGCA

TGTTCATTATATGTTGCCTGCCGCAAAAGCATTATTCCCACTGTTGGGAAGGGTATCATG

GAAGGAAATTGTGTTTCACTTACCAGAATACTGCGTTCAGCTAAACTAAGCTTAATACAG

TTTTTTAGTAAAATGAAGAAGTGGATGGACATGTCAAACCTGCCGCAAGAATTTCGTGAA

CGTGTAGAAAGGCTAGAAAGAAATTTTGAGGTGTCTACTGTAATTTTCAAAAAATTTGAG

CCAATTTTTTTAGATATATTTCAAAATCCATATGAAGAGCAACCAAAGTTCCCACGAAGC

AGGAAACAGAGAAGGATTCCTTGCAGCGTTAAGGATCTCTTTAATTTCTGCTGGACGCTC

TTTGTTTATACTAAGGGTAATTTTCGTATGATTGGAGATGATTTAGTAAACTCTTACCAT

TTACTTCTGTGCTGCTTGGATCTGATTTTTGCCAATGCCATTATATGCCCAAATAGACGT

GACTTGCTGAATCCAACATTTAAAGGTTTACCATCTGATTTCCATACTGCTGACTTTAAG

GCTTCCGAAGAGCCTCCCTGCATCATTGCTGTACTGTGTGAACTGCATGATGGACTTCTA

GTAGAAGCAAAAGGAATAAAGGAGCACTATTTTAAGCCATATATTTCAAAACTCTTTGAT

AGAAAGGTTTTAAAAGGAGAATGCCTCCTGGACCTTTCTAGTTTTACTGATAATAGCAAA

GCAGTGAACAAGGAGTATGAAGAATATGTTTTAACTGTTGGTGACTTTGATGAGAGGATC

TTTTTGGGAGCAGATGCAGAAGAGGAAATTGGAACTCCTCGAAAGTTCACTGGTGAAACC

CCATTAGGGAAACTGACAGCACAAGCTAATGTGGACTGTAGTCTTCAACAGCACTTTGAA

AAAAAAACATCATTTGCACCTTCTACCCCACTGACAGGACGGCGATATTTACGAGAAAAA

GAAGCAGTCATTACTCCTGTTGCTTCAGCTACTCAAAGTGTGAGCCGATTACAGAGTATT

GTGGCTGGACTGAAAAATGCACCAAGTGAACAACTTATAAATATTTTTGAATCTTGTATT

CGTAATCCAATGGAAAACATCATGAAAATAGTGAAAGGAATAGGGGAGACTTTCTGCCAA

CACTATACTCAATCAACAGATGAACAGCCAGGATCACACATAGATTTTGCTATAAACAGA

CTAAAGCTTGCAGAAATTTTGTATTATAAAATATTAGAGACTGTAATGGTTCAGGAAACA

CGAAGACTTCATGGAATGGACATGTCAGTTCTTTTAGAGCAGGATATATTTCATCGTTCC

TTGATGGCTTGTTGTTTGGAAATTGTGCTCTTTGCCTATAGCTCACCTCGTACTTTCCCC

TGGATTATTGAAGTTCTCAATTTACGACCATTTTATTTTTATAAGGTTATTGAGGTGGTG

ATCCGCTCAGAGGAAGGGCTTTCCAGGGACATGGTGAAACACCTGAACAGCATTGAAGAA

CAAATTTTAGAGAGTTTAGCGTGGAGTCACGATTCTGCATTGTGGGAGGCTCTCCAGGCT

TCAGCAAACAAAGTTCCTACTTGTGAAGAAGTTATATTCCCAAATAACTTTGAAACAGGA

AATGGGGGAAATGTACAGGGACATCTTCCCATGATGCCGATGTCTCCTCTAATGCATCCA

AGGGTCAAAGAAGTTCGGACTGACAGTGGGAGTCTTCGAAGGGATATGCAACCATTGTCT

CCAATTTCTGTCCATGAACGCTACAGTTCTCCTACTGCAGGGAGTGCTAAGAGAAGACTT

TTTGGGGAAGACCCCCCAAAGGAAATGCTTGTGGACAGGATCATAACAGAAGGAACAAAA

TTGAAAATTGCCCCTTCTTCAAGCATTACTGCTGAAAATATATCAATTTCACCTGGGCAG

AGTCTTCTAACTATGGCCACAGCCATAGTCACAGGGACAACAGGACATAAAGTTACAATC

CCATTGCACGGTATTGCAAATGATGCTGGAGAAATCACACTGATACCAATTTCCATGAAC

ACAGCTCAGGAGTCCAAAGTCGAGAGTCCTATATCACTTACTGCTCAGTCATTAATTGGT

GCTTCTCCGAAACAGACTCATCTGACTAAAGCGCAAGAGGCACATCCAACTGGAATAAGC

AAACCAAAGAGAACTGGTTCTTTAGCACTGTTTTATAGAAAGGTCTATCATTTGGCAAGT

GTCCGCTTACGTGATTTATGTTTAAAACTGGATGTTTCAAATGAGTTACGAAGGAAGATA

TGGACATGTTTTGAATTTACTTTAGTTCACTGCCCTGATCTTATGAAAGACAGACATTTG

GATCAGCTGCTCCTTTGTGCCTTTTACATCATGGCAAAGGTAACAAAAGAAGAAAGAACT

TTTCAGGAAATAATGAAAAGTTACAGGAATCAGCCCCAGGCTAATAGTCATGTCTATAGA

AGTGTACTATTGAAAAGTATTCCAAGAGAAGTTGTGGCATACAATAAAAACATAAATGGT

GATTTTGAAATGACAGATTGTGACTTGGAAGATGCTACAAAAACACCTGACTGTTCCAGT

GGACCAGTGAAAGAGGAAAGAGGTGATCTTATCAAATTTTACAATACAATATATGTAGCA

AGAGTGAAGTCATTTGCACTGAAATACGATTTGTCAAATCAGGACCATGTGATGGAAGCT

CCACCTCTCTCTCCTTTTCCACATATTAAGCAACAACCAGGCTCACCACGCCGCATTTCC

CAGCAACACTCCATTTATGTTTCCCCACATAAGAATGGGTCAGGCCTTACACCAAGGAGT

GCTCTGCTATATAAGTTCAATGGCAGCCCTTCTAAGAGTTTGAAAGATATCAACAATATG

ATACGGCAGGGTGAACAAAGAACCAAGAAGCGAGCAATAACCATCGATGGTGATGCGGAA

TCACCTGCCAAACGCCTCTGTCAAGAAAATGATGATGTTTTGCTTAAACGACTACAGGAT

GTTGTCAGTGAGAGAGCAAATCAT

Rbl1-Bt

ATGGACGAGGACGATCCCCACGCCGAGGGGGCGGCAGTGGTCGCCGCGGCCGGGGAAGCG

CTGCAGGCCCTGTGCCAGGAGCTGAACCTGGACGAGGGGAGCGCGGCCGAAGCCCTGGAC

GACTTCACTGCCATCCGGGGCAACTACAGCCTAGAGGGAGAGGTTATACACTGGTTGGCG

TGTTCGTTGTATGTTGCTTGCCGCAAAAGCATCATTCCCACAGTTGGGAAGGGTATCATG

GAAGGAAATTGCGTTTCACTTACCAGAATACTACGTTCAGCTAAATTAAGTTTAATACAG

TTTTTTAGTAAAATGAAGAAATGGATGGACATGTCAAACCTGCCACAAGAATTTCGTGAA

CGTATAGAAAGGCTAGAAAGAAATTTTGAGGTGTCTACTGTAATTTTCAAAAAATTTGAG

CCAATTTTTTTAGATATATTTCAAAATCCATATGAAGAGCCACCAAAGTTACCACGAAGC

AGGAAGCAGAGGAGGATTCCTTGCAGTGTTAAAGAGCTCTTTAATTTCTGCTGGACACTG

TTTGTTTATACTAAGGGTAATTTTCGTATGATTGGAGATGATTTAGTAAACTCTTATCAT

TTACTTCTGTGCTGCTTGGATCTGATTTTTGCCAATGCCATTATGTGCCCAAATAGACAA

GAGTTGCTAAATCCATCATTTAAAGGTTTACCATCTAATTTCCAAACTGCTGACTTTCGG

GCTTCTGAAGAACCTCCCTGCATCATTCCTGTACTGTGTGAACTGCATGACGGACTTCTG

GTAGAAGCCAAAGGAATAAAGGAGCATTACTTTAAGCCATATATTTCAAAACTCTTTGAT

AGGAAGATTTTAAAGGGCGAATGCCTTCTGGACCTTTGCAGTTTTACTGATAATAGCAAA

GCAGTGAACAAGGAGTACGAAGAATATGTTTTAACTGTTGGTGACTTTGATGAGAGGATC

TTTTTGGGAGCAGATGCAGAAGAGGAAATTGGAACCCCTCGGAAGTTCACTGGTGACGGC

CCATTAGGGAAACTGACAGCACAGGCTAATGTGGAGTGTAACCTTCAACATCATTTTGAA

AAAAAAACATCATTTGCACCTTCTACCCCACTGACAGGACGGCGATATTTAAGAGAAAAA

GAAGCAGTCATTACTCCTGTTGCTTCAGCTACCCAAAGTGTGAGCCGGCTACAGAGTATT

GTAGCTGGCTTGAAAAATGCACCAAGTGAACAACTTATAAATATTTTTGAGTCTTGTATG

CGTAATCCTATGGAAAATATTATGAAAATAGTGAAAGGAATAGGAGAGACTTTCTGCCAA

CACTATACTCAATCAACAGATGAACAGCCAGGATCTCACATAGACTTTGCTGTAAACAGA

CTAAAGCTTGCAGAAATTTTGTATTATAAAATATTAGAGACTGTAATGGTGCAGGAAACA

CGAAGACTTCATGGAATGGACATGTCAGTTCTTTTAGAGCAGGATATATTTCATCACTCC

TTGATGGCCTGTTGTTTGGAAATAGTGCTCTTTGCCTACAGCTCACCTCGTACTTTTCCC

TGGATTATTGAGGTTCTTAATCTGCGACCGTTTTATTTTTATAAGGTTATTGAGGTGGTA

ATCCGCTCAGAGGAGGGACTTTCCAGAGACATGGTAAAACACCTGAACAGCATTGAAGAA

CAGATTTTGGAGAGTTTAGCATGGAGTCATGATTCTGCGCTGTGGGAGGCTCTCCAGGCT

TCTGAAAACAGAGTTCCTACCTGTGAAGAAGTTATATTCCCAAACAATTTTGAAACAGGA

AGTGGAGGAAATGTACAGGGACATCTTCCCATGATGCCAATGTCTCCTCTGATGCATCCA

AGGGTCAAAGAAGTTCGGACTGACAGTGGAAGTCTTCGAAAGGATATGCAACCATTGTCT

CCGATTTCTGTCCATGAACGCTACAGTTCTCCTACTGCTGGGAGTGCTAAGAGAAGACTT

TTTGGGGAAGACCCCCCAAAGGAAATACTTATGGACAGGATCATTACAGAAGGAACAAAA

TTGAAAATTGCTCCTTCTTCAAGCATCACTGCTGAAAATATATCAATTTCACCCGGGCAT

AGTCTTCTGACAATGGCCACAGCCATAGTAGCAGGGACAACAGGACATAAAGTTACAATC

CCATTGCATGGTATTGCAAATGATGCTGGAGAGATCACACTGATACCAATTTCCATGAAT

ACAACTCAGGAGTCCAAAGTTGAGAGTCCTGTATCACTTACAGCTCAGTCATTAATTGGT

GCTTCTCCAAAGCAGACCCATTTGACCAAAGCACAAGAGGTACATCCAATTGGAATAAGC

AAACCAAAGAGAACTGGGTCCTTAGCACTGTTTTATAGAAAGGTCTATCATTTGGCAAGT

GTACGCTTACGTGATTTATGTTTAAAACTGGATGTTTCAAATGAGTTAAGAAGGAAGATA

TGGACATGTTTTGAATTCACTTTAGTTCACTGTCCTGATCTGATGAAAGACAGACATTTG

GATCAGCTCCTCCTTTGTGCCTTTTATATCATGGCAAAGGTAACAAAAGAAGAAAGAACT

TTTCAAGAAATAATGAAAAGTTATAGGAATCAGCCCCAAGCTAATAGTCACGTCTATAGA

AGTGTACTATTGAAAAGTATTCCAAGAGAAGTTGTGGCATACAGTAAAAACCTGAACGGT

GATTTTGAAATGACAGATTGTGACTTGGAAGATGCTACAAAAACACCGGACTGCTCCAGT

GGACCAGTGAAAGAGGAGAGAGGTGATCTTATCAAATTTTACAATACAATATATGTAGGA

AGAGTGAAGTCATTCGCATTGAAATACGACTTGTCAAATCAGGACCATGTGATGGAAGCT

CCGCCACTCTCCCCTTTTCCACATATTAAGCAACAGCCAGGCTCCCCACGCCGCATTTCC

CAGCAGCACTCCATTTATGTGTCCCCACACAAGAATGGGTCGGGCCTTACACCAAGGAGT

GCTCTGCTATATAAATTCAACGGCAGCCCTTCTAAGAGTTTGAAAGATATCAACAACATG

ATAAGGCAAGGTGAGCAGAGAACCAAGAAGCGAGCAATAACCATCGATGGTGATGCAGAA

TCACCTGCCAAACGCCTCTGTCAAGAAAATGATGATGTTTTACTTAAACGACTACAGGAT

GTTGTCAGTGAAAGAGCAAACCAC

Rbl1-Rn

ATGTTCGAGGACGAGCCCCACGCCGAGGGGGCGGCAGCCGTCGCCGCGGCCAGGGAGGCG

CTGCAGGCCCTGTGCCAGGAGCTGAACCTGGACGAGGGGAGCGCCGCCGAAGCCCTGGAC

GACTTCACCGCCATCCGCGGCAACTACAGCCTAGAGGGGGAAGTTATACACTGGCTGGCA

TGCTCTTTGTACGTCGCTTGCCGCAAGAGCATCATTCCTACCGTGGGAAAGGGCGTCATG

GAAGGAAACTGTGTTTCGCTGACCAGAATACTACGCTCAGCTAAGTTAAGCTTAATTCAG

TTTTTTAGTAAAATGAAGAAGTGGATGGACATGTCAAACCTACCACAAGAATTCCGGGAG

CGTATAGAAAGGCTAGAAAGAAATTTTGAAGTATCTACAGTAATTTTTAAAAAATTTGAG

CCCATTTTTTTAGATATCTTTCAAAATCCATATGAAGAGCTACCAAAGTTGCCACGAAGC

AGAAAGCAGAGGAGGATTCCTTGCAGTGTTAAGGATCTCTTTAATTTCTGCTGGACGCTC

TTCGTTTACACTAAGGGTAATTTTCGTATGATTGGTGATGATTTAGTAAACTCATATCAT

TTACTTCTGTGCTGCTTGGACCTGATTTTTGCCAATGCTATAATGTGTCCAAATAGACGA

GACTTGTTAAATCCATCATTTAAAGGTTTACCATCGGATTTCCATGCTGTGAACTTCAAA

GCTGCAGAAGAACCACCCTGTATCATTGCTGTACTTTGCGATCTGCACGATGGACTTTTA

GTAGAAGCAAAAGGAATAAAGGAGCACTACTTCAAACCATATATTTCAAAACTCTTTGAC

AAGAAGATTTTAAAAGGTGAATGTCTCTTGGATCTTTCCAGTTTTACTGATAATAGCAAA

GCAGTGAACAAGGAGTATGAAGAGTATGTTCTAACTGTTGGGGACTTTGACGAGAGGATC

TTTTTGGGAGCAGATGCAGAGGAGGAGATCGGAACACCTCGAAAGTTCGCTGCTGACACC

CAATTTGGGAAACTGACATCACAGGCCAGTGTGGACTGCAACCTTCAACAACACTTTGAA

AAAAAACGGTCATTTGCACCTTCTACCCCACTTACTGGACGGCGCTATTTACAAGAAAAA

GAGGCAGTTACCACACCTGTTGCTTCAGCTACTCAGAGTGTAAGCCGATTACAGAGCATT

GTAGCCGGATTGAAAAGTGCCCCGAGCGAGCAGCTTCTGACTATTTTTGAATCTTGTATG

CGGAATCCGATGGGAAACATTGTAAAAATAGTGAAAGGAATAGGAGAGACTTTCTGCCAG

CACTATACCCAGTCCACAGATAAACAGCCAGGATCTCACATAGACTTTGCTGTAAACAGA

CTGAAACTGGCAGAAATTTTGTATTATAAAATACTAGAGACTATAATGGTCCAGGAAACA

CGACGACTTCATGGAATGGACATGTCAGTTCTTTTAGAACAAGACATATTTCACAGGTCC

TTGCTGGCTTGTTGTTTGGAAATTGTGCTCTTTGCCTATAGCTCACCCCGTACTTTCCCT

TGGATCATTGACGTTCTCGGTTTGCAGCCGTTTTACTTCTATAAGGTTATTGAGGTGGTG

ATCCGCTCAGAGGAGGGGCTCTCCAGAGACATGGTGAAACACCTAAACAGCATTGAAGAG

CAGATTTTGGAGAGTTTAGCTTGGACTAATAATTCTGCACTGTGGGAAGCTCTCCGTGCT

TCTGCAAATAAAGTTCCTTCCTGTGAAGAAGTTATATTCCCAAATAACTTTGAAATAGGA

AATGGAGGAAGTGTGCAAGGCCATCTTCCCATGATGCCAATGTCTCCAATAATACATCCA

AGAGTCAAGGAAGTTCGCACTGACAGTGGGAGCCTTCGAAAAGATATGCAGCCACTGTCT

CCCATCTCCGTCCATGAGCGCTACAGCTCCCCTGCCGCAGGAAGTGCTAAGAGGAGACTC

TTTGGTGATGACCCACCAAAGGAGACATTGATGGAAAAGATTATGGCAGAAGGAACACAG

CTGAAAATTGCTCCT---TCAAGTGTGACTGCTGAAAGCTTGTCAATTTCCCCTGGGCAA

GCTCTTCTCACAATGGCCACGACCACAGTCACAGGGACGACGGGACGGAAGGTTACCGTT

CCTTTGCATGGTATTGCCAATGATGCTGGAGAAATCACACTGGTTCCTATTTCCATGAAT

ACAACTCAGGACTCCACAGCTGAGAGCCTTGTATCACTAACTGCACAGTCATTAATTGGT

GCTTCTCCAAAACAGACCCATCTGACTAAAACGCAAGACGCTCCTCTGACCGGAATAAGC

AAACCAAAGAGAACTGGGTCCTTAGCACTGTTTTATAGAAAGGTCTATCATTTGGCAAGT

GTACGCTTACGTGATTTATGTTTAAAACTTGATGTTTCGAATGAGTTACGAAGGAAGATC

TGGACATGTTTTGAATTCACTTTAGTTCACTGCCCTGATTTAATGAAAGATAGGCATTTG

GATCAGCTCCTTCTGTGTGCCTTTTACATCATGGCCAAAGTAACAAAAGAAGAAAGAACT

TTTCAAGAAATAATGAAAAGTTACAGAAATCAGCCACAAGCTAATAGTCACGTATACAGG

AGTGTTCTCTTGAAAAGTATTCCAGGAGAAGTTGTGGCATAC------------AATGGT

GACTATGAGATGACTGATGGTGACATAGAAGATGCCACAAAAACTCCCAACTGTTCCAGT

GAACCAGTGAAAGAGGAAAGAGGTGATCTTATCAAATTCTACAATGCGATATATGTAGGA

AGAGTGAAGTCATTTGCATTGAAGTATGATTTGTCCAATCAGGACCATATAATGGATGCT

CCACCGCTCTCTCCTTTCCCACATATTAAGCAGCAGCCGGGCTCCCCACGCCGCATTTCT

CAGCAGCATTCCATTTATGTCTCTCCGCACAAGAACGCATCAGGCCTCACCCCCAGGAGC

GCACTACTCTACAAGTTCAACGGCAGCCCCTCTAAGAGTAGGAAGGTTCTGAGAAACATG

GTTAACAACGGAGAGAAAAGAGCCAAGAAGCGTGTGATCGCCATCAGCGGAGATGCAGAG

TCACCTGCCAAACGTCTCTGCCAGGAGAACGATGATGTTTTACTTAAACGACTGCAGGAT

GTTGTCAGTGAAAGAGCGAATCAT

**7.The Codon sequences alignment For RBL1 proteins (Nuc format)**

5 3423

Rbl2-Hs

ATGCCGTCGGGAGGTGACCAGTCGCCACCGCCCCCGCCTCCCCCTCCGGCGGCGGCAGCC

TCGGATGAGGAGGAGGAGGACGACGGCGAGGCGGAAGACGCCGCGCCGCCTGCCGAGTCG

CCCACCCCTCAGATCCAGCAGCGGTTCGACGAGCTGTGCAGCCGCCTCAACATGGACGAG

GCGGCGCGGGCCGAGGCCTGGGACAGCTACCGCAGCATGAGCGAAAGCTACACGCTGGAG

GGAAATGATCTTCATTGGTTAGCATGTGCCTTATATGTGGCTTGCAGAAAATCTGTTCCA

ACTGTAAGCAAAGGGACAGTGGAAGGAAACTATGTATCTTTAACTAGAATCCTGAAATGT

TCAGAGCAGAGCTTAATCGAATTTTTTAATAAGATGAAGAAGTGGGAAGACATGGCAAAT

CTACCCCCACATTTCAGAGAACGTACTGAGAGATTAGAAAGAAACTTCACTGTTTCTGCT

GTAATTTTTAAGAAATATGAACCCATTTTTCAGGACATCTTTAAATACCCTCAAGAGGAG

CAACCTCGTCAGCAGCGAGGAAGGAAACAGCGGCGACAGCCCTGTACTGTGTCTGAAATT

TTCCATTTTTGTTGGGTGCTTTTTATATATGCAAAAGGTAATTTCCCCATGATTAGTGAT

GATTTGGTCAATTCTTATCACCTGCTGCTGTGTGCTTTGGACTTAGTTTATGGAAATGCA

CTTCAGTGTTCTAATCGTAAAGAACTTGTGAACCCTAATTTTAAAGGCTTATCTGAAGAT

TTTCATGCTAAAGATTCTAAACCTTCCTCTGACCCCCCTTGTATCATTGAGAAACTGTGT

TCCTTACATGATGGCCTAGTTTTGGAAGCAAAGGGGATAAAGGAACATTTCTGGAAACCC

TATATTAGGAAACTTTATGAAAAAAAGCTCCTTAAGGGAAAAGAAGAAAATCTCACTGGG

TTTCTAGAACCTGGGAACTTTGGAGAGAGTTTTAAAGCCATCAATAAGGCCTATGAGGAG

TATGTTTTATCTGTTGGGAATTTAGATGAGCGGATATTTCTTGGAGAGGATGCTGAGGAG

GAAATTGGGACTCTCTCAAGGTGTCTGAACGCTGGTTCAGGAACAGAGACTGCTGAAAGG

GTGCAGATGAAAAACATCTTACAGCAGCATTTTGACAAGTCCAAAGCACTTAGAATCTCC

ACACCACTAACTGGTGTTAGGTACATTAAGGAGAATAGCCCTTGTGTGACTCCAGTTTCT

ACAGCTACGCATAGCTTGAGTCGTCTTCACACCATGCTGACAGGCCTCAGGAATGCACCA

AGTGAGAAACTGGAACAGATTCTCAGGACATGTTCCAGAGATCCAACCCAGGCTATTGCT

AACAGACTGAAAGAAATGTTTGAAATATATTCTCAGCATTTCCAGCCAGACGAGGATTTC

AGTAATTGTGCTAAAGAAATTGCCAGCAAACATTTTCGTTTTGCGGAGATGCTTTACTAT

AAAGTATTAGAATCTGTTATTGAGCAGGAACAAAAAAGACTAGGAGACATGGATTTATCT

GGTATTCTGGAACAAGATGCGTTCCACAGATCTCTCTTGGCCTGCTGCCTTGAGGTCGTC

ACTTTTTCTTATAAGCCTCCTGGGAATTTTCCATTTATTACTGAAATATTTGATGTGCCT

CTTTATCATTTTTATAAGGTGATAGAAGTATTCATTAGAGCAGAAGATGGCCTTTGTAGA

GAGGTGGTAAAACACCTTAATCAGATTGAAGAACAGATCTTAGATCATTTGGCATGGAAA

CCAGAGTCTCCACTCTGGGAAAAAATTAGAGACAATGAAAACAGAGTTCCTACATGTGAA

GAGGTCATGCCACCTCAGAACCTGGAAAGGGCAGATGAAATTTGCATTGCTGGCTCCCCT

TTGACTCCCAGAAGGGTGACTGAAGTTCGTGCTGATACTGGAGGACTTGGAAGGAGCATA

ACATCTCCAACCACATTATACGATAGGTACAGCTCCCCACCAGCCAGCACTACCAGAAGG

CGGCTATTTGTTGAGAATGATAGCCCCTCTGATGGAGGGACGCCTGGGCGCATGCCCCCA

CAGCCCCTAGTCAATGCTGTCCCTGTGCAGAATGTATCTGGGGAGACTGTTTCTGTCACA

CCAGTTCCTGGACAGACTTTGGTCACCATGGCAACCGCCACTGTCACAGCCAACAATGGG

CAAACGGTAACCATTCCTGTGCAAGGTATTGCCAATGAAAATGGAGGGATAACATTCTTC

CCTGTCCAAGTCAATGTTGGGGGGCAGGCACAAGCTGTGACAGGCTCCATCCAGCCCCTC

AGTGCTCAGGCCCTGGCTGGAAGTCTGAGCTCTCAACAGGTGACAGGAACAACTTTGCAA

GTCCCTGGTCAAGTGGCCATTCAACAGATTTCCCCAGGTGGCCAACAGCAGAAGCAAGGC

CAGTCTGTAACCAGCAGTAGTAATAGACCCAGGAAGACCAGCTCTTTATCGCTTTTCTTT

AGAAAGGTATACCATTTAGCAGCTGTCCGCCTTCGGGATCTCTGTGCCAAACTAGATATT

TCAGATGAATTGAGGAAAAAAATCTGGACCTGCTTTGAATTCTCCATAATTCAGTGTCCT

GAACTTATGATGGACAGACATCTGGACCAGTTATTAATGTGTGCCATTTATGTGATGGCA

AAGGTCACAAAAGAAGATAAGTCCTTCCAGAACATTATGCGTTGTTATAGGACTCAGCCG

CAGGCCCGGAGCCAGGTGTATAGAAGTGTTTTGATAAAAGGGAAAAGAAAAAGAAGAAAT

TCTGGCAGCAGTGATAGCAGAAGCCATCAGAATTCTCCAACAGAACTAAACAAAGATAGA

ACCAGTAGAGACTCCAGTCCAGTTATGAGGTCAAGCAGCACCTTGCCAGTTCCACAGCCC

AGCAGTGCTCCTCCCACACCTACTCGCCTCACAGGTGCCAACAGTGACATGGAAGAAGAG

GAGAGGGGAGACCTCATTCAGTTCTACAACAACATCTACATCAAACAGATTAAGACATTT

GCCATGAAGTACTCACAGGCAAAT------ATGGATGCTCCTCCACTCTCTCCCTATCCA

TTTGTAAGAACAGGCTCCCCTCGCCGAATACAGTTGTCTCAAAATCATCCTGTCTACATT

TCCCCACATAAAAATGAAACAATGCTTTCTCCTCGAGAAAAGATTTTCTATTACTTCAGC

AACAGTCCTTCAAAGAGACTGAGAGAAATTAATAGTATGATACGCACAGGAGAAACTCCT

ACTAAAAAGAGAGGAATTCTTTTGGAAGATGGAAGTGAATCACCTGCAAAAAGAATTTGC

CCAGAAAATCATTCTGCCTTATTACGCCGTCTCCAAGATGTAGCTAATGACCGTGGTTCC

CAC

Rbl2-Mm

ATGGCATCTGGAGGCAACCAGTCGCCACCGCCTCCTCCA---------GCTGCTGCAGCC

AGCTCGGAGGAAGAGGAGGAGGATGGCGACGCCGCGGATCGCGCGCAGCCCGCGGGGTCC

CCGAGCCATCAGATCCAGCAGCGGTTCGAGGAGCTGTGCAGCCGCCTCAACATGGACGAG

GCGGCGCGCGCCGAGGCCTGGAGCAGCTACCGCAGCATGAGCGAGAGCTACACGCTGGAG

GGAAATGACCTTCATTGGTTAGCATGTGCCTTATATGTGGCTTGCAGAAAATCTGTTCCA

ACTGTGAGCAAAGGGACCGCTGAAGGAAACTATGTATCTTTAACCAGAATCCTTCGCTGT

TCGGAGCAGAGCCTAATTGAATTTTTTAACAAGATGAAGAAGTGGGAAGACATGGCAAAT

CTGCCCCCACATTTCCGAGAACGTACTGAAAGATTAGAAAGAAACTTCACTGTTTCTGCT

GTGATTTTTAAGAAATATGAACCCATTTTTCAAGACATTTTTAAATATCCCCAAGAAGAA

CAGCCTCGCCAGCAAAGAGGAAGAAAACAGAGGCGACAGCCCTGTACCACATCAGAAATT

TTCCATTTTTGCTGGGTGCTTTTTATATATGCGAAAGGGAACTTCCCCATGATTAGCGAT

GATCTGGTCAATTCCTACCATCTTCTGCTGTGCGCATTAGATTTAGTCTATGGAAATGCC

CTTCAGTGTTCTAACCGTAAAGAACTTGTGAACCCTAATTTTAAAGGCCTGTCCGAGGAC

TGTCACCCCAAGGACTCTAAAGCGTCCTCCGACCCGCCGTGTGTCATTGAGAAGCTGTGC

TCCTTACACGACGGTCTAGTGTTGGAGGCCAAGGGGATAAAGGAACACTTCTGGAAACCC

TATATTAGGAAACTGTTTGAGAAAAAGCTTCTCAAGGGGAAGGAAGAAAATCTTACTGGC

TTCCTGGAGCCCGGAAACTTTGGAGAGAGTTTTAAGGCCGTTAATAAGGCATATGAAGAA

TACGTGTTAGCCGCTGGGAATCTGGATGAGCGCGTATTCCTTGGTGAGGATGCTGAGGAG

GAAGTTGGGACTCTGTCTCGGTGTCTAAGTGCTGCCTCAGGTACAGAGAGTGCTGAACGG

ACGCAGATGAGAGACATCTTGCAGCAGCATCTTGACAAGTCTAAAGCACTTAGAGTCTGC

ACACCACTGACTGGCGTGAGGTATGTTCAGGAGAACAGCCCGTGTGTGACTCCCGTCTCC

ACAGCTGCACACAGCCTGAGCCGTCTTCACACCATGCTGTCCGGCCTCAGGAATGCACCC

AGTGAGAAGCTGGAGCGGATACTCAGGTCATGTTCCCGAGATCCAACTCAGGCTATCGCT

GACAGATTGAAAGAAATGTACGAAATATATTCTCAGCATTTCCAGCCAGATGAGAATTTT

AGTAATTGTGCTAAAGAAATTGCCAACAAACATTTTCGTTTTGCAGAAATGCTCTACTAT

AAAGTATTAGAGTCTGTTATTGAGCAAGAACAAAAAAGATTGGGAGACATGGATTTATCT

GGTGTTCTGGAGCATGACGCATTCCACAGGTCACTCTTGGCCTGCTGCCTTGAGGTGGTC

GCTTTTTCCCATAAGCCTCCTGGGAATTTTCCATTTATTGCTGAAATATTTGATGTACCA

CATTATCATTTTTATAAGGTAATTGAAGTATTTATTAGAGCAGAAGACGGTCTTTGCAGA

GAAGTGGTCAAACACCTCAATCAGATTGAAGAACAAATTTTAGACCATTTGGCATGGAAA

ACCAAGTCCCCACTGTGGGACAGAATTAGAGATAATGAAAACAGAGTCCCTACTTGTGAA

GAGGTCATGCCACCTCAAAACCTAGAGAGAACAGATGAAATTTACATCGCTGGCTCTCCC

TTAACCCCGAGAAGGGTGGGTGAAGTTCGTGCTGATGCTGGAGGACTTGGAAGAAGTATA

ACGTCTCCAACCACATTGTATGACAGGTACAGCTCCCCAACAGTCAGCACTACTAGAAGG

CGGCTATTCGAG---AATGATAGTCCCTCTGAAGGAAGCACATCTGGGCGCATCCCCCCA

CAACCCCTAGTCAACGCTGTCCCCGTGCAGAATGTACCTGGGGAGACTGTTTCTGTCACA

CCAGTTCCTGGACAGACCTTGGTCACCATGGCAACAGCCACTGTCACGGCCAACAATGGA

CAAACAGTGACCATTCCAGTCCAAGGTATTGCCAACGAAAATGGAGGGATAACCTTCTTC

CCAGTCCAAGTCAACGTTGGGGGCCAGGCCCAGGCTGTCGCTGGCTCTATCCAGCCCCTC

AGTGCTCAAGCACTGGCTGGAAGTCTGAGTTCCCAACAGGTGACAGGAACCACTTTGCAA

GTCCCTGGTCCGGTGGCCATTCAACAGATTTCCCCTGGTGGACAACAGCAGAACCCAGGC

CAGCCACTAACCAGCAGCAGTATCCGGCCGCGGAAGACTAGCTCCTTAGCGCTCTTCTTT

AGAAAGGTTTACTACTTAGCCGGTGTCCGCCTTCGAGATCTTTGTATAAAACTAGATATT

TCAGATGAACTGAGGAAAAAAATTTGGACCTGCTTTGAATTCTCTATAATCCAGTGCACC

GAACTTATGATGGACAGACATCTGGACCAGCTGTTGATGTGTGCCATTTATGTGATGGCA

AAGGTCACAAAAGAAGACAGGTCCTTCCAGAACATCATGCGTTGTTACAGAACTCAGCCA

CAGGCCCGGAGCCAGGTGTACAGAAGTGTCTTGATAAAAGGGAAAAGA------AGAAAC

TCTGGCAGCAGTGAGAGCAGAAGCCATCAGAATTCTCCAACCGAACTAAATACAGACAGA

GCCAGTAGAGATTCCAGCCCAGTGATGAGGTCAAACAGCACCCTACCAGTTCCACAGCCC

AGCAGTGCCCCTCCTACACCAACTCGACTCACGGGTGCCAGCAGTGACGTTGAAGAGGAG

GAACGAGGAGACCTCATTCAGTTCTACAACAACATCTATAGGAAGCAAATCCAAGCGTTT

GCCATGAAGTACTCGCAGGCAAACGCGCAGACGGACACTCCTCCCCTCTCTCCCTATCCA

TTTGTAAGAACAGGCTCCCCTCGCCGAGTACAGTTATCTCAAAGTCATCCTATCTACATT

TCCCCACATAACAACGAAGCAATGCCTTCTCCTCGAGAGAAGATTTTTTACTACTTCAGC

AACAGCCCATCAAAGAGACTGAGGGAAATCAACAGTATGATACGGACAGGAGAGACTCCA

ACTAAAAAGAGAGGGATTCTCTTGGACGACGGAAGTGAATCACCTGCAAAAAGAATCTGC

CCAGAGAATCACTCTGCTCTGTTACGTCGTCTCCAGGATGTGGCGAATGACCGAGGTTCA

CAG

Rbl2-Cf

ATGCCGTCGGGAGGCGACCAGTCGCCGCCGCCCCCGCCTCCCCCTCCGGCGGCGGCAGCC

TCGGATGAGGAGGAGGAAGACGACGGGGAGGCGGAGGACGCCGCGCAGCCGGCCCGGTCG

CCGGCCCCTCAGACCCAGCAGCGGTTCGACGAGCTGTGCAGCCGCCTCAACATGGACGAG

GCGGCGCGGGCCGAGGCCTGGGACAGCTACCGCAACATGAGCGAGAGCTACACGCTGGAG

GGAAATGATCTTCACTGGTTAGCATGTGCCTTATATGTGGCTTGCAGAAAATCTGTGCCA

ACTGTAAGCAAAGGGACAGTTGAAGGAAACTATGTATCTTTAACTAGAATCCTGAGATGT

TCAGAGCAGAGCTTAATTGAATTTTTTAACAAGATGAAGAAGTGGGAAGACATGGCAAAT

CTCCCCCCACATTTCAGAGAACGTACCGAGAGATTAGAAAGAAACTTCACTGTTTCAGCG

GTAATTTTTAAGAAATATGAACCCATTTTTCAGGACATTTTTAAATATCCTCAAGAGGAG

CAGCCCCGTCAACAGAGAGGAAGAAAACAGCGGCGACAGCCCTGTACCGTGTCTGAAGTT

TTCCATTTTTGTTGGGTGCTCTTTATATATGCAAAAGGTAATTTCCCCATGATTAGTGAT

GATTTGGTCAATTCTTATCATCTTCTGCTGTGTGCTTTGGACTTGGTTTATGGAAATGCC

CTTCAGTGTTCCAATCGTAAAGAATTGGTGAACCCTAATTTTAAAGGCCTGTCTGAAGAT

TTTCATGCTAAGGACTCTAAACCTTCCTCTGACCCACCTTGTGTCATTGAGAAACTCTGT

TCCTTACATGATGGTCTAGTTTTGGAAGCAAAGGGAATAAAGGAACATTTCTGGAAACCC

TATATTAGGAAACTTTATGAAAAAAAGCTCCTTAAAGGAAAAGAAGAAAATCTTACAGGG

TTTTTAGAACCTGGGAATTTTGGAGAGAGTTTTAAAGCCATCAATAAAGCCTATGAGGAG

TATGTTTTATCTGTTGGGAATTTAGATGAAAGGATATTTCTTGGAGATGATGCTGAAGAG

GAAATTGGGACTCTCTCAAAGTGTCTGAATTCTGGTTCAGGAACAGAGACTGCTGAAAGG

GTGCAGATGAAAAACATCTTGCAGCAGCACTTTGACAAGTCTAAAGCACTTAGAATCTCC

ACACCACTTACTGGTGTGAGGTACATGAAGGATAACAGCCCTTGTGTGACTCCAGTGTCT

ACAGCAACACACAGCTTAAGCCGTCTTCACACCATGCTAACAGGCCTCAGGAATGCACCA

AGTGAGAAACTGGAACAGATACTAAGGACATGTTCTAGAGATCCAACACAGGCCATTGCC

AACAGATTGAAAGAAATGTATGAAATATATTCTCAGCATTGCCAGCCAGATGAGGATTTC

AGTAAT---TCTAAAGAAATTGCCAGCAAACATTTTCGTTTTGCAGAAATGCTTTACTAT

AAAGTATTAGAATCTGTTATTGAACAGGAACAGAAAAGACTGGGAGACATGGATTTATCT

GGCATTCTGGAACAAGATGCATTCCATAGATCACTTTTAGCCTGCTGCCTTGAGGTCGTC

ACTTTTTCTTATAAGCCTCCTGGGAATTTTCCATTTATTACTGAAATATTTGATGTGCCA

CTTTATCATTTTTATAAGGTGATAGAAGTATTCATTAGAGCAGAAGATGGTCTTTGTAGA

GAAGTGGTAAAACACCTTAATCAGATTGAAGAACAGATCTTGGATCATTTGGCATGGAAA

CCAGAGTCTCCACTCTGGGACAGAATTAGAGACAATGAAAACAGAGTTCCTACTTGTGAA

GAGGTTATGCCACCCCAGAACCTGGAAAGAGCAGATGATATTTGTATTGCTGGATCTCCT

TTGACTCCCAGAAGGGTGAGTGAAGTTCGTGCGGATACTGGAGGACTTGGAAGAAGCATA

ACATCTCCAGCCACATTATATGATAGATACAGCTCCCCAACAGCCAGCTCTACCAGAAGG

CGGCTATTTGTTGAGAATGATAGCCCTTCTGATGGAGGGACACCTGGGCGCATTCCCCCA

CAACCCCTCGTCAATGCTGTCCCTGTGCAGAATGTATCTGGGGAGACTGTTTCTGTCACA

CCAGTTCCTGGACAGACTTTGGTCACTATGGCAACAGCCACTGTCACAGCCAACAATGGA

CAAACAGTGACCATTCCTGTACAAGGTATTGCGAATGAAAATGGAGGGATAACGTTCTTC

CCAGTCCAGGTCAATGTTGGAGGGCAGGCACAAGCCGTGACTGGCTCCATCCAGCCCCTC

AGTGCTCAGGCCCTGGCTGGAAGTTTGAGCTCTCAACAGGTGACAGGAACAACCTTGCAA

GTCCCTGGTCAGGTGGCCATTCAACAGATTTCCCCAGGTGGACATCAGCAGAAACAAGGC

CCACCTTTAACTGGCAGCAGTATTAGACCCAGGAAGACCAGCTCTTTATCACTCTTCTTT

AGAAAGGTTTACCACTTGGCAGGTGTCCGCCTTCGGGATCTTTGTGCTAAACTGGATATT

TCAGATGAACTGAGGAAAAAAATCTGGACCTGTTTTGAATTCTCCATAATTCAGTGTCCT

GAACTTATGATGGACAGACACCTGGACCAGCTGTTGATGTGTGCCATTTATGTGATGGCA

AAGGTCACAAAAGAAGATAAGTCCTTCCAGAATATTATGCGTTGTTACAGGACTCAGCCA

CAGGCCCGGAGCCAGGTATACAGAAGTGTTTTGATAAAAGGGAAAAGGAAAAGAAGAAAT

TCTGGCAGCAGTGATAGCAGAAGCCATCAGAATTCTCCAACAGAACTAAACAAAGACAGA

ACCAGCAGAGACTCCAGTCCTGTCATGAGGTCAAGCAGCACCTTGCCAGTCCCACAGCCT

AGCAGTGCCCCTCCGACACCTACTCGTCTCACAGGTGCCAACAGTGACATGGAAGAAGAA

GAGCGGGGAGACCTCATTCAGTTCTACAACAATATCTACATAAAACAAATTAAAACATTT

GCCATGAAGTACTCACAGGCAAACATA---ATGGATGCTCCTCCACTCTCTCCCTATCCG

TTTGTAAGAACAGGTTCTCCTCGCCGAATACAGCTGTCTCAAAACCATCCTGTCTACATT

TCTCCACATAAAAATGAAACAATGCTTTCTCCTCGAGAAAAGATTTTCTATTACTTCAGC

AACAGTCCTTCAAAGAGACTGAGAGAAATTAATAGTATGATACGGACAGGAGAAACTCCA

ACCAAAAAGAGAGGGATTCTTTTGGAAGATGGAAGCGAATCCCCTGCAAAAAGAATTTGC

CCAGAAAATCATTCTGCCTTATTACGCCGTCTCCAAGACGTAGCTAATGACCGAGGTTCT

CAC

Rbl2-Bt

ATGCCGTCGGGAGGCGACCAATCGCCGCCGCCCCCGCCTCCCCCTCCGGCGGCGGCGGCC

TCAGATGAGGAGGAGGAGGACGATGGCGAGGCGGAGGACGCCGCGCAGCCGTCCCGGTCG

CCGGCCCCTCAGACCCAGCAGCGGTTTGACGAGCTGTGCAGCCGTCTGAACATGGACGAG

GCGGCGCGGGCCGAGGCCTGGGAGAGCTACCGGAGCATGAGCGAGAGCTACACGCTGGAG

GGAAATGATCTTCATTGGTTAGCATGTGCCTTATATGTGGCTTGCAGAAAATCTGTGCCA

ACTGTAAGCAAGGGGACAGTTGAAGGAAACTATGTATCTTTAACTCGAATCCTGCGATGT

TCTGAGCAGAGCTTAATTGAATTCTTTAACAAGATGAAGAAATGGGAAGACATGGCCAAT

CTCCCTCCACATTTCAGAGAACGTACTGAGAGATTAGAAAGAAACTTCACTGTTTCTGCT

GTAATTTTTAAGAAATATGAACCCATCTTTCAGGACATTTTTAAATATCCTCAAGAAGAG

CAGCCGCGTCAGCAGAGAGGAAGAAAACAGCGGCGACAGCCCTGTACCGTGTCTGAAGTT

TTCCATTTTTGTTGGGTGCTTTTTATATATGCAAAAGGTAATTTTCCCATGATTAGTGAT

GATTTGGTTAATTCTTATCATCTTCTGCTGTGTGCTTTGGACTTAGTTTATGGAAATGCC

CTTCAATGTTCCAATCGTAAAGAACTTGTGAACCCTAATTTTAAAGGTCTATCTGAAGAT

TTTCATGCTAAGGATTCTAAACCTTCCTCCGACCCACCTTGCGTCATTGAGAAACTGTGT

TCCTTACATGATGGCTTAGTTTTGGAAGCAAAAGGAATAAAGGAACATTTCTGGAAACCT

TATATTAGGAAACTTTATGAAAAAAAGCTCCTTAAGGGAAAAGAAGAAAATCTGACTGGT

TTTTTAGAACCTGGGAATTTTGGAGAGAGTTTTAAAGCCATCAATAAAGCCTATGAGGAG

TATGTTTTATCTGTTGGGAATTTAGACGAGCGGATTTTTCTGGGCGAGGATGCTGAAGAG

GAAATTGGGACTCTCTCAAGGTGCCTGAACACTGGTTCAGGAGCAGAGACCGCTGAGAGG

GTGCAGATGAAAAACATCCTGCAGCAGCACTTCGACAAGTCTAAAGCACTTAGAGTCTCC

ACACCACTTACCGGTGTGAGGTACATTAAGGACAGCAGCCCTTGTGTGACTCCAGTTTCT

ACAGCTACACATAGCTTGAGTCGCCTTCACACCATGCTCACGGGCCTCAGGAATGCACCG

AGTGAGAGACTGGAACAGACCCTAAGGTCATGTTCCAGAGATCCAACCCAGGCTATTGCC

AACAGATTGAAAGAAATGTATGAGATATATTCTCAGCATTTCCAGTCAGAGGAGGATGGC

AGTAATTGTGCTAAAGATATTGCCAGCAAACATTTTCGTTTTGCAGAGATGCTTTACTAT

AAAGTATTAGAATCTGTTATTGAGCAGGAACAAAAAAGACTGGGAGACATGGATTTATCT

GGCATTCTGGAACAAGATGCATTCCACAGATCACTCTTGGCGTGCTGCCTTGAGGTCGTC

ACTTTTTCTTATAAGCCTCCTGGGAATTTTCCATTTATTACTGAAATATTCGATGTGCCA

CTTTATCATTTTTACAAGGTGATAGAAGTATTCATTAGAGCAGAAGATGGCCTTTGTAGA

GAGGTGGTAAAACACCTTAATCAGATTGAAGAACAAATCTTGGATCATTTAGCCTGGAAA

CCAGAGTCTCCGCTCTGGGACAGGATTAGAGACAATGAAAACAGAGTTCCCACATGCGAA

GAGGTCATGCCACCTCAGAACCTGGAAAGAGCAGATGAAATTTGTATTGCTGGCTCCCCT

CTGACTCCCAGAAGGGTGAGTGAAGTTCGTGCTGATTCTGGAGGACTTGGAAGAAGCATA

TCATCTCCAACCACGCTGTATGATAGGTACAGCTCCCCAACAGCCAGTTCTACCAGGAGG

CGGCTATTTGTGGAGAATGACAGCCCCACTGATGGAGGGACCCCCGGGCGCACTCCCCCG

CAGCCTCTAGTCAATGCTGTCCCCGTGCAGAATGTAGCTGGGGAGGCTGTATCTGTCACA

CCGGTTCCTGGACAGACCTTGGTCACCATGGCAACAGCCACAGTCACAGCCAACAATGGA

CAAACAGTGACCATTCCTGTACAAGGTATTGCCAATGAGAATGGAGGAATAACGTTCTTC

CCAGTCCAAGTCAATGTTGGGGGGCAGGCGCAAGCTGTGACCGGCTCCATCCAGCCCCTC

AGTGCTCAGGCCCTGGCTGGAAGCTTAAGCTCGCAACAGGTGACAGGAACAACCTTGCAA

GTCCCTGGTCAGGTGGCCATTCAACAGATTTCCCCTGGTGGACCACAGCAGAAACAAGGC

CTACCTTTAACCAGCAGCAGTATCAGACCCAGGAAGACAAGCTCTTTATCGCTTTTCTTT

AGGAAGGTTTATCACTTAGCAGGTGTCCGCCTTCGGGATCTTTGTGCTAAATTGGATATT

TCAGATGAACTGAGGAAAAAAATCTGGACCTGCTTTGAATTCTCCATAATTCAGTGTCCT

GAACTTATGATGGACAGACATCTGGACCAGTTGTTAATGTGTGCCATTTATGTGATGGCA

AAGGTCACAAAAGAAGACAAGTCCTTCCAGAATATTATGCGTTGCTATAGGACTCAGCCA

CAGGCCCGGAGCCAGGTGTATAGAAGTGTTTTGATAAAAGGGAAAAGGAGAAGAAGAAAT

TCTGGCAGCAGTGACAGCAGAAGTCATCAGAATTCTCCGACAGAACTAAACAAAGACAGA

ACCAGCAGAGACTCCAGTCCTGTCATGAGGTCCAGCAGCACCTTGCCAGTCCCACAGCCG

AGCAGCGCCCCTCCTACCCCGACTCGCCTCACGGGGGCCAACAGTGACGTGGAGGAGGAG

GAGAGGGGGGACCTCATCCAGTTCTACAACAACATCTATATCAGACAGATGAAAACGTTT

GCCATGAAGTACTCACAGGCAAATGCA---ATGGATGCTCCTCCACTCTCTCCCTATCCA

TTTGTAAGAACAGGCTCTCCTCGCCGAATACAGTTGTCTCAGAACCATCCTGTCTACATT

TCCCCGCATAAAAATGAAGCGATGCTTTCTCCTCGAGAAAAGATTTTCTACTACTTCAGC

AACAGCCCTTCAAAGAGACTGAGAGAAATTAACAGTATGATACGGACAGGAGAAACCCCA

ACCAAAAAGAGAGGGATCCTTTTGGAAGATGGAAGTGAATCACCTGCAAAAAGAATCTGC

CCAGAAAATCATTCTGCCTTATTACGTCGTCTCCAAGATGTAGCTAATGACCGGGGTTCC

CAC

Rbl2-Rn

ATGGCATCTGGAGGCAACCAGTCGTCACCTCCTCCTCCT---------GCTGCTGCAGCC

AGCTCAGAGGAAGAGGAGGAGGATGGCGACACCGCGGACCGGGCGCAGCCCGCAGGGTCC

CCAAGCCATCAGATCCAACAGCGGTTCGAAGAGCTGTGCAGCCGCCTTAACATGGACGAG

GCGGCACGGGCCGAGGCCTGGAGCAGCTACCGCAGCATGAGCGAGAGCTACACGCTGGAG

GGAAATGATCTCCATTGGTTAGCATGTGCCTTATATGTGGCTTGCAGAAAATCTGTTCCT

ACTGTGAGCAAAGGGACTGCTGAAGGAAACTATGTGTCTTTAACTAGAATCCTTCGATGT

TCTGAGCAAAGCCTAATTGAATTTTTTAACAAGATGAAGAAGTGGGAAGATATGGCAAAT

TTACCCCCACACTTCCGAGAACGAACCGAAAGATTAGAAAGAAACTTCACTGTCTCCGCT

GTGATCTTTAAGAAATATGAACCCATTTTTCAAGACATTTTTAAATATCCCCAAGAAGAA

CAACCTCGCCAGCAAAGAGGAAGAAAGCAGAGGCGACAGCCCTGTACCACATCTGAAATT

TTCCATTTTTGTTGGGTGCTTTTTATATACGCAAAAGGAAACTTCCCCATGATCAGTGAT

GATCTGGTCAATTCCTACCATCTTCTGCTGTGTGCACTAGATTTAGTTTATGGAAATGCC

CTACAATGCTCTAACCGGAAAGAACTTGTGAACCCTAATTTTAAAGGCGTGTCCGAGGAC

GGCCACCCCAGGGACTCTCACCCGTCCTCTGATCCACCGTGTGTCATTGAGAAGCTCTGC

TCCTTACACGACGGCCTAGTGCTGGAGGCAAAGGGGATAAAGCAGCATTTCTGGAAACCC

TACATTAGGAAGCTTTTTGAAAAGAAGCTCCTCAGGGGGAAGGAAGAAAATCTCACTGGC

TTCCTGGAGCCCGGGAACTTCGCAGAGAGCTTTAAGGCTGTTAATAAGGCGTATGAAGAA

TACGTGTTAGCCACTGGGAGTCTGGATGAGCGGATATTTCTTGGCGAGGATGCGGAGGAG

GAAGTTGGAACTTTCTCACGGTGTGTAAGTGCTGCCTCGGGCACAGAGAGTGCCGAACGA

ACGCAGATGAGAGACATCTTGCAGCAGCATCTTGACAAGTCTAAAACACTTAGAGTCTGC

AACCCACTGACTGGCGTGCGGTATGTTCAGGAGAACAGCCCTTGTGTGACTCCCGTCTCC

ACAGCAACACACAGCCTGAACCGTCTTCACACCATGCTGGCCGGCCTCAGGAATGCACCC

AGTGAGAAGCTAGAACAGATACTCAGGTCATGTTCCCGAGATCCGACTCGGGCTATCGCT

GACAGGTTGAGAGAGATGTATGAAATATATTCTCAGCATTTCCAGCCAGACGAGAATGTT

AGTAATTGTGCTAAAGAAATGGCCAACAAACACTTTCGTTTTGCAGAAATGCTTTACTAT

AAAGTATTAGAGTCTGTTATTGAGCAAGAACAGAAGAGATTGGGAGACATGGACTTATCT

GGTGTTCTGGAGCAAGATGCATTCCATAAGTCACTCCTGGCCTGCTGCCTTGAGGTGGTC

GCTTTTTCCTATAAGCCTCCTGGAAATTTCCCATTTATTGCTGAAATATTTGATGTACCA

CATTATCATTTTTATAAGGTAATAGAAGTATTTATTAGAGCAGAAGACGGTCTTTGCAGA

GAAGTGGTCAAACACCTCAATCAGATTGAAGAACAAATTTTAGACCACTTGGCATGGAAA

ACCAAGTCCCCACTGTGGGATAGAATTAGAGATAATGAAAACAGAGTCCCTACATGTGAA

GAGGTTACACCACCTCACAACCTAGAGAGAACAGATGAAATCTATATTGCTGGCTCTCCC

TTAACTCCGAGAAGGGTGGGTGAAGTTCGCACTGATGCTGGAGGACTTGGAAGAAGTGTG

ACATCTCCAACCACACTGTATGACAGGTACAGTTCCCCAACAGTCAGTACTACTAGAAGG

CGGCTATTTGAG---AGTGACAGTCCCTCTGAAGGAAGCACGGCTGGGCGCATCCCCCCA

CAACCCCTAGTCAATGCTGTCCCTGTGCAGAATGTATCTGGGGAGACTGTTTCTGTCACA

CCAGTTCCTGGACAGACCTTGGTCACCATGGCAACAGCCACTGTCACAGCCAACAATGGA

CAAACAGTGACCATTCCAGTACAAGGTATTGCCAACGAAAATGGAGGGATAACATTCTTC

CCAGTCCAAGTGAATGTTGGGGGCCAGGCACAGGCTGTGACTGGCTCTATCCAGCCCCTC

AGTGCTCAAGCGCTGGCTGGAAGTCTGAGTTCCCAACAGGTGACAGGAACAACTTTGCAA

GTCCCCGGTCCGGTGGCCATTCAACAAATTTCCCCTGGAGGACAACAGCAGAACCAAGGC

CAGCCGTTAACCAGCAGCAGTATCAGGCCTCGGAAGACTAGCTCCTTATCACTTTTCTTT

AGAAAGGTTTACTACTTAGCTGGTGTCCGCCTTCGAGACCTTTGTACAAAACTAGATATT

TCAGATGAACTGAGGAAAAAAATTTGGACCTGCTTTGAATTCTCTATAGTCCAGTGTCCT

GAACTTATGATGGACAGACATCTGGACCAGTTGTTAATGTGTGCCATTTACGTGATGGCA

AAGGTCACAAAAGAAGACAAGTCCTTCCAGAATATCATGCGCTGTTACAGGACTCAGCCA

CAGGCCCGGAGCCAGGTATACAGAAGTGTCTTGATAAAAGGGAAAAGA------AGAAAT

TCTGGCAGCTGTGAGAACAGAAGCCATCAGAATTCTCCAACAGAACTAAATACAGACAGA

GCCAGTAGAGACTCCAGTCCAGTTATGAGGTCAAACAGCACCTTACCAGTTCCACAGCCC

AGCAGTGCCCCTCCTACACCAACTCGACTCACAGGTGCCAACAGTGACATTGAAGAGGAG

GAAAGAGGAGACCTCATTCAGTTCTACAACAACATCTACAGGAAACAAATCCAAACGTTT

GCCATGAAGTACTCACAGGCAAACTCCCAAATGGACACCCCTCCCCTGTCTCCCTATCCG

TTTGTAAGAACGGGCTCCCCTCGCCGAGTACAGTTATCTCAAAGTCACCCTATCTACATT

TCCCCACATAAAAACGAAGCAATGCTTTCTCCTCGAGAGAAGATTTTTTACTACTTCAGC

AACAGCCCATCAAAGAGACTGAGAGAAATTAACAGTATGATACGGACAGGAGAGACTCCA

ACTAAAAAGAGAGGGATTCTCTTGGACGATGGAAGTGAATCGCCTGCAAAAAGAATTTGC

CCAGAAAATCATTCTGCTCTGTTACGTCGTCTCCAAGATGTAGCTAATGACCGAGGTTCA

CAC
